# Chemical Synthesis of Acybolins Reveals Their Antiproliferative Activity

**DOI:** 10.1021/acs.joc.6c01819

**Published:** 2026-09-19

**Authors:** Raju Hazra, Prabhakara R. Tharra, Jovita Mary, Ondřej Naar, Karel Souček, Jakub Švenda

**Affiliations:** † Department of Chemistry, Faculty of Science, 37748 Masaryk University, Brno 625 00, Czech Republic; ‡ Department of Cytokinetics, Institute of Biophysics of the Czech Academy of Sciences, Brno 612 00, Czech Republic; § International Clinical Research Center, St. Anne’s University Hospital, Brno 656 91, Czech Republic

## Abstract

An efficient synthetic access to the acybolin class of inducible secondary metabolites is being reported. The approach developed features coupling between the antibiotic (−)-bactobolin A and various acybolin side chains without the need for protecting groups. The biological evaluation of synthetic acybolins in human cancer cell cultures revealed their potent antiproliferative effects.

## Graphical abstract

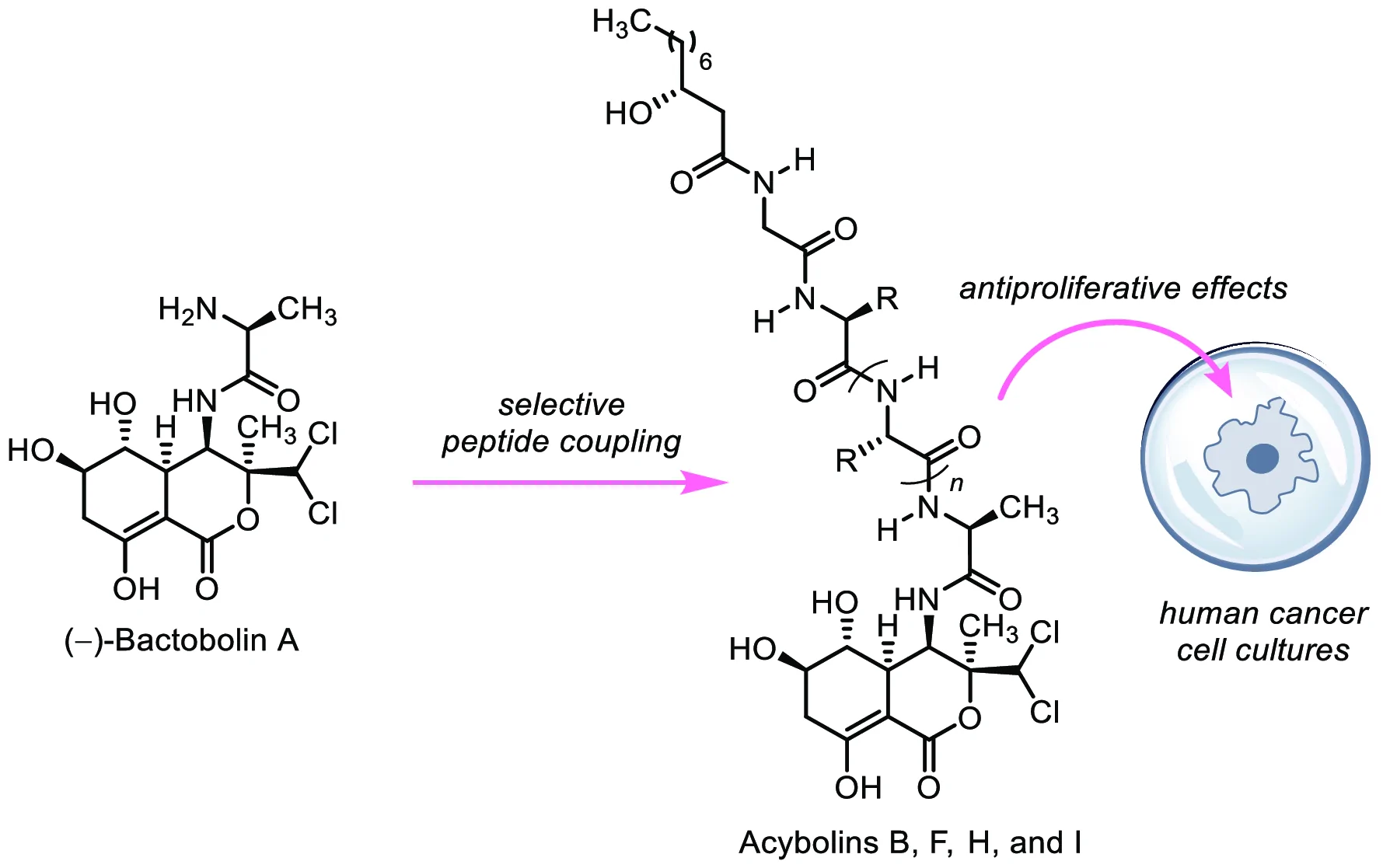

## 1 Introduction

Acybolins are a small group of complex secondary metabolites discovered in 2016 by the Seyedsayamdost laboratory.^1^ Not observed under normal laboratory growth conditions, acybolin biosynthesis was induced by exposure of *Burkholderia thailandensis* cultures to the antibiotic trimethoprim. Interestingly, acybolins were later detected in co-cultures of *B. thailandensis* with mammalian cells^2^ (co-culturing can induce secondary metabolism^3,4^ ). The exact biosynthetic pathway for acybolins is not known, but it shares the nonribosomal peptide/polyketide biosynthetic gene cluster with the bactobolin class of natural antibiotics; the latter has been characterized in significant detail.^5–7^ In *B. thailandensis*, a quorum-sensing-controlled transcriptional regulator ScmR was identified as the repressor and gatekeeper of the biosynthetic gene cluster for bactobolins and acybolins (among other secondary metabolites).^8,9^

The complex structures of acybolins, represented in Scheme 1 by acybolins F, B, H, and I (**4**–**7**), were determined using 2D NMR and/or mass spectrometry.^1^ The dichlorinated bicyclic scaffold embedded within corresponds to bactobolin A (**1**), a potent natural antibiotic.^10^ Bactobolin A (**1**) acts as a translation inhibitor by targeting the 50S subunit of the bacterial ribosome.^11,12^ The free amino group of the l-alanine residue of bactobolin A (**1**) establishes critical polar interactions within the tight binding site at the ribosome.^12^ More complex bactobolins containing l-alanine-based di- or tripeptide side chains (e.g., bactobolins B (**2**) and E (**3**) in Scheme 1) would not be congruent with this binding mode and were reported as significantly weaker or inactive antibiotics (e.g., MIC values against *Bacillus subtilis*: 1.56 μg/mL for bactobolin A (**1**) versus >200 μg/mL for bactobolin E (**3**)).^11,7^ Acybolins are at the end of this spectrum of complexity, as their side chains comprise tripeptides, tetrapeptides, or pentapeptides and uniformly feature a (*R*)-β-hydroxydecanoyl *N*-capping group (see Scheme 1). The acybolins, too, were reported to display weak antibacterial activity.^13^ While their function in bacterial communities is currently enigmatic, acybolins were suggested to be bivalent molecules:^1^ In part, potential prodrugs of the bactobolin class of ribosome inhibitors. In addition, the (*R*)-β-hydroxydecanoyl group present in acybolins is part of the *B. thailandensis*-encoded quorum-sensing signal (acyl homoserine lactones), which was speculated to confer a signaling function.^1^

**Scheme 1:**
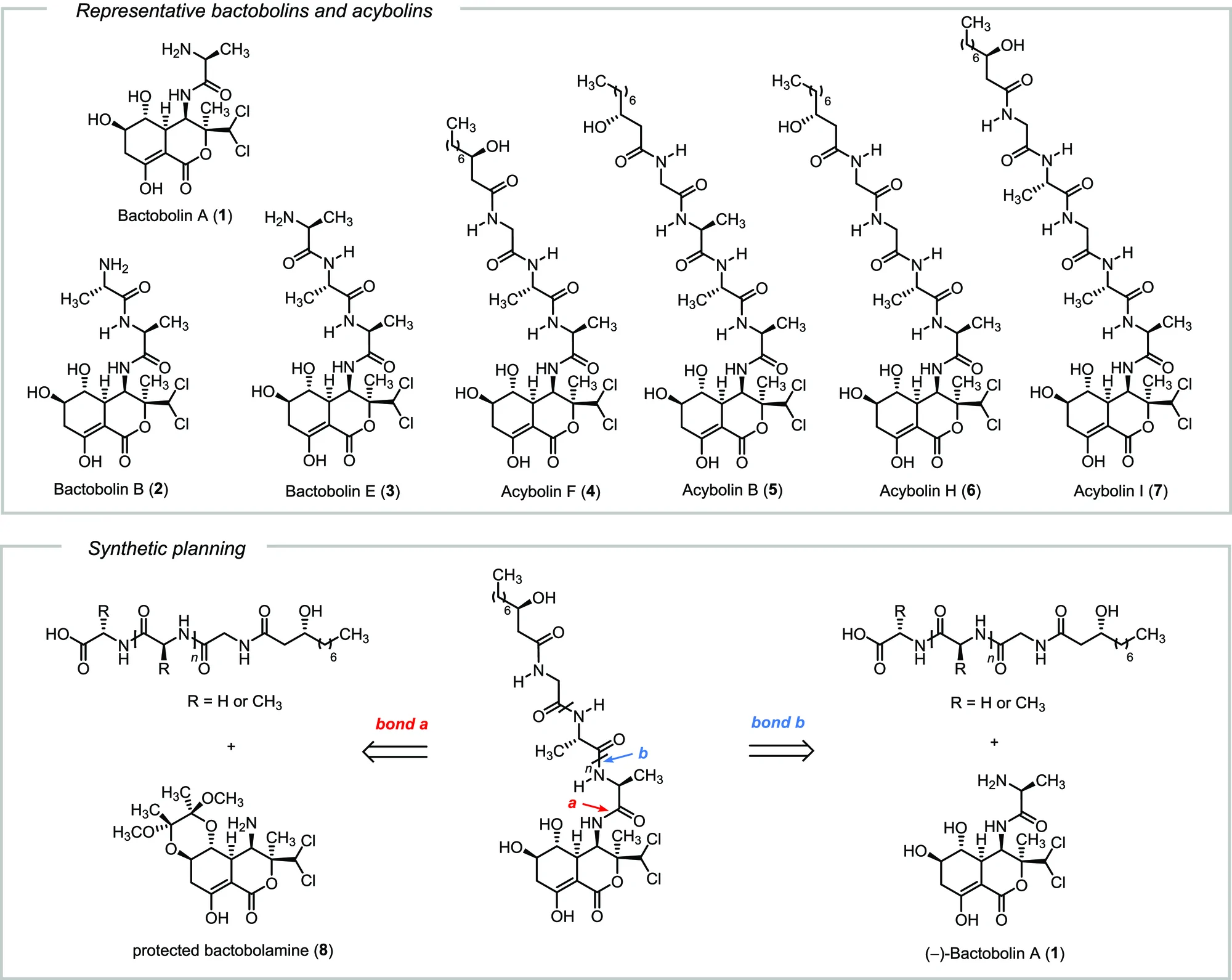
Structures of Selected Bactobolins and Acybolins (Top); Examined Retrosynthetic Disconnections (Bottom)

Probing these hypotheses experimentally requires access to acybolins. Isolation methods yielded single milligram or submilligram quantities of these complex metabolites. Here, we describe the first chemical synthesis of acybolins and report preliminary findings regarding their antiproliferative activity.

## 2 Results and Discussion

In planning a synthetic route to acybolins, we targeted disconnections of the peptidic side chains that maximize convergency (bond a, Scheme 1). This led to two principal components, bactobolamine and the *N*-acylated peptides. Protected bactobolamine (**8**) is a known intermediate in our synthesis of bactobolin A (**1**).^14^ It is also accessible semisynthetically by the Edman degradation of natural bactobolin A (**1**).^15^ The *N*-acylated peptide side chains can be readily simplified to β-hydroxydecanoic acid and individual amino acids.

In the forward direction, we prepared the β-hydroxydecanoic acid enantioselectively using cinchona alkaloid-catalyzed ketene–aldehyde cycloaddition^16^ between acetyl chloride and octanal (Scheme 2). Under the catalysis of *O*-trimethylsilyl quinidine (TMS-QD, **11a**) or *O*-trimethylsilyl quinine (TMS-QN, **11b**), the volatile β-lactone products *R*-**12** and *S*-**12** were obtained in good yields (78 and 96%, respectively, Scheme 2). β-Lactone hydrolysis and coupling to benzyl glycinate gave UV-active amides *R*-**13** and *S*-**13** suitable for HPLC analysis of enantiomeric purity (*R*-**13**: 95:5 er; *S*-**13**: 95:5 er). We processed these intermediates separately in three steps involving benzyl ester hydrogenolysis, coupling to *N*-protected amino acids or dipeptides, and benzyl ester hydrogenolysis (see the Supporting Information (SI) for details). The natural (derived from *R*-**13**) and epimeric (derived from *S*-**13**) side chains (**14**–**17**) would assess the potential effect of the remote stereocenter at β-hydroxydecanoyl (assigned by Mosher ester analysis in the isolation literature^1^).

**Scheme 2:**
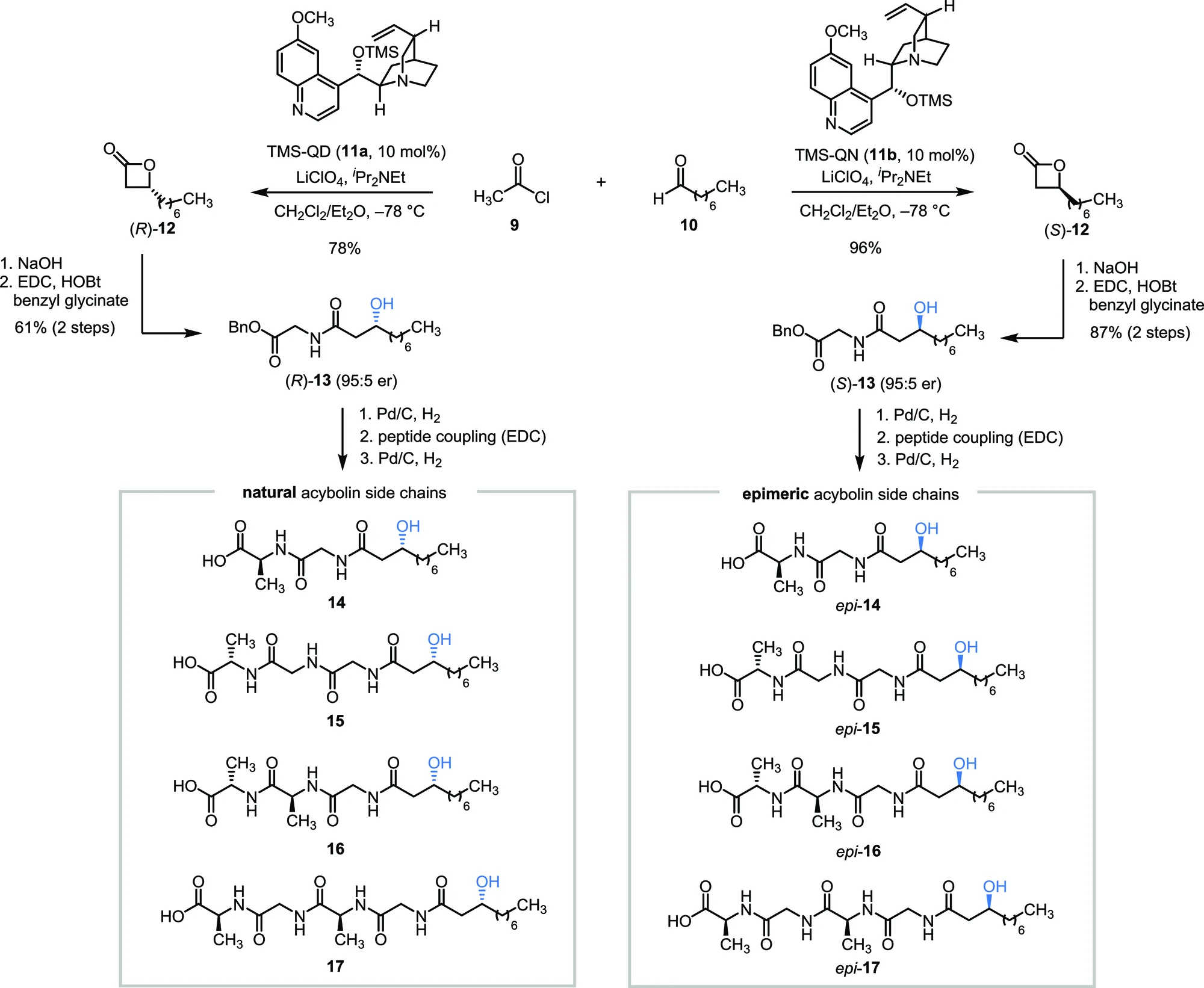
Enantioselective Preparation of the Acybolin Side Chains in Natural and Epimeric Configurations

With a general route to various acybolin side chains established, we explored their couplings to protected bactobolamine (**8**), which we prepared in 14 steps from quinic acid.^14^ Using the acybolin side-chain *epi*-**16** and bactobolamine **8**, we failed to obtain the expected product (masked 15′-*epi*-acybolin F) with various coupling agents (e.g., COMU, HATU, EDC, T3P). Even simpler *N*-Boc-protected l-Ala–l-Ala dipeptide failed to couple to bactobolamine **8** (not shown). This was remarkable considering the previously successful introduction of *N*-Boc-protected l-alanine onto the protected bactobolamine **8** en route to bactobolin A.^14^ These observations underscore the crowded nature of the primary amine within **8**.

As attempts to remove the bulky acetal from **8** led to significant decomposition, we pursued an alternative disconnection at the more distant peptide bond (bond b, Scheme 1). Focusing on bactobolin A (**1**), rather than bactobolamine (**8**), we were able to couple *N*-Boc l-Ala–l-Ala dipeptide using COMU^17^ to obtain, after deprotection, synthetic bactobolin E (**3**) (76% over 2 steps; Scheme 3).

**Scheme 3:**
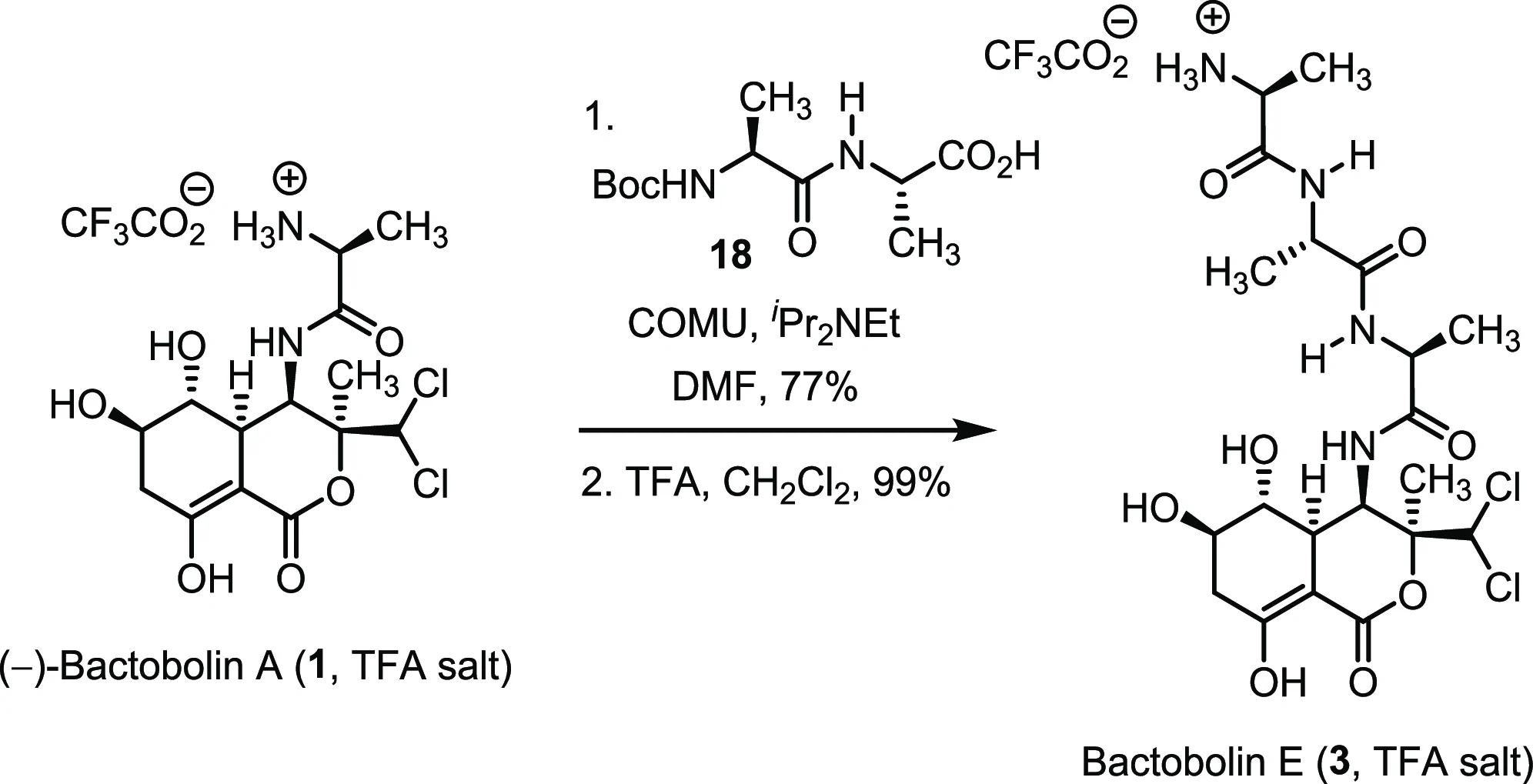
Conversion of Bactobolin A (**1**) to Bactobolin E (**3**)

However, the same conditions failed with the more complex acybolin side-chain *epi*-**16**. Eventually, we found that propanephosphonic anhydride^18^ (T3P, 2.0 equiv) in combination with sodium bicarbonate (4.0 equiv) in dimethylformamide promoted the coupling between the trifluoroacetate salt of bactobolin A (**1**) and side-chain *epi*-**16** at 25 °C to provide 19′-*epi*-acybolin B (19′-*epi*-**5**) in 43% yield. Upon replacing sodium bicarbonate with *N*,*N*-diisopropylethylamine (4.0 equiv, added in two portions), the yield increased to 62% (Scheme 4). These optimized conditions were then applied to couplings between the trifluoroacetate salt of bactobolin A (**1**) and all acybolin side chains prepared in Scheme 2, bearing the natural (*R*) and epimeric (*S*) configuration of the β-hydroxydecanoyl. A total of eight synthetic acybolins were prepared [acybolins B, F, H, and I (**4**–**7**) and their epimeric counterparts (*epi*-**4**–**7**)] in 53–62% yields and quantities sufficient to support biological evaluation (tens of milligrams). The NMR and MS data of synthetic acybolin B (**5**), but also of 19′-*epi*-**5**, agreed closely with those reported for the natural isolate (^1^H NMR Δ­(ppm): 0.01–0.05, ^13^C NMR Δ­(ppm): 1.0–1.8). Structures of the remaining acybolins F, H, and I are supported by the NMR data but could not be compared directly to the original isolates.^1^

**Scheme 4:**
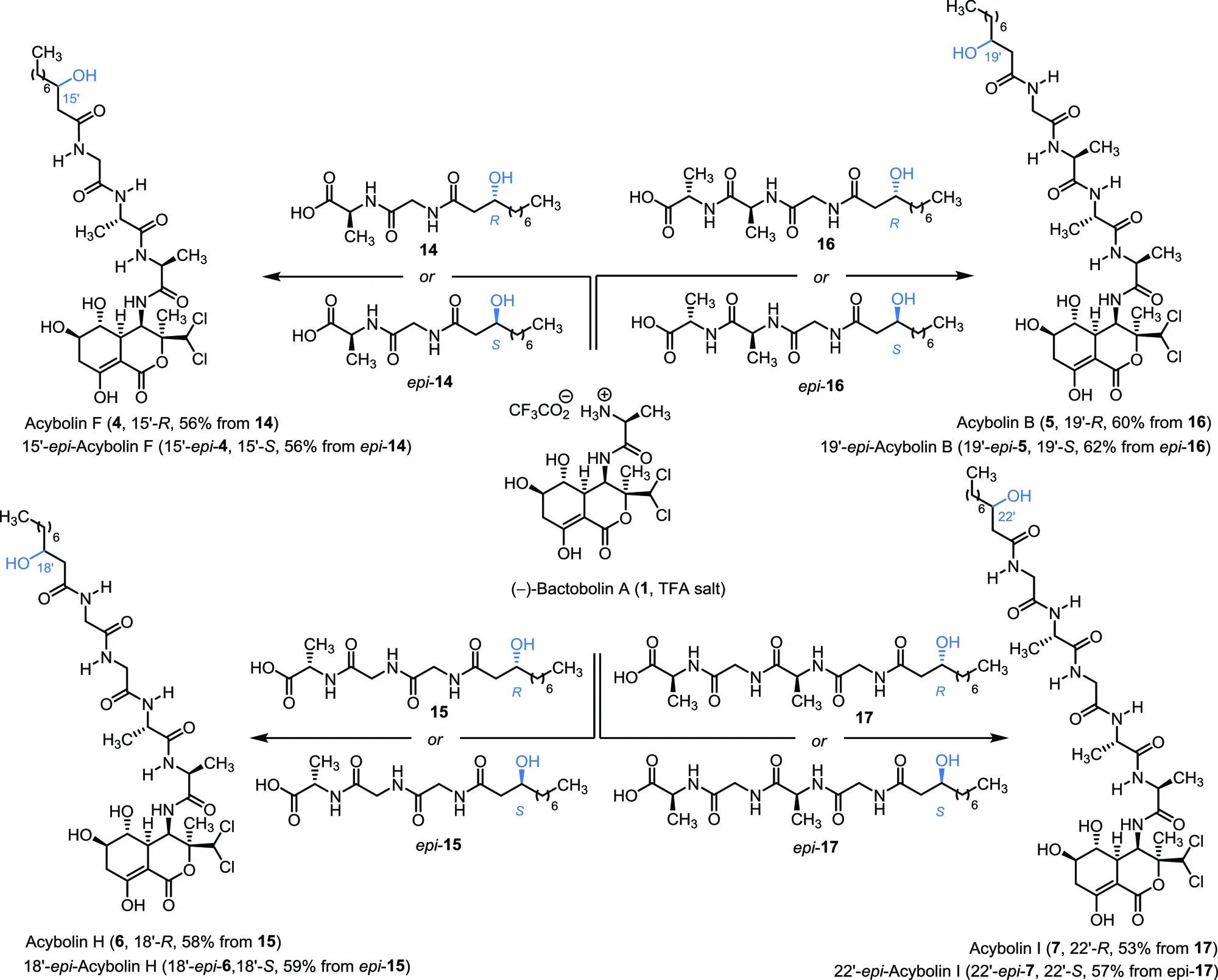
Single-Step Assembly of Acybolins F, H, B, I, and Their Epimeric Counterparts from (−)-Bactobolin A^a^

a Optimized coupling conditions: bactobolin A (**1**, TFA salt) (1 equiv), side chain (**14**–**17** or *epi*-**14**–**17**) (2.0 equiv), T3P (2.0 equiv), *i*-Pr_2_NEt (4.0 equiv), DMF, 0–23 °C, 24 h.

Acybolins, like the more complex bactobolins, were reported to exhibit poor antibacterial activity.^13^ This may be due to their elaborate side chains and impaired binding to the ribosome. Aware that acybolin biosynthesis was induced in the presence of mammalian cells,^2^ we investigated the effect of acybolins on four human cancer cell lines (HCC38, HeLa, MCF7, and MDA-MB-231). The CellTitter-Glo viability assay was used to monitor ATP-dependent metabolic activity in cells, and the synthetic bactobolin A (**1**) served as the positive control and a benchmark standard. Figure 1 shows select data from the MCF7 and MDA-MB-231 cell lines (data for all herein-prepared acybolins against the four cancer cell lines are provided in the SI).

**Figure 1:**
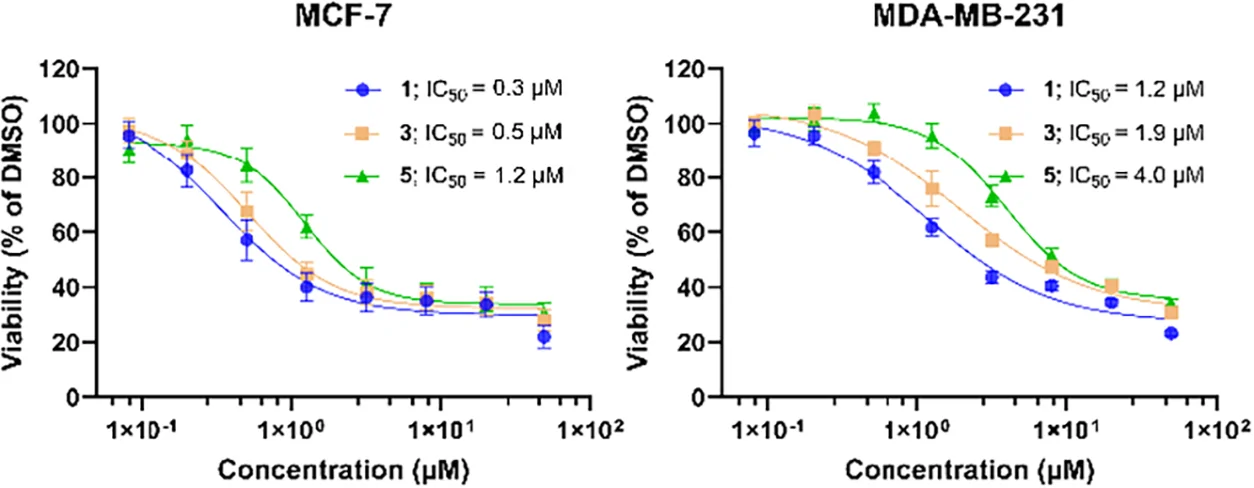
Antiproliferative activity and dose–response for bactobolin A (**1**), bactobolin E (**3**), and acybolin B (**5**) in cultures of MCF7 and MDA-MB-231 cells (see the SI for assay details).

As expected, bactobolin A (**1**) potently inhibited cell growth with IC_50_ values of 0.3 μM (MCF7) and 1.2 μM (MDA-MB-231). Notably, the acybolins were also active. The most potent acybolin B (**5**) displayed IC_50_ values of 1.2 μM (MCF7) and 4.0 μM (MDA-MB-231) after 48 h of cell treatment; the activity of 19′-*epi*-acybolin B (19′-*epi*-**5**) was similar (see the SI). Considering the outcome, we also tested synthetic bactobolin E (**3**), itself a poor antibiotic^7^ that lacks the β-hydroxydecanoyl capping group of acybolins but features the tripeptidic side chain. Recorded activity for bactobolin E (**3**) with IC_50_ values of 0.5 μM (MCF7) and 1.9 μM (MDA-MB-231) was close to bactobolin A (**1**).

Flow cytometry analysis of the cell cycle progression upon treatment with bactobolin A (**1**), bactobolin E (**3**), and 19′-*epi*-acybolin B (19′-*epi*-**5**) indicated a time-dependent redistribution of cells from G1 toward S and G2/M phases (see the SI). Since annexin V/propidium iodide analysis showed only modest cell death (≤20%; see the SI), the observed cellular response seems to be predominantly antiproliferative.

The activity of bactobolin E (**3**) and acybolin B (**5**) being comparable to that of bactobolin A (**1**) supports the acybolin prodrug theory.^1^ The acybolin-producing *Burkholderia* genus comprises aerobic Gram-negative bacilli, including human pathogens (e.g., melioidosis caused by *Burkholderia pseudomallei* ^19^), and it may be of interest to investigate whether acybolin biosynthesis induced in the presence of eukaryotic cells is part of a colonization strategy employed by pathogenic *Burkholderia*.

## 3 Conclusions

In summary, we have described the first chemical synthesis of the inducible secondary metabolites acybolins. The complex structures of acybolins B, F, H, I, and their epimeric counterparts (**4**–**7** and *epi*-**4**–**7**, respectively) were prepared in 17 steps from (−)-quinic acid (longest linear sequence) and quantities sufficient to support biological investigation. The final coupling between bactobolin A (**1**) and the various acybolin side chains proceeded without protecting groups, which has important practical implications for the semisynthetic production of acybolins using fermentation. Securing access to acybolins and establishing their antiproliferative activity provides the necessary framework for exploring these complex secondary metabolites in the future.

## Supplementary Material



## Data Availability

The data underlying this study are available in the published article and its Supporting Information.

## Notes

(a) Detailed synthetic procedures, (b) a complete set of dose–response curves, (c) quantification of apoptosis and cell cycle distribution, and (d) copies of NMR spectra and HPLC chromatograms (PDF)
