# Supplementary material for: Chemical Synthesis of Acybolins Reveals Their Antiproliferative Activity
Source: J Org Chem. 2026 Sep 19;91(39):13295–300. doi: 10.1021/acs.joc.6c01819 (PMC13640428; doi:10.1021/acs.joc.6c01819)
Supplement: Supplementary file 1 [file jo6c01819_si_001.pdf]

Supporting Information

Chemical Synthesis of Acybolins Reveals Their Antiproliferative Activity

Raju Hazra,[a] Prabhakara R. Tharra,[a] Jovita Mary,[a] Ondřej Naar,[b,c] Karel Souček,[b,c] and Jakub Švenda[a,c,*]

[a] Department of Chemistry, Faculty of Science, Masaryk University, Brno 625 00, Czech Republic

[b] Institute of Biophysics, Czech Academy of Sciences, Brno 612 65, Czech Republic

[c] International Clinical Research Center, St. Anne's University Hospital, Brno 656 91, Czech Republic

*Email: svenda@chemi.muni.cz

TABLE OF CONTENTS

Enantioselective preparations of *β*-lactones (*R*)-12 and (*S*)-12, and benzyl 3-hydroxydecanoyl glycinates (*R*)-13 and (*S*)-13 .... S4

General procedure for the convergent assembly of acybolins and *epi*-acybolins from (−)-bactobolin A........ S9

Acybolin F (4) S9

Synthesis of the side chain of acybolin F (14) S9

Assembly of acybolin F (4) S12

Acybolin B (5)........ S13

Synthesis of the side chain of acybolin B (16)........ S13

Assembly of acybolin B (5)........ S16

Acybolin H (6) S17

Synthesis of the side chain of acybolin H (15) S17

Assembly of acybolin H (6)........ S20

Acybolin I (7)........ S21

Synthesis of the side chain of acybolin I (17)........ S21

Assembly of acybolin I (7) S24

15'-*epi*-Acybolin F (15'-*epi*-4)........ S25

19'-*epi*-Acybolin B (19'-*epi*-5)........ S26

18'-*epi*-Acybolin H (18'-*epi*-6) S28

22'-*epi*-Acybolin I (22'-*epi*-7)........ S29

Synthesis of 19'-*epi*-acybolin B via bactobolin E (linear synthesis)........ S31

Synthesis of bactobolin E (3, TFA salt)........ S32

Comparison of ^{1}H and ^{13}C NMR data for natural acybolin B, synthetic acybolin B, and synthetic 19'-*epi*-acybolin B.........S34

Biological studies........ S37

Copies of HPLC chromatograms........ S44

References........ S44

Copies of NMR spectra........ S47

NMR spectra of acybolin F S55

NMR spectra of acybolin B S62

NMR spectra of acybolin H........ S69

NMR spectra of acybolin I S80

NMR spectra of *epi*-acybolins S91

NMR spectra of bactobolin E S104

Instrumentation

Proton nuclear magnetic resonance (^{1}H NMR) spectra were recorded using Bruker Avance 500 (500 MHz), or Bruker Avance 300 (300 MHz) NMR spectrometers at 23 °C. Proton chemical shifts are expressed in parts per million (ppm, δ scale) and are referenced to residual protium in the NMR solvents ($CDCl_3$: δ = 7.26 ppm (s), CD_3OD: δ = 3.31 ppm (quint), HDO = 4.79 ppm, $(CD_3)_2CO$: δ = 2.05 ppm (quint), and H_2O: δ = 1.56 ppm). Data are represented as follows: chemical shift, multiplicity (s = singlet, d = doublet, t = triplet, q = quartet, quint = quintet, dt = doublet of triplet, td = triplet of doublet, dd = doublet of doublet, ddd = doublet of doublet of doublet, dtd = doublet of triplet of doublet, qd = quartet of doublet, ABq = AB quartet, ABqd = AB quartet of doublet, m = multiplet and/or multiple resonances, app = apparent, br = broad), coupling constants (*J*) in Hertz, and integration. Carbon nuclear magnetic resonance (^{13}C NMR) spectra were recorded using Bruker Avance 500 (126 MHz), or Bruker Avance 300 (75 MHz) NMR spectrometers at 23 °C. Carbon chemical shifts are expressed in parts per million (ppm, δ scale) and are referenced to the carbon resonance of the NMR solvent ($CDCl_3$: δ = 77.2 ppm (t), CD_3OD: δ = 49.0 ppm (sept), $(CD_3)_2CO$: δ = 29.8 ppm (sept)). Fourier transform infrared (FTIR) spectra were obtained using an ALPHA Bruker FTIR spectrometer. Data are represented as follows: frequency of absorption (cm^{-1}), intensity of absorption (vs = very strong, s = strong, m = medium, w = weak, br = broad). Optical rotations were measured on the AUTOPOL IV polarimeter. High-resolution mass spectra were obtained on Agilent 6224 Accurate-Mass TOF LC-MS with dual electrospray/chemical ionization mode.

Materials

All reagents were obtained from commercial suppliers (Sigma-Aldrich, Acros Organics, Lach-Ner, Fluorochem, Enamine, TCI Chemical, BLD Pharmatech, Thermo Fisher Scientific) and were used without further purification. Quinidine (CAS: 56-54-2) was used as received to prepare catalyst **11a** (TMS-QD). Quinine (CAS: 130-95-0) was used as received to prepare catalyst **11b** (TMS-QN). Toluene and dichloromethane were dried and stored over activated 4Å molecular sieves; other solvents were used as received.

Experimental procedures

All reactions were performed in round-bottom flasks fitted with rubber septa under a positive pressure of argon, unless noted otherwise. All reactions were monitored by thin-layer chromatography (TLC) using aluminum plates pre-coated with silica gel (silica gel 60 F254, Merck) impregnated with a fluorescent indicator. TLC plates were visualized by exposure to ultraviolet light (λ = 254 nm) and/or by submersion in aqueous ceric ammonium molybdate (CAM), aqueous potassium permanganate ($KMnO_4$), ethanolic phosphomolybdic acid (PMA), ethanolic vanillin, or ethanolic *p*-anisaldehyde (ANIS) solutions followed by brief heating. All solutions were concentrated by rotary evaporation at 40 °C, unless noted otherwise. Flash-column chromatography (FCC) was performed using silica gel (60 Å, 230–400 mesh, Fluorochem).

Enantioselective preparations of *β*-lactones (*R*)-12 and (*S*)-12, and benzyl 3-hydroxydecanoyl glycinates (*R*)-13 and (*S*)-13

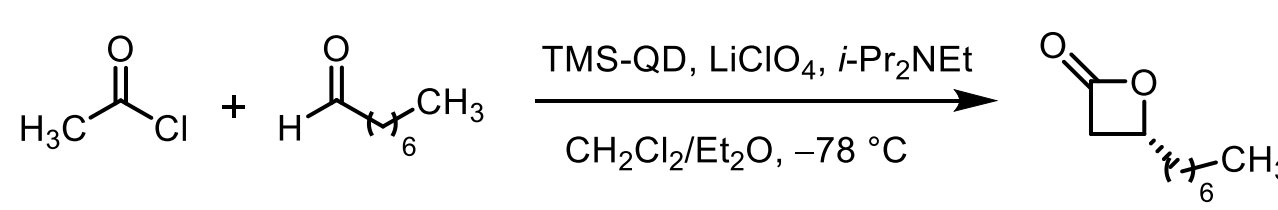

9 10 (*R*)-**12**

The procedure for the preparation of *β*-lactone, (*R*)-4-heptyloxetan-2-one ((*R*)-**12**), was adapted from the report by Zhu and coworkers.[1] Lithium perchlorate (8.30 g, 78.01 mmol, 2.0 equiv) was added to a solution of trimethylsilylquinidine (TMS-QD) catalyst **11a** (synthesized from quinidine) (1.54 g, 3.89 mmol, 0.1 equiv) in a 2:1 mixture of dichloromethane and diethyl ether (75 mL). The reaction mixture was cooled to –78 °C under an argon atmosphere. *N,N*-diisopropylethylamine (17 mL, 97.50 mmol, 2.5 equiv) was added, followed by octanal (**10**) (5.00 g, 38.99 mmol, 1 equiv). Then, a solution of acetyl chloride (**9**) (5.54 mL, 77.99 mmol, 2.0 equiv) in dichloromethane was added dropwise over 4 h. The reaction mixture was quenched with diethyl ether (50 mL) and filtered through silica gel eluting with diethyl ether. The filtrate was concentrated in vacuo, and the crude product was purified by flash chromatography (2−5% Et_2O in pentane) to obtain *β*-lactone (*R*)-**12** as a colorless oil (5.18 g, 78%).

(*R*)-**12**: $[\alpha]_{589}^{23}$ = +24.7 (*c* 0.72, $CHCl_3$). **TLC** (10% ethyl acetate in cyclohexane; $KMnO_4$): $R_f \approx$ 0.39. **^{1}H NMR** (500 MHz, chloroform-*d*): δ 4.49 (dtd, *J* = 7.4, 5.9, 4.3 Hz, 1H), 3.49 (dd, *J* = 16.3, 5.8 Hz, 1H), 3.05 (dd, *J* = 16.2, 4.3 Hz, 1H), 1.90 – 1.82 (m, 1H), 1.78 – 1.70 (m, 1H), 1.50 – 1.20 (m, 10H), 0.88 (app t, *J* = 7.1 Hz, 3H). **^{13}C{^{1}H} NMR** (126 MHz, chloroform-*d*): δ 168.5, 71.4, 43.0, 34.8, 31.8, 29.2, 29.2, 25.0, 22.7, 14.2. **FTIR** (neat) cm^{-1}: 2957, 2928, 2857, 1827, 1466, 1414, 1126, 947, 862, 815.

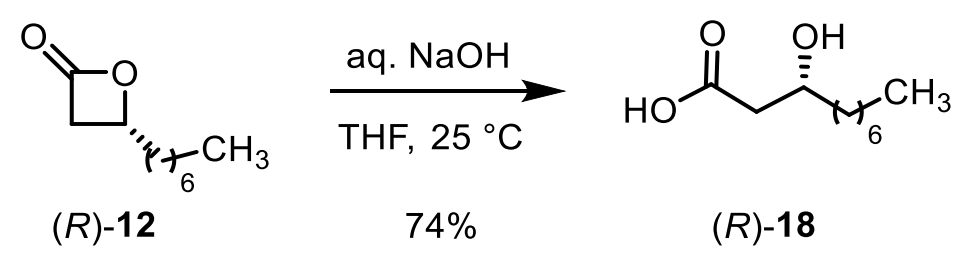

An aqueous solution of sodium hydroxide (868 mg, 25.54 mmol, 1.5 equiv) in water (8 mL) was added to a solution of *β*-lactone (*R*)-**12** (2.90 g, 17.03 mmol, 1 equiv) in tetrahydrofuran (30 mL) at 25 °C. The reaction mixture was stirred for 2 h. The mixture was diluted with water (50 mL), washed with ethyl acetate (20 mL) to remove any organic impurities. Aqueous layer was acidified by 1 M HCl solution (30 mL), extracted by ethyl acetate (2 × 100 mL), and dried over anhydrous magnesium sulfate. The dried solution was filtered and evaporated under reduced pressure to afford (*R*)-3-hydroxydecanoic acid[2] ((*R*)-**18**) as a white solid (2.38 g, 74%). The sign of optical rotation agrees, and the magnitude of optical rotation is reasonably close to the 1951 report of Lemieux and Giguère's ($[\alpha]_{589}^{25}$= –21 (*c* 2.5, $CHCl_3$))[3] and 2014 report of Vleeschouwer's ($[\alpha]_{589}^{25}$= –16.3 (*c* 1.08, CH_2Cl_2))[2] for the same (*R*)-enantiomer.

(*R*)-**18**: $[\alpha]_{589}^{23}$ = –15.4 (*c* 0.82, $CHCl_3$). **^{1}H NMR** (500 MHz, chloroform-*d*): δ 4.06 – 4.00 (m, 1H), 2.56 (dd, *J* = 16.5, 3.1 Hz, 1H), 2.46 (dd, *J* = 16.5, 9.0 Hz, 1H), 1.59 – 1.50 (m, 1H), 1.50 – 1.40 (m, 2H), 1.38 – 1.22 (m, 9H), 0.88 (app t, *J* = 7.1 Hz, 3H). **^{13}C{^{1}H} NMR** (126 MHz, chloroform-*d*): δ 177.8, 68.2, 41.3, 36.7, 31.9, 29.6, 29.3, 25.6, 22.9, 14.2. **FTIR** (neat) cm^{-1}: 3556, 3534, 2953, 2922, 2870, 2847, 1678, 1467, 1433, 1392, 1291, 1203, 1064, 934, 869. **HRMS** (APCI–) *m/z*: $[M - H]^-$ Calcd for $C_{10}H_{19}O_3$ 187.1340; Found 187.1343.

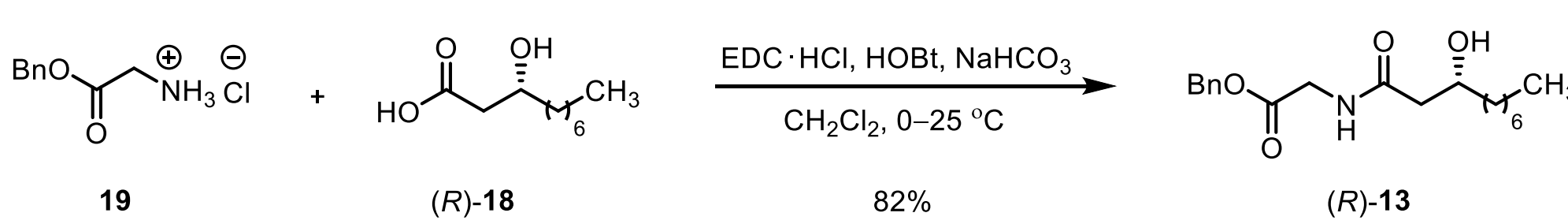

Benzyl glycinate salt **19** (1.17g, 5.84 mmol, 1.1 equiv) was added to a stirred solution of (*R*)-3-hydroxydecanoic acid ((*R*)-**18**) (1.00 g, 5.31 mmol, 1 equiv), sodium bicarbonate (1.34 g, 15.93 mmol, 3.0 equiv), HOBt (2.44 g, 15.93 mmol, 3.0 equiv), and EDC·HCl (2.47 g, 15.93 mmol, 3.0 equiv) in dichloromethane (40 mL) at 0 °C. The resulting reaction mixture was stirred for 2 h at 0 °C. After this time, the reaction mixture was allowed to reach 25 °C and stirred for 2 h. Then, water (50 mL) was added, the biphasic mixture was separated, and the aqueous phase was extracted with dichloromethane (40 mL). The combined organic phases were washed with a saturated aqueous solution of sodium bicarbonate (30 mL) and brine (30 mL). The washed organic phases were dried over anhydrous sodium sulfate, the dried solution was filtered, and the filtrate was concentrated in vacuo to provide crude (*R*)-**13**. The obtained residue was subjected to flash column chromatography purification (gradient elution with 40% ethyl acetate in cyclohexane) to afford (*R*)-**13** as a white amorphous solid (1.78 g, 82%). The enantiomeric purity of (*R*)-**13** was determined by HPLC (95:5 er).

(*R*)-**13**: $[\alpha]_{589}^{23}$ = −61.9 (*c* 1.0, CH_3OH). **TLC** (40% ethyl acetate in cyclohexane; PMA): $R_f \approx 0.34$. **^{1}H NMR** (500 MHz, chloroform-*d*): δ 7.39 – 7.32 (m, 5H), 6.39 (app t, J = 5.5 Hz, 1H), 5.19 (app s, 2H), 4.11 and 4.06 (ABqd, J_{AB} = 18.3 Hz, J = 5.4 Hz, 2H), 4.02 – 3.96 (m, 1H), 3.32 (br s, 1H), 2.43 (dd, J = 15.1, 2.7 Hz, 1H), 2.31 (dd, J = 15.1, 9.1 Hz, 1H), 1.58 – 1.48 (m, 1H), 1.48 – 1.39 (m, 2H), 1.37 – 1.22 (m, 9H), 0.88 (app t, J = 7.1 Hz, 3H). **^{13}C{^{1}H} NMR** (126 MHz, chloroform-*d*): δ 172.8, 170.1, 135.2, 128.8 (2 × C), 128.8, 128.6 (2 × C), 68.9, 67.5, 42.9, 41.5, 37.0, 31.9, 29.6, 29.4, 25.6, 22.8, 14.2. **FTIR** (neat) cm^{-1}: 3409, 3295, 2917, 2850, 1746, 1641, 1538, 1455, 1407, 1261, 1181, 1129. **HRMS** (APCI+) *m/z*: $[M + H]^+$ Calcd for $C_{19}H_{30}NO_4$ 336.2169; Found 336.2170.

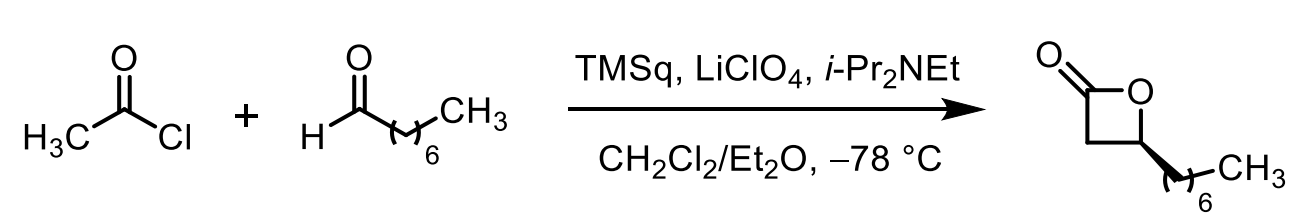

The procedure for the preparation of *β*-lactone, (*S*)-4-heptyloxetan-2-one ((*S*)-**12**), was adapted from the method reported by Zhu and coworkers.[1] $LiClO_4$ (996 mg, 9.36 mmol, 2.0 equiv) was added to a solution of the trimethylsilylquinine (TMS-QN) catalyst **11b** (synthesized from quinine) (186 mg, 0.47 mmol, 0.1 equiv) in a 2:1 mixture of dichloromethane and diethyl ether (9.0 mL).

The reaction mixture was cooled to –78 °C under argon atmosphere. *N,N*-diisopropylethylamine (2 mL, 11.70 mmol, 2.5 equiv) was added, followed by octanal (**10**) (600 mg, 4.68 mmol, 1 equiv). A solution of acetyl chloride (**9**) (0.7 mL, 9.36 mmol, 2.0 equiv) in dichloromethane (4 mL) was added dropwise over 4 h. The reaction mixture was quenched with diethyl ether (12 mL) and filtered through silica gel eluting with the same. The filtrate was concentrated in vacuo, and the crude product mixture was purified by flash chromatography (2–5% diethyl ether in pentane) to obtain *β*-lactone, (*S*)-4-heptyloxetan-2-one ((*S*)-**12**) as a colorless oil (764 mg, 96%). Both ^{1}H NMR and ^{13}C NMR matched exactly with (*R*)-**12**.

<u>(*S*)-**12**</u>: TLC (10% ethyl acetate in hexane; PMA): R_f = 0.3. **^{1}H NMR** (500 MHz, chloroform-*d*) δ: 4.49 (dtd, *J* = 7.3, 5.8, 4.2 Hz, 1H), 3.49 (dd, *J* = 16.3, 5.7 Hz, 1H), 3.04 (dd, *J* = 16.2, 4.2 Hz, 1H), 1.91 – 1.81 (m, 1H), 1.78 – 1.69 (m, 1H), 1.50 – 1.20 (m, 10H), 0.88 (app t, *J* = 7.0 Hz, 3H). **^{13}C{^{1}H} NMR** (126 MHz, chloroform-*d*) δ: 168.4, 71.4, 43.0, 34.8, 31.8, 29.2, 29.2, 25.0, 22.7, 14.1.

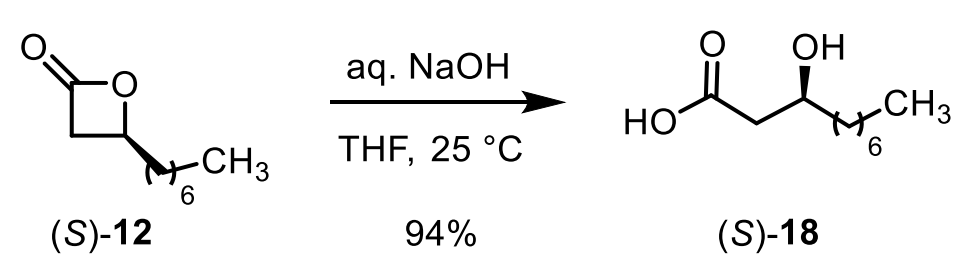

An aqueous solution of sodium hydroxide (264 mg, 25.54 mmol, 1.5 equiv) in water (2.0 mL) was added to a solution of *β*-lactone (*S*)-**12** (750 mg, 4.41 mmol, 1 equiv) in tetrahydrofuran (20 mL) at 25 °C. The reaction mixture was stirred for 2 h. The mixture was diluted with water (15 mL), washed with ethyl acetate (20 mL) to remove any organic impurities. Aqueous layer was acidified by 1 M HCl solution (15 mL), extracted by ethyl acetate (2 × 25 mL), and dried over anhydrous magnesium sulfate. The dried solution was filtered and evaporated under reduced pressure to afford (*S*)-3-hydroxydecanoic acid[2] ((*S*)-**18**) as a white solid (783 mg, 94%). The sign of optical rotation agrees, and the magnitude of optical rotation is reasonably close to the 2014 report of Vleeschouwer's ($[\alpha]_{589}^{25}$= +16.5 (*c* 1.0, CH_2Cl_2))[2] for the same (*S*)-enantiomer.

<u>(*S*)-**18**</u>: $[\alpha]_{589}^{23}$ = +15.0 (*c* 0.65, $CHCl_3$). **^{1}H NMR** (500 MHz, methanol-d_4): δ 4.00 – 3.94 (m, 1H), 2.44 (dd, *J* = 15.2, 4.9 Hz, 1H), 2.37 (dd, *J* = 15.3, 8.0 Hz, 1H), 1.55 – 1.41 (m, 3H), 1.40 – 1.24 (m, 9H), 0.90 (app t, *J* = 7.1 Hz, 3H). **^{13}C{^{1}H} NMR** (126 MHz, methanol-d_4): δ 175.7, 69.4, 43.2, 38.1, 33.0, 30.6, 30.4, 26.6, 23.7, 14.4.

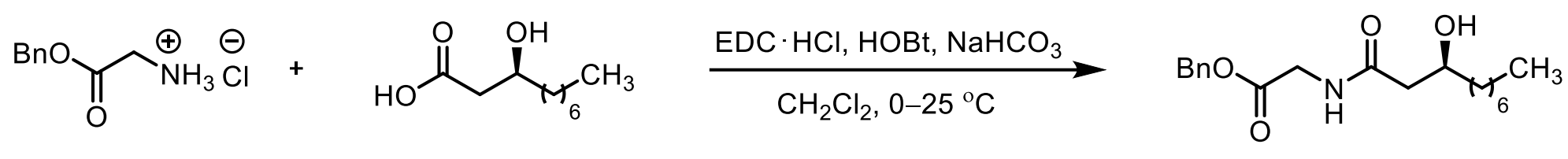

19 (*S*)-18 93% (*S*)-13

Benzyl glycinate salt **19** (235 mg, 1.17 mmol, 1.1 equiv) was added to a stirred mixture of (*S*)-3-hydroxydecanoic acid ((*S*)-**18**) (200 mg, 1.06 mmol, 1 equiv), sodium bicarbonate (267 mg, 3.18 mmol, 3.0 equiv), HOBt (487 mg, 3.18 mmol, 3.0 equiv), and EDC·HCl (609 mg, 3.18 mmol, 3.0 equiv) in dichloromethane (7.0 mL) at 0 °C. The resulting reaction mixture was stirred for 2 h at 0 °C. After this time, the reaction mixture was allowed to reach room temperature and stirred for 2 h. Then, water (20 mL) was added, the biphasic mixture was separated, and the aqueous phase was extracted with dichloromethane (2 × 25 mL). The combined organic phases were washed with a saturated aqueous solution of sodium bicarbonate (15 mL) and brine (15 mL). The washed organic phases were dried over anhydrous sodium sulfate, the dried solution was filtered, and the filtrate was concentrated in vacuo to provide crude (*S*)-**13**. The obtained residue was subjected to flash column chromatography purification (gradient elution with 10–50% ethyl acetate in cyclohexane) to afford benzyl ester (*S*)-**13** as a white amorphous solid (330 mg, 93%). The enantiomeric purity of (*S*)-**13** was determined by HPLC (95:5 er). Both ^{1}H NMR and ^{13}C NMR matched exactly with (*R*)-**13**.

(*S*)-**13**: **TLC** (50% ethyl acetate in cyclohexane; $KMnO_4$): $R_f \approx 0.41$. **^{1}H NMR** (500 MHz, chloroform-*d*): δ 7.40 – 7.32 (m, 5H), 6.39 (app t, *J* = 5.5 Hz, 1H), 5.19 (aap s, 2H), 4.11 and 4.06 (ABqd, J_{AB} = 18.4 Hz, *J* = 5.5 Hz, 2H), 4.02 – 3.96 (m, 1H), 3.31 (br s, 1H), 2.43 (dd, *J* = 15.1, 2.7 Hz, 1H), 2.31 (dd, *J* = 15.1, 9.1 Hz, 1H), 1.57 – 1.48 (m, 1H), 1.48 – 1.39 (m, 2H), 1.38 – 1.21 (m, 9H), 0.88 (app t, *J* = 7.1 Hz, 3H). **^{13}C{^{1}H} NMR** (126 MHz, chloroform-*d*): δ 172.8, 170.1, 135.2, 128.8 (2 × C), 128.8, 128.6 (2 × C), 68.9, 67.5, 42.9, 41.5, 37.0, 31.9, 29.6, 29.4, 25.6, 22.8, 14.2. **FTIR** (neat) cm^{-1}: 3413, 3296, 2919, 2851, 1757, 1747, 1657, 1643, 1564, 1549, 1194, 1185, 1079, 697. **HRMS** (APCI+) *m/z*: $[M + H]^+$ Calcd for $C_{19}H_{30}NO_4$ 336.2169; Found 336.2166.

General procedure for the convergent assembly of acybolins and *epi*-acybolins from (−)-bactobolin A

(−)-Bactobolin A (**1**) (TFA salt, 1 equiv) and the corresponding acybolin side chain (2.0 equiv) were added to a dry round-bottom flask equipped with a magnetic stirring bar. The flask was evacuated and backfilled with argon, then anhydrous *N,N*-dimethylformamide was added. The resulting homogeneous mixture was cooled to 0 °C in an ice bath, and the first portion of *N,N*-diisopropylethylamine (2.0 equiv) was added. After stirring for 5 min at 0 °C, propanephosphonic anhydride (T3P, 2.0 equiv) was added. After stirring for 20 min at 0 °C, the second portion of *N,N*-diisopropylethylamine (2.0 equiv) was added at 0 °C. The reaction mixture was allowed to slowly warm to 23 °C (gradual melting of the ice bath) and then stirred for 24 h at 23 °C. The reaction mixture was quenched by the addition of ice and then 10% aqueous solution of lithium chloride. The reaction mixture was extracted with ethyl acetate. Combined organic extracts were dried over anhydrous sodium sulfate, filtered, and concentrated under reduced pressure. The crude residue was purified by flash column chromatography (gradient elution with 1−5% methanol in dichloromethane) to afford the target acybolin and *epi*-acybolin.

Acybolin F (4)

Synthesis of the side chain of acybolin F (14)

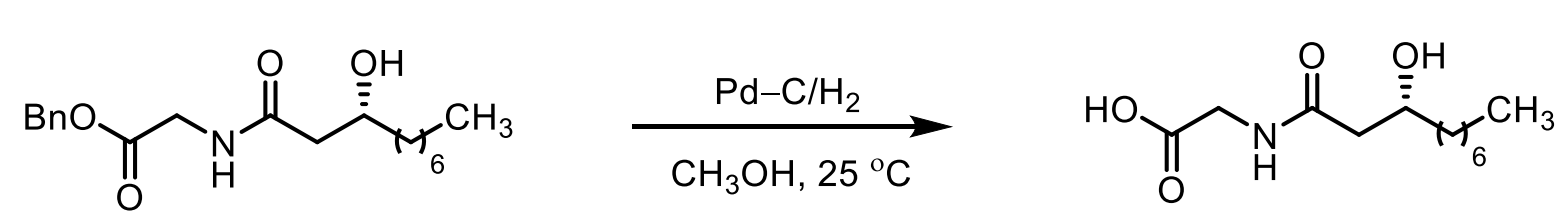

(*R*)-**13** 97% (*R*)-**19**

Palladium on charcoal (10% Pd basis; 147 mg) was added to a stirred solution of benzyl ester (*R*)-**13** (1.47 g, 4.38 mmol, 1 equiv) in methanol (65 mL) at 25 °C. After stirring the resulting suspension for 10 min at 25 °C, the flask was briefly evacuated, and a double-layered balloon filled with hydrogen gas was attached. The mixture was stirred vigorously for 1 h at 25 °C and then filtered through a celite pad. The celite was washed with methanol (3 × 50 mL). The filtrate was

concentrated in vacuo to afford the corresponding hydroxy acid (*R*)-**19** as a white amorphous solid (1.05 g, 97%).

<u>(*R*)-**19**:</u> $[\alpha]_{589}^{23}$= –72.3 (*c* 1.0, CH_3OH). **^{1}H NMR** (500 MHz, acetone-d_6): δ 7.50 (s, 1H), 3.98 (s, 1H), 3.96 (s, 1H), 3.94 – 3.90 (m, 1H), 2.43 (dd, *J* = 14.7, 3.7 Hz, 1H), 2.30 (dd, *J* = 14.6, 8.4 Hz, 1H), 1.50 – 1.43 (m, 3H), 1.37 – 1.23 (m, 9H), 0.88 (app t, *J* = 7.0 Hz, 3H). **^{13}C{^{1}H} NMR** (126 MHz, acetone-d_6): δ 173.3, 171.4, 69.2, 43.5, 41.3, 37.9, 32.6, 30.4, 30.1, 26.3, 23.3, 14.4. **FTIR** (neat) cm^{-1}: 3317, 2955, 2918, 2850, 1749, 1646, 1540, 1467, 1440, 1406, 1213, 1130. **HRMS** (APCI+) *m/z*: $[M + H]^+$ Calcd for $C_{12}H_{24}NO_4$ 246.1700; Found 246.1699.

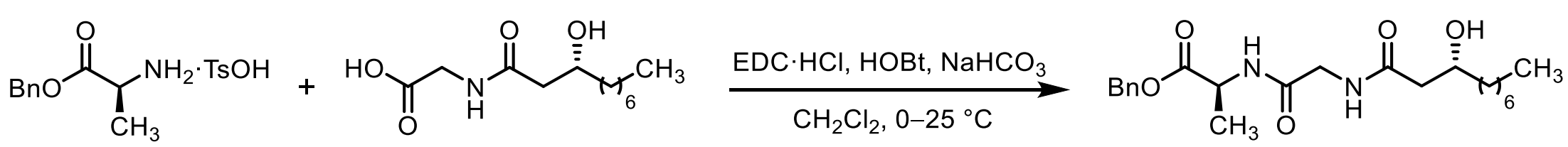

20 (*R*)-**19** 21

p-Tosylate salt of L-alanine benzyl ester **20** (250 mg, 0.71 mmol, 1 equiv) was added to a stirred solution of hydroxy acid (*R*)-**19** (192 mg, 0.78 mmol, 1.1 equiv), sodium bicarbonate (179 mg, 2.13 mmol, 3.0 equiv), HOBt (288 mg, 2.13 mmol, 3.0 equiv), and EDC·HCl (409 mg, 2.13 mmol, 3.0 equiv) in dichloromethane (10 mL) at 0 °C. The resulting reaction mixture was stirred for 2 h at 0 °C. After this time, the reaction mixture was allowed to reach room temperature and stirred for 2 h. The reaction mixture was quenched by adding water (10 mL). The aqueous phase was extracted with dichloromethane (30 mL), and the combined organic phases were washed with saturated aqueous sodium bicarbonate solution (20 mL) and brine (20 mL). The organic phases were dried over anhydrous sodium sulfate, the dried solution was filtered, and the filtrate was concentrated in vacuo to provide the crude benzyl ester **21**. The obtained residue was subjected to flash column chromatography purification (gradient elution with 80% ethyl acetate in cyclohexane) to afford benzyl ester **21** as a white amorphous solid (60 mg, 34%).

<u>**21**:</u> $[\alpha]_{589}^{23}$ = –29.9 (*c* 1.0, CH_3OH). **TLC** (5% methanol in dichloromethane; PMA) $R_f \approx 0.34$. **^{1}H NMR** (500 MHz, methanol-d_4): δ 7.37 – 7.34 (m, 4H), 7.34 – 7.29 (m, 1H), 5.17 and 5.15 (ABq, *J* = 12.3 Hz, 2H), 4.47 (q, *J* = 7.3 Hz, 1H), 4.00 – 3.94 (m, 1H), 3.92 and 3.87 (ABq, *J* = 16.8 Hz, 2H), 2.40 (dd, *J* = 13.9, 4.3 Hz, 1H), 2.32 (dd, *J* = 14.0, 8.6 Hz, 1H), 1.54 – 1.42 (m, 3H), 1.39 (d, *J* = 7.3 Hz, 3H), 1.37 – 1.26 (m, 9H), 0.90 (app t, *J* = 7.1 Hz, 3H). **^{13}C{^{1}H} NMR** (126 MHz, methanol-d_4): δ 174.9, 173.9, 171.5, 137.3, 129.6 (2 × C), 129.3, 129.1 (2 × C), 70.0, 67.9, 49.6,

44.7, 43.3, 38.3, 33.0, 30.6, 30.4, 26.6, 23.7, 17.4, 14.4. **FTIR** (neat) cm^{-1}: 3281, 3067, 2954, 2918, 2851, 1741, 1645, 1583, 1453, 1334, 1154, 729. **HRMS** (APCI+) *m/z*: $[M + H]^+$ Calcd for $C_{22}H_{35}N_2O_5$ 407.2540; Found 407.2537.

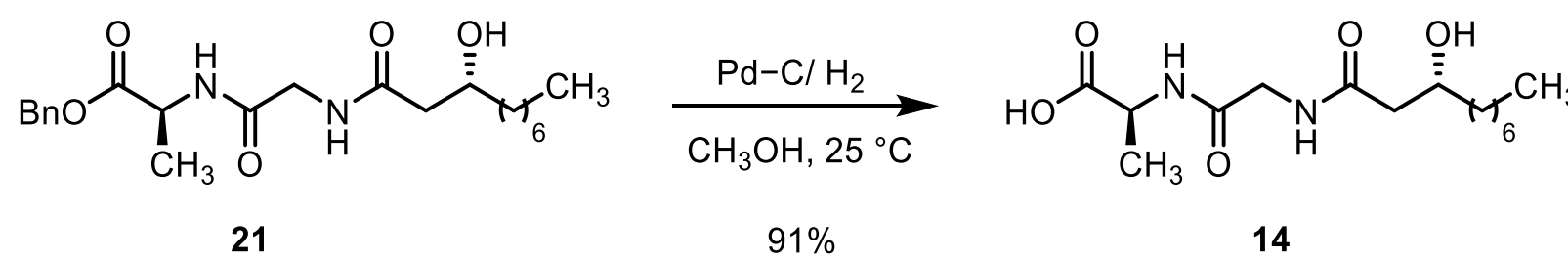

Palladium on charcoal (10% Pd basis; 6.0 mg) was added to a stirred solution of benzyl ester **21** (60 mg, 0.17 mmol, 1 equiv) in methanol (6 mL) at 25 °C. After stirring the resulting suspension for 10 min at 25 °C, the flask was evacuated, and a double-layered balloon filled with hydrogen gas was attached. The mixture was stirred vigorously for 1 h at 25 °C. The reaction mixture was then filtered through a Celite pad. The Celite was washed with methanol (3 × 15 mL). The filtrate was concentrated in vacuo to afford acybolin F side chain **14** as a white amorphous solid (40 mg, 86%).

<u>Acybolin F side chain **14**</u>: $[\alpha]_{589}^{23}$ = –16.0 (*c* 1.0, CH_3OH). **^{1}H NMR** (500 MHz, methanol-d_4): δ 4.41 (qd, *J* = 7.3, 2.1 Hz, 1H), 4.00 – 3.96 (m, 1H), 3.93 and 3.86 (ABq, *J* = 16.8 Hz, 2H), 2.40 (dd, *J* = 13.9, 4.2 Hz, 1H), 2.32 (dd, *J* = 13.9, 8.7 Hz, 1H), 1.55 – 1.42 (m, 3H), 1.40 (d, *J* = 7.3 Hz, 3H), 1.38 – 1.25 (m, 9H), 0.90 (app t, *J* = 7.1 Hz, 3H). **^{13}C{^{1}H} NMR** (126 MHz, methanol-d_4): δ 175.8, 174.9, 171.4, 70.0, 49.3, 44.7, 43.3, 38.4, 33.0, 30.6, 30.4, 26.6, 23.7, 17.7, 14.4. **FTIR** (neat) cm^{-1}: 3334, 3095, 2922, 2851, 1745, 1727, 1655, 1563, 1224, 954, 868. **HRMS** (APCI+) *m/z*: $[M + H]^+$ Calcd for $C_{15}H_{29}N_2O_5$ 317.2071; Found 317.2072.

Assembly of acybolin F (4)

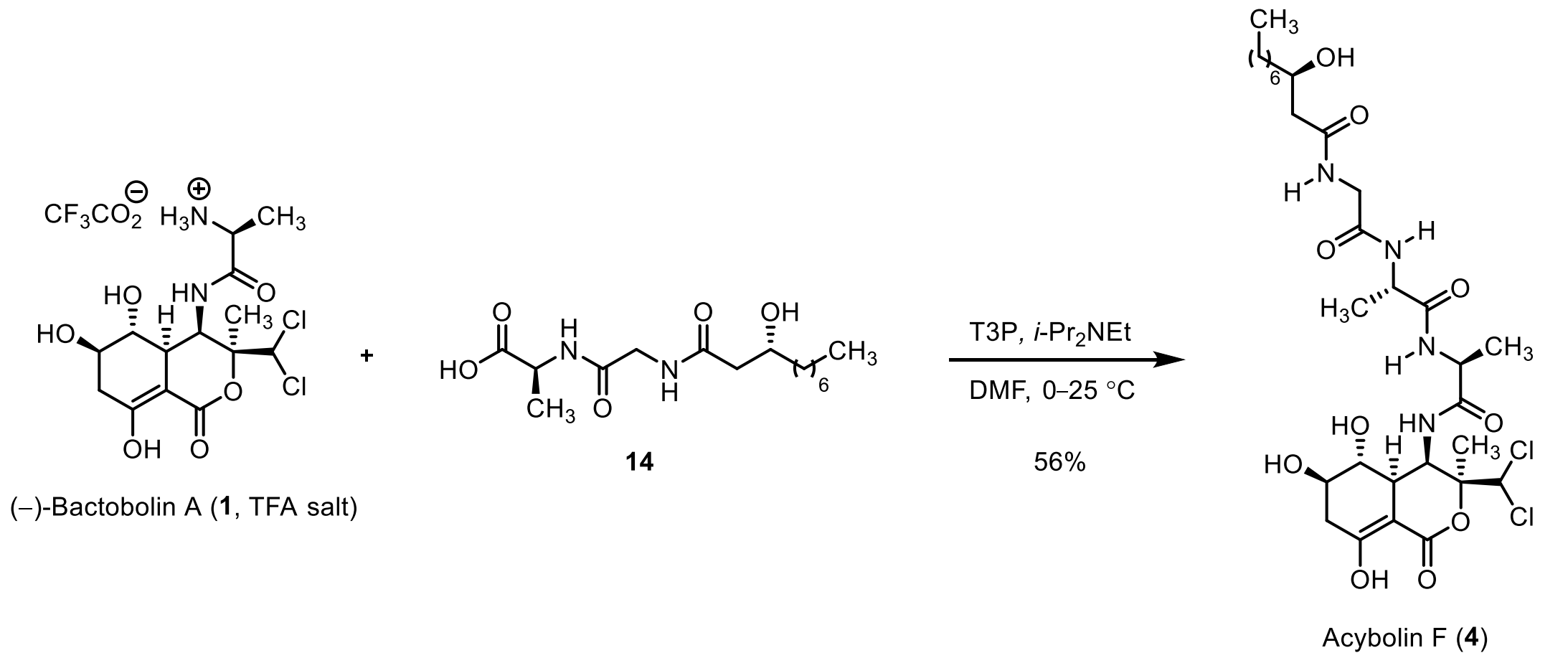

The general procedure described on page 9 was followed. The specific experiment used (–)-bactobolin A (**1**, TFA salt) (20 mg, 0.04 mmol, 1 equiv), acybolin F side chain **14** (25 mg, 0.08 mmol, 2.0 equiv), T3P reagent (24 µL, 0.08 mmol, 2.0 equiv), and *N,N*-diisopropylethylamine (28 µL, 0.16 mmol, 4.0 equiv) in *N,N*-dimethylformamide (2.0 mL). The quench was performed with 10% aqueous solution of lithium chloride (15 mL) and extraction with ethyl acetate (3 × 15 mL). Acybolin F (**4**) was obtained as a white amorphous solid (15 mg, 56%) after purification.

Acybolin F (**4**): $[\alpha]_{589}^{23}$ = –26.4 (*c* 0.42, CH_3OH). **TLC** (10% methanol in dichloromethane; UV, $KMnO_4$): $R_f \approx 0.45$. **^{1}H NMR** (500 MHz, methanol-*d*$_4$): δ 6.20 (s, 1H), 4.86 (d, *J* = 3.7 Hz, 1H), 4.35 (q, *J* = 7.3 Hz, 1H), 4.14 (q, *J* = 7.2 Hz, 1H), 4.03 – 3.97 (m, 1H), 3.92 – 3.78 (m, 3H), 3.21 (app t, *J* = 9.4 Hz, 1H), 2.91 (app dt, *J* = 9.2, 2.6 Hz, 1H), 2.80 (dd, *J* = 18.7, 6.5 Hz, 1H), 2.49 (dd, *J* = 14.0, 4.6 Hz, 1H), 2.42 (ddd, *J* = 18.9, 10.2, 2.6 Hz, 1H), 2.37 (dd, *J* = 14.1, 8.2 Hz, 1H), 1.68 (s, 3H), 1.54 – 1.47 (m, 2H), 1.41 (d, *J* = 7.3 Hz, 3H), 1.35 (d, *J* = 7.3 Hz, 3H), 1.33 – 1.28 (m, 10H), 0.90 (app t, *J* = 7.0 Hz, 3H). **^{13}C{^{1}H} NMR** (126 MHz, methanol-*d*$_4$): δ 176.6, 176.1, 175.3, 175.1, 172.1, 170.4, 90.9, 87.0, 76.2, 72.3, 70.0, 69.9, 51.3, 50.9, 47.9, 44.6, 44.0, 40.5, 38.3, 38.0, 33.0, 30.7, 30.4, 26.6, 23.7, 19.3, 17.6, 17.5, 14.4. **FTIR** (neat) cm^{-1}: 3294, 2927, 2855, 1650, 1536, 1232, 1125, 1069, 795. **HRMS** (APCI+) *m/z*: $[M + H]^+$ Calcd for $C_{29}H_{47}Cl_2N_4O_{10}$ 681.2664; Found 681.2658.

Acybolin B (5)

Synthesis of the side chain of acybolin B (16)

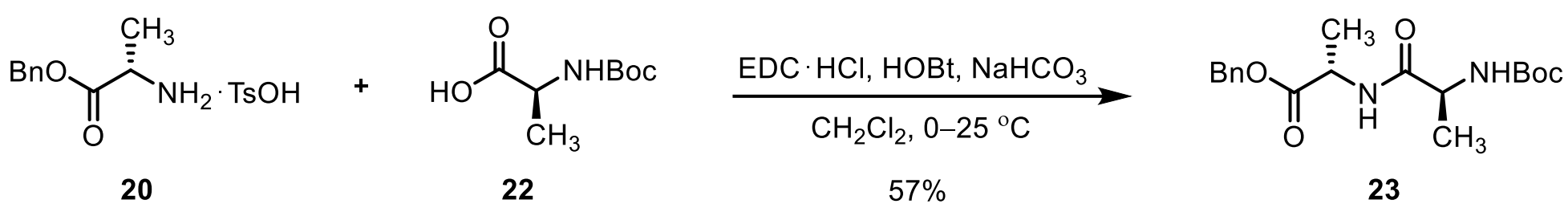

N-Boc-L-alanine (**22**) (0.68 g, 3.62 mmol, 1 equiv) was added to a stirred solution of *p*-toluenesulfonate salt of L-alanine benzyl ester (**20**) (1.40 g, 3.98 mmol, 1.1equiv), HOBt (1.47 g, 10.86 mmol, 3.0 equiv), and EDC·HCl (2.08 g, 10.86 mmol, 3.0 equiv) in dichloromethane (15 mL) at 25 °C. The resulting reaction mixture was stirred for 10 min, followed by the addition of *N*,*N*-diisopropylethylamine (1.26 mL, 7.24 mmol, 2.0 equiv). The reaction mixture was allowed to stir at 25 °C for 24 h. Then, 10% aqueous solution of citric acid was added with stirring for an additional 5 min. The reaction mixture was transferred to a separatory funnel and diluted with ethyl acetate (20 mL). The aqueous phase was separated, and the organic phase was washed sequentially with a 10% aqueous solution of citric acid (13 mL), a saturated aqueous solution of sodium bicarbonate (30 mL), and brine solution (30 mL). The organic phase was dried over anhydrous sodium sulfate and concentrated in vacuo. The obtained residue was subjected to flash-column chromatography purification (gradient elution with 40% ethyl acetate in cyclohexane) to afford protected dipeptide **23** as a white amorphous solid (730 mg, 57%).

23: **TLC** (60% ethyl acetate in cyclohexane; PMA): $R_f \approx 0.32$. **^{1}H NMR** (500 MHz, chloroform-*d*): δ 7.41 – 7.29 (m, 5H), 6.63 (d, *J* = 5.8 Hz, 1H), 5.19 and 5.15 (ABq, *J* = 12.3 Hz, 2H), 5.01 (br s, 1H), 4.61 (app quint, *J* = 7.2 Hz, 1H), 4.23 – 4.04 (m, 1H), 1.44 (s, 9H), 1.41 (d, *J* = 7.2 Hz, 3H), 1.34 (d, *J* = 7.1 Hz, 3H). **^{13}C{^{1}H} NMR** (126 MHz, chloroform-*d*): δ 172.7, 172.3, 155.6, 135.5, 128.8 (2 × C), 128.6, 128.3 (2 × C), 80.3, 67.3, 50.1, 48.3, 28.4 (3 × C), 18.5, 18.4. **FTIR** (neat) cm^{-1}: 3307, 3066, 2979, 1741, 1659, 1520, 1454, 1366, 1247, 1069, 749. **HRMS** (APCI+) *m/z*: $[M + H]^+$ Calcd for $C_{18}H_{27}N_2O_5$ 351.1914; Found 351.1911.

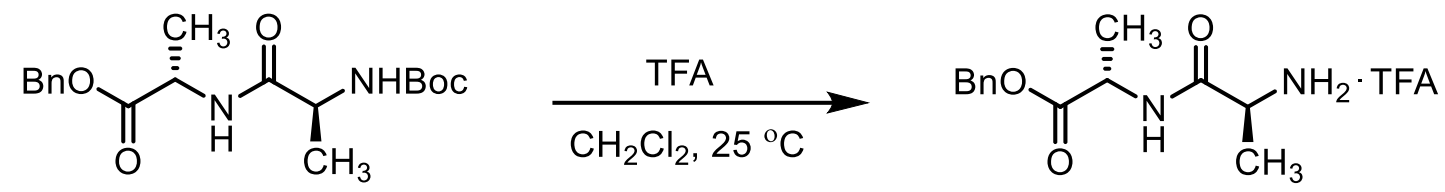

23 65% **24**

A solution of trifluoracetic acid in dichloromethane (20% by volume, 16 mL) was added to a stirred solution of protected dipeptide **23** (553 mg, 1.57 mmol, 1 equiv) in dichloromethane (16 mL) at 25 °C. The reaction mixture was allowed to stir for 16 h at 25 °C. Then, the reaction mixture was concentrated in vacuo under reduced pressure. The residue was successively concentrated from diethyl ether and dichloromethane. The obtained residue was subjected to flash column chromatography purification (elution with 10% methanol in dichloromethane) to afford the trifluoroacetate salt **24** as a colorless sticky solid (356 mg, 65%).

__24:__ **^{1}H NMR** (500 MHz, chloroform-*d*): δ 7.81 (br s, 1H), 7.33 – 7.27 (m, 5H), 5.14 and 5.07 (ABq, *J* = 12.2 Hz, 2H), 4.48 – 4.43 (app quint, *J* = 7.1 Hz 1H), 4.14 (br s, 1H), 1.41 (br s, 3H), 1.36 (d, *J* = 7.0 Hz, 3H). **^{13}C{^{1}H} NMR** (126 MHz, chloroform-*d*): δ 172.5, 170.2, 135.3, 128.7 (2 × C), 128.6, 128.3 (2 × C), 67.5, 49.5, 48.9, 17.0, 16.9. **FTIR** (neat) cm^{-1}: 3234, 3070, 3036, 2990, 2945, 1737, 1666, 1558, 1456, 1388, 1344, 1200, 1135, 1051, 990. **HRMS** (APCI+) *m/z*: [M – TFA + H]$^+$ Calcd for $C_{13}H_{19}N_2O_3$ 251.1390; Found 251.1388.

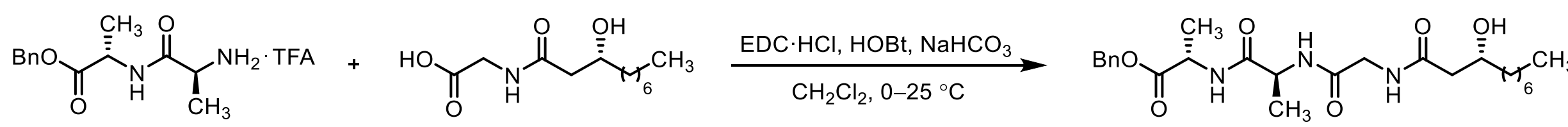

24 (*R*)-**19** 81% **25**

Trifluoroacetate salt **24** (356 mg, 0.98 mmol, 1.2 equiv) was added to a stirred solution of hydroxy acid (*R*)-**19** (200 mg, 0.81 mmol, 1 equiv), sodium bicarbonate (205 mg, 2.44 mmol, 3.0 equiv), HOBt (330 mg, 2.44 mmol, 3.0 equiv), and EDC·HCl (469 mg, 2.44 mmol, 3.0 equiv) in dichloromethane (15 mL) at 0 °C. The resulting reaction mixture was stirred for 2 h at 0 °C. Then, the reaction mixture was allowed to reach room temperature and stirred for 2 h. Then, water (20 mL) was added. The biphasic mixture was separated, and the aqueous phase was extracted with dichloromethane (3 × 25 mL). The combined organic phases were washed with a saturated aqueous solution of sodium bicarbonate (25 mL) and brine (25 mL). The organic phases were dried over anhydrous sodium sulfate, the dried solution was filtered, and the filtrate was concentrated in vacuo. The obtained residue was subjected to flash column chromatography purification (gradient

elution with 8% methanol in dichloromethane) to afford benzyl ester **25** as a white amorphous solid (315 mg, 81%).

<u>**25**:</u> $[\alpha]_{589}^{23} = -54.3$ (*c* 1.0, CH_3OH). **TLC** (5% methanol in dichloromethane; PMA): $R_f \approx 0.52$. **^{1}H NMR** (500 MHz, methanol-d_4): δ 7.35 (d, *J* = 4.4 Hz, 4H), 7.34 – 7.28 (m, 1H), 5.17 and 5.13 (ABq, *J* = 12.3 Hz, 2H), 4.44 (q, *J* = 7.3 Hz, 1H), 4.38 (q, *J* = 7.1 Hz, 1H), 4.02 – 3.93 (m, 1H), 3.90 and 3.82 (ABq, *J* = 16.7 Hz, 2H), 2.40 (dd, *J* = 13.9, 4.1 Hz, 1H), 2.31 (dd, *J* = 13.9, 8.7 Hz, 1H), 1.55 – 1.43 (m, 3H), 1.40 (d, *J* = 7.3 Hz, 3H), 1.38 – 1.22 (m, 12H), 0.90 (app t, *J* = 7.0 Hz, 3H). **^{13}C{^{1}H} NMR** (126 MHz, methanol-d_4): δ 175.1, 174.8, 173.8, 171.4, 137.3, 129.6 (2 × C), 129.3, 129.2 (2 × C), 70.0, 67.9, 50.1, 49.6, 44.7, 43.6, 38.4, 33.0, 30.7, 30.4, 26.6, 23.7, 18.1, 17.2, 14.4. **FTIR** (neat) cm^{-1}: 3285, 2921, 2851, 1740, 1642, 1619, 1549, 1454, 1404, 1323, 1218, 1166, 1068, 695. **HRMS** (APCI+) m/z: $[M + H]^+$ Calcd for $C_{25}H_{40}N_3O_6$ 478.2912; Found 478.2915.

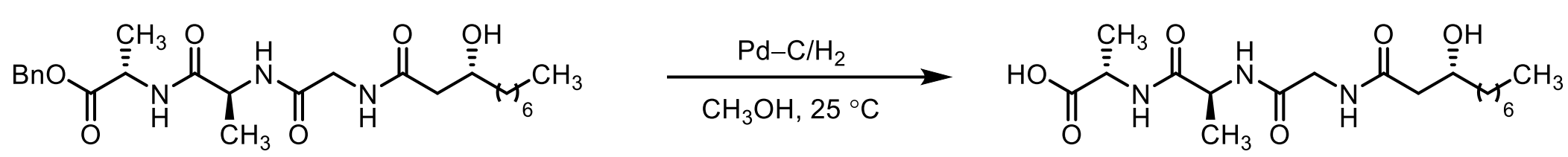

Palladium on charcoal (10% Pd basis; 20 mg) was added to a stirred solution of benzyl ester **25** (200 mg, 0.42 mmol, 1 equiv) in methanol (12 mL) at 25 °C. After stirring the resulting suspension for 10 min at 25 °C, the flask was evacuated, and a double-layered balloon filled with hydrogen gas was attached. The mixture was stirred vigorously for 1 h at 25 °C. The reaction mixture was then filtered through a Celite pad, and the pad was washed with methanol (3 × 20 mL). The filtrate was concentrated in vacuo to afford the acybolin B side chain **16** as a white amorphous solid (168 mg, 96%).

<u>Acybolin B side chain **16**:</u> $[\alpha]_{589}^{23} = -32.3$ (*c* 1.0, CH_3OH). **^{1}H NMR** (500 MHz, methanol-d_4): δ 4.40 (q, *J* = 7.2 Hz, 1H), 4.35 (q, *J* = 7.4 Hz, 1H), 4.02 – 3.95 (m, 1H), 3.92 and 3.84 (ABq, *J* = 16.7 Hz, 2H), 2.41 (dd, *J* = 13.9, 4.1 Hz, 1H), 2.32 (dd, *J* = 13.9, 8.7 Hz, 1H), 1.55 – 1.43 (m, 2H), 1.40 (d, *J* = 7.3 Hz, 3H), 1.36 (d, *J* = 7.2 Hz, 3H), 1.34 – 1.24 (m, 10H), 0.90 (app t, *J* = 7.1 Hz, 3H). **^{13}C{^{1}H} NMR** (126 MHz, methanol-d_4): δ 176.0, 175.0, 174.5, 171.4, 70.0, 50.2, 49.5, 44.7, 43.6, 38.4, 33.0, 30.7, 30.4, 26.6, 23.7, 18.1, 17.7, 14.4. **FTIR** (neat) cm^{-1}: 3410, 3279, 3090, 2957,

2921, 2852, 2072, 1734, 1641, 1620, 1551, 1455, 1407, 1224, 695. **HRMS** (APCI+) *m/z*: [M + H]$^+$ Calcd for $C_{18}H_{34}N_3O_6$ 388.2442; Found 388.2444.

Assembly of acybolin B (5)

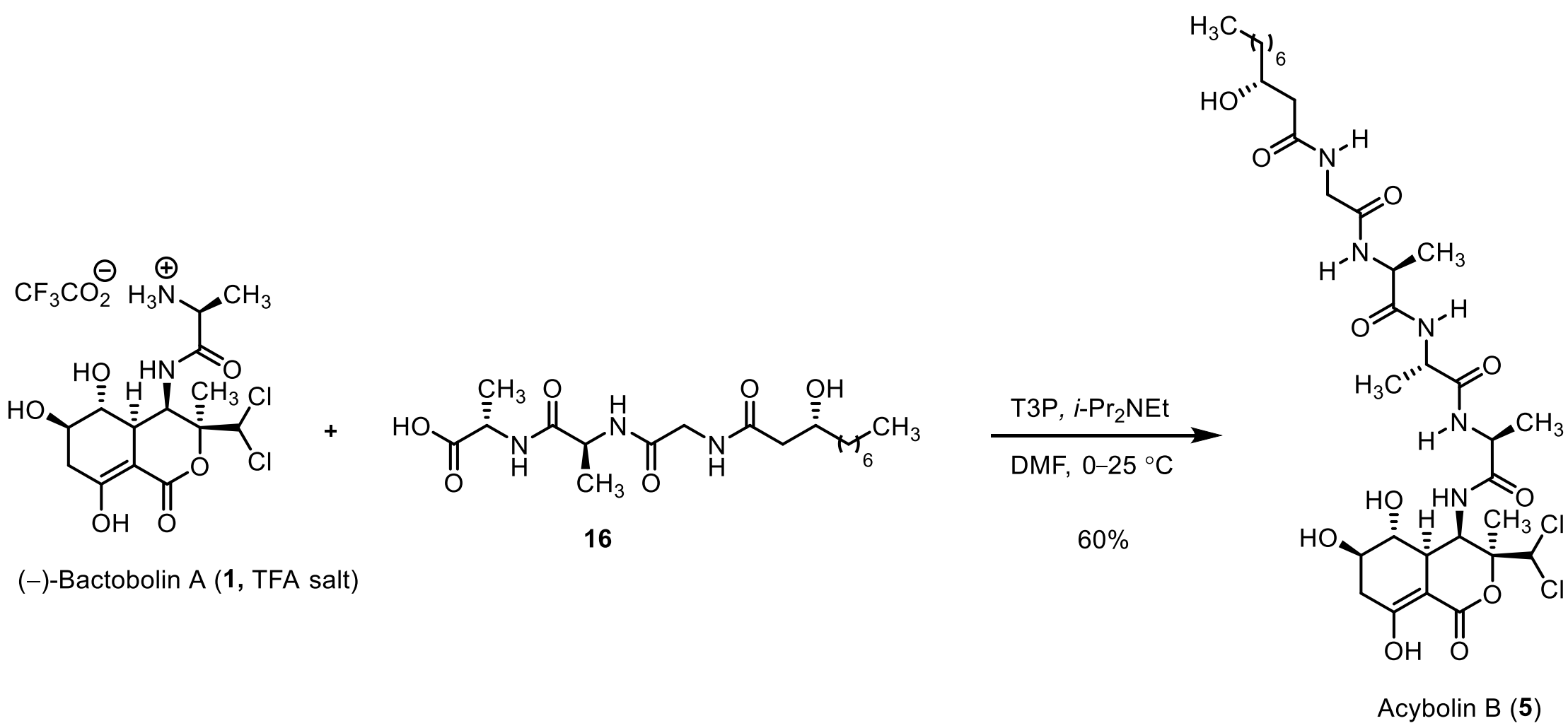

The general procedure described on page 9 was followed. This specific experiment used (–)-bactobolin A (**1**, TFA salt) (20 mg, 0.04 mmol, 1 equiv), acybolin B side chain **16** (31 mg, 0.08 mmol, 2.0 equiv), T3P reagent (24 µL, 0.08 mmol, 2.0 equiv), and *N,N*-diisopropylethylamine (28 µL, 0.16 mmol, 4.0 equiv) in *N,N*-dimethylformamide (2.0 mL). The quench was performed with 10% aqueous solution of lithium chloride (15 mL) and extraction with ethyl acetate (3 × 15 mL). Acybolin B (**5**) was obtained as a white amorphous solid (18 mg, 60%) after purification.

<u>Acybolin B (**5**)</u>: $[\alpha]_{589}^{23}$ = –24.0 (*c* 0.23, CH_3OH). **TLC** (10% methanol in dichloromethane; UV, $KMnO_4$): $R_f \approx 0.52$. **^{1}H NMR** (500 MHz, methanol-d_4): δ 6.31 (s, 1H), 4.85 (d, *J* = 3.8 Hz, 1H), 4.37 (q, *J* = 7.3 Hz, 1H), 4.18 (q, *J* = 7.2 Hz, 1H), 4.06 (q, *J* = 7.3 Hz, 1H), 4.01 – 3.96 (m, 1H), 3.94 (d, *J* = 16.5 Hz, 1H), 3.84 (dd, *J* = 9.9, 6.6 Hz, 1H), 3.80 (d, *J* = 16.4 Hz, 1H), 3.21 (app t, *J* = 9.3 Hz, 1H), 2.92 (app dt, *J* = 9.3, 2.7 Hz, 1H), 2.78 (dd, *J* = 18.6, 6.7 Hz, 1H), 2.49 – 2.39 (m, 2H), 2.32 (dd, *J* = 14.2, 8.1 Hz, 1H), 1.67 (s, 3H), 1.53 – 1.48 (m, 2H), 1.43 (d, *J* = 7.3 Hz, 3H), 1.39 (d, *J* = 7.3 Hz, 3H), 1.37 (d, *J* = 7.3 Hz, 3H), 1.35 – 1.28 (m, 10H), 0.90 (app t, *J* = 7.1 Hz, 3H). **^{13}C{^{1}H} NMR** (126 MHz, methanol-d_4): δ 176.4, 176.2, 176.1, 175.3, 175.2, 173.0, 170.3, 90.9, 86.9, 76.2, 72.4, 70.0, 69.9, 51.8 (2 × C), 50.7, 48.0, 44.5, 44.0, 40.4, 38.3, 38.0, 33.0, 30.7, 30.4, 26.6, 23.7, 19.3, 18.0, 17.3, 17.0, 14.4. **FTIR** (neat) cm^{-1}: 3297, 2927, 2856, 1651, 1536,

1232, 1125, 1067, 795. **HRMS** (APCI+) *m/z*: $[M + H]^+$ Calcd for $C_{32}H_{52}Cl_2N_5O_{11}$ 752.3035; Found 752.3030.

Acybolin H (6)

Synthesis of the side chain of acybolin H (15)

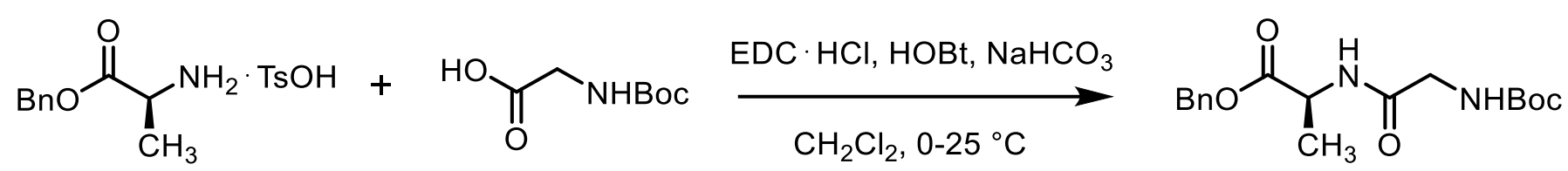

20 26 75% 27

p-Toluenesulfonate salt of L-alanine benzyl ester (**20**) (2.00 g, 5.69 mmol, 1 equiv) was added to a stirred solution of *N*-Boc glycine (**26**) (1.10 g, 6.26 mmol, 1.1 equiv), sodium bicarbonate (1.43 g, 17.07 mmol, 3.0 equiv), HOBt (2.30 g, 17.07 mmol, 3.0 equiv), and EDC·HCl (3.27 g, 17.07 mmol, 3.0 equiv) in dichloromethane (40 mL) at 0 °C. The resulting reaction mixture was stirred at 0 °C for 2 h. After this time, the reaction mixture was allowed to reach room temperature and stirred for 2 h. The reaction mixture was quenched by adding water (40 mL). The aqueous phase was extracted with dichloromethane (60 mL), and the combined organic phases were washed with saturated aqueous sodium bicarbonate solution (20 mL) and brine (40 mL). The organic phases were dried over anhydrous sodium sulfate, the dried solution was filtered, and the filtrate was concentrated in vacuo. The obtained residue was subjected to flash column chromatography purification (gradient elution with 10% methanol in dichloromethane) to afford the protected dipeptide **27** as a pale-yellow sticky solid (1.30 g, 75%).

27: **TLC** (40% ethyl acetate in cyclohexane; PMA): $R_f \approx 0.3$. **^{1}H NMR** (500 MHz, chloroform-*d*): δ 7.42 – 7.29 (m, 5H), 6.67 (d, *J* = 7.5 Hz, 1H), 5.19 and 5.16 (ABq, *J* = 12.3 Hz, 3H, CH_2 and NH), 4.64 (app quint, *J* = 7.2 Hz, 1H), 3.89 – 3.72 (m, 2H), 1.45 (s, 9H), 1.42 (d, *J* = 7.2 Hz, 3H). **^{13}C{^{1}H} NMR** (126 MHz, chloroform-*d*): δ 172.7, 169.1, 156.1, 135.4, 128.8 (2 × C), 128.6, 128.3 (2 × C), 80.5, 67.3, 48.3, 44.4, 28.4 (3 × C), 18.5. **FTIR** (neat) cm^{-1}: 3309, 3066, 2978, 2935, 1738, 1714, 1664, 1511, 1274, 1196, 1050, 736. **HRMS** (APCI–) *m/z*: $[M + Cl]^-$ Calcd for $C_{17}H_{24}N_2O_5Cl$ 371.1379; Found 371.1383.

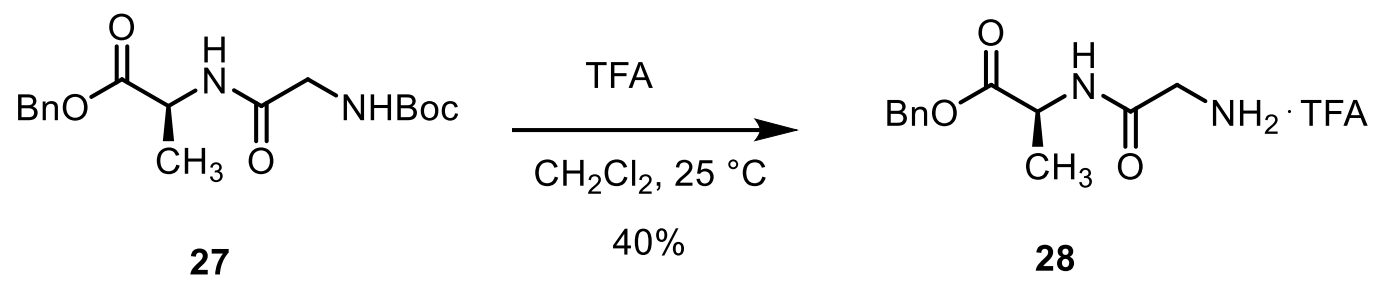

A solution of trifluoroacetic acid in dichloromethane (20% by volume, 25 mL) was added to a stirred solution of protected dipeptide **27** (1.2 g, 3.56 mmol, 1 equiv) in dichloromethane (25 mL) at 25 °C. The reaction mixture was allowed to stir for 16 h at room temperature. Then, the reaction mixture was concentrated in vacuo under reduced pressure. Sequential concentration of the resulting residue, first from diethyl ether and then from dichloromethane, yielded a residue, which was subjected to flash column chromatography purification (elution with 10% methanol in dichloromethane) to afford the trifluoroacetate salt **28** as a colorless sticky solid (500 mg, 40%).

<u>**28**:</u> **TLC** (10% methanol in dichloromethane; PMA) $R_f \approx 0.33$. **^{1}H NMR** (500 MHz, methanol-*d*$_4$): δ 7.37 – 7.35 (m, 4H), 7.34 – 7.29 (m, 1H), 5.17 (app s, 2H), 4.53 (q, *J* = 7.3 Hz, 1H), 3.72 and 3.69 (ABq, *J* = 16.1 Hz , 2H), 1.41 (d, *J* = 7.3 Hz, 3H). **^{13}C{^{1}H} NMR** (126 MHz, methanol-*d*$_4$): δ 173.7, 167.1, 137.2, 129.6 (2 × C), 129.4, 129.2 (2 × C), 68.1, 49.7, 41.4, 17.4. **FTIR** (neat) cm^{-1}: 3069, 2987, 1735, 1668, 1558, 1499, 1455, 1433, 1375, 1248, 1200, 1180, 1131, 1045, 912, 837, 799, 736, 722. **HRMS** (APCI+) *m/z*: [M – TFA + H]$^+$ Calcd for $C_{12}H_{17}N_2O_3$ 237.1234; Found 237.1234.

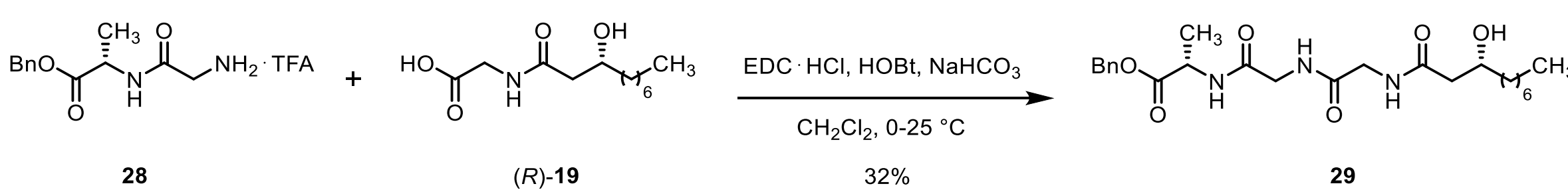

Trifluoroacetate salt **28** (200 mg, 0.57 mmol, 1 equiv) was added to a stirred solution of hydroxy acid (*R*)-**19** (154 mg, 0.63 mmol, 1.1 equiv), sodium bicarbonate (143 mg, 1.71 mmol, 3.0 equiv), HOBt (231 mg, 1.71 mmol, 3.0 equiv), and EDC·HCl (328 mg, 1.71 mmol, 3.0 equiv) in dichloromethane (10 mL) at 0 °C. The resulting reaction mixture was stirred at 0 °C for 2 h. After this time, the reaction mixture was allowed to reach room temperature and stirred for 2 h. The reaction mixture was quenched by adding water (15 mL). The aqueous phase was extracted with dichloromethane (30 mL), and the combined organic phases were washed with saturated aqueous sodium bicarbonate solution (20 mL) and brine (20 mL). The organic phases were dried over anhydrous sodium sulfate, the dried solution was filtered, and the filtrate was concentrated in

vacuo. The obtained residue was subjected to flash column chromatography purification (gradient elution with 10% methanol in dichloromethane) to afford benzyl ester **29** as a white amorphous solid (85 mg, 32%).

<u>**29**:</u> $[\alpha]^{23}_{589}$= –28.5 (*c* 1.0, CH_3OH). **TLC** (10% methanol in dichloromethane; PMA): $R_f \approx 0.6$. **^{1}H NMR** (500 MHz, chloroform-*d*): δ 7.41 – 7.30 (m, 5H), 7.25 – 7.23 (m, 1H), 6.77 (d, *J* = 7.2 Hz, 1H), 6.45 (app t, *J* = 6.0 Hz, 1H), 5.20 and 5.15 (ABq, *J* = 12.3 Hz, 2H), 4.58 (app quint, *J* = 7.3 Hz, 1H), 4.34 (dd, *J* = 17.2, 7.8 Hz, 1H), 4.19 (dd, *J* = 17.0, 6.1 Hz, 1H), 4.14 – 4.02 (m, 1H), 3.75 – 3.64 (m, 2H), 2.47 (dd, *J* = 13.8, 3.1 Hz, 1H), 2.25 (dd, *J* = 13.8, 10.3 Hz, 1H), 1.51 (m, 1H), 1.48 (d, *J* = 7.3 Hz, 3H), 1.46 – 1.20 (m, 11H), 0.87 (app t, *J* = 7.1 Hz, 3H). **^{13}C{^{1}H} NMR** (126 MHz, chloroform-*d*): δ 174.0, 173.8, 169.0, 168.9, 135.3, 128.8 (2 × C), 128.7, 128.2 (2 × C), 69.6, 67.5, 48.5, 43.9, 43.7, 42.6, 37.8, 32.0, 29.6, 29.4, 25.6, 22.8, 17.5, 14.2. **FTIR** (neat) cm^{-1}: 3300, 3089, 2922, 2853, 1742, 1648, 1543, 1454, 1418, 1200, 1153, 1080, 958, 902, 873, 729, 694. **HRMS** (APCI+) *m/z*: $[M + H]^+$ Calcd for $C_{24}H_{38}N_3O_6$ 464.2755; Found 464.2757.

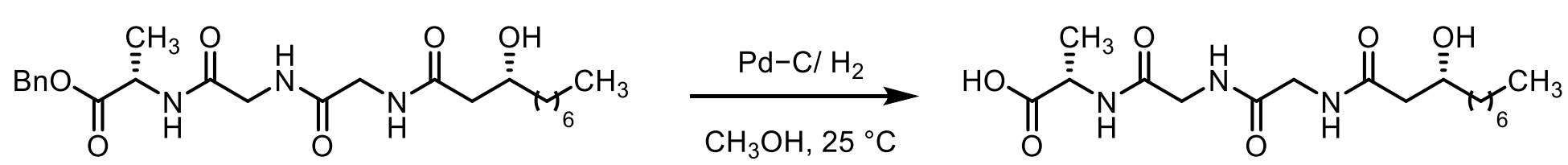

29 **15**

Palladium on charcoal (10% Pd basis; 8.5 mg) was added to a stirred solution of benzyl ester **29** (85 mg, 0.23 mmol, 1 equiv) in methanol (7 mL) at 25 °C. After stirring the resulting suspension for 10 min at 25 °C, the flask was evacuated, and a double-layered balloon filled with hydrogen gas was attached. The mixture was stirred vigorously for 1 h at 25 °C. The reaction mixture was then filtered through Celite. The Celite was washed with methanol (3 × 15 mL). The filtrate was concentrated in vacuo to afford the acybolin H side chain **15** as a white amorphous solid (40 mg, 58%).

<u>Acybolin H side chain **15**:</u> $[\alpha]^{23}_{589}$ = –11.4 (*c* 1.0, CH_3OH). **^{1}H NMR** (500 MHz, methanol-d_4): δ 4.41 (q, *J* = 7.3 Hz, 1H), 4.04 – 3.97 (m, 1H), 3.93 / 3.84 and 3.93 / 3.88 (two ABq, *J* = 16.8 Hz, 4H), 2.42 (dd, *J* = 14.0, 4.2 Hz, 1H), 2.34 (dd, *J* = 14.0, 8.8 Hz, 1H), 1.54 – 1.45 (m, 2H), 1.42 (d, *J* = 7.3 Hz, 3H), 1.39 – 1.25 (m, 10H), 0.91 (app t, *J* = 7.1 Hz, 3H). **^{13}C{^{1}H} NMR** (126 MHz, methanol-d_4): δ 176.1, 175.4, 172.3, 171.2, 70.0, 49.5, 49.3, 44.6, 43.9, 43.2, 38.4, 33.0, 30.6, 30.4, 26.6, 23.7, 17.6, 14.4. **FTIR** (neat) cm^{-1}: 3496, 3313, 3068, 2919, 2849, 1720, 1639, 1456, 1376,

1270, 1173, 1088, 1019, 953, 888, 765, 683. **HRMS** (APCI+) *m/z*: $[M + H]^+$ Calcd for $C_{17}H_{32}N_3O_6$ 374.2286; Found 374.2285.

Assembly of acybolin H (6)

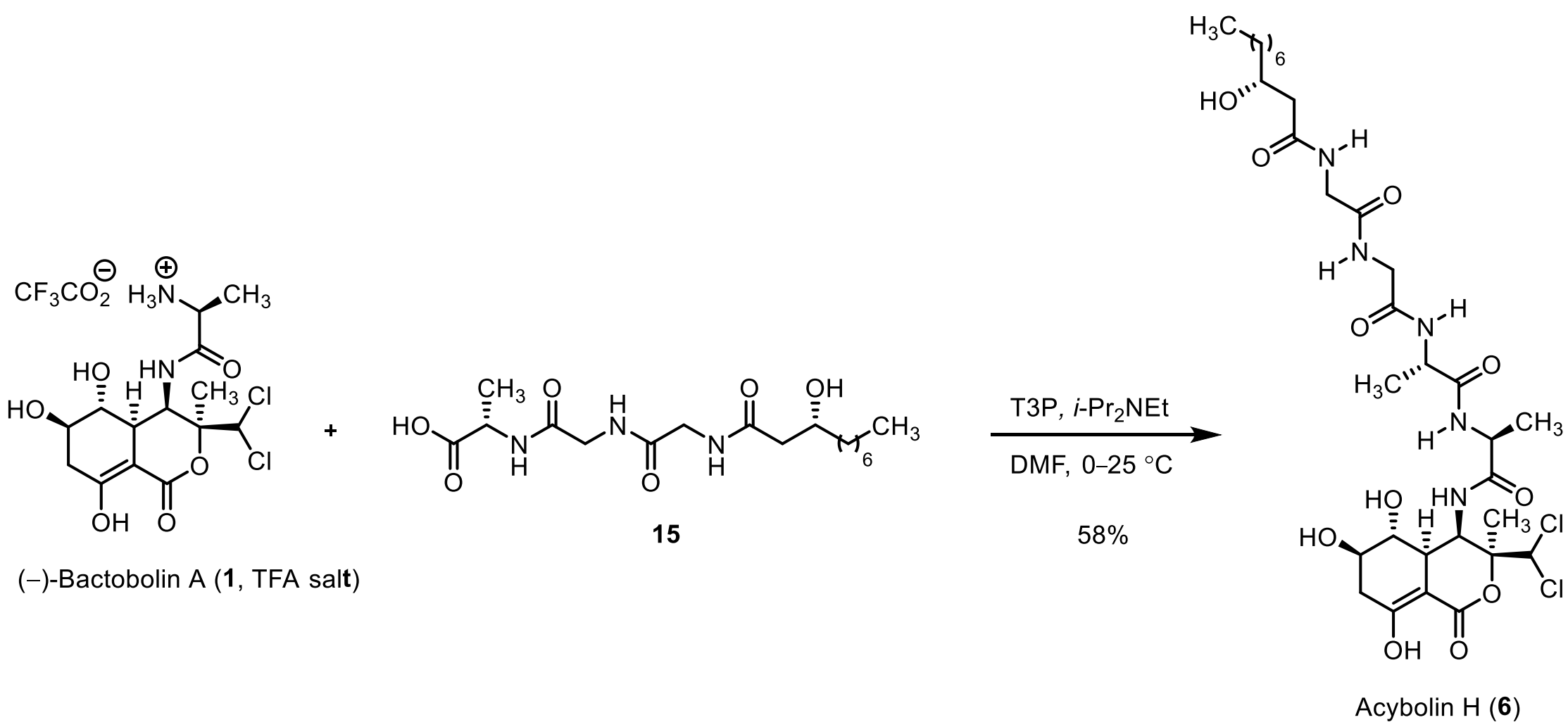

The general procedure described on page 9 was followed. The specific experiment used bactobolin A (**1**, TFA salt) (20 mg, 0.04 mmol, 1 equiv), acybolin H side chain **15** (30 mg, 0.08 mmol, 2.0 equiv), T3P reagent (24 µL, 0.08 mmol, 2.0 equiv), and *N,N*-diisopropylethylamine (28 µL, 0.16 mmol, 4.0 equiv) in *N,N*-dimethylformamide (2.0 mL). The quench and extraction were performed with 10% aqueous solution of lithium chloride (15 mL), and ethyl acetate (3 × 15 mL). Acybolin H (**6**) was obtained as a white amorphous solid (17 mg, 58%) after purification.

Acybolin H (**6**): $[\alpha]_{589}^{23}$ = –26.4 (*c* 0.4, CH_3OH). **TLC** (10% methanol in dichloromethane; UV, $KMnO_4$): $R_f \approx 0.45$. **^{1}H NMR** (500 MHz, methanol-d_4): δ 6.18 (s, 1H), 4.87 (d, *J* = 3.6 Hz, 1H), 4.34 (q, *J* = 7.2 Hz, 1H), 4.13 (q, *J* = 7.2 Hz, 1H), 4.03 – 3.96 (m, 1H), 3.94 (app s, 1H), 3.92 (app s, 1H), 3.91 – 3.86 (m, 1H), 3.86 – 3.79 (m, 2H), 3.22 (app t, *J* = 9.4 Hz, 1H), 2.91 (app dt, *J* = 9.3, 3.2 Hz, 1H), 2.79 (dd, *J* = 18.6, 6.9 Hz, 1H), 2.47 – 2.31 (m, 3H), 1.68 (s, 3H), 1.53 – 1.47 (m, 2H), 1.42 (d, *J* = 7.5 Hz, 3H), 1.38 (d, *J* = 7.3 Hz, 3H), 1.36 – 1.26 (m, 10H), 0.90 (app t, *J* = 7.0 Hz, 3H). **^{13}C{^{1}H} NMR** (126 MHz, methanol-d_4): δ 176.6, 176.1, 175.2, 175.2, 172.9, 171.9, 170.3, 86.9, 79.5, 76.2, 72.3, 70.0, 69.9, 51.4, 50.9, 47.9, 44.6, 44.0, 43.7, 40.5, 38.4, 38.0, 33.0, 30.7, 30.4, 26.6, 23.7, 19.3, 17.6, 17.4, 14.4. **FTIR** (neat) cm^{-1}: 3295, 2927, 2856, 1651, 1536, 1233, 1125, 1069, 795. **HRMS** (APCI+) *m/z*: $[M + H]^+$ Calcd for $C_{31}H_{50}Cl_2N_5O_{11}$ 738.2878; Found 738.2875.

Acybolin I (7)

Synthesis of the side chain of acybolin I (17)

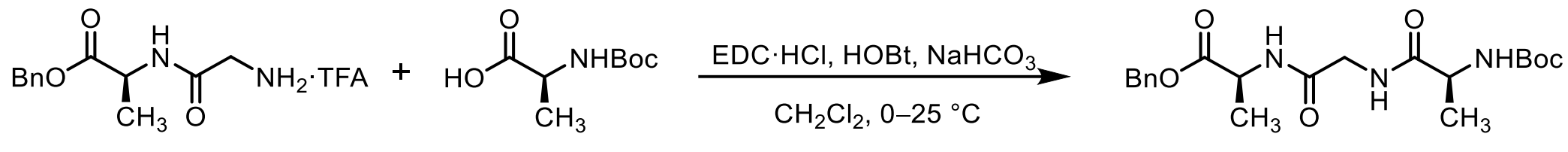

Trifluoroacetate salt **28** (300 mg, 0.86 mmol, 1 equiv) was added to a stirred *N*-Boc L-alanine (**22**) (178 mg, 0.94 mmol, 1.1 equiv), sodium bicarbonate (216 mg, 2.57 mmol, 3.0 equiv), HOBt (347 mg, 2.57 mmol, 3.0 equiv), and EDC·HCl (493 mg, 2.57 mmol, 3.0 equiv) in dichloromethane (10 mL) at 0 °C. The resulting reaction mixture was stirred at 0 °C for 2 h. After this time, the reaction mixture was allowed to reach room temperature and stirred for 2 h. The reaction mixture was quenched by adding water (10 mL). The aqueous phase was extracted with dichloromethane (40 mL), and the combined organic phases were washed with a saturated aqueous sodium bicarbonate solution (20 mL) and brine (20 mL). The organic phases were dried over anhydrous sodium sulfate, the dried solution was filtered, and the filtrate was concentrated in vacuo. The obtained residue was subjected to flash column chromatography purification (gradient elution with 10% methanol in dichloromethane) to afford protected tripeptide **30** as a white amorphous solid (262 mg, 71%).

30: **TLC** (100% ethyl acetate; $KMnO_4$): $R_f \approx 0.35$. **^{1}H NMR** (500 MHz, methanol-*d*$_4$): δ 7.37 – 7.35 (m, 4H), 7.34 – 7.29 (m, 1H), 5.18 and 5.15 (ABq, J = 12.4 Hz, 2H), 4.48 (qd, J = 7.3, 2.2 Hz, 1H), 4.02 (q, J = 7.1 Hz, 1H), 3.94 – 3.81 (m, 2H), 1.43 (s, 9H), 1.41 (d, J = 7.3 Hz, 3H), 1.32 (d, J = 7.1 Hz, 3H). **^{13}C{^{1}H} NMR** (126 MHz, methanol-*d*$_4$): δ 176.4, 173.8, 171.4, 157.9, 137.3, 129.6 (2 × C), 129.3, 129.1 (2 × C), 80.7, 67.9, 51.9, 49.7, 43.3, 28.7 (3 × C), 17.8, 17.4. **FTIR** (neat) cm^{-1}: 3329, 2976, 2927, 1744, 1683, 1641, 1514, 1248, 1159, 748. **HRMS** (APCI+) *m/z*: $[M + H]^+$ Calcd for $C_{20}H_{30}N_3O_6$ 408.2129; Found 408.2132.

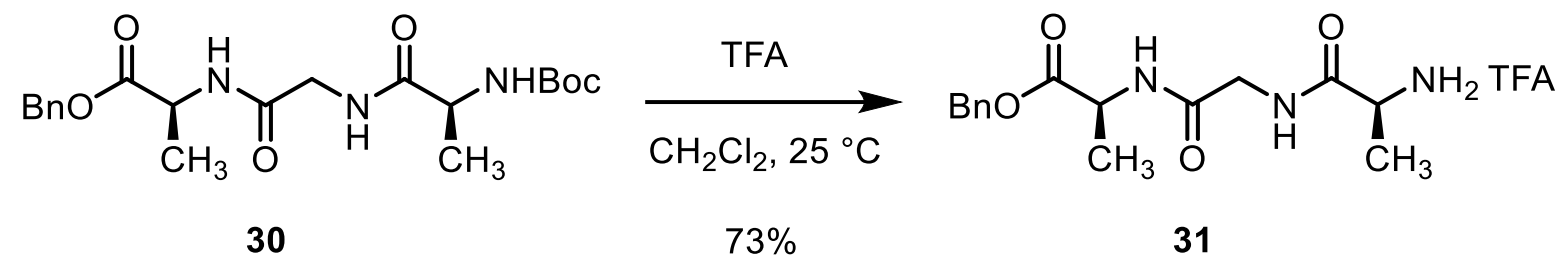

A solution of trifluoroacetic acid in dichloromethane (20% by volume, 8 mL) was added to a stirred solution of protected tripeptide **30** (237 mg, 0.58 mmol, 1 equiv) in dichloromethane (8 mL) at 25 °C. The reaction mixture was allowed to stir for 16 h at 25 °C. Then, the reaction mixture was concentrated in vacuo under reduced pressure. The residue was successively concentrated from diethyl ether and dichloromethane, and the obtained residue was subjected to flash column chromatography purification (elution with 10% methanol in dichloromethane) to obtain the trifluoroacetate salt **31** as an off-white amorphous solid (180 mg, 73%).

<u>31</u>: **TLC** (10% methanol in dichloromethane; $KMnO_4$) $R_f \approx 0.3$. **^{1}H NMR** (500 MHz, methanol-d_4): δ 7.37 – 7.34 (m, 4H), 7.34 – 7.30 (m, 1H), 5.16 (app s, 2H), 4.47 (qd, J = 7.3, 2.1 Hz, 1H), 4.00 – 3.91 (m, 3H), 1.52 (d, J = 7.0 Hz, 3H), 1.40 (d, J = 7.3 Hz, 3H). **^{13}C{^{1}H} NMR** (126 MHz, methanol-d_4): δ 173.9, 171.5, 170.8, 137.3, 129.6 (2 × C), 129.3, 129.1 (2 × C), 68.0, 50.3, 49.7, 42.9, 42.8, 17.4. **FTIR** (neat) cm^{-1}: 3280, 3081, 2919, 1738, 1673, 1649, 1535, 1247, 1201, 840, 799. **HRMS** (APCI+) m/z: $[M - TFA + H]^+$ Calcd for $C_{15}H_{22}N_3O_4$ 308.1605; Found 308.1604.

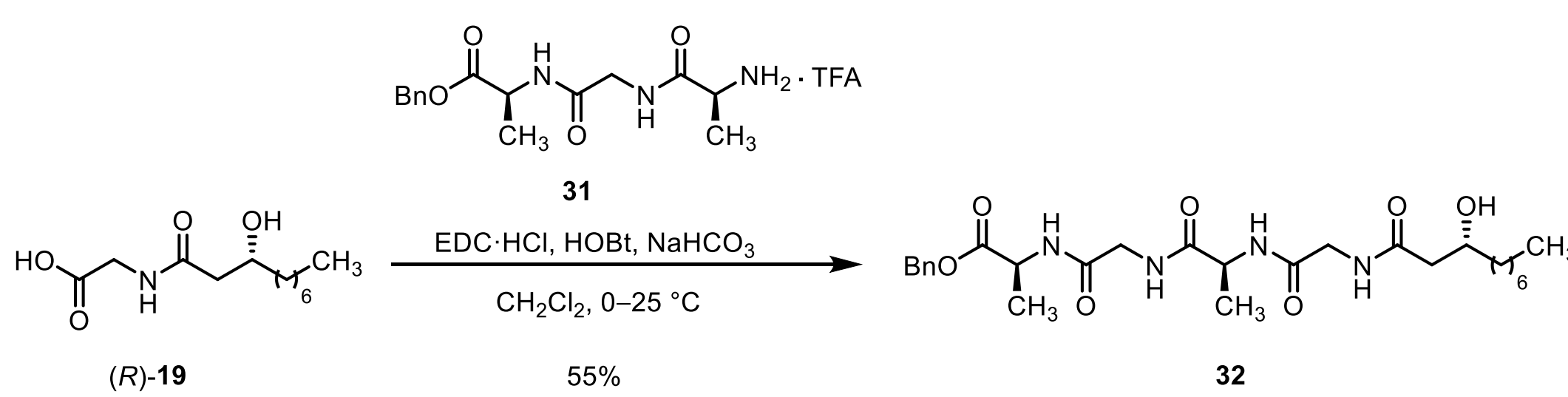

The trifluoroacetate salt **31** (100 mg, 0.24 mmol, 1 equiv) was added to a stirred solution of hydroxy acid (R)-**19** (64 mg, 0.26 mmol, 1.1 equiv), $NaHCO_3$ (60 mg, 0.75 mmol, 3.0 equiv), HOBt (96 mg, 0.71 mmol, 3.0 equiv), EDC·HCl (136 mg, 0.71 mmol, 3.0 equiv) in dichloromethane (5 mL) at 0 °C. The resulting reaction mixture was stirred at 0 °C for 2 h. After this time, the reaction mixture was allowed to reach room temperature and stirred for 2 h. The reaction mixture was quenched by adding water (10 mL). The aqueous phase was extracted with dichloromethane (30 mL), and the combined organic phases were washed with a saturated aqueous sodium bicarbonate solution (20 mL) and brine (20 mL). The organic phases were dried over

anhydrous sodium sulfate, the dried solution was filtered, and the filtrate was concentrated in vacuo to provide the crude residue containing benzyl ester **32.** The obtained residue was subjected to flash column chromatography purification (gradient elution with 10% methanol in dichloromethane) to afford benzyl ester **32** as a white amorphous solid (70 mg, 55%).

<u>**32**:</u> $[\alpha]_{589}^{23}$ = –24.1 (*c* 1.0, CH_3OH). **TLC** (10% methanol in dichloromethane; $KMnO_4$) $R_f \approx 0.53$. **^{1}H NMR** (500 MHz, methanol-d_4): δ 7.38 – 7.34 (m, 4H), 7.34 – 7.28 (m, 1H), 5.18 and 5.15 (AB q, *J* = 12.4 Hz, 2H), 4.47 (q, *J* = 7.3 Hz, 1H), 4.30 (q, *J* = 7.2 Hz, 1H), 4.00 – 3.95 (m, 1H), 3.94 (d, *J* = 1.7 Hz, 1H), 3.91 (d, *J* = 1.5 Hz, 1H), 3.84 (d, *J* = 5.5 Hz, 1H), 3.81 (d, *J* = 5.4 Hz, 1H), 2.41 (dd, *J* = 14.0, 4.1 Hz, 1H), 2.31 (dd, *J* = 14.0, 8.8 Hz, 1H), 1.54 – 1.44 (m, 2H), 1.41 (d, *J* = 7.3 Hz, 3H), 1.37 (d, *J* = 7.2 Hz, 3H), 1.35 – 1.25 (m, 10H), 0.90 (app t, *J* = 7.1 Hz, 3H). **^{13}C{^{1}H} NMR** (126 MHz, methanol-d_4): δ 175.4, 175.2, 173.8, 171.9, 171.4, 137.3, 129.6 (2 × C), 129.3, 129.1 (2 × C), 70.0, 68.0, 50.9, 49.7, 44.7, 43.7, 43.3, 38.4, 33.0, 30.6, 30.4, 26.6, 23.7, 17.4, 17.4, 14.4. **FTIR** (neat) cm^{-1}: 3290, 2953, 2924, 2853, 1733, 1626, 1540, 1271, 1155, 734. **HRMS** (APCI+) *m/z*: $[M + H]^+$ Calcd for $C_{27}H_{43}N_4O_7$ 535.3126; Found 535.3123.

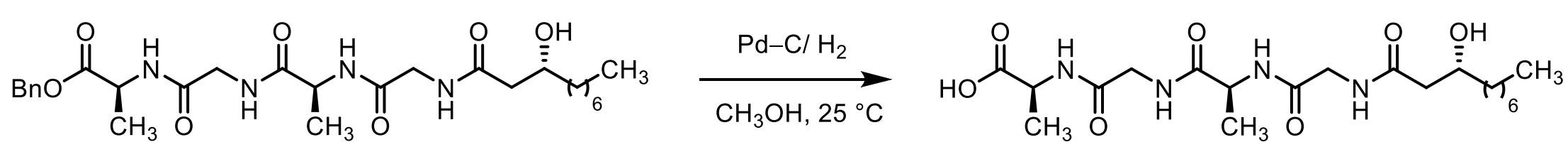

32 **17**

Palladium on charcoal (10% Pd basis; 6.9 mg) was added to a stirred solution of benzyl ester **32** (69 mg, 0.13 mmol, 1 equiv), in methanol (5 mL) at 25 °C. After stirring the resulting suspension for 10 min at 25 °C, the flask was evacuated, and a double-layered balloon filled with hydrogen gas was attached. The mixture was stirred vigorously for 1 h at 25 °C and then filtered through Celite. The Celite was washed with methanol (3 × 15 mL). The filtrate was concentrated in vacuo to afford acybolin I side chain **17** as a white amorphous solid (45 mg, 77%).

<u>Acybolin I side chain **17**:</u> $[\alpha]_{589}^{23}$ = –20.9 (*c* 1.0, CH_3OH). **^{1}H NMR** (500 MHz, methanol-d_4): δ 4.39 (q, *J* = 7.3 Hz, 1H), 4.33 (q, *J* = 7.1 Hz, 1H), 4.00 – 3.96 (m, 1H), 3.94 / 3.83 and 3.93 / 3.83 (two ABq, *J* = 16.8 Hz, 4 H), 2.42 (dd, *J* = 14.0, 4.2 Hz, 1H), 2.32 (dd, *J* = 14.0, 8.8 Hz, 1H), 1.54 – 1.46 (m, 2H), 1.41 (d, *J* = 7.3 Hz, 3H), 1.38 (d, *J* = 7.2 Hz, 3H), 1.36 – 1.25 (m, 10H), 0.90 (app t, *J* = 7.1 Hz, 3H). **^{13}C{^{1}H} NMR** (126 MHz, methanol-d_4): δ 175.9, 175.4, 175.2, 171.8, 171.6, 70.0, 50.8, 49.5, 44.7, 43.7, 43.3, 38.4, 33.0, 30.7, 30.4, 26.6, 23.7, 17.7, 17.5, 14.4. **FTIR** (neat)

cm^{-1}: 3735, 3300, 3079, 2926, 2855, 2325, 2164, 1721, 1688, 1627, 1450,1368. **HRMS** (APCI+) *m/z*: $[M + H]^+$ Calcd for $C_{20}H_{37}N_4O_7$ 445.2657; Found 445.2653.

Assembly of acybolin I (7)

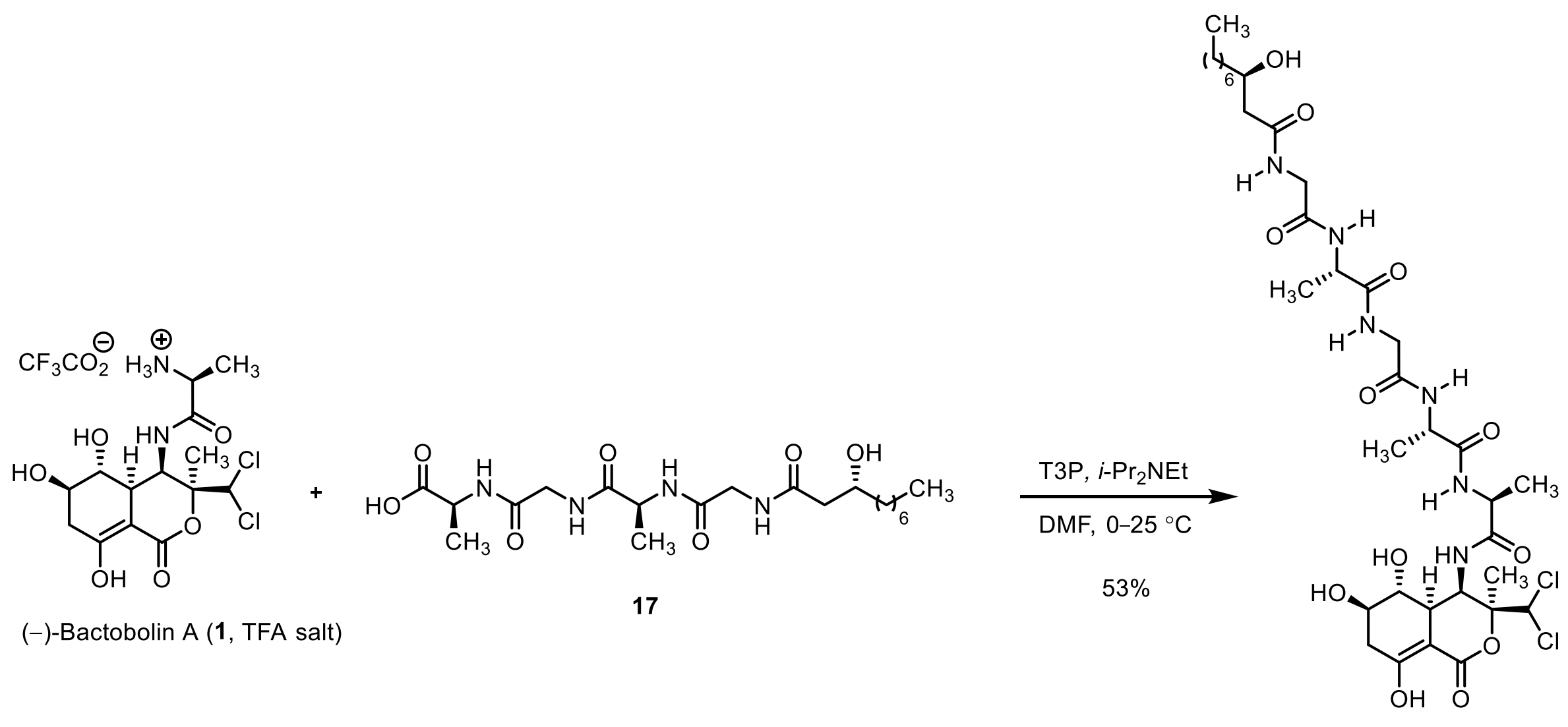

(–)-Bactobolin A (**1**, TFA salt)

17

Acybolin I (**7**)

The general procedure described on page 9 was followed. The specific experiment used (–)-bactobolin A (**1**, TFA salt) (20 mg, 0.04 mmol, 1 equiv), acybolin I side chain **17** (36 mg, 0.08 mmol, 2.0 equiv), T3P reagent (24 µL, 0.08 mmol, 2.0 equiv), *N,N*-diisopropylethylamine (28 µL, 0.16 mmol, 4.0 equiv) in dimethylformamide (2.0 mL). The quench was performed with a 10% aqueous solution of lithium chloride (15 mL) and extraction with ethyl acetate (3 × 15 mL). Acybolin I (**7**) was obtained as a white amorphous solid (17 mg, 53%) after purification.

Acybolin I (7): $[\alpha]_{589}^{23}$ = –33.8 (*c* 0.22, CH_3OH). **TLC** (10% methanol in dichloromethane; UV, $KMnO_4$): $R_f \approx 0.52$. **^{1}H NMR** (500 MHz, methanol-d_4): δ 6.31 (s, 1H), 4.86 (d, *J* = 3.7 Hz, 1H), 4.37 (q, *J* = 7.2 Hz, 1H), 4.27 (q, *J* = 7.2 Hz, 1H), 4.14 (q, *J* = 7.3 Hz, 1H), 3.99 – 3.94 (m, 2H), 3.94 – 3.87 (m, 1H), 3.87 – 3.83 (m, 2H), 3.83 – 3.80 (m, 2H), 3.21 (app t, *J* = 9.4 Hz, 1H), 2.92 (app dt, *J* = 9.2, 2.6 Hz, 1H), 2.79 (dd, *J* = 18.5, 6.7 Hz, 1H), 2.48 – 2.38 (m, 2H), 2.32 (dd, *J* = 14.0, 8.5 Hz, 1H), 1.68 (s, 3H), 1.53 – 1.47 (m, 2H), 1.43 (d, *J* = 5.3 Hz, 3H), 1.41 (d, *J* = 5.3 Hz, 3H), 1.38 (d, *J* = 7.4 Hz, 3H), 1.35 – 1.27 (m, 10H), 0.90 (app t, *J* = 7.1 Hz, 3H). **^{13}C{^{1}H} NMR** (126 MHz, methanol-d_4): δ 176.7, 176.4, 176.1, 175.2, 175.2, 172.4, 172.1, 170.3, 90.8, 86.9, 76.2, 72.4, 70.0 (2 × C), 51.7, 51.4, 50.9, 47.9, 44.6, 44.1, 43.8, 40.4, 38.4, 38.0, 33.0, 30.7, 30.4, 26.6,

23.7, 19.3, 17.8, 17.4, 17.4, 14.4. **FTIR** (neat) cm^{-1}: 3293, 2928, 2856, 1650, 1537, 1232, 1125, 1069, 795. **HRMS** (APCI+) *m/z*: $[M + H]^+$ Calcd for $C_{34}H_{55}Cl_2N_6O_{12}$ 809.3250; Found 809.3243.

15'-*epi*-Acybolin F (15'-*epi*-4)

Side chain of 15'-*epi*-acybolin F (*epi*-14)

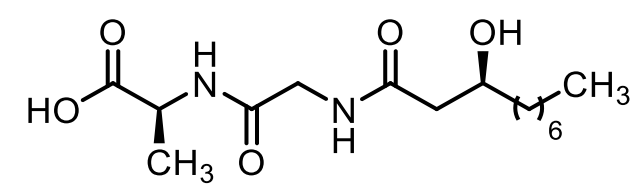

epi-**14**

Side chain *epi*-**14**: $[\alpha]_{589}^{23} = -5.0$ (*c* 0.8, CH_3OH). **^{1}H NMR** (500 MHz, methanol-d_4): δ 4.41 (q, *J* = 7.3 Hz, 1H), 4.01 – 3.95 (m, 1H), 3.93 and 3.87 (ABq, *J* = 16.8 Hz, 2H), 2.42 (dd, *J* = 14.0, 4.3 Hz, 1H), 2.33 (dd, *J* = 14.1, 8.4 Hz, 1H), 1.53 – 1.44 (m, 2H), 1.40 (d, *J* = 7.3 Hz, 3H), 1.38 – 1.24 (m, 10H), 0.90 (app t, *J* = 7.1 Hz, 3H). **^{13}C{^{1}H} NMR** (126 MHz, methanol-d_4): δ 175.8, 174.8, 171.4, 69.9, 44.7, 43.3, 38.3, 33.0, 30.6, 30.4, 26.7, 23.7, 17.7, 14.4. **FTIR** (neat) cm^{-1}: 3418, 3382, 3258, 2956, 2925, 2849, 1724, 1659, 1615, 1536, 1461, 1406, 1334, 1310, 1245, 1192, 1103, 1083, 1017, 981, 871, 722. **HRMS** (APCI–) *m/z*: $[M - H]^-$ Calcd for $C_{15}H_{27}N_2O_5$ 315.1925; Found 315.1926.

Assembly of 15'-*epi*-acybolin F (15'-*epi*-4)

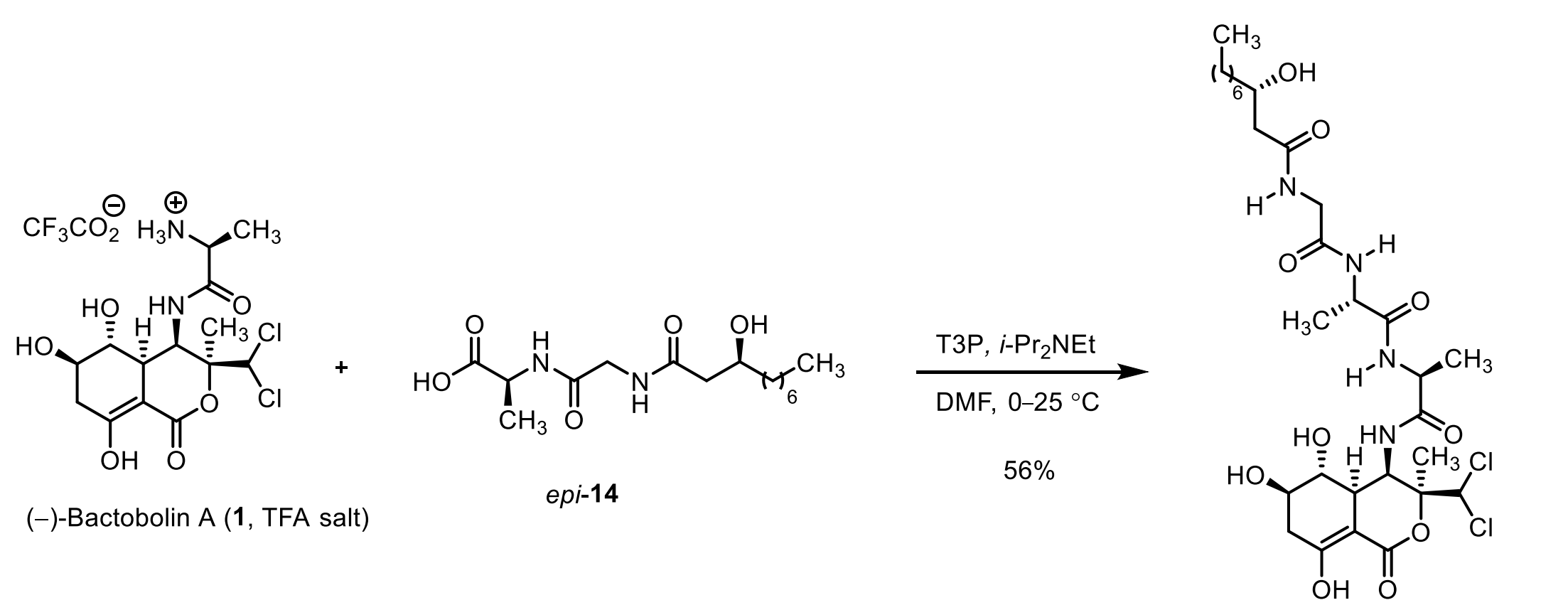

15'-*epi*-Acybolin F (15'-*epi*-**4**)

The general procedure described on page 9 was followed. The specific experiment used bactobolin A (**1**, TFA salt) (30 mg, 0.06 mmol, 1 equiv), side chain (*epi*-**14**) (38 mg, 0.12 mmol, 2.0 equiv), T3P reagent (36 μL, 0.12 mmol, 2.0 equiv), *N,N*-diisopropylethylamine (42 μL, 0.24 mmol, 4.0

equiv) in *N,N*-dimethylformamide (2.0 mL). The quench was performed with 10% aqueous solution of lithium chloride (15 mL) and extraction with ethyl acetate (3 × 15 mL). 15'-*epi*-Acybolin F (15'-*epi*-**4**) was obtained as a white amorphous solid (23 mg, 56%) after purification.

<u>15'-*epi*-Acybolin F (15'-*epi*-**4**):</u> $[\alpha]_{589}^{23}$ = –17.4 (*c* 0.71, CH_3OH). **TLC** (10% methanol in dichloromethane; UV, $KMnO_4$): $R_f \approx 0.63$. **^{1}H NMR** (500 MHz, methanol-d_4): δ 6.23 (s, 1H), 4.86 (d, *J* = 3.6 Hz, 1H), 4.36 (q, *J* = 7.2 Hz, 1H), 4.12 (q, *J* = 7.2 Hz, 1H), 4.05 – 3.96 (m, 1H), 3.89 – 3.77 (m, 3H), 3.22 (app t, *J* = 9.4 Hz, 1H), 2.91 (app dt, *J* = 9.4, 2.9 Hz, 1H), 2.78 (dd, *J* = 18.6, 6.5 Hz, 1H), 2.49 – 2.40 (m, 2H), 2.40 – 2.29 (m, 1H), 1.68 (s, 3H), 1.55 – 1.47 (m, 2H), 1.43 (d, *J* = 7.3 Hz, 3H), 1.35 (d, *J* = 7.1 Hz, 3H), 1.34 – 1.24 (m, 10H), 0.89 (app t, *J* = 7.0 Hz, 3H). **^{13}C{^{1}H} NMR** (126 MHz, methanol-d_4): δ 176.5, 176.1, 175.5, 175.1, 172.3, 170.3, 90.9, 86.9, 76.2, 72.3, 70.1, 69.9, 51.5, 50.9, 48.0, 44.5, 44.2, 40.5, 38.4, 37.9, 33.0, 30.6, 30.4, 26.6, 23.7, 19.3, 17.6, 17.4, 14.4. **FTIR** (neat) cm^{-1}: 3287, 2925, 2855, 1643, 1533, 1272, 1230, 1123, 1065, 794, 630, 581. **HRMS** (APCI–) *m/z*: $[M - H]^-$ Calcd for $C_{29}H_{45}Cl_2N_4O_{10}$ 679.2518; Found 679.2523.

19'-*epi*-Acybolin B (19'-*epi*-5)

Side chain of 19'-*epi*-acybolin B (*epi*-16)

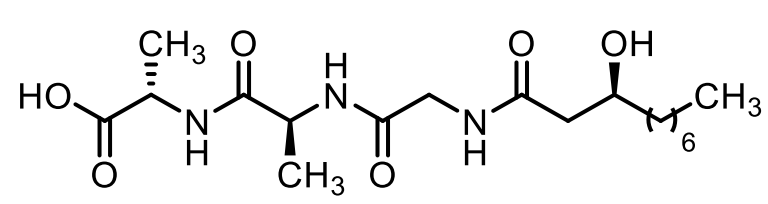

epi-**16**

<u>Side chain *epi*-**16**:</u> $[\alpha]_{589}^{23}$ = − 22.4 (*c* 0.7, CH_3OH). **^{1}H NMR** (300 MHz, methanol-d_4): δ 4.42 (q, *J* = 7.1 Hz, 1H), 4.34 (q, *J* = 7.3 Hz, 1H), 4.04 – 3.96 (m, 1H), 3.92 and 3.83 (ABq, *J* = 16.8 Hz, 2H), 2.42 (dd, *J* = 13.9, 4.3 Hz, 1H), 2.32 (dd, *J* = 14.0, 8.5 Hz, 1H), 1.55 – 1.45 (m, 2H), 1.40 (d, *J* = 7.3 Hz, 3H), 1.36 (d, *J* = 7.1 Hz, 3H), 1.32 (s, 10H), 0.90 (app t, *J* = 6.9 Hz, 3H). **^{13}C{^{1}H} NMR** (75 MHz, methanol-d_4): δ 175.9, 175.1, 174.5, 171.4, 69.9, 50.1, 49.5, 44.7, 43.6, 38.3, 33.0, 30.6, 30.4, 26.6, 23.7, 18.1, 17.7, 14.4. **FTIR** (neat) cm^{-1}: 3292, 3126, 2924, 2852, 1715, 1653, 1640, 1540, 1456, 1417, 1385, 1238, 1176, 1083, 1029, 942, 872. **HRMS** (APCI–) *m/z*: $[M -H]^-$ Calcd for $C_{18}H_{32}N_3O_6$ 386.2297; Found 386.2299.

Assembly of 19'-*epi*-acybolin B (19'-*epi*-5)

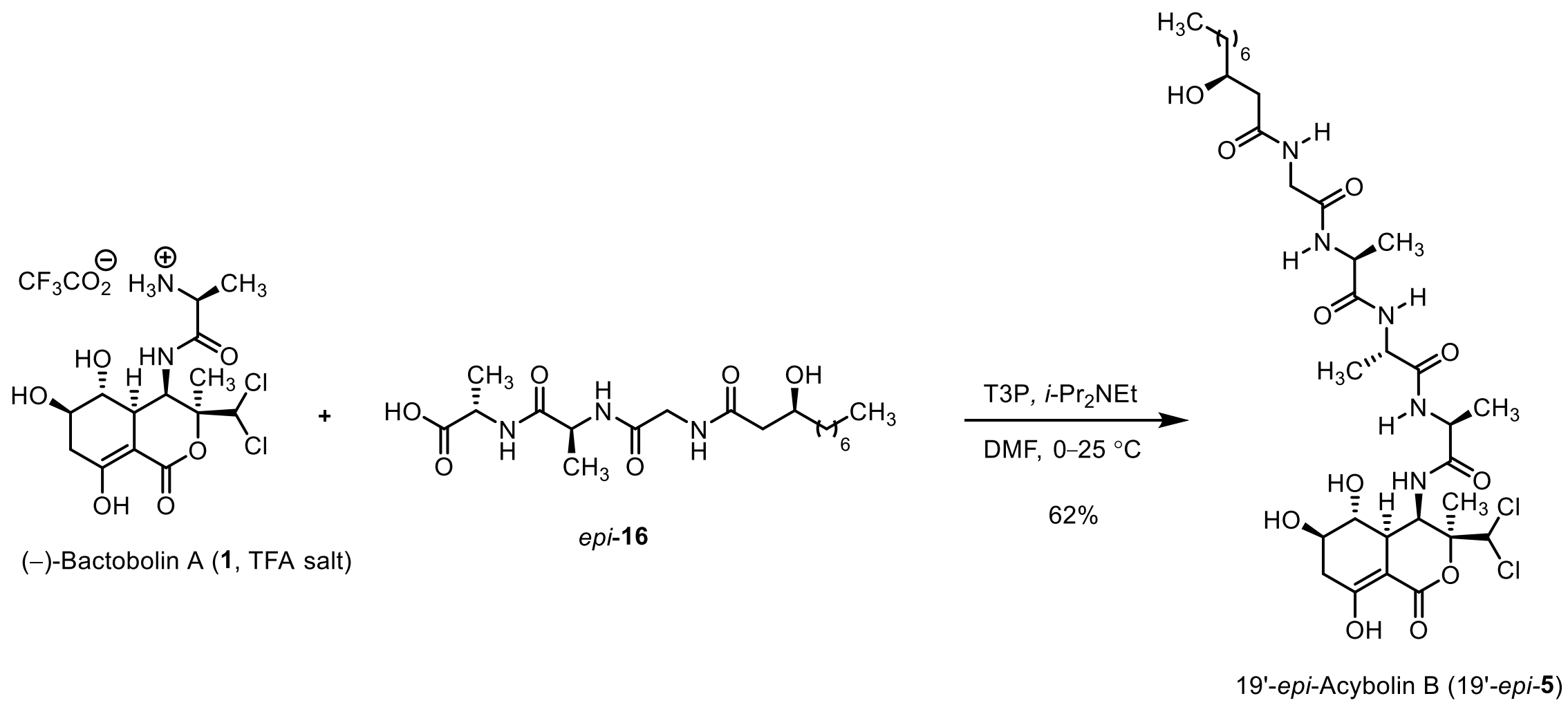

The general procedure described on page 9 was followed. This specific experiment used bactobolin A (**1**, TFA salt) (30 mg, 0.06 mmol, 1 equiv), side chain (*epi*-**16**) (46 mg, 0.12 mmol, 2.0 equiv), T3P reagent (36 μL, 0.12 mmol, 2.0 equiv), *N,N*-diisopropylethylamine (42 μL, 0.24 mmol, 4.0 equiv) in *N,N*-dimethylformamide (2.0 mL). The quench was performed with a 10% aqueous solution of lithium chloride (15 mL) and extraction with ethyl acetate (3 × 15 mL). 19'-*epi*-Acybolin B (19'-*epi*-**5**) was obtained as a white amorphous solid (28 mg, 62%) after purification.

19'-*epi*-Acybolin B (19'-*epi*-**5**): $[\alpha]_{589}^{23}$ = –18.3 (*c* 0.66, CH_3OH). **TLC** (10% methanol in dichloromethane; UV, $KMnO_4$): $R_f \approx 0.57$. **^{1}H NMR** (500 MHz, methanol-d_4): δ 6.33 (s, 1H), 4.85 (d, *J* = 3.8 Hz, 1H), 4.37 (q, *J* = 7.3 Hz, 1H), 4.17 (q, *J* = 7.3 Hz, 1H), 4.05 (q, *J* = 7.3 Hz, 1H), 4.02 – 3.96 (m, 1H), 3.90 and 3.84 (ABq, *J* = 16.5 Hz, 2H), 3.84 – 3.80 (m, 1H), 3.22 (app t, *J* = 9.3 Hz, 1H), 2.92 (app dt, *J* = 9.4, 2.9 Hz, 1H), 2.78 (dd, *J* = 18.5, 6.6 Hz, 1H), 2.45 (ddd, *J* = 18.6, 10.1, 2.5 Hz, 1H), 2.40 (dd, *J* = 14.0, 4.1 Hz, 1H), 2.34 (dd, *J* = 14.1, 8.8 Hz, 1H), 1.67 (s, 3H), 1.55 – 1.47 (m, 2H), 1.44 (d, *J* = 7.2 Hz, 3H), 1.40 (d, *J* = 7.3 Hz, 3H), 1.37 (d, *J* = 7.2 Hz, 3H), 1.35 – 1.25 (m, 10H), 0.90 (app t, *J* = 7.1 Hz, 3H). **^{13}C{^{1}H} NMR** (126 MHz, methanol-d_4): δ 176.4, 176.2, 176.1, 175.4, 175.4, 173.1, 170.3, 90.9, 86.9, 76.2, 72.4, 70.0, 69.9, 51.9 (2 × C), 50.8, 48.0, 44.4, 44.2, 40.4, 38.4, 38.0, 33.0, 30.6, 30.4, 26.6, 23.7, 19.3, 18.0, 17.2, 16.9, 14.4. **FTIR** (neat) cm^{-1}: 3454, 3154, 2928, 2855, 1654, 1536, 1456, 1233, 1173, 1126, 1067, 795. **HRMS** (APCI+) *m/z*: $[M + H]^+$ Calcd for $C_{32}H_{52}Cl_2N_5O_{11}$ 752.3035; Found 752.3042.

18'-*epi*-Acybolin H (18'-*epi*-6)

Side chain of 18'-*epi*-acybolin H (*epi*-15)

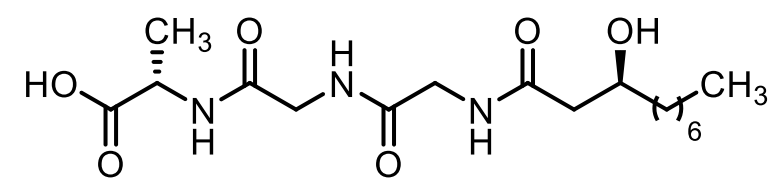

epi-**15**

<u>Side chain (*epi*-**15**):</u> $[\alpha]_{589}^{23}$ = –3.4 (*c* 0.53, CH_3OH) **^{1}H NMR** (500 MHz, methanol-d_4): δ 4.39 (q, *J* = 7.3 Hz, 1H), 4.02 – 3.96 (m, 1H), 3.93 / 3.86 and 3.93 / 3.88 (two ABq, *J* = 16.7 Hz, 4H), 2.43 (dd, *J* = 14.1, 4.1 Hz, 1H), 2.34 (dd, *J* = 14.1, 8.8 Hz, 1H), 1.52 – 1.46 (m, 2H), 1.41 (d, *J* = 7.3 Hz, 3H), 1.38 – 1.25 (m, 10H), 0.90 (app t, *J* = 7.1 Hz, 3H). **^{13}C{^{1}H} NMR** (126 MHz, methanol-d_4): δ 176.2, 175.2, 172.3, 171.1, 69.9, 49.6, 44.7, 43.8, 43.2, 38.4, 33.0, 30.6, 30.4, 26.6, 23.7, 17.8, 14.4. **FTIR** (neat) cm^{-1}: 3293, 2928, 2855, 1731, 1652, 1545, 1455, 1412, 1227. **HRMS** (APCI+) *m/z*: $[M + H]^+$ Calcd for $C_{17}H_{32}N_3O_6$ 374.2286; Found 374.2284.

Assembly of 18'-*epi*-acybolin H (18'-*epi*-6)

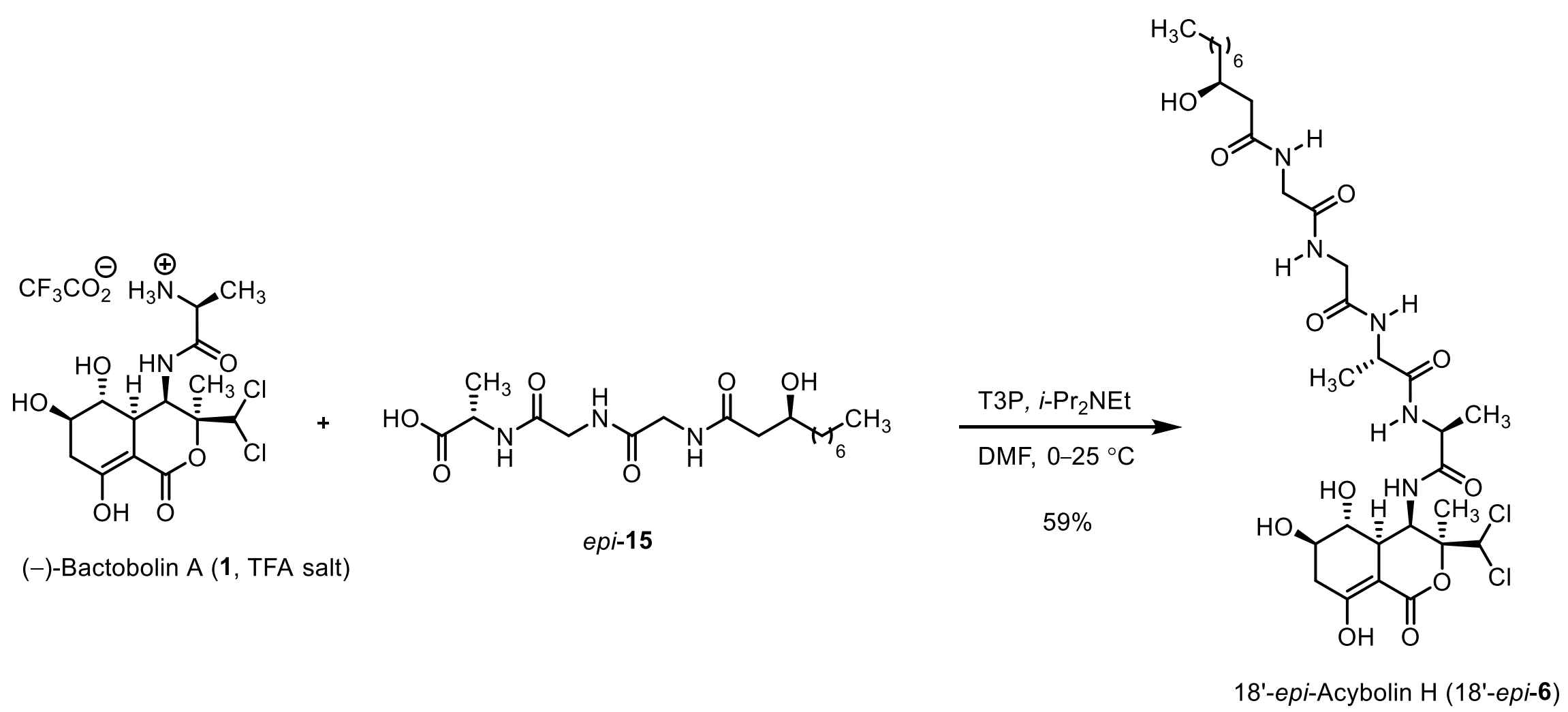

The general procedure described on page 9 was followed. The specific experiment used bactobolin A (TFA salt **1**, 30 mg, 0.06 mmol, 1 equiv), side chain *epi*-**15** (45 mg, 0.12 mmol, 2.0 equiv), T3P reagent (36 µL, 0.12 mmol, 2.0 equiv), *N,N*-diisopropylethylamine (42 µL, 0.24 mmol, 4.0 equiv) in *N,N*-dimethylformamide (2.0 mL). The quench was performed with 10% aqueous solution of

lithium chloride (15 mL) and extraction with ethyl acetate (3 × 15 mL). 18'-*epi*-Acybolin H (18'-*epi*-**6**) was obtained as a white amorphous solid (26 mg, 59%) after purification.

18'-*epi*-Acybolin H (18'-*epi*-**6**): $[\alpha]_{589}^{23}$ = –16.5 (*c* 0.56, CH_3OH). **TLC** (10% methanol in dichloromethane; UV, $KMnO_4$): $R_f \approx 0.67$. **^{1}H NMR** (500 MHz, methanol-d_4): δ 6.18 (s, 1H), 4.87 (d, *J* = 3.7 Hz, 1H), 4.33 (q, *J* = 7.3 Hz, 1H), 4.11 (q, *J* = 7.3 Hz, 1H), 4.04 – 3.94 (m, 2H), 3.90 and 3.88 (ABq, *J* = 6.0 Hz, 2H), 3.85 – 3.78 (m, 2H), 3.23 (app t, *J* = 9.4 Hz, 1H), 2.91 (app dt, *J* = 9.3, 2.7 Hz, 1H), 2.78 (dd, *J* = 18.6, 6.6 Hz, 1H), 2.46 – 2.38 (m, 2H), 2.35 (dd, *J* = 14.1, 8.8 Hz, 1H), 1.68 (s, 3H), 1.53 – 1.48 (m, 2H), 1.42 (d, *J* = 7.3 Hz, 3H), 1.38 (d, *J* = 7.2 Hz, 3H), 1.36 – 1.27 (m, 10H), 0.90 (app t, *J* = 7.1 Hz, 3H). **^{13}C{^{1}H} NMR** (126 MHz, methanol-d_4): δ 176.6, 176.2, 175.3, 175.2, 173.0, 172.0, 170.4, 90.9, 87.0, 76.2, 72.3, 70.0, 70.0, 51.6, 51.0, 47.9, 44.6, 44.1, 43.8, 40.5, 38.5, 38.0, 33.0, 30.7, 30.4, 26.6, 23.7, 19.3, 17.6, 17.4, 14.4. **FTIR** (neat) cm^{-1}: 3266, 2929, 2472, 2409, 1638, 1538, 1458, 1324, 1274, 1228, 1181, 1127, 1072, 848, 794, 633, 584, 479. **HRMS** (APCI+) *m/z*: $[M + H]^+$ Calcd for $C_{31}H_{50}Cl_2N_5O_{11}$ 738.2878; Found 738.2874.

22'-*epi*-Acybolin I (22'-*epi*-7)

Side chain of 22'-*epi*-acybolin I (*epi*-17)

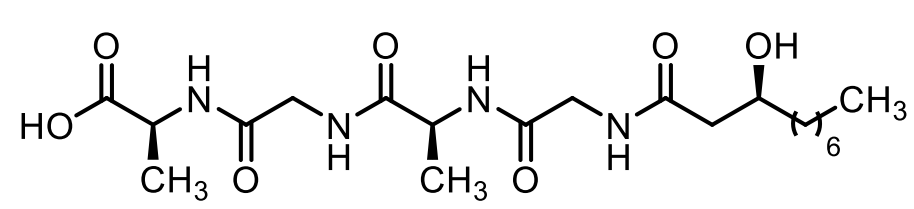

epi-**17**

Side chain (*epi*-**17**): $[\alpha]_{589}^{23}$ = –11.7 (*c* 0.52, CH_3OH) **^{1}H NMR** (500 MHz, methanol-d_4): δ 4.39 (q, *J* = 7.4 Hz, 1H), 4.34 (q, *J* = 7.2 Hz, 1H), 4.02 – 3.97 (m, 1H), 3.95 and 3.83 (ABq, *J* = 16.9 Hz, 2H), 3.90 and 3.86 (ABq, *J* = 16.7 Hz, 2H), 2.42 (dd, *J* = 14.0, 4.2 Hz, 1H), 2.33 (dd, *J* = 14.0, 8.7 Hz, 1H), 1.53 – 1.45 (m, 2H), 1.41 (d, *J* = 7.3 Hz, 3H), 1.38 (d, *J* = 7.2 Hz, 3H), 1.35 – 1.25 (m, 10H), 0.90 (app t, *J* = 7.1 Hz, 3H). **^{13}C{^{1}H} NMR** (126 MHz, methanol-d_4): δ 176.1, 175.4, 175.2, 171.8, 171.1, 69.9, 50.8, 49.6, 44.6, 43.7, 43.4, 38.4, 33.0, 30.7, 30.4, 26.6, 23.7, 17.8, 17.6, 14.4. **FTIR** (neat) cm^{-1}: 3310, 2928, 1749, 1733, 1717, 1698, 1683, 1669, 1653, 1648, 1637, 1575, 1558, 1541, 1522, 1508, 1457. **HRMS** (APCI+) *m/z*: $[M + H]^+$ Calcd for $C_{20}H_{37}N_4O_7$ 445.2657; Found 445.2655.

Assembly of 22'-*epi*-acybolin I (22'-*epi*-7)

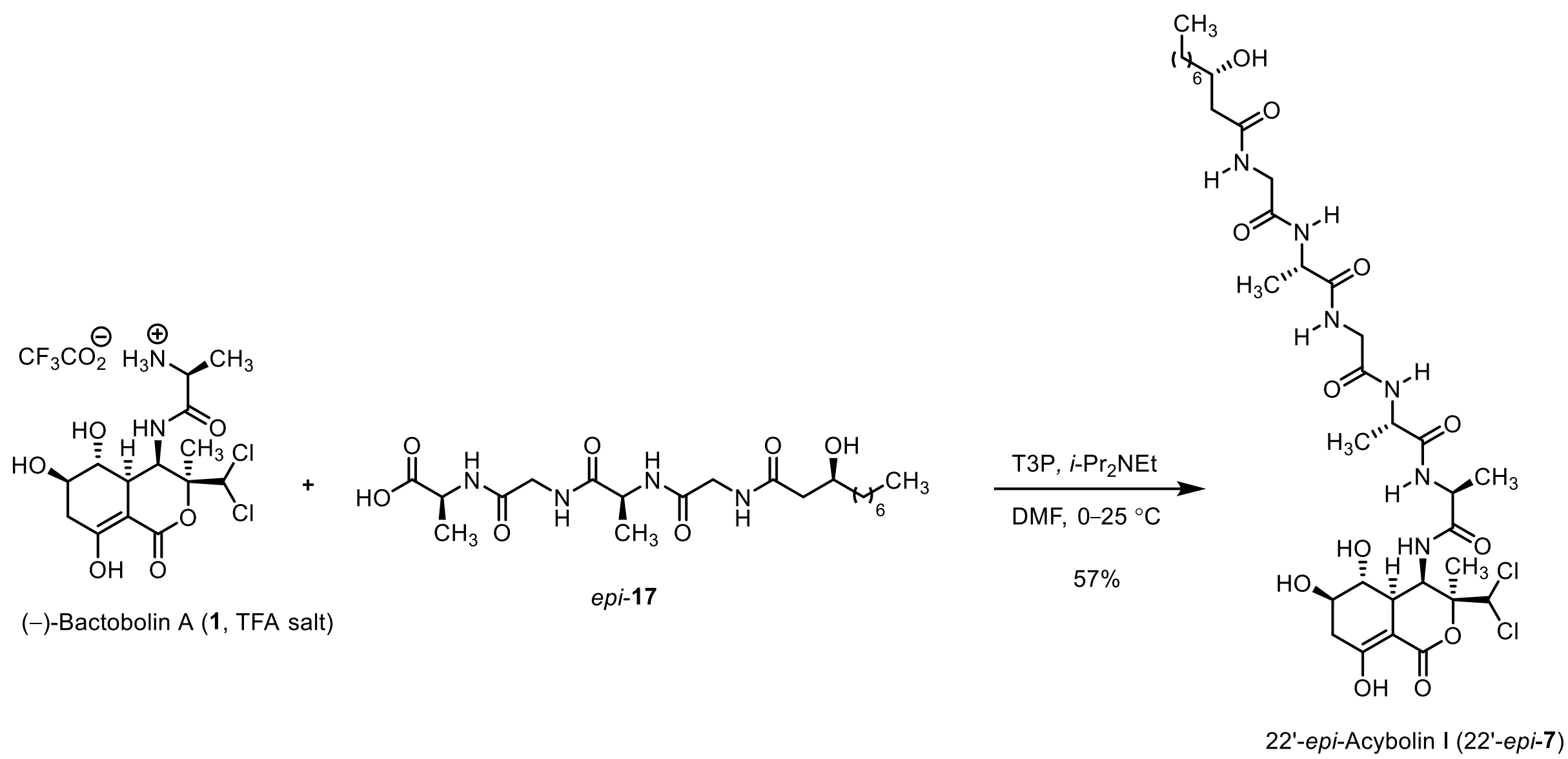

The general procedure described on page 9 was followed. The specific experiment used bactobolin A (**1**, TFA salt) (30 mg, 0.06 mmol, 1 equiv), side chain (*epi*-**17**) (53 mg, 0.12 mmol, 2.0 equiv), T3P reagent (36 µL, 0.12 mmol, 2.0 equiv), *N,N*-diisopropylethylamine (42 µL, 0.24 mmol, 4.0 equiv) in dimethylformamide (2.0 mL). The quench was performed with a 10% aqueous solution of lithium chloride (15 mL) and extraction with ethyl acetate (3 × 15 mL), respectively. 22'-*epi*-Acybolin I (22'-*epi*-**7**) was obtained as a white amorphous solid (28 mg, 57%) after purification.

<u>22'-*epi*-Acybolin I (22'-*epi*-**7**):</u> $[\alpha]_{589}^{23}$ = –16.7 (c = 0.62, CH_3OH). **TLC** (10% methanol in dichloromethane; UV, $KMnO_4$): $R_f \approx 0.34$. **^{1}H NMR** (500 MHz, methanol-d_4): δ 6.32 (s, 1H), 4.86 (d, *J* = 3.7 Hz, 1H), 4.37 (q, *J* = 7.3 Hz, 1H), 4.27 (q, *J* = 7.2 Hz, 1H), 4.15 (q, *J* = 7.3 Hz, 1H), 4.03 – 3.95 (m, 1H), 3.89 (app s, 2H), 3.84 (dd, *J* = 9.8, 6.6 Hz, 1H), 3.81 (app s, 2H), 3.21 (app t, *J* = 9.3 Hz, 1H), 2.92 (app dt, *J* = 9.3, 2.6 Hz, 1H), 2.79 (dd, *J* = 18.7, 6.6 Hz, 1H), 2.44 (ddd, *J* = 18.8, 10.2, 2.5 Hz, 1H), 2.41 (dd, *J* = 14.0, 4.1 Hz, 1H), 2.33 (dd, *J* = 14.1, 8.8 Hz, 1H), 1.68 (s, 3H), 1.55 – 1.45 (m, 2H), 1.42 (app t, *J* = 7.2 Hz, 6H), 1.38 (d, *J* = 7.2 Hz, 3H), 1.35 – 1.27 (m, 10H), 0.90 (app t, *J* = 7.1 Hz, 3H). **^{13}C{^{1}H} NMR** (126 MHz, methanol-d_4): δ 176.7, 176.4, 176.1, 175.4, 175.2, 172.4, 172.1, 170.3, 90.8, 86.9, 76.2, 72.4, 70.0, 70.0, 51.7, 51.4, 50.9, 47.9, 44.5, 44.2, 43.9, 40.5, 38.4, 38.0, 33.0, 30.7, 30.4, 26.6, 23.7, 19.3, 17.8, 17.4 (2 × C), 14.4. **FTIR** (neat) cm^{-1}: 3296, 2927, 2856, 1650, 1537, 1455, 1383, 1232, 1124, 1068, 795, 582. **HRMS** (APCI+) *m/z*: $[M + H]^+$ Calcd for $C_{34}H_{55}Cl_2N_6O_{12}$ 809.3250; Found 809.3244.

Synthesis of 19'-*epi*-acybolin B via bactobolin E (linear synthesis)

Synthesis of protected bactobolin E (34)

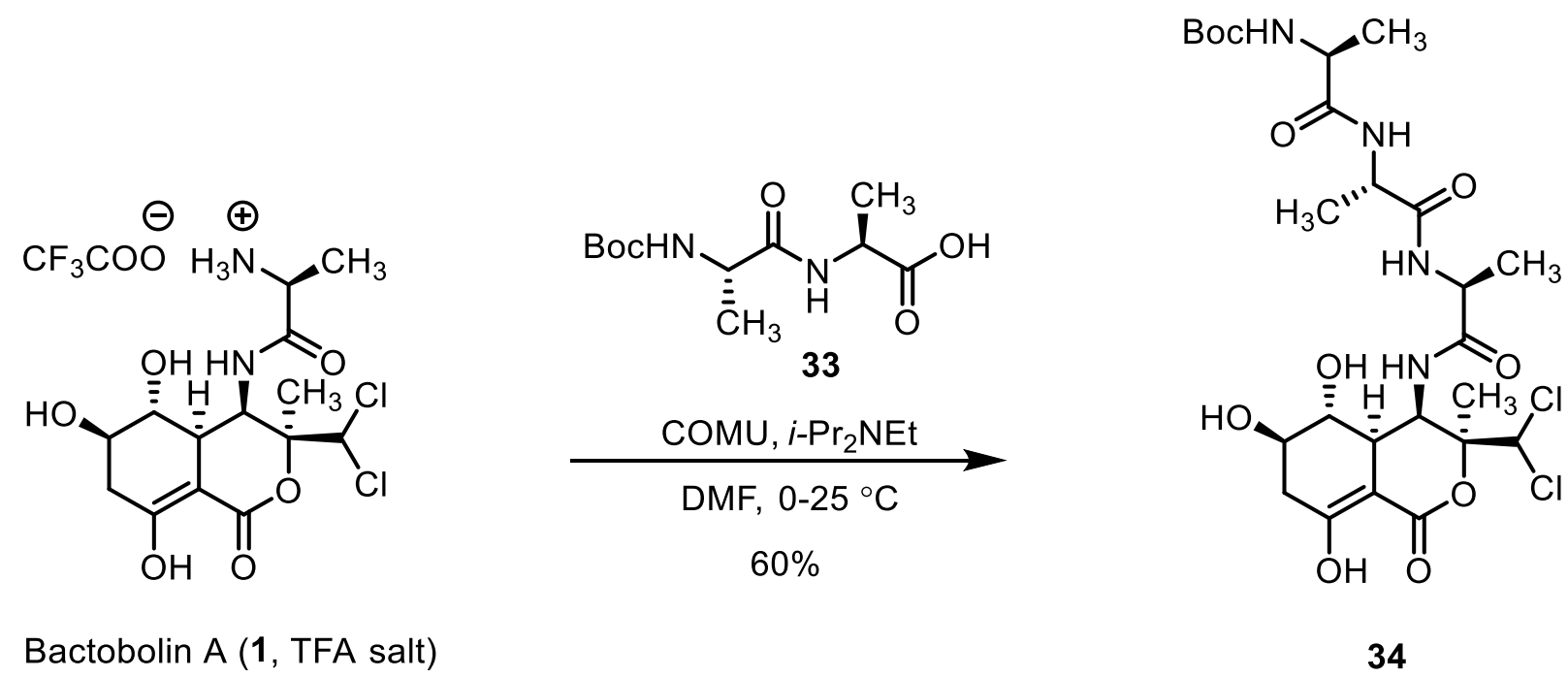

To a solution of dipeptide **33** (105 mg, 0.40 mmol, 2.0 equiv) in anhydrous *N,N*-dimethylformamide (2 mL) was added the first portion of *N,N*-diisopropylethylamine (70 µL, 0.40 mmol, 2.0 equiv) at 0 °C, and the mixture was stirred for 5 min. Then, COMU (172 mg, 0.40 mmol, 2.0 equiv) was added, and the reaction mixture was stirred at 0 °C for 30 min. The solution of bactobolin A (**1**, TFA salt) (100 mg, 0.20 mmol, 1 equiv) in anhydrous *N,N*-dimethylformamide (3.0 mL) was added to the above reaction mixture at 0 °C, followed by the addition of the second portion of *N,N*-diisopropylethylamine (105 µL, 0.60 mmol, 3.0 equiv). The reaction mixture was stirred at 0 °C for 30 min, then slowly warmed to 23 °C and stirred at this temperature for 40 h (complete reaction according to TLC analysis). The reaction mixture was quenched by the addition of a saturated aqueous solution of ammonium chloride (10 mL), followed by a 10 % aqueous solution of lithium chloride (30 mL). The reaction mixture was extracted with ethyl acetate (3 × 20 mL). The combined organic layers were washed with a saturated aqueous solution of sodium bicarbonate (3 × 15 mL) and brine (2 × 15 mL). The washed combined organic extracts were dried over anhydrous sodium sulfate, filtered, and concentrated under reduced pressure. The crude residue was purified by flash column chromatography (gradient elution with 1−5% methanol in dichloromethane) to afford the target compound **34** as a white amorphous solid (97 mg, 77% yield).

Protected bactobolin E (**34**): (10% methanol in dichloromethane; UV, $KMnO_4$): $R_f \approx 0.4$. **^{1}H NMR** (500 MHz, methanol-d_4): δ 6.41 (s, 1H), 4.84 (d, *J* = 3.9 Hz, 1H), 4.45 (q, *J* = 7.2 Hz, 1H), 4.04 – 3.95 (m, 2H), 3.84 (app td, *J* = 9.8, 6.6 Hz, 1H), 3.20 (app t, *J* = 9.4 Hz, 1H), 2.96 – 2.89 (m, 1H), 2.78 (dd, *J* = 18.6, 6.7 Hz, 1H), 2.40 (ddd, *J* = 18.7, 10.0, 2.5 Hz, 1H), 1.68 (s, 3H), 1.50 (s, 9H), 1.46 (d, *J* = 7.4 Hz, 3H), 1.35 (d, *J* = 7.3 Hz, 3H), 1.30 (d, *J* = 7.2 Hz, 3H). **^{13}C{^{1}H} NMR** (126

MHz, methanol-d_4): δ 177.7, 176.3, 176.0, 175.5, 170.3, 159.2, 91.1, 86.9, 81.4, 76.3, 72.5, 69.9, 52.9, 52.2, 50.4, 48.1, 40.3, 37.9, 28.8 (3 × C), 19.3, 18.4, 17.4, 17.1. **HRMS** (APCI+) *m/z*: [M + H]$^+$ Calcd for $C_{25}H_{39}Cl_2N_4O_{10}$ 625.2038; Found 625.2043.

Synthesis of bactobolin E (3, TFA salt)

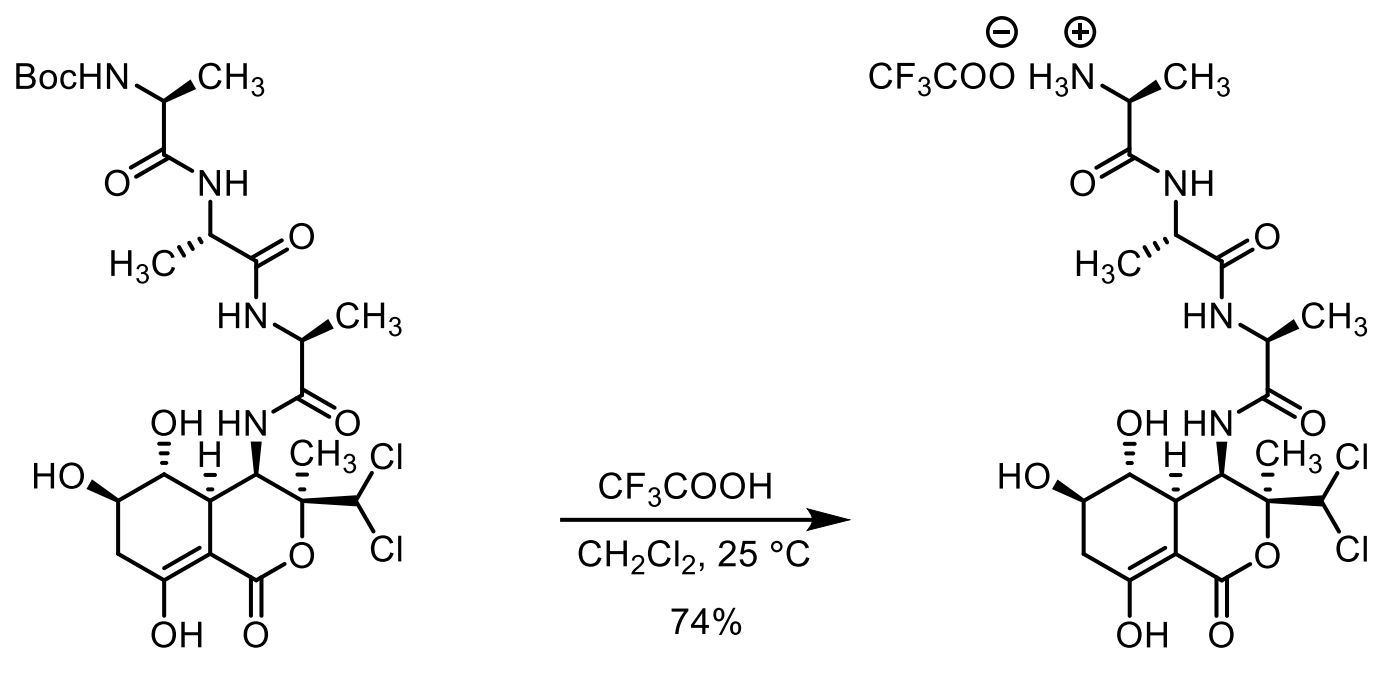

34 Bactobolin E (**3**,TFA salt)

Trifluoroacetic acid (0.5 mL) was added to a solution of protected bactobolin E (**34**) (75 mg, 0.12 mmol, 1 equiv) in dichloromethane (10 mL) at 25 °C. The reaction mixture was stirred for 35 h, then the solvent was evaporated under reduced pressure. The crude residue was purified by flash column chromatography (gradient elution with 5−20% methanol in dichloromethane) to afford the bactobolin E[4] (**3**, TFA salt) as a white amorphous solid (76 mg, 99% yield).

Bactobolin E (**3**, TFA salt): $[\alpha]_{589}^{23}$ = –25.8 (*c* 0.59, CH_3OH). TLC (10% methanol in dichloromethane; UV, $KMnO_4$): $R_f \approx 0.1$. **^{1}H NMR** (500 MHz, methanol-d_4): δ 6.24 (s, 1H), 4.88 (d, *J* = 3.6 Hz, 1H), 4.33 (q, *J* = 7.1 Hz, 2H), 3.97 (q, *J* = 7.0 Hz, 1H), 3.86 (app td, *J* = 9.7, 6.8 Hz, 1H), 3.35 (s, 1H), 3.17 (app t, *J* = 9.4 Hz, 1H), 2.92 (app dt, *J* = 9.3, 2.6 Hz, 1H), 2.85 (dd, *J* = 18.8, 6.8 Hz, 1H), 2.39 (ddd, *J* = 18.8, 10.0, 2.5 Hz, 1H), 1.68 (s, 3H), 1.53 (d, *J* = 7.0 Hz, 3H), 1.38 (app t, *J* = 6.9 Hz, 6H). **^{13}C{^{1}H} NMR** (126 MHz, methanol-d_4): δ 177.1, 175.9, 174.5, 171.1, 170.3, 90.7, 87.0, 76.1, 72.5, 69.9, 50.7, 50.6, 50.2, 47.6, 40.3, 38.1, 19.1, 18.0, 17.6, 17.6. **^{19}F NMR** (471 MHz, methanol-d_4): δ –76.88. **FTIR** (neat) cm^{-1}: 3418, 3274, 2923, 1667, 1548, 1236, 1202, 1132, 1070, 840, 800, 723. **HRMS** (APCI–) *m/z*: [M – H]$^-$ Calcd for $C_{22}H_{30}Cl_2F_3N_4O_{10}$ 637.1297; Found 637.1294.

Synthesis of 19'-*epi*-acybolin B (19'-*epi*-5)

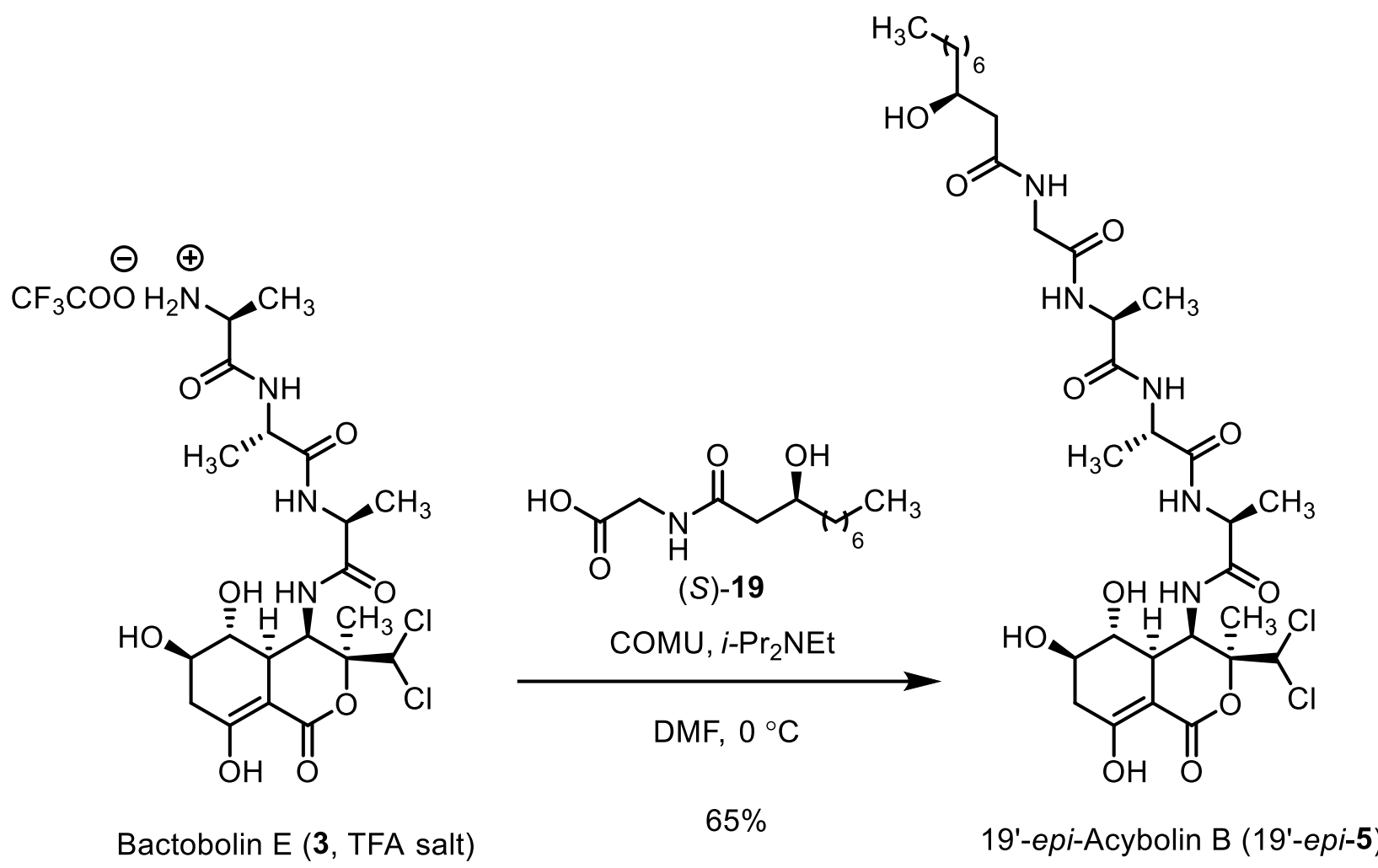

Side chain (*S*)-**19** (15 mg, 0.06 mmol, 2.0 equiv) was added to a dry round-bottom flask equipped with a magnetic stirring bar. The flask was evacuated and backfilled with argon, then anhydrous *N,N*-dimethylformamide (1.0 mL) was added. The resulting solution was cooled to 0 °C in an ice bath, and the first portion of *N,N*-diisopropylethylamine (11 μL, 0.06 mmol, 2.0 equiv) was added. After stirring for 5 min at 0 °C, COMU (27 mg, 0.06 mmol, 2.0 equiv) was added. After stirring for 30 min at 0 °C, a solution of bactobolin E (**3**, TFA salt) (20 mg, 0.03 mmol, 1 equiv) in anhydrous *N,N*-dimethylformamide (1.0 mL) was added at 0 °C, followed by the addition of the second portion of *N,N*-diisopropylethylamine (16 μL, 0.09 mmol, 3.0 equiv). The reaction mixture was stirred at 0 °C for 60 min. Then, the reaction mixture was quenched with a saturated aqueous solution of ammonium chloride (2.0 mL) and extracted with ethyl acetate (3 × 20 mL). Combined organic extracts were sequentially washed with 10% aqueous solution of lithium chloride (2 × 10 mL), a saturated aqueous solution of sodium bicarbonate (2 × 10 mL), and brine (15 mL). The washed organic extracts were dried over anhydrous sodium sulfate, filtered, and concentrated under reduced pressure. The crude residue was purified by flash column chromatography (gradient elution with 5−10% methanol in dichloromethane) to afford the target 19'-*epi*-acybolin B (19'-*epi*-**5**) as a white amorphous solid (15 mg, 65% yield). NMR analysis of 19'-*epi*-acybolin B has reported on page 27.

Comparison of ^{1}H and ^{13}C NMR data for natural acybolin B, synthetic acybolin B, and synthetic 19'-*epi*-acybolin B.

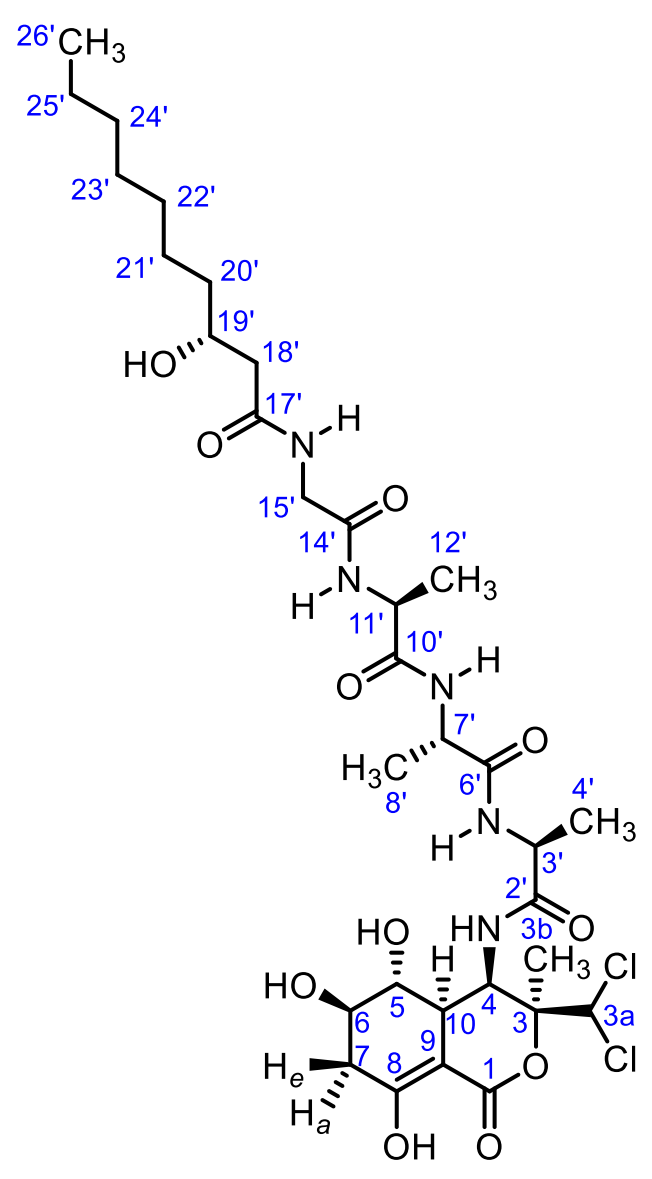

Acybolin B (**5**)

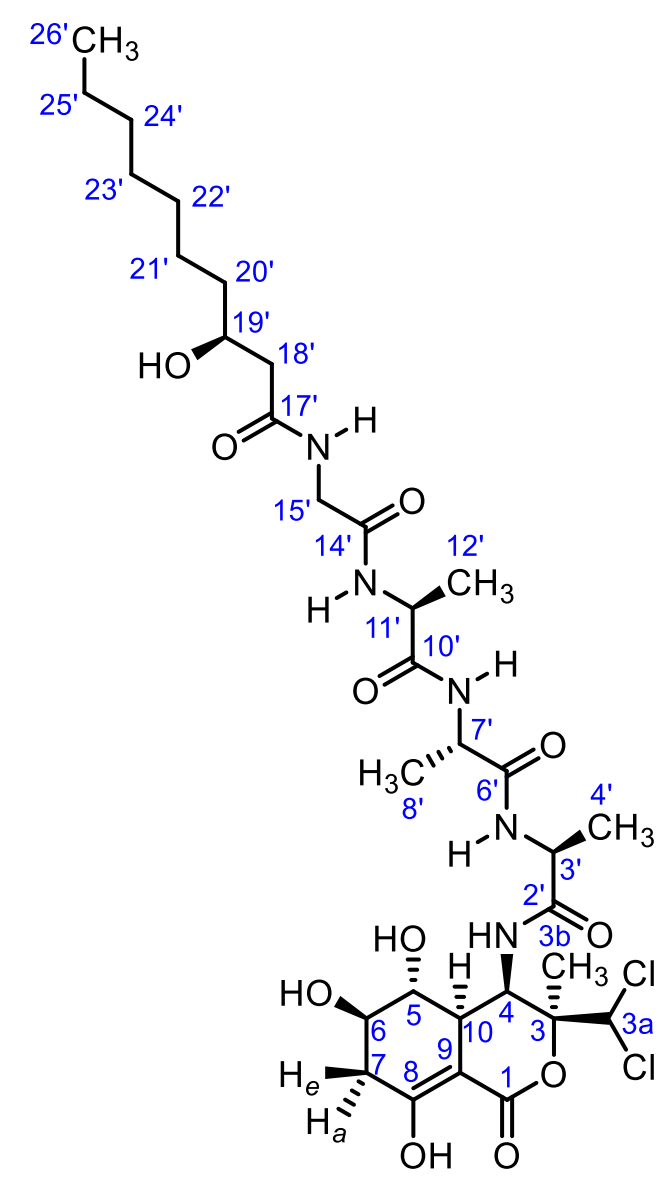

19'-*epi*-Acybolin B (19'-*epi*-**5**)

^{1}H NMR data comparison

Hydrogen atom position	natural acybolin B[5] (^{1}H NMR 800 MHz, methanol-d_4)	synthetic acybolin B (^{1}H NMR 500 MHz, methanol-d_4)	Δppm	synthetic 19'-*epi*-acybolin B (^{1}H NMR 500 MHz, methanol-d_4)	Δppm
1					
2					
3a	6.29 (s, 1H)	6.31 (s, 1H)	0.02	6.33 (s, 1H)	0.04
3b	1.64 (s, 3H)	1.67 (s, 3H)	0.03	1.67 (s, 3H	0.03
4	4.81 (d, *J* = 3.5 Hz, 1H)	4.85 (d, *J* = 3.8 Hz, 1H)	0.04	4.85 (d, *J* = 3.8 Hz, 1H)	0.04
5	3.18 (t, *J* = 9.3 Hz, 1H)	3.21 (app t, *J* = 9.3 Hz, 1H)	0.03	3.22 (app t, *J* = 9.3 Hz, 1H)	0.04
6	3.79 (1 H)	3.84 (dd, *J* = 9.9, 6.6 Hz, 1H)	0.05	3.82 (m, 1H)	0.03
7 (H_e)	2.75 (dd, *J* = 18.5, 6.5 Hz, 1H)	2.78 (dd, *J* = 18.6, 6.7 Hz, 1H)	0.03	2.78 (dd, *J* = 18.5, 6.6 Hz, 1H)	0.03
7 (H_a)	2.4 (1H)	2.44 (m, 1H)	0.04	2.45 (ddd, *J* = 18.6, 10.1, 2.5 Hz, 1H)	0.05
8					
9					

10	2.89 (m, 1H)	2.92 (app dt, *J* = 9.3, 2.7 Hz, 1H)	0.03	2.92 (app dt, *J* = 9.4, 2.9 Hz, 1H)	0.03
2´					
3´	4.34 (q, *J* = 7.2 Hz, 1H)	4.37 (q, *J* = 7.3 Hz, 1H)	0.03	4.37 (q, *J* = 7.3 Hz, 1H)	0.03
4´	1.41 (d, *J* = 7.1 Hz, 3 H)	1.43 (d, *J* = 7.3 Hz, 3 H)	0.02	1.44 (d, *J* = 7.2 Hz, 3 H)	0.03
6´					
7´	4.16 (q, *J* = 7.3 Hz, 1 H)	4.18 (q, *J* = 7.2 Hz, 1 H)	0.02	4.17 (q, *J* = 7.3 Hz, 1 H)	0.01
8´	1.35 (d, *J* = 7.4 Hz, 3 H)	1.37 (d, *J* = 7.3 Hz, 3 H)	0.02	1.37 (d, *J* = 7.2 Hz, 3 H)	0.02
10´					
11´	4.04 (q, *J* = 7.3 Hz, 1H)	4.06 (q, *J* = 7.3 Hz, 1H)	0.02	4.05 (q, *J* = 7.3 Hz, 1H)	0.01
12´	1.37 (d, *J* = 7.5 Hz, 3 H)	1.39 (d, *J* = 7.3 Hz, 3 H)	0.02	1.40 (d, *J* = 7.3 Hz, 3 H)	0.03
14´					
15´(Ha)	3.93 (d, *J* = 16.4 Hz, 1 H)	3.94 (d, *J* = 16.5 Hz, 1 H)	0.01	3.90 (ABq, *J* = 16.5 Hz, 1 H)	-0.03
15´(Hb)	3.77 (d, *J* = 16.5 Hz, 1H)	3.80 (d, *J* = 16.4 Hz, 1H)	0.03	3.83 (ABq *J* = 16.5 Hz, 1H)	0.06
17´					
18´(Ha)	2.41 (dd, *J* = 14.1, 4.7 Hz, 1H)	2.44 (m, 1H)	0.03	2.40 (dd, *J* = 14.0, 4.1 Hz, 1H)	-0.01
18´(Hb)	2.31 (dd, *J* = 14.1, 8.1 Hz, 1H)	2.32 (dd, *J* = 14.2, 8.1 Hz, 1H)	0.01	2.34 (dd, *J* = 14.1, 8.8 Hz, 1H)	0.03
19´	3.95 (1H)	3.98 (m, 1H)	0.03	3.99 (m, 1H)	0.04
20´	1.47 (m, 2H)	1.51 (m, 2H)	0.04	1.51 (m, 2H)	0.04
21´(Ha)	1.42 (1H)	1.43 (1H)	0.01	1.44 (1H)	0.02
21´(Hb)	1.31 (1H)	1.33 (1H)	0.02	1.34 (1H)	0.03
22´	1.29 (2H)	1.32 (2H)	0.03	1.33 (2H)	0.04
23´		1.32 (2H)		1.33 (2H)	
24´	1.27 (2H)	1.30 (2H)	0.03	1.32 (2H)	0.05
25´	1.28 (2H)	1.31 (2H)	0.03	1.31 (2H)	0.03
26´	0.88 (t, *J* = 7.0 Hz, 3H)	0.90 (app t, *J* = 7.1 Hz, 3H)	0.02	0.90 (app t, *J* = 7.1 Hz, 3H)	0.02

^{13}C NMR data comparison

Carbon atom position	natural acybolin B[5] ($^{13}C\{^1H\}$ NMR 200 MHz, methanol-d_4)	synthetic acybolin B ($^{13}C\{^1H\}$ NMR 126 MHz, methanol-d_4)	Δ_{ppm}	synthetic 19'-*epi*-acybolin B ($^{13}C\{^1H\}$ NMR 126 MHz, methanol-d_4)	Δ_{ppm}
1		170.3		170.3	
3	85.2	86.9	1.7	86.9	1.7
3a	74.7	76.2	1.5	76.2	1.5
3b	17.8	19.3	1.5	19.3	1.5
4	46.7	48.0	1.3	48.0	1.3
5	71.0	72.4	1.4	72.4	1.4
6	68.6	70.0	1.4	70.0	1.4
7	37.0	38.0	1.0	38.0	1.0
8	175.4	176.4	1.0	176.4	1.0
9	89.1	90.9	1.8	90.9	1.8
10	38.8	40.4	1.6	40.4	1.6
2′	174.7	176.2	1.5	176.2	1.5
3′	49.2	50.7	1.5	50.8	1.6
4′	16.6	18.0	1.4	18.0	1.4
6′	174.6	176.1	1.5	176.1	1.5
7′	50.3	51.8	1.5	51.9	1.6
8′	15.8	17.0	1.2	16.9	1.1
10′	173.8	175.3	1.5	175.4	1.6
11′	50.3	51.8	1.5	51.9	1.6
12′	15.5	17.3	1.8	17.2	1.7
14′	171.5	173.0	1.5	173.1	1.6
15′	42.5	44.0	1.5	44.2	1.7
17′	173.7	175.2	1.5	175.4	1.7
18′	43.1	44.5	1.4	44.4	1.3
19′	68.4	69.9	1.5	69.9	1.5
20′	36.9	38.3	1.4	38.4	1.5
21′	25.2	26.6	1.4	26.6	1.4
22′	29.1	30.4	1.3	30.4	1.3
23′		30.7		30.6	
24′	31.5	33.0	1.5	33.0	1.5
25′	22.3	23.7	1.4	23.7	1.4
26′	12.9	14.4	1.5	14.4	1.5

Biological studies

Cell culture

Human cancer cell lines MCF7 (ATCC), HCC38 (ATCC), HeLa (DSMZ), and MDA-MB-231 (ATCC) were maintained under standard culture conditions at 37 °C in a humidified atmosphere containing 5% CO_2. MCF7 and MDA-MB-231 cells were cultured in DMEM, while HCC38 and HeLa DSMZ cells were cultured in RPMI-1640, all supplemented with 10% fetal bovine serum and 1% penicillin–streptomycin. Cells were routinely tested for mycoplasma contamination and were used at low passage numbers.

Compound treatment and dose–response setup

Dose-response experiments were performed in 384-well plates using an automated liquid handling system (epMotion 5075, Eppendorf). Cells were seeded at a density optimized for each cell line to ensure exponential growth during the assay period and were allowed to attach overnight. Compounds were prepared as DMSO stock solutions and dispensed into assay plates using serial dilutions to generate a concentration–response series (50, 20, 8, 3.2, 1.28, 0.512, 0.2048, 0.08192 µM). Final DMSO concentration was kept constant across all wells (max 0.5%). Automated dispensing ensured uniform compound delivery and minimized technical variability. Each concentration was tested in technical quadruplicate, and vehicle-treated wells served as controls for normalization. The experiment was performed in two independent repetitions.

CellTiter-Glo viability assay

Cell viability was assessed using the CellTiter-Glo Luminescent Cell Viability Assay (Promega) according to the manufacturer's instructions. Following compound treatment, CellTiter-Glo reagent was added directly to the wells using automated dispensing, the plates were shaken to induce cell lysis, and luminescence was measured after signal stabilization using a Tecan Spark system. Raw luminescence values were normalized to the mean of vehicle control wells and expressed as a percentage of DMSO-treated controls. Normalized viability data from two independent experiments were pooled for analysis. Mean viability values and SEM were calculated for each compound and concentration. Dose–response profiles are shown in Supplementary Figures S1–S4; IC_{50} values were determined in GraphPad Prism 11 (Sigmoidal 4PL).

Annexin V (ANX)/Propidium iodide (PI) assay

Apoptosis was analyzed (Figure S5) using an Annexin V assay (EXBIO ApoFlowEx FITC Kit, #ED7044) according to the manufacturer's instructions and as previously described.[6] Following compound treatment (**1**, 1 μM; **3**, 1 μM; 19'-*epi*-**5**, 20 μM), HeLa cells, including floating populations, were collected, stained with Annexin V and propidium iodide, and analyzed by flow cytometry using a BD FACSVerse instrument. Experiments were performed in two independent biological repeats.

Cell cycle analysis

HeLa cells were treated with compounds **1**, 1 μM; **3**, 1 μM; 19'-*epi*-**5**, 20 μM, or vehicle control (DMSO ≤0.5%) (Figure S6) and collected at indicated time points (1, 4, 24, 48, and 72 h). Cells were harvested, including floating populations, fixed in 70% ethanol, and stained with DAPI as previously described.[7] DNA content was analyzed using a BD FACSVerse flow cytometer (BD Biosciences), and cell cycle distribution (G1, S, G2/M) was quantified using ModFit LT software. Experiments were performed in two independent biological repeats.

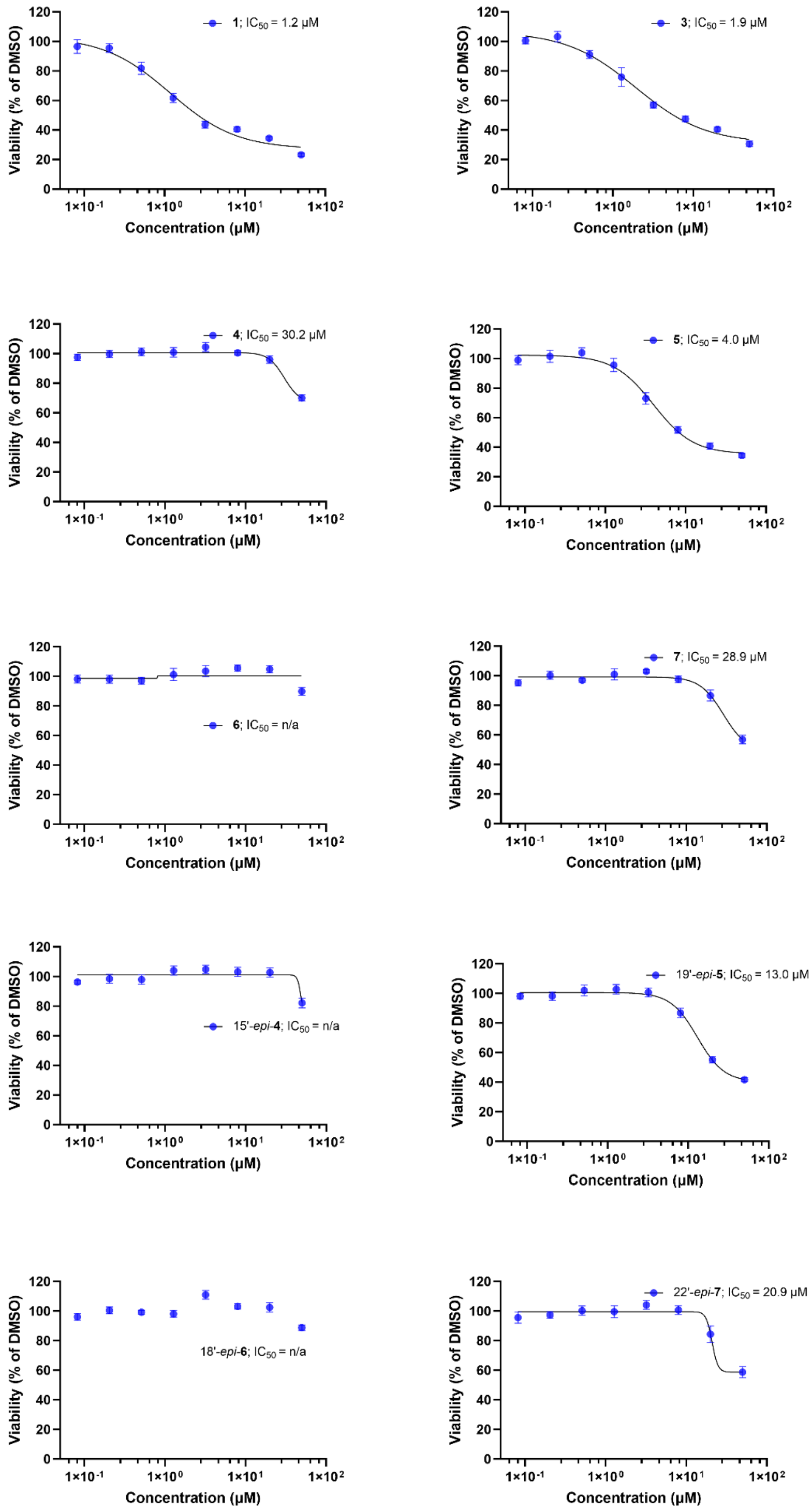

Figure S1: MDA-MB-231 cell line. Block-based dose–response profiles of the compound series measured by CellTiter-Glo viability assays. Data represent pooled measurements from two independent experiments following normalization to DMSO controls. Points indicate mean viability (% of DMSO), and error bars represent SEM. Dose-response curves and IC50s were determined in GraphPad Prism 11.

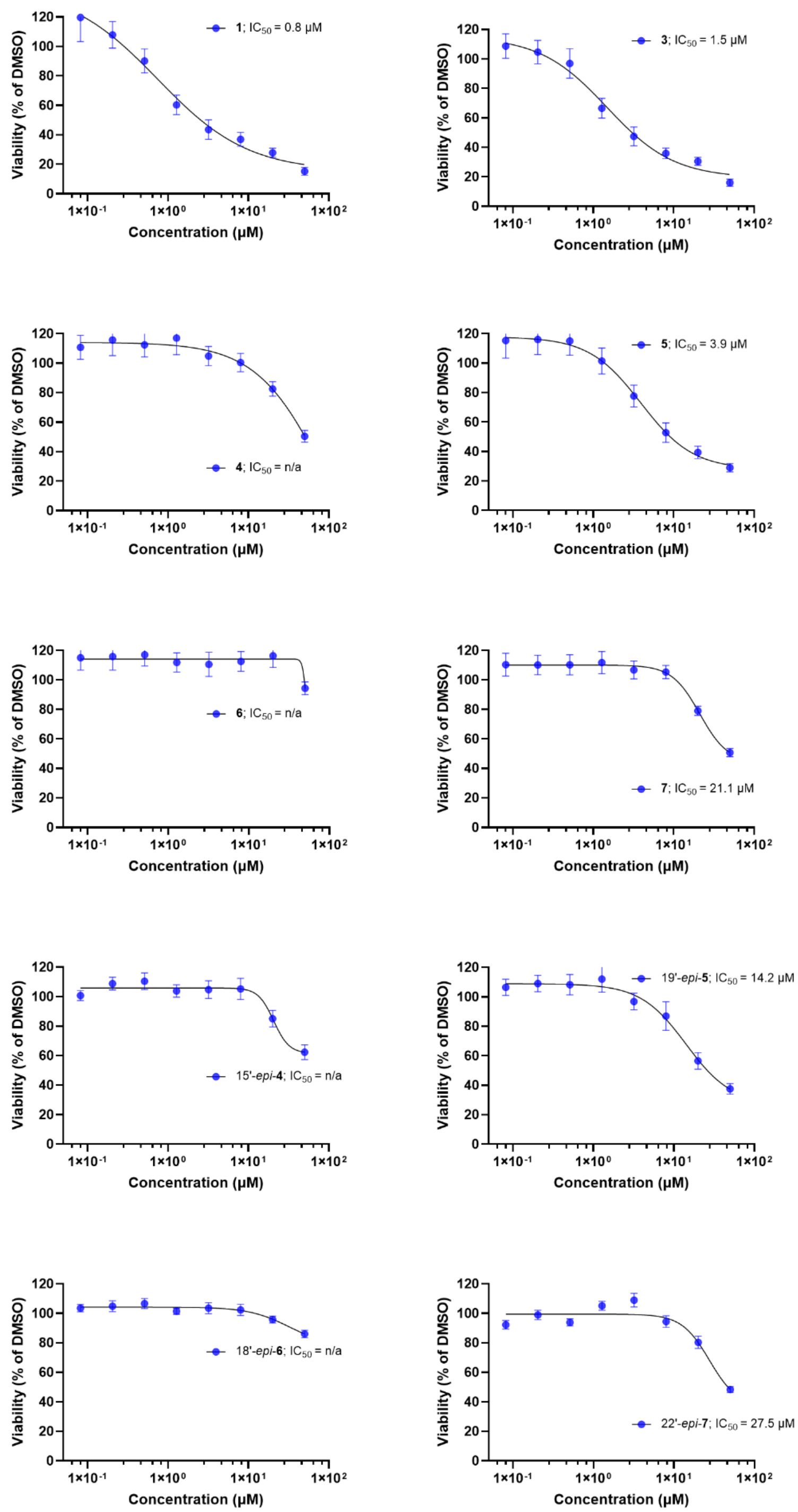

Figure S2: HCC38 cell line. Block-based dose–response profiles of the compound series measured by CellTiter-Glo viability assays. Data represent pooled measurements from two independent experiments following normalization to DMSO controls. Points indicate mean viability (% of DMSO), and error bars represent SEM. Dose-response curves and IC50s were determined in GraphPad Prism 11.

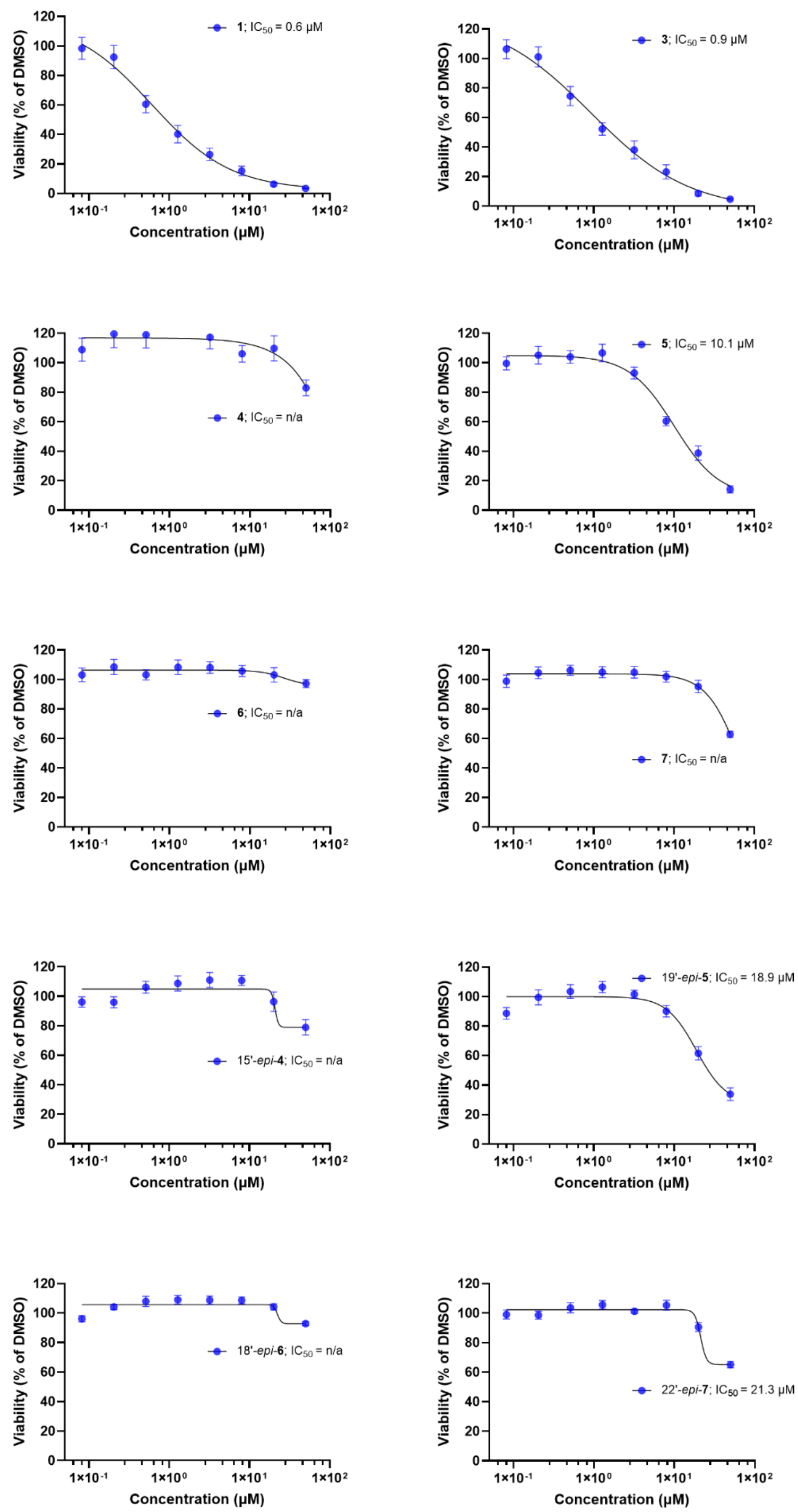

Figure S3: HeLa cell line. Block-based dose–response profiles of the compound series measured by CellTiter-Glo viability assays. Data represent pooled measurements from two independent experiments following normalization to DMSO controls. Points indicate mean viability (% of DMSO), and error bars represent SEM. Dose-response curves and IC50s were determined in GraphPad Prism 11.

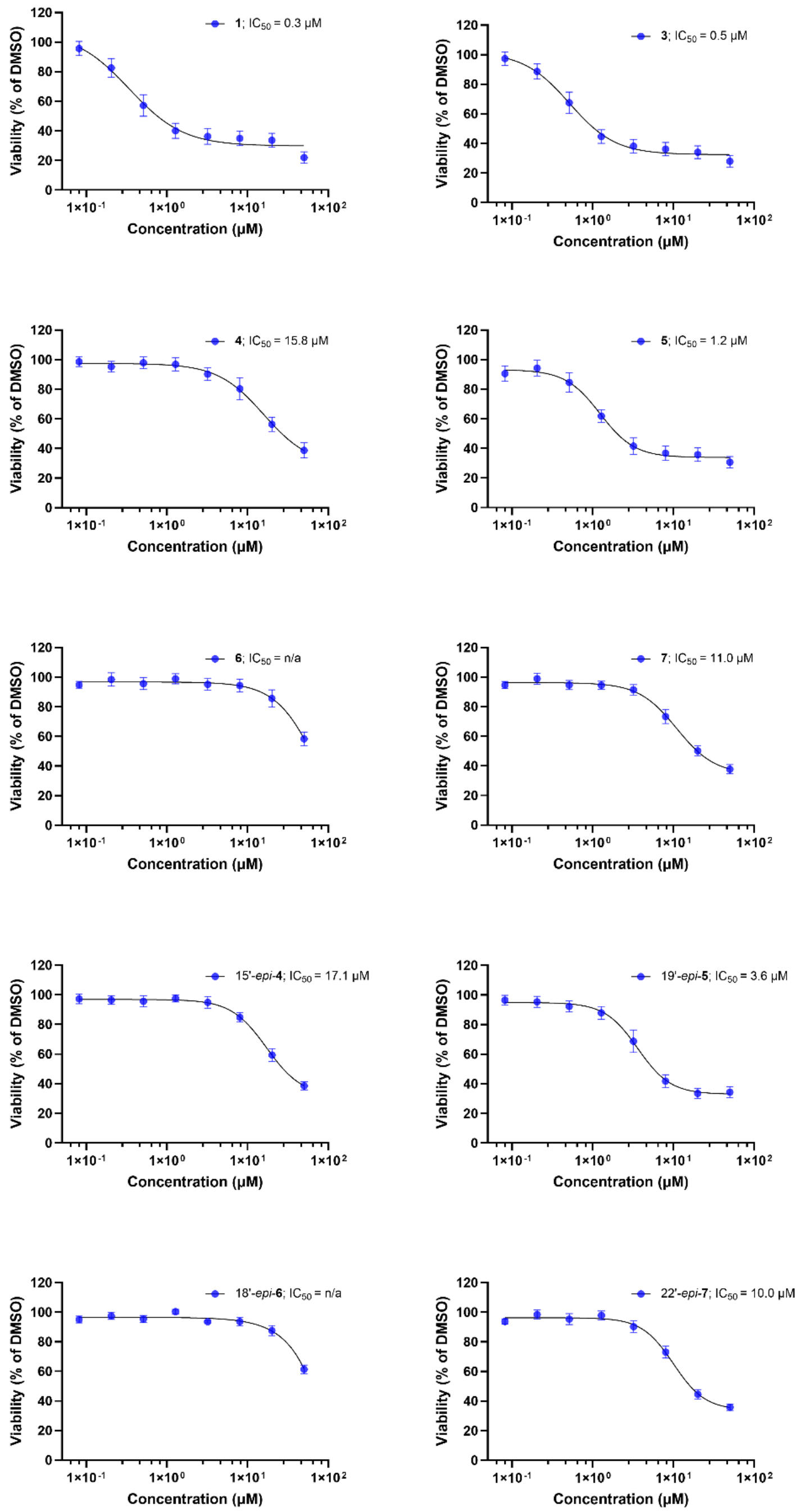

Figure S4: MCF7 cell line. Block-based dose–response profiles of the compound series measured by CellTiter-Glo viability assays. Data represent pooled measurements from two independent experiments following normalization to DMSO controls. Points indicate mean viability (% of DMSO), and error bars represent SEM. Dose-response curves and IC50s were determined in GraphPad Prism 11.

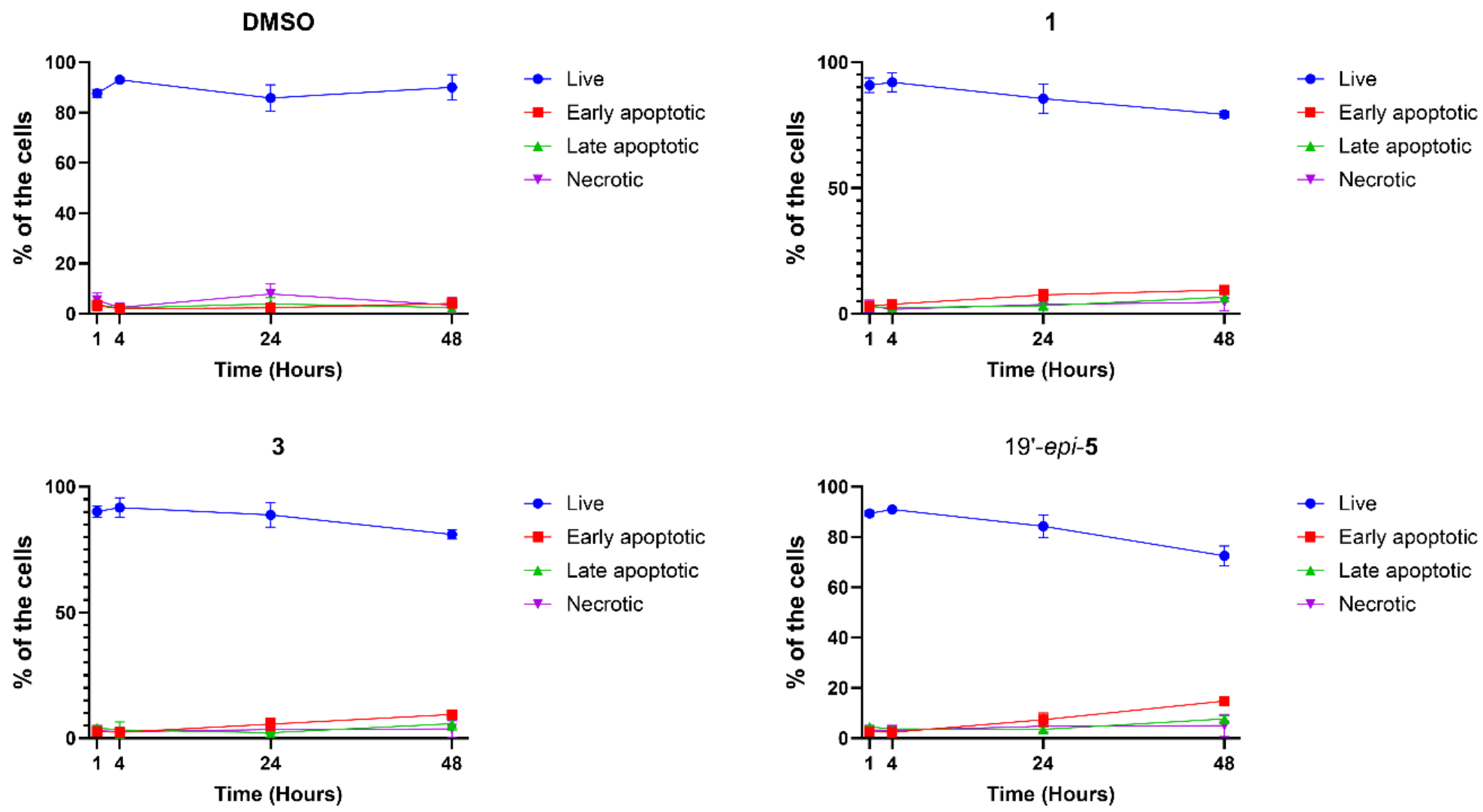

Figure S5: Quantification of apoptosis following compound treatment using an Annexin V/Propidium iodide assay. HeLa cells were stained and analyzed by flow cytometry as described in Methods. Values represent mean ± SD from two independent experiments.

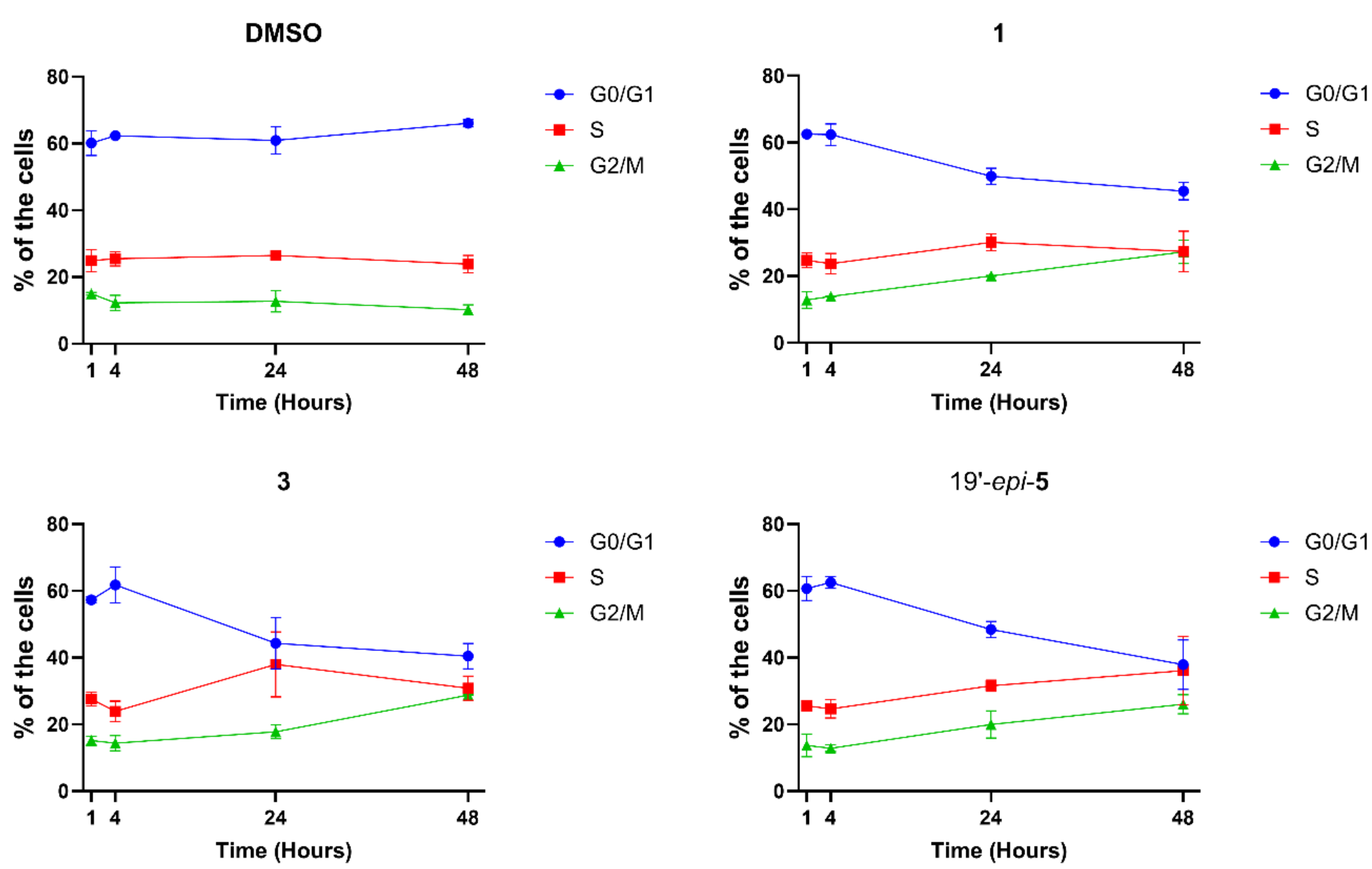

Figure S6: Time-dependent cell cycle distribution following compound treatment at minimal effective concentrations. HeLa cells were fixed in 70% ethanol, stained with DAPI, and analyzed by flow cytometry (BD FACSVerse). Cell cycle phases were quantified using ModFit LT software. Graphs show the relative proportions of cells in G1, S, and G2/M phases over time. Data represent mean ± SD from two independent experiments.

References

1. Zhu, C.; Shen, X.; Nelson, S. G. *J. Am. Chem. Soc.* **2004**, *126*, 5352–5353.
2. Vleeschouwer, M. De.; Sinnaeve, D.; Begin, J. Van. Den.; Coenye, T.; Martins, J.C.; Madder, A. *Chem. Eur. J.* **2014**, *20*, 7766–7775.
3. Lemieux, R. U.; Giguère, P. A. *Can. J. Chem.* **1951**, *29*, 678–690.
4. Carr, G.; Seyedsayamdost, M. R.; Chandler, J. R.; Greenberg, E. P.; Clardy, J. *Org. Lett.* **2011**, *13*, 3048–3051.
5. Okada, B. K.; Wu, Y.; Mao, D.; Bushin, L. B.; Seyedsayamdost, M. R. *ACS Chem. Biol.* **2016**, *11*, 2124–2130.
6. Drápela, S.; Khirsariya, P.; Weerden, W. M. van.; Fedr, R.; Suchánková, T.; Búzová, D.; Červený, J.; Hampl, A.; Puhr, M.; Watson, W. R.; Culig, Z.; Krejčí, L.; Paruch, K.; Souček, K. *Mol. Oncol.* **2020**, *14*, 2487–2503.
7. Fedr, R.; Kahounová, Z.; Remšík, J.; Reiterová, M.; Kalina, T.; Souček, K.; *Sci. Rep.* **2023**, *13*, 4889.

Copies of HPLC Chromatograms

HPLC analyses of racemic and enantiomerically enriched 13

Chiralpak IA column, 250 × 4.6 mm, λ = 254 nm, 20% isopropyl alcohol in hexane, flow rate: 1.0 mL/min.

Figure S7: HPLC chromatogram of (±)-**13**.

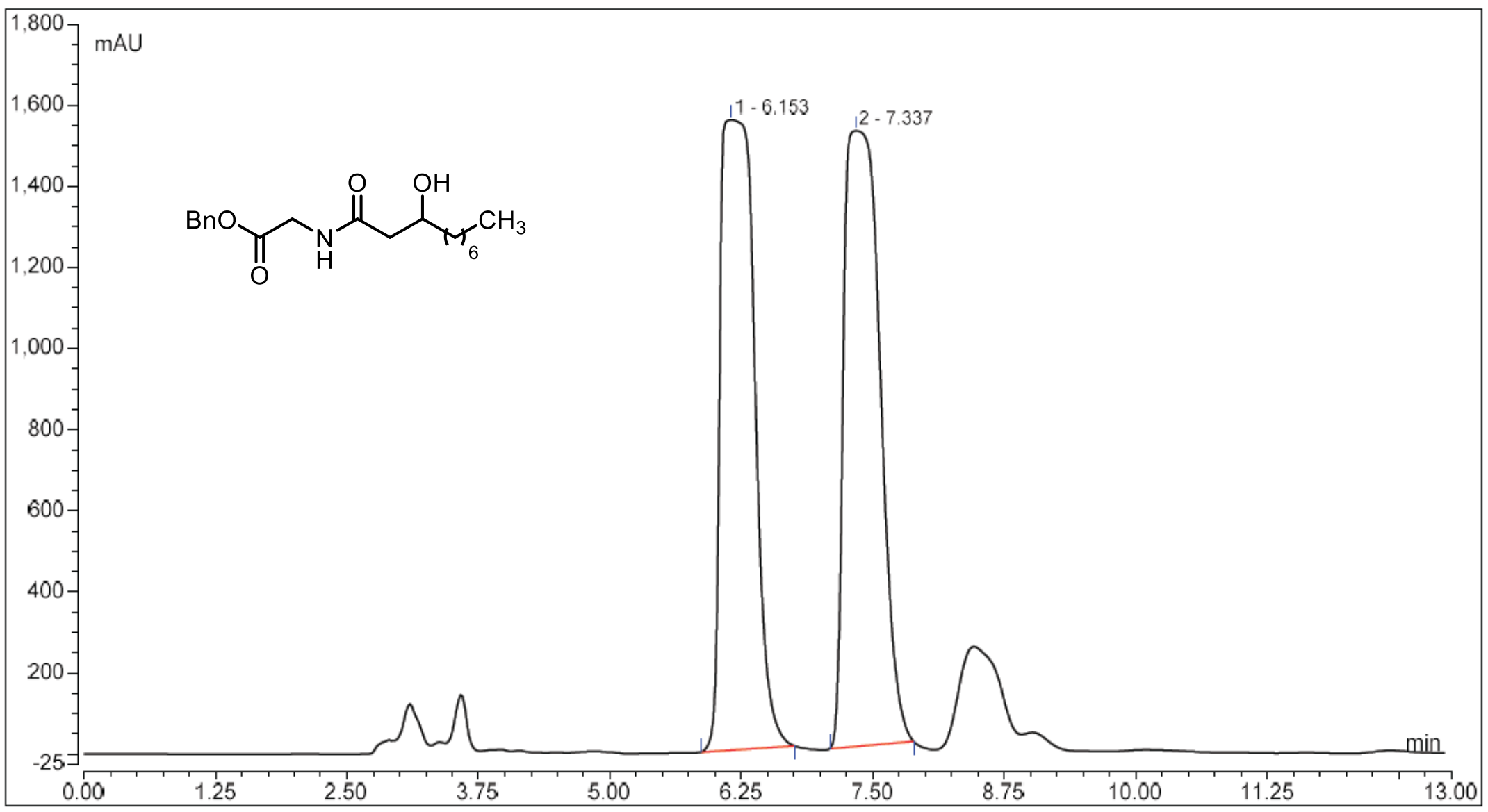

Peak	Retention Time (min)	Area (mAU × min)	Height (mAU)	Relative Area (%)
1	6.153	580.970	1554.222	49.52
2	7.337	600.889	1518.264	50.48
Total		1181.860	3072.486	100

Figure S8: HPLC chromatogram of enantiomerically enriched (*S*)-**13**

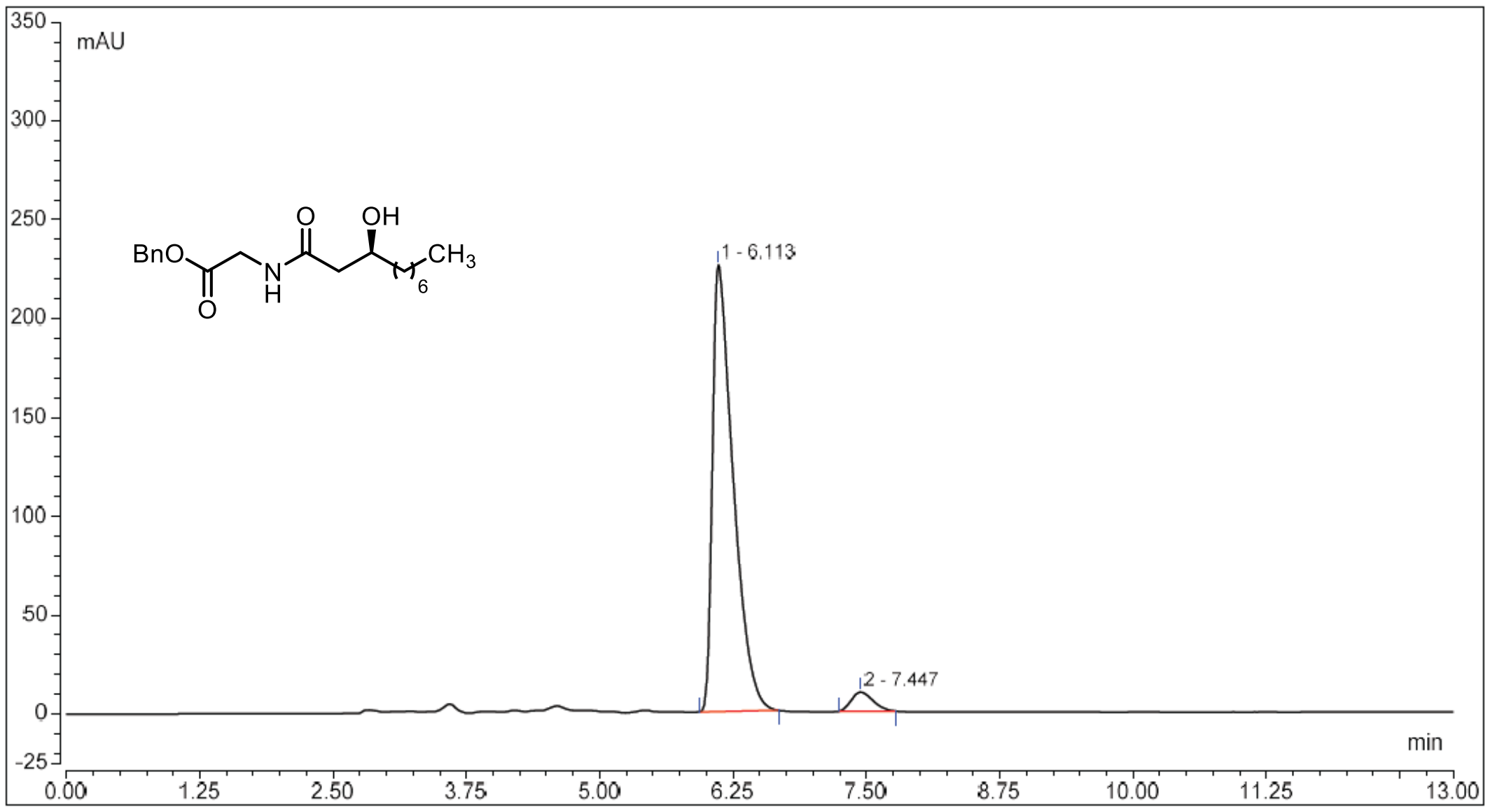

Peak	Retention Time (min)	Area	Height	Relative Area (%)
1	6.113	51.063	225.978	95.71
2	7.447	2.287	9.683	4.29
Total		53.350	235.661	100

Figure S9: HPLC chromatogram of enantiomerically enriched (*R*)-**13**

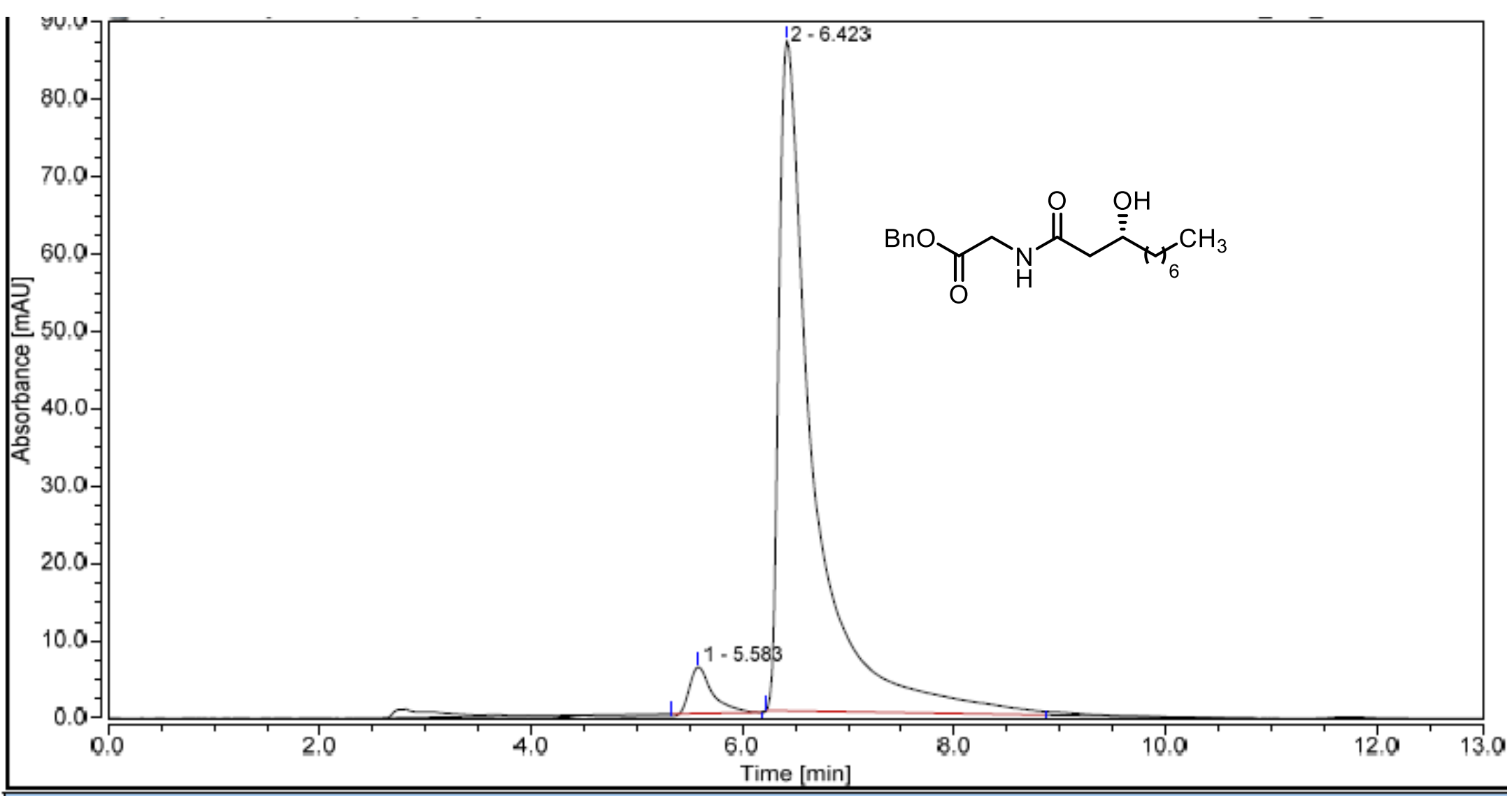

Integration Results

No.	Peak Name	Retention Time min	Area mAU*min	Height mAU	Relative Area %	Relative Height %	Amount n.a.
1		5.583	1.489	6.014	4.33	6.49	n.a.
2		6.423	32.869	86.723	95.67	93.51	n.a.
**Total:**			**34.358**	**92.737**	**100.00**	**100.00**	

COPIES OF NMR SPECTRA

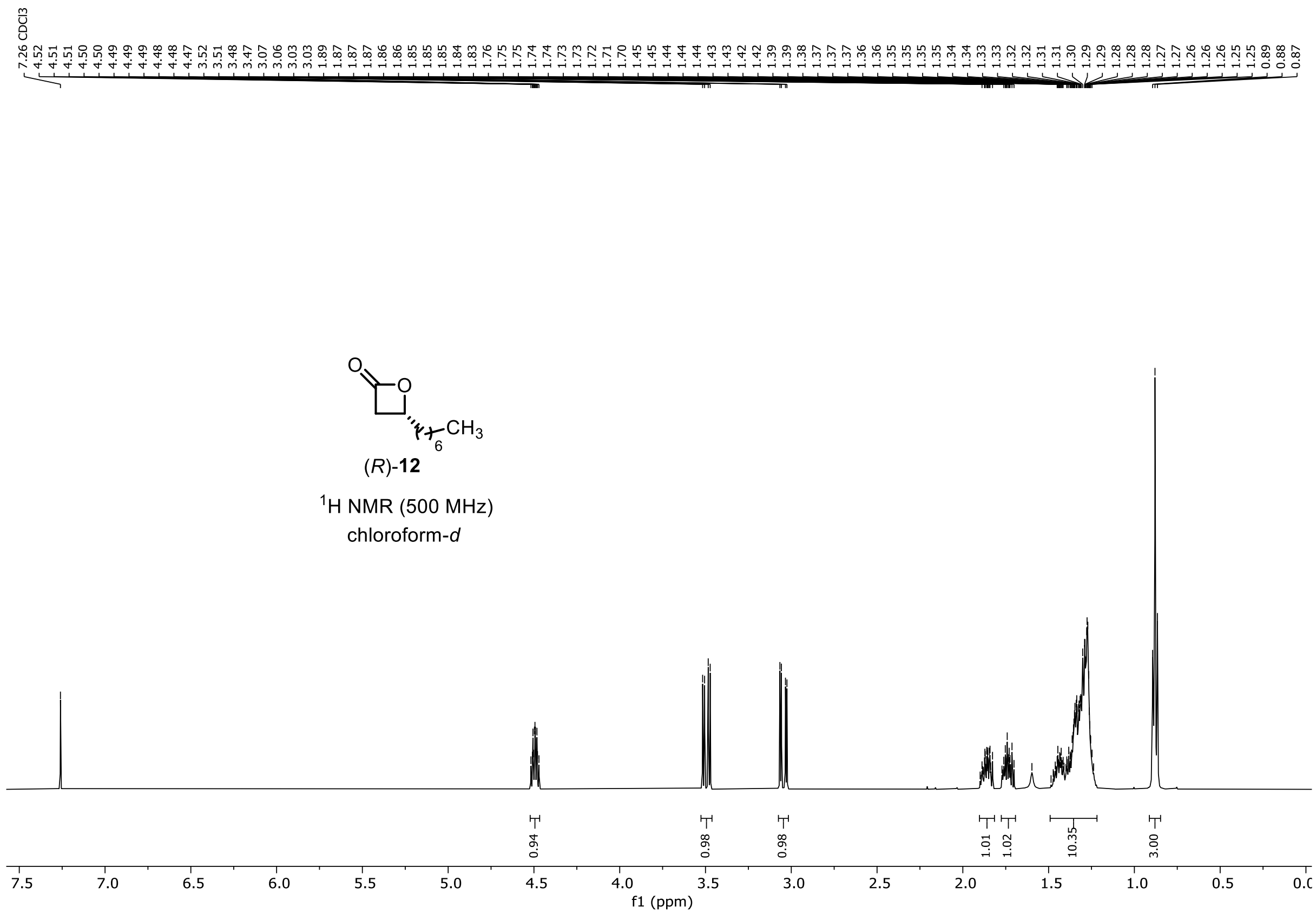
7.26 CDCl3
4.52 4.51 4.51 4.50 4.50 4.49 4.49 4.49 4.48 4.48 4.47 3.52 3.51 3.48 3.47 3.07 3.06 3.03 3.03 1.89 1.87 1.87 1.87 1.86 1.86 1.85 1.85 1.85 1.84 1.83 1.76 1.75 1.75 1.74 1.74 1.73 1.73 1.72 1.71 1.70 1.45 1.45 1.44 1.44 1.44 1.43 1.43 1.42 1.42 1.39 1.39 1.38 1.37 1.37 1.37 1.36 1.35 1.35 1.35 1.35 1.34 1.33 1.33 1.32 1.32 1.31 1.31 1.30 1.29 1.29 1.28 1.28 1.28 1.27 1.27 1.26 1.26 1.25 1.25 0.89 0.88 0.87
(*R*)-**12**
^{1}H NMR (500 MHz)
chloroform-*d*
0.94
0.98
0.98
1.01
1.02
10.35
3.00
f1 (ppm)

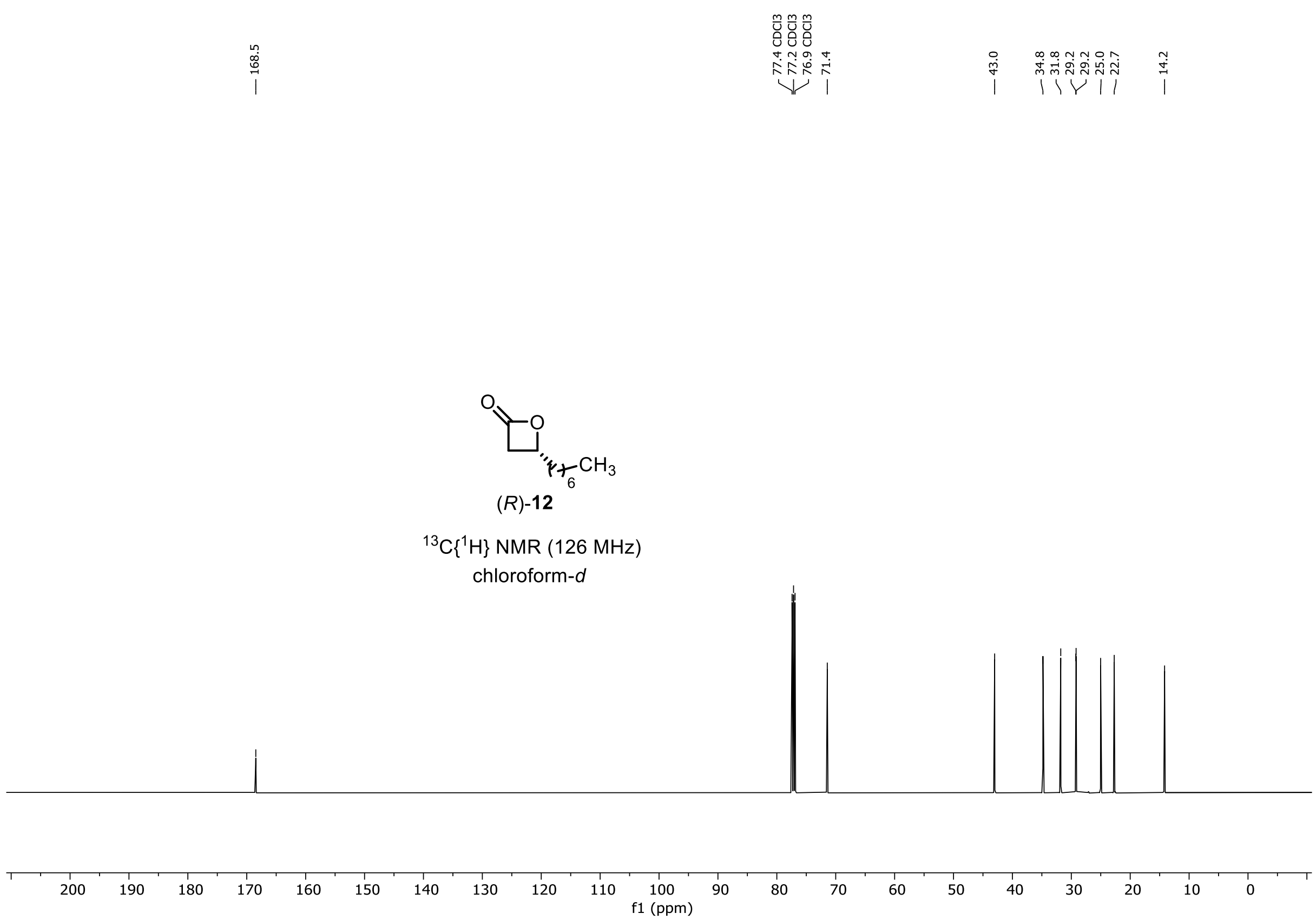
168.5
77.4 CDCl3
77.2 CDCl3
76.9 CDCl3
71.4
43.0
34.8
31.8
29.2
29.2
25.0
22.7
14.2
(*R*)-**12**
^{13}C{^{1}H} NMR (126 MHz)
chloroform-*d*
f1 (ppm)

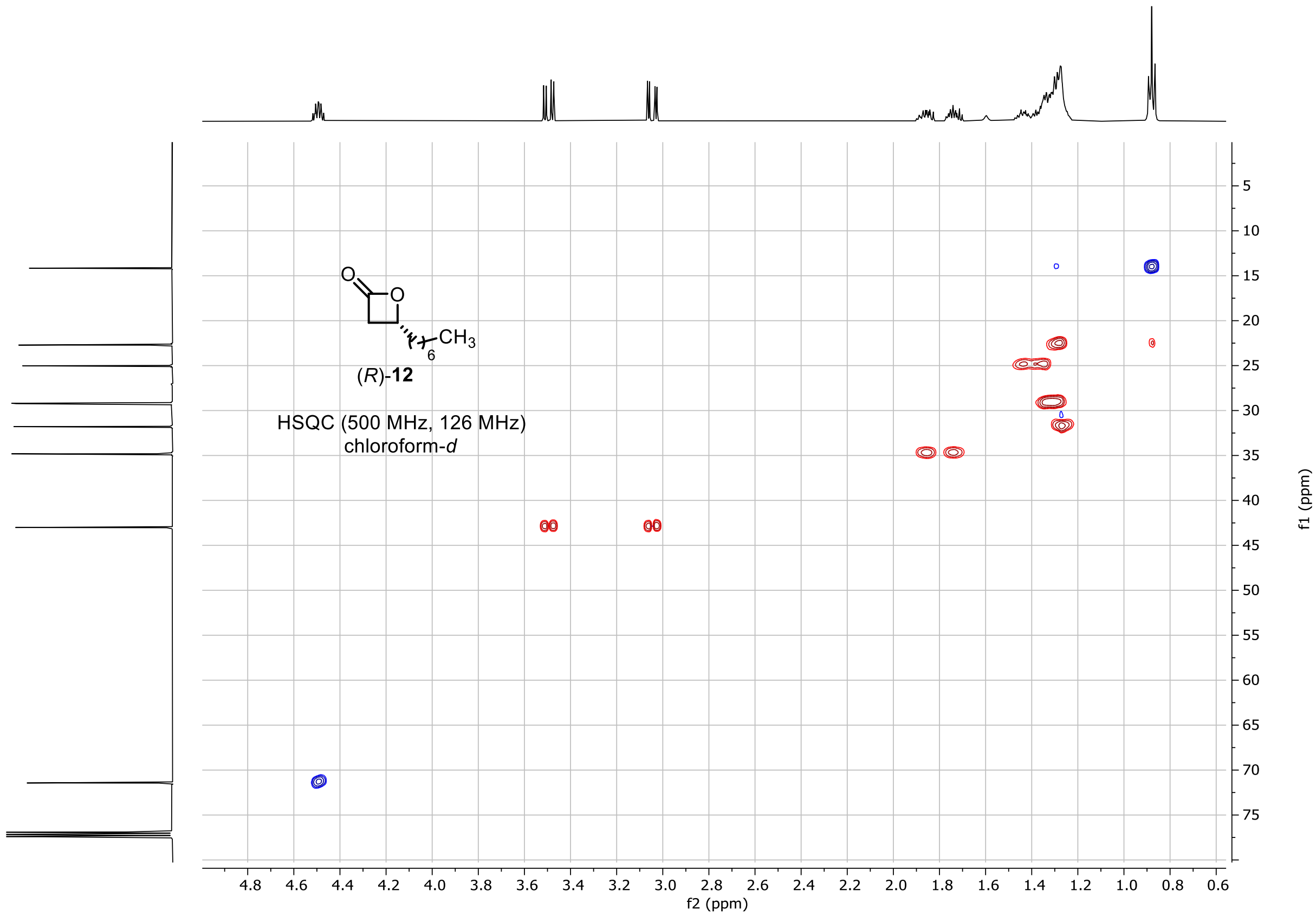

(R)-12
HSQC (500 MHz, 126 MHz)
chloroform-d
f2 (ppm)
f1 (ppm)

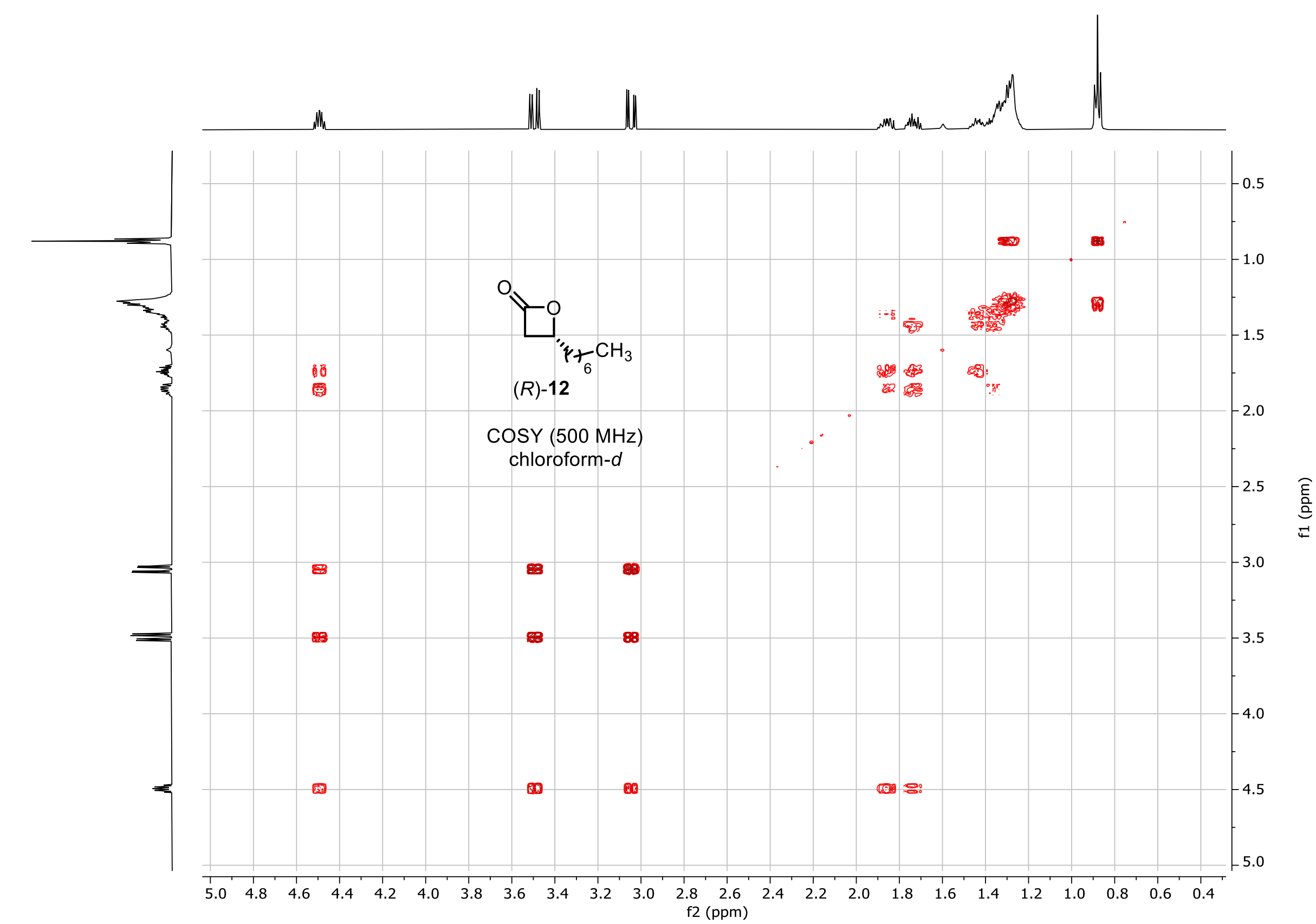

(R)-12
COSY (500 MHz)
chloroform-d
f2 (ppm)
f1 (ppm)

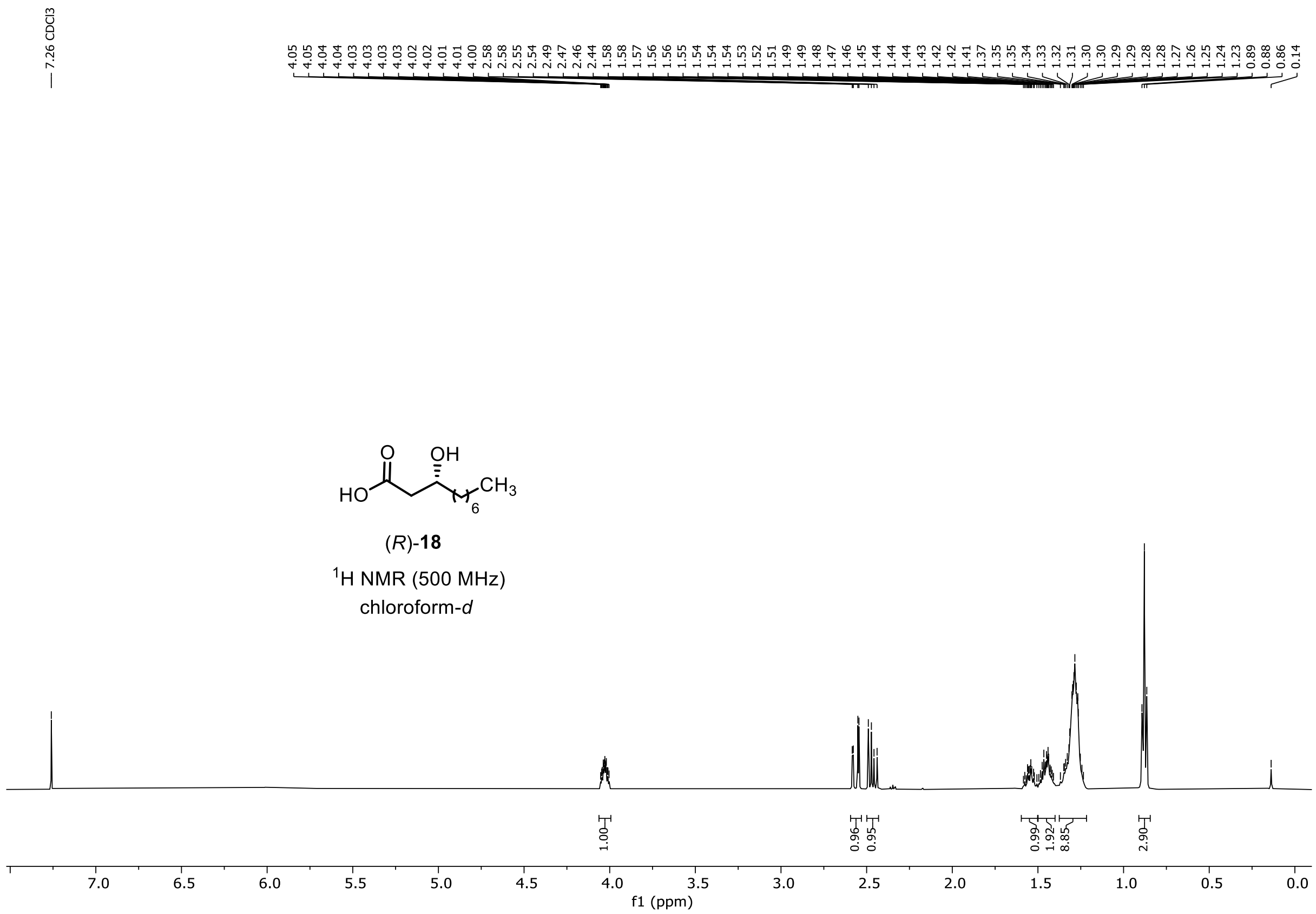
7.26 CDCl3
4.05
4.05
4.04
4.03
4.03
4.03
4.03
4.02
4.02
4.01
4.01
4.00
2.58
2.58
2.55
2.54
2.49
2.47
2.46
2.44
1.58
1.58
1.57
1.56
1.56
1.55
1.54
1.54
1.54
1.54
1.53
1.52
1.51
1.49
1.49
1.48
1.47
1.46
1.45
1.44
1.44
1.43
1.42
1.42
1.41
1.37
1.35
1.35
1.34
1.33
1.32
1.31
1.30
1.30
1.29
1.29
1.28
1.28
1.27
1.26
1.25
1.24
1.23
0.89
0.88
0.86
0.14
HO
O
OH
CH3
6
(R)-18
1H NMR (500 MHz)
chloroform-d
1.00
0.96
0.95
0.99
1.92
8.85
2.90
7.0
6.5
6.0
5.5
5.0
4.5
4.0
3.5
3.0
2.5
2.0
1.5
1.0
0.5
0.0
f1 (ppm)

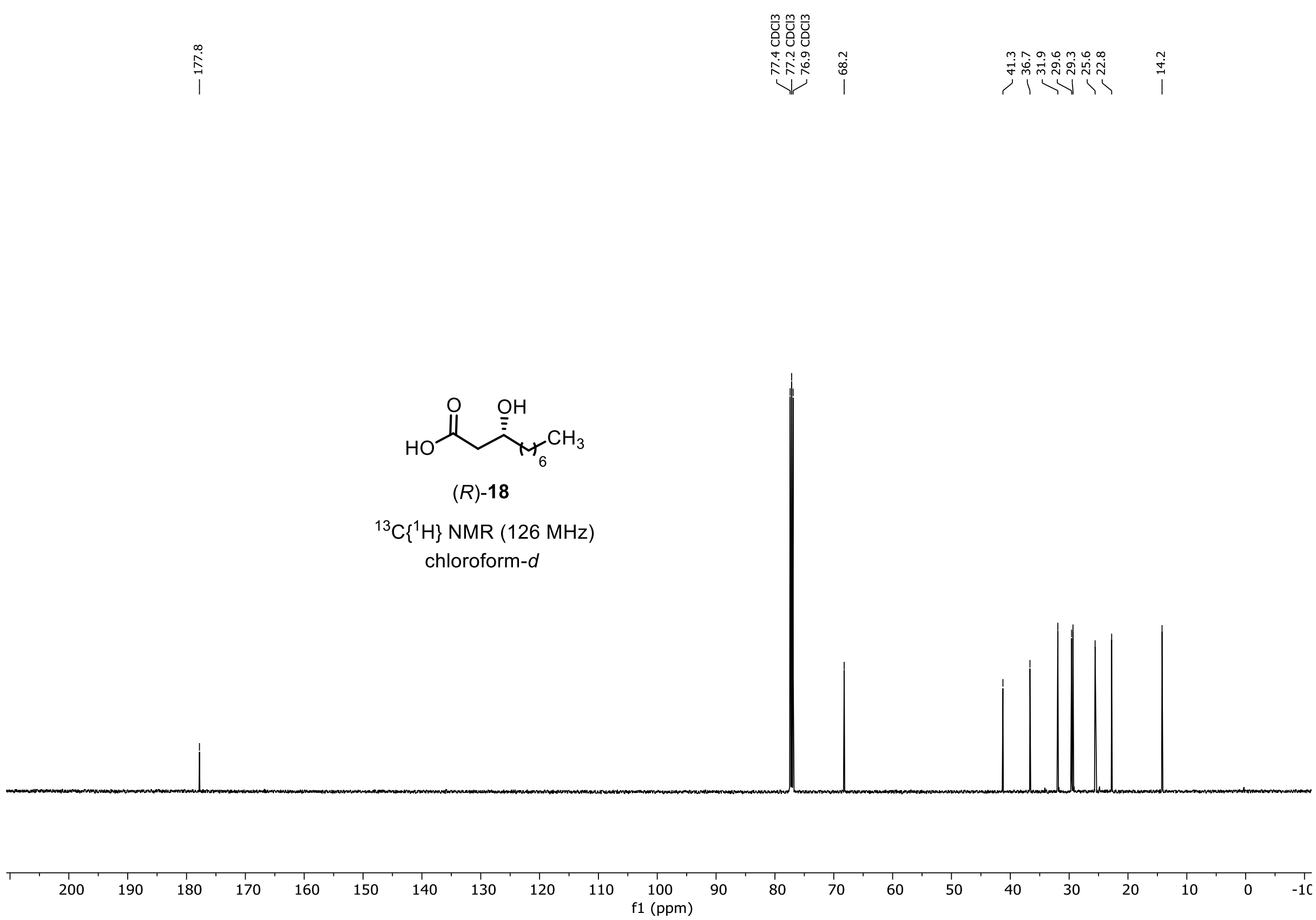
177.8
77.4 CDCl3
77.2 CDCl3
76.9 CDCl3
68.2
41.3
36.7
31.9
29.6
29.3
25.6
22.8
14.2
HO
O
OH
CH3
6
(R)-18
13C{1H} NMR (126 MHz)
chloroform-d
200
190
180
170
160
150
140
130
120
110
100
90
80
70
60
50
40
30
20
10
0
-10
f1 (ppm)

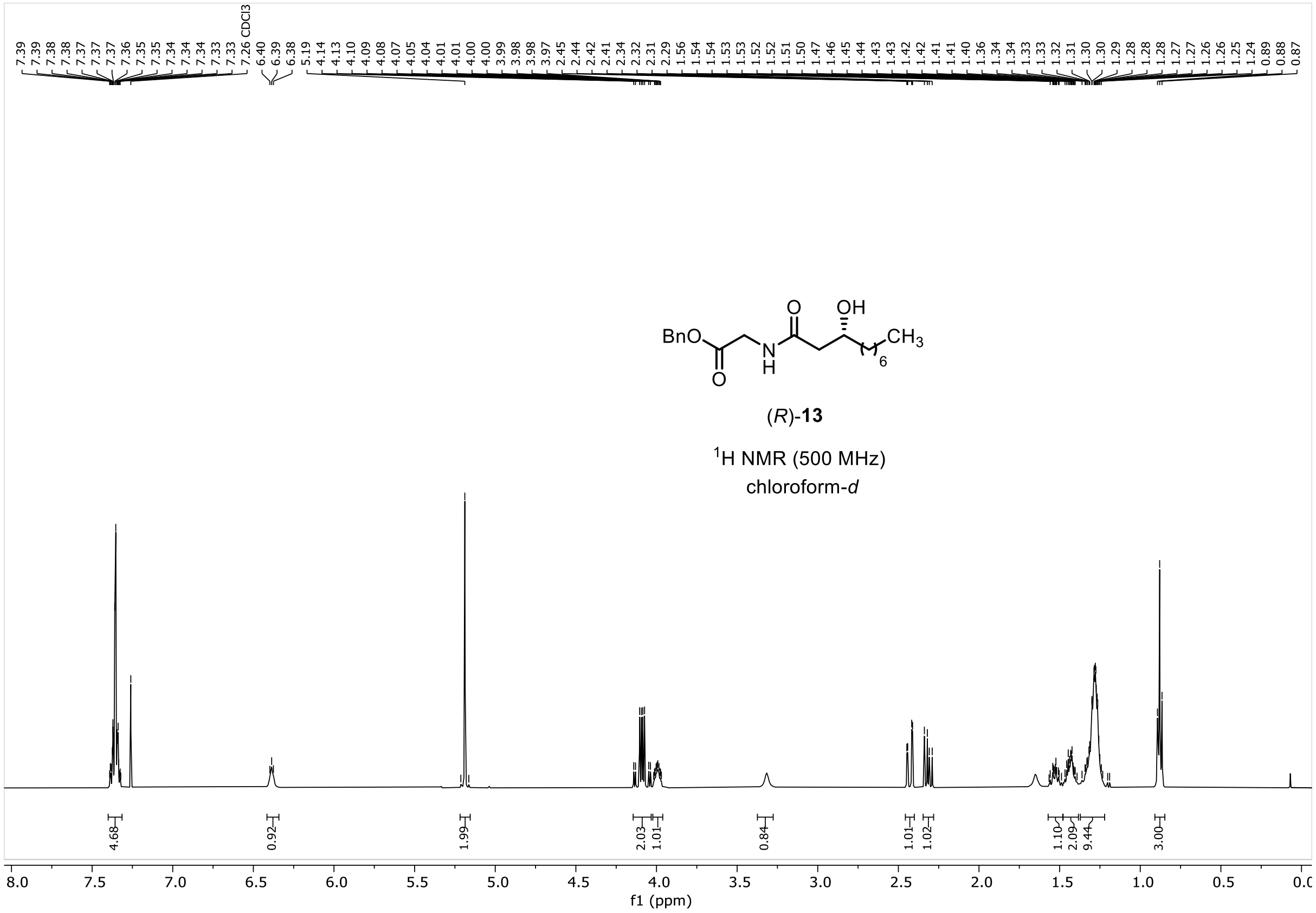
BnO
(R)-13
1H NMR (500 MHz)
chloroform-d
f1 (ppm)

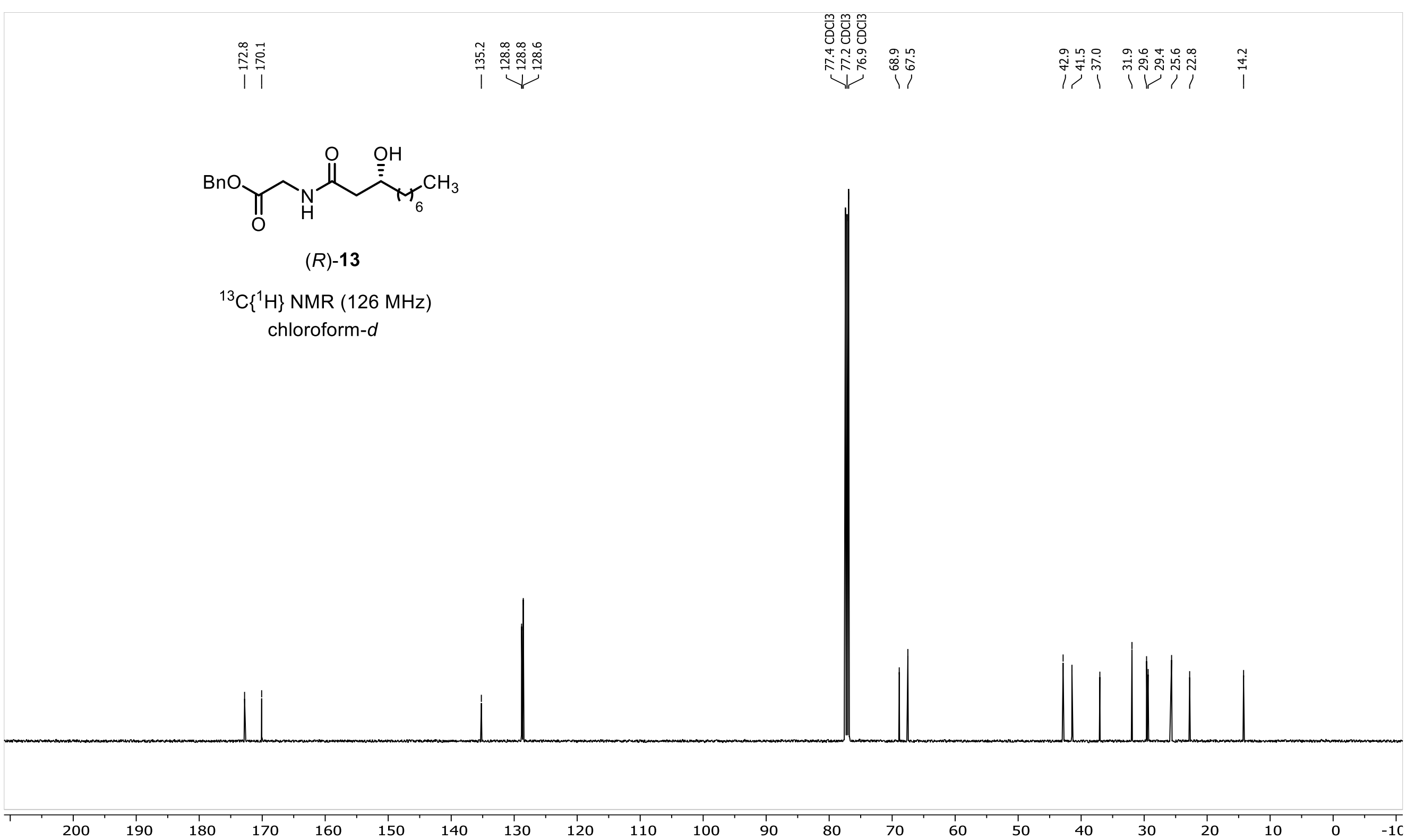
172.8
170.1
135.2
128.8
128.8
128.6
77.4 CDCl3
77.2 CDCl3
76.9 CDCl3
68.9
67.5
42.9
41.5
37.0
31.9
29.6
29.4
25.6
22.8
14.2
BnO
(R)-13
13C{1H} NMR (126 MHz)
chloroform-d
f1 (ppm)

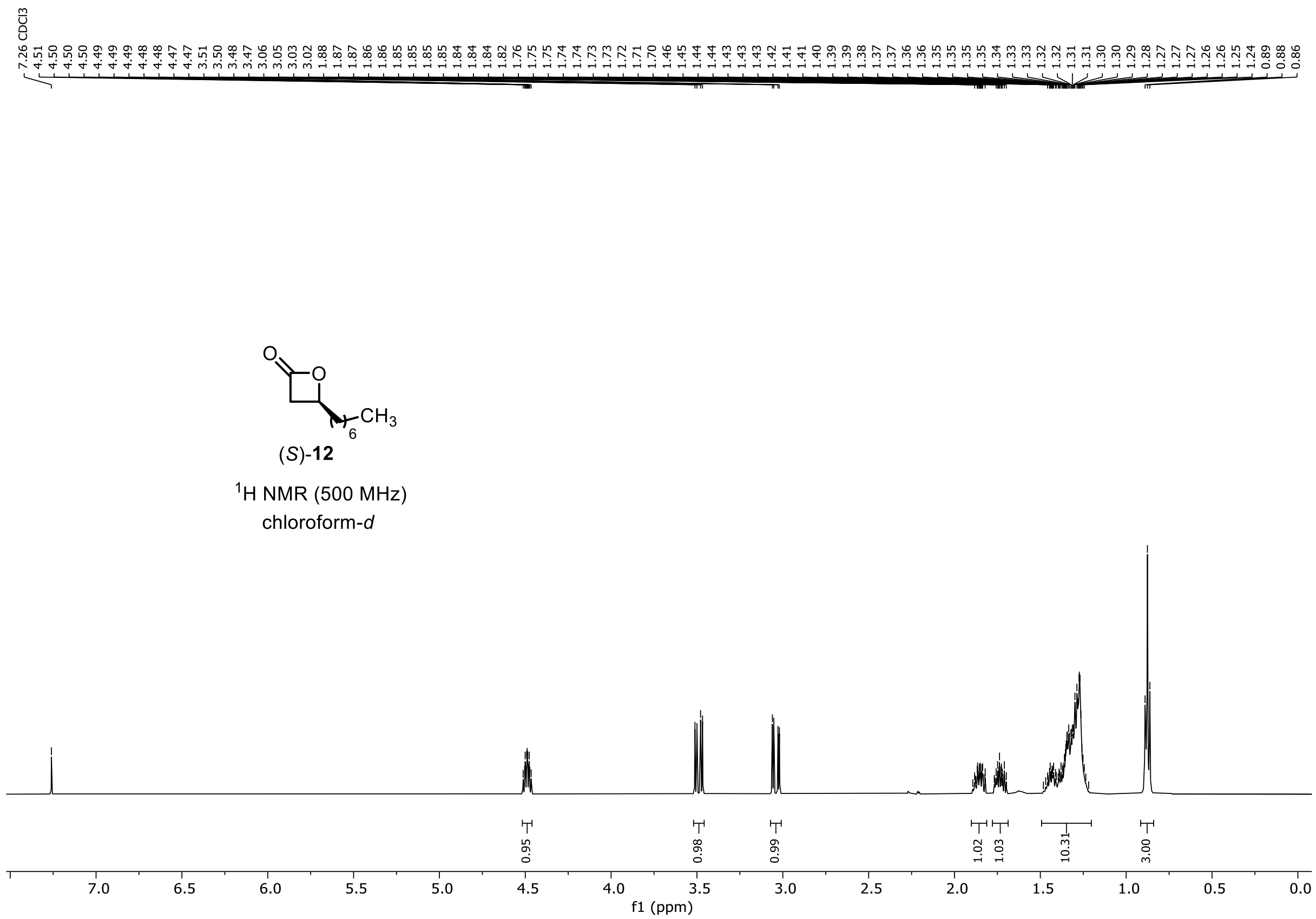
7.26 CDCl3
4.51 4.50 4.50 4.49 4.49 4.49 4.48 4.48 4.47 4.47 3.51 3.50 3.48 3.47 3.06 3.05 3.03 3.02 1.88 1.87 1.87 1.86 1.85 1.85 1.85 1.85 1.84 1.84 1.84 1.82 1.76 1.75 1.75 1.74 1.74 1.73 1.73 1.72 1.71 1.70 1.46 1.45 1.44 1.44 1.44 1.43 1.43 1.42 1.41 1.41 1.40 1.39 1.39 1.38 1.37 1.37 1.36 1.36 1.35 1.35 1.35 1.35 1.34 1.33 1.33 1.32 1.32 1.31 1.31 1.30 1.29 1.28 1.27 1.27 1.27 1.26 1.26 1.25 1.24 0.89 0.88 0.86
O
O
CH3
6
(S)-12
1H NMR (500 MHz)
chloroform-d
0.95
0.98
0.99
1.02
1.03
10.31
3.00
7.0 6.5 6.0 5.5 5.0 4.5 4.0 3.5 3.0 2.5 2.0 1.5 1.0 0.5 0.0
f1 (ppm)

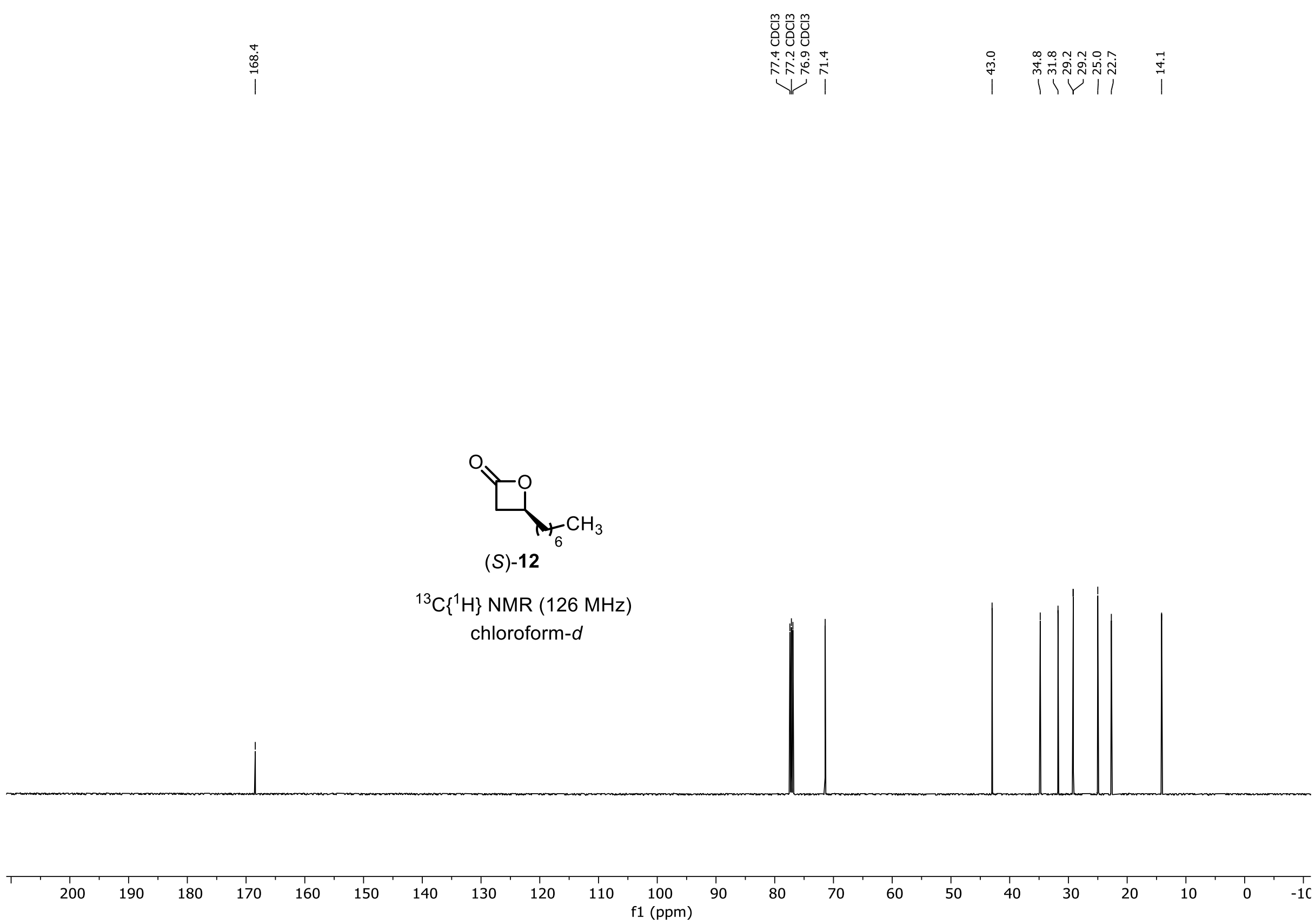
168.4
77.4 CDCl3
77.2 CDCl3
76.9 CDCl3
71.4
43.0
34.8
31.8
29.2
29.2
25.0
22.7
14.1
O
O
CH3
6
(S)-12
13C{1H} NMR (126 MHz)
chloroform-d
200 190 180 170 160 150 140 130 120 110 100 90 80 70 60 50 40 30 20 10 0 -10
f1 (ppm)

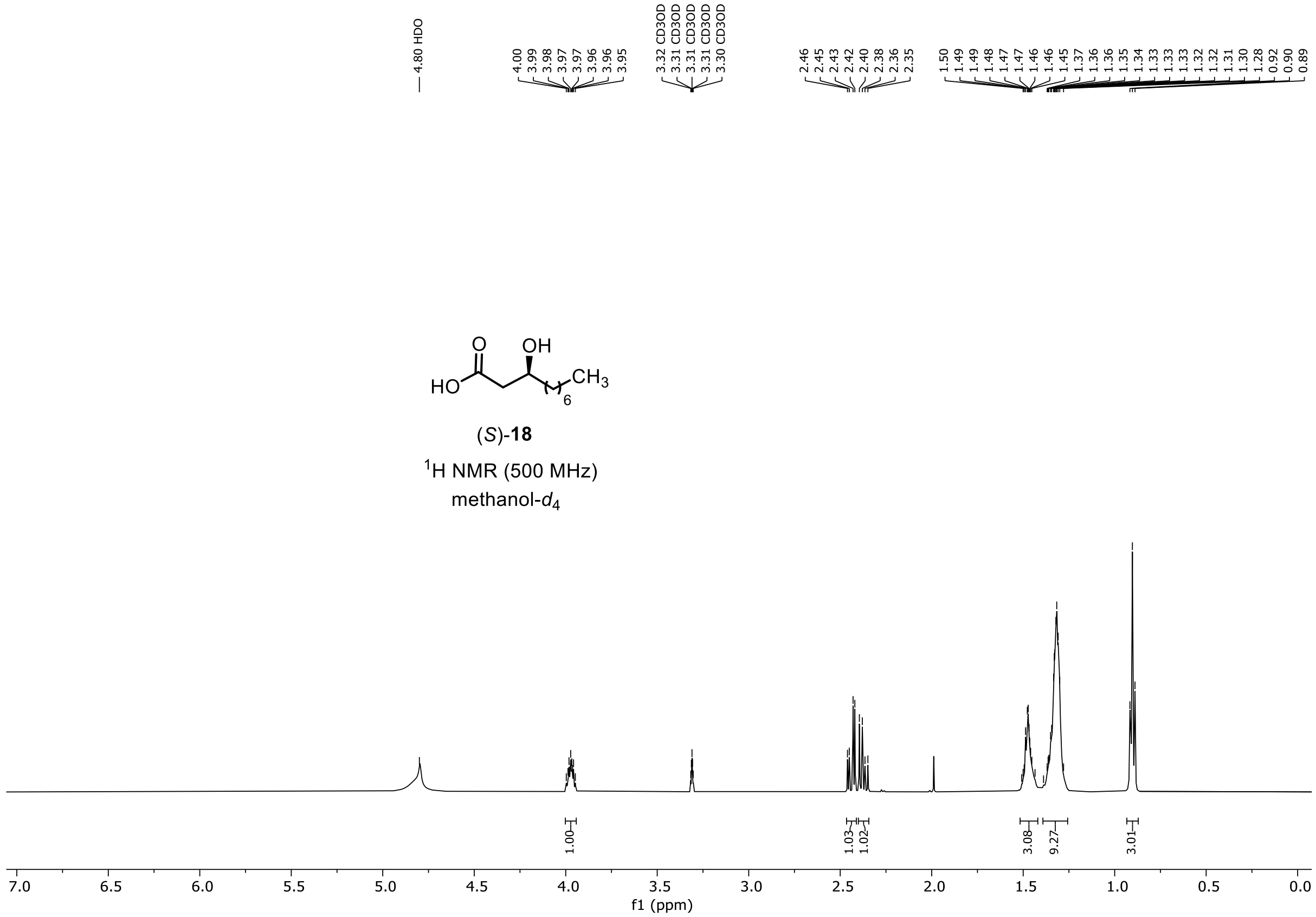
4.80 HDO
4.00 3.99 3.98 3.97 3.97 3.96 3.96 3.95
3.32 CD3OD 3.31 CD3OD 3.31 CD3OD 3.31 CD3OD 3.30 CD3OD
2.46 2.45 2.43 2.42 2.40 2.38 2.36 2.35
1.50 1.49 1.49 1.48 1.47 1.47 1.46 1.46 1.45 1.37 1.36 1.36 1.35 1.34 1.33 1.33 1.33 1.32 1.32 1.31 1.30 1.28 0.92 0.90 0.89
O
OH
HO
CH3
6
(S)-18
1H NMR (500 MHz)
methanol-d4
1.00
1.03
1.02
3.08
9.27
3.01
7.0 6.5 6.0 5.5 5.0 4.5 4.0 3.5 3.0 2.5 2.0 1.5 1.0 0.5 0.0
f1 (ppm)

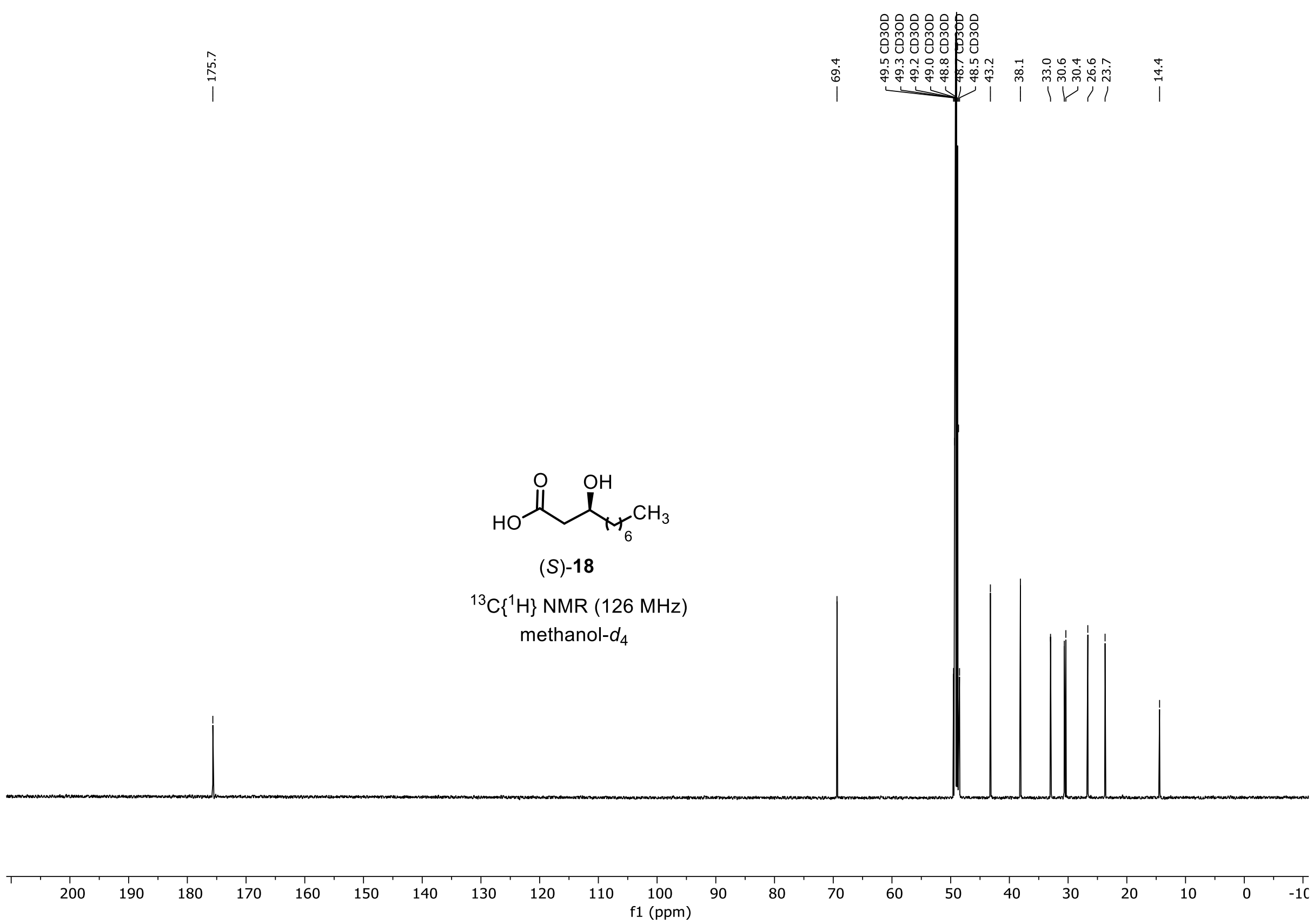
175.7
69.4
49.5 CD3OD 49.3 CD3OD 49.2 CD3OD 49.0 CD3OD 48.8 CD3OD 48.7 CD3OD 48.5 CD3OD
43.2
38.1
33.0
30.6
30.4
26.6
23.7
14.4
O
OH
HO
CH3
6
(S)-18
13C{1H} NMR (126 MHz)
methanol-d4
200 190 180 170 160 150 140 130 120 110 100 90 80 70 60 50 40 30 20 10 0 -10
f1 (ppm)

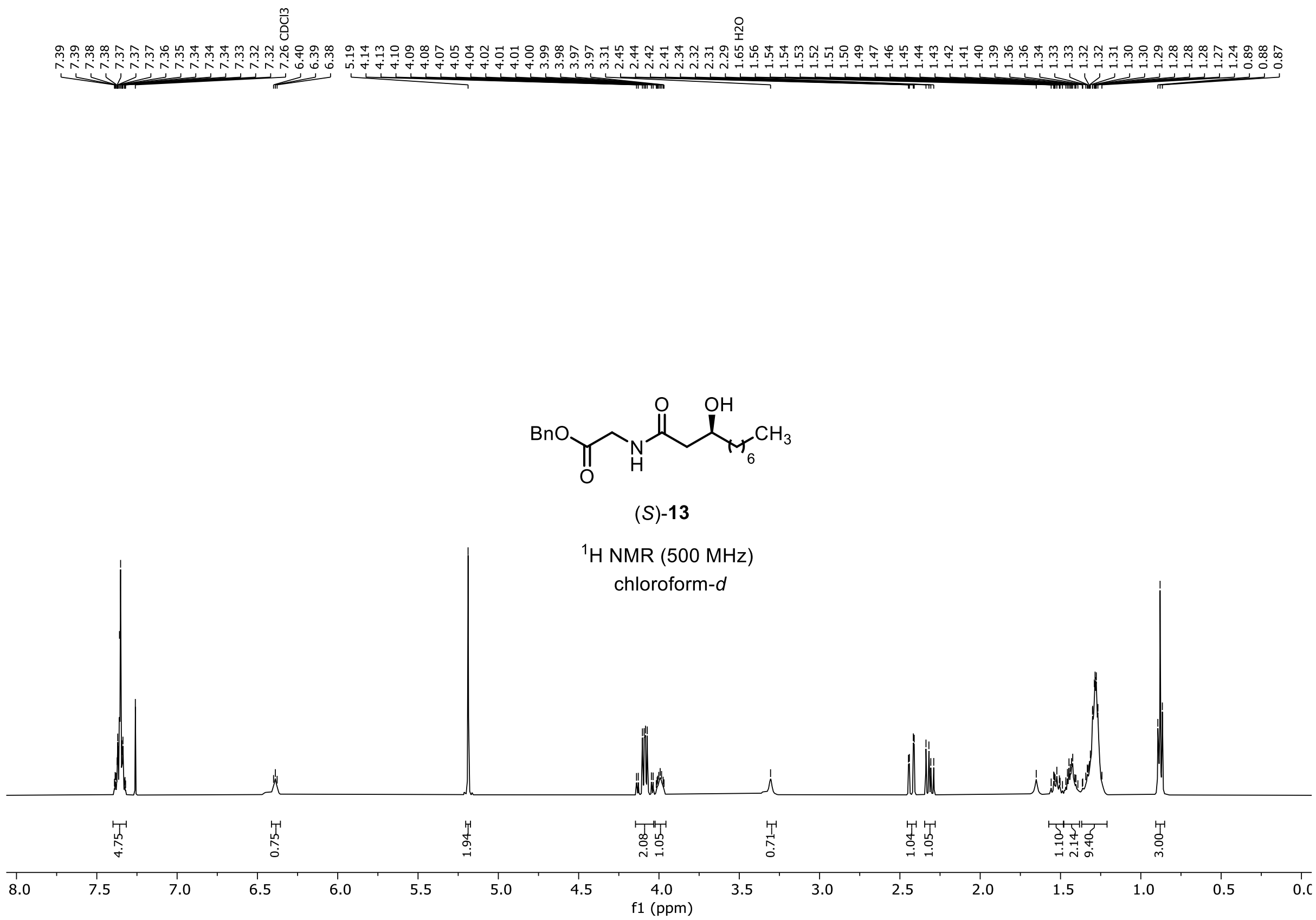

BnO
N
H
O
O
OH
CH3
6
(S)-13
1H NMR (500 MHz)
chloroform-d
f1 (ppm)

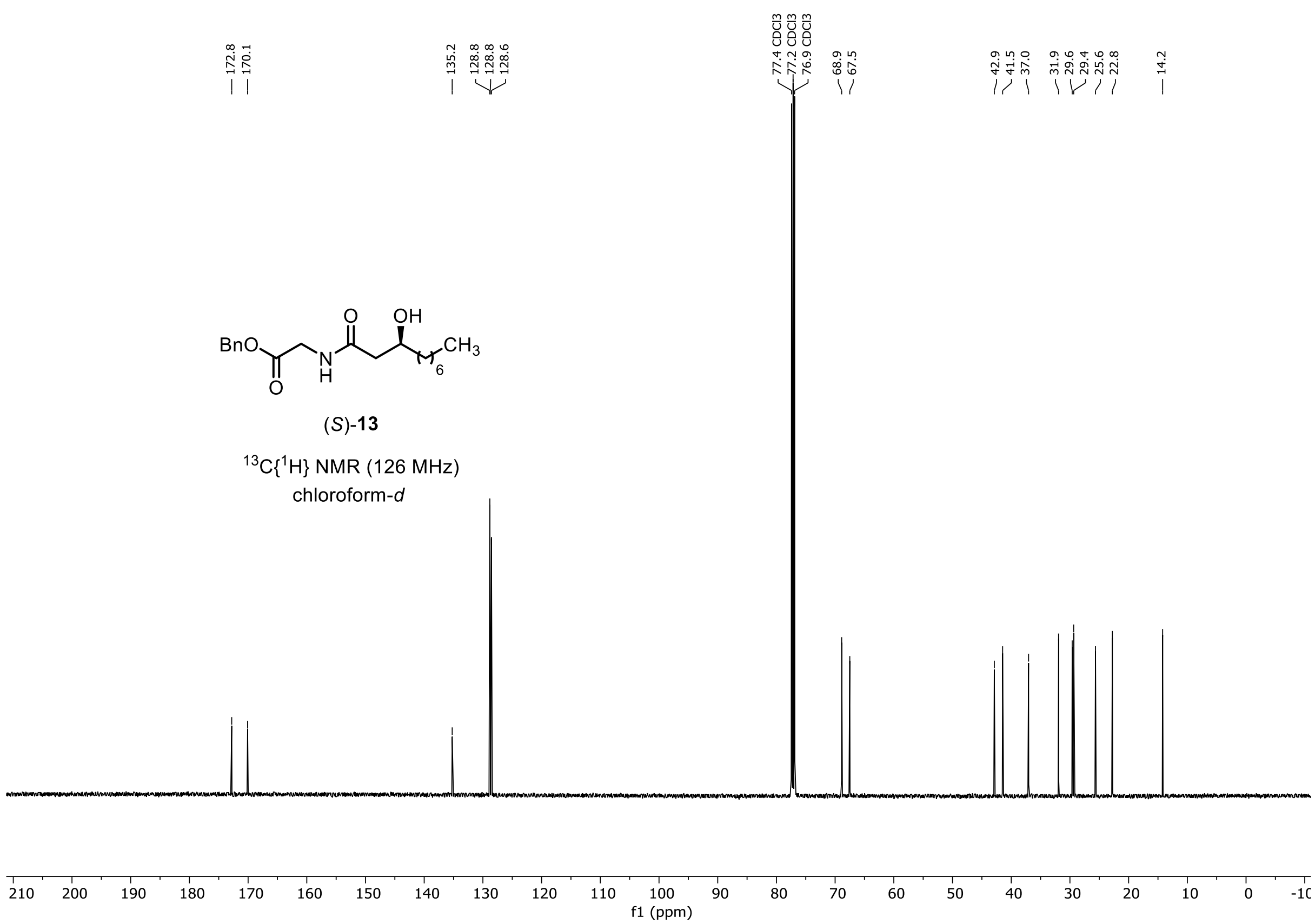

172.8
170.1
135.2
128.8
128.8
128.6
77.4 CDCl3
77.2 CDCl3
76.9 CDCl3
68.9
67.5
42.9
41.5
37.0
31.9
29.6
29.4
25.6
22.8
14.2
BnO
N
H
O
O
OH
CH3
6
(S)-13
13C{1H} NMR (126 MHz)
chloroform-d
f1 (ppm)

NMR SPECTRA OF ACYBOLIN F

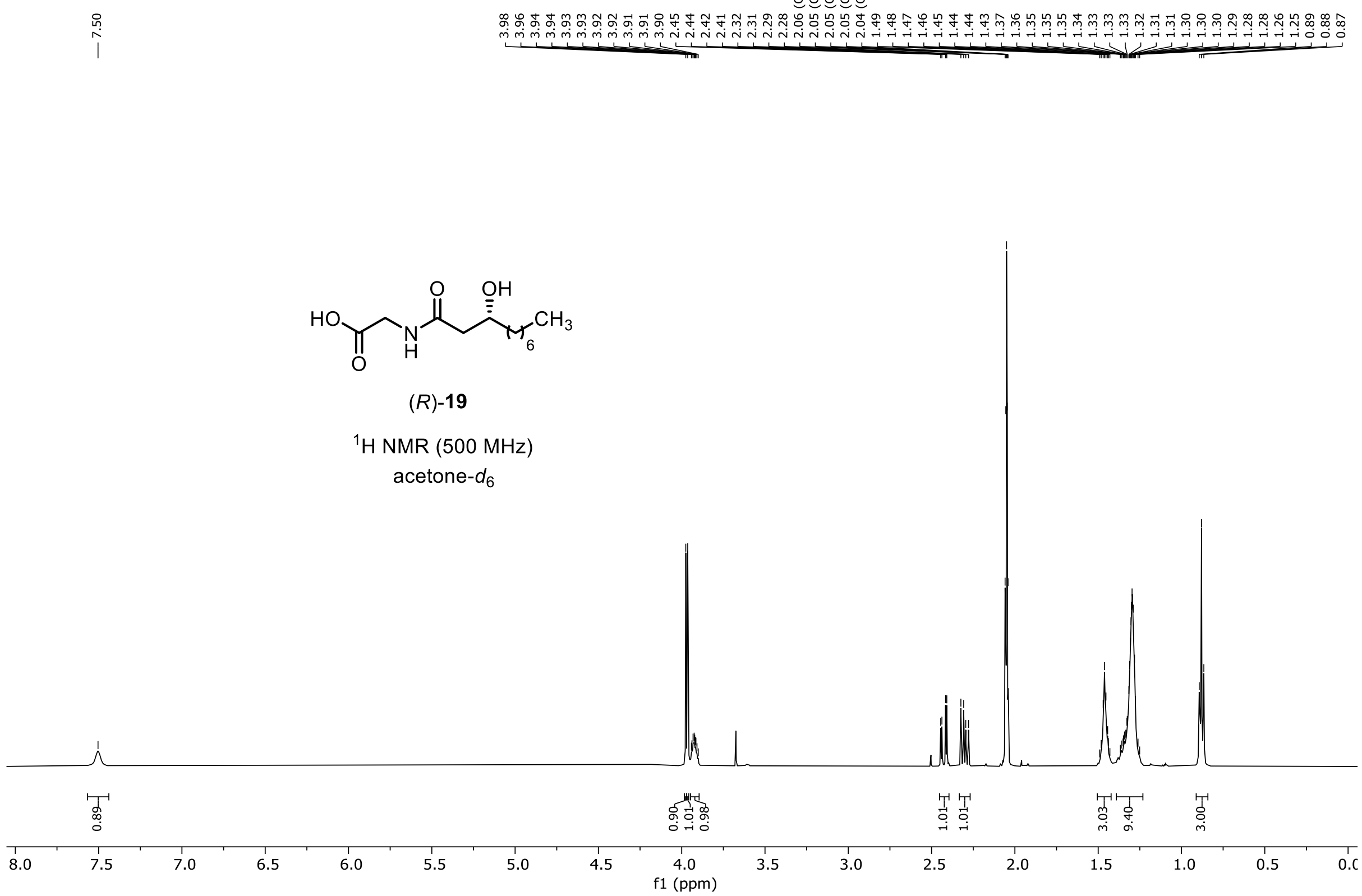
(R)-19
1H NMR (500 MHz)
acetone-d6
f1 (ppm)

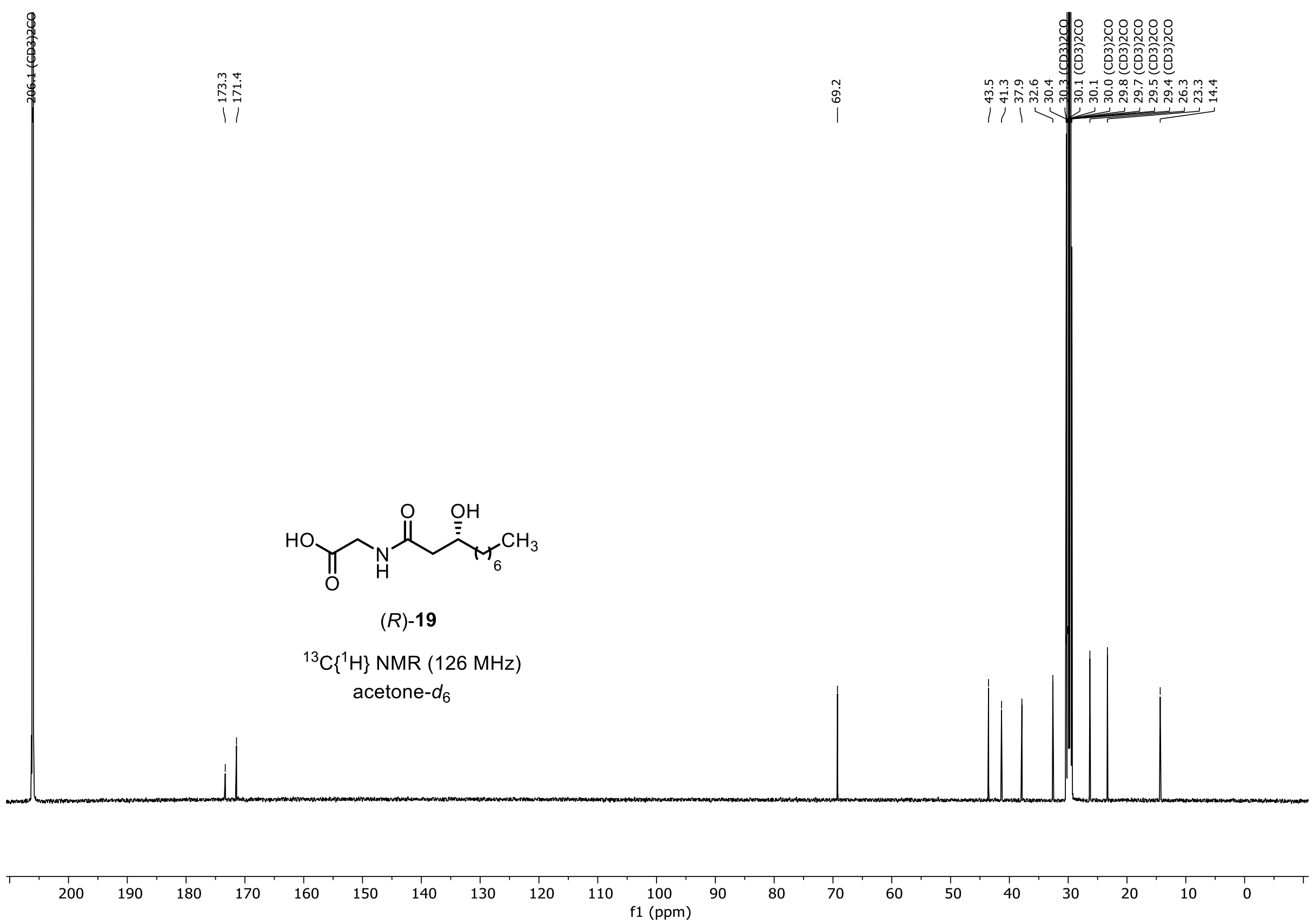
(R)-19
13C{1H} NMR (126 MHz)
acetone-d6
f1 (ppm)

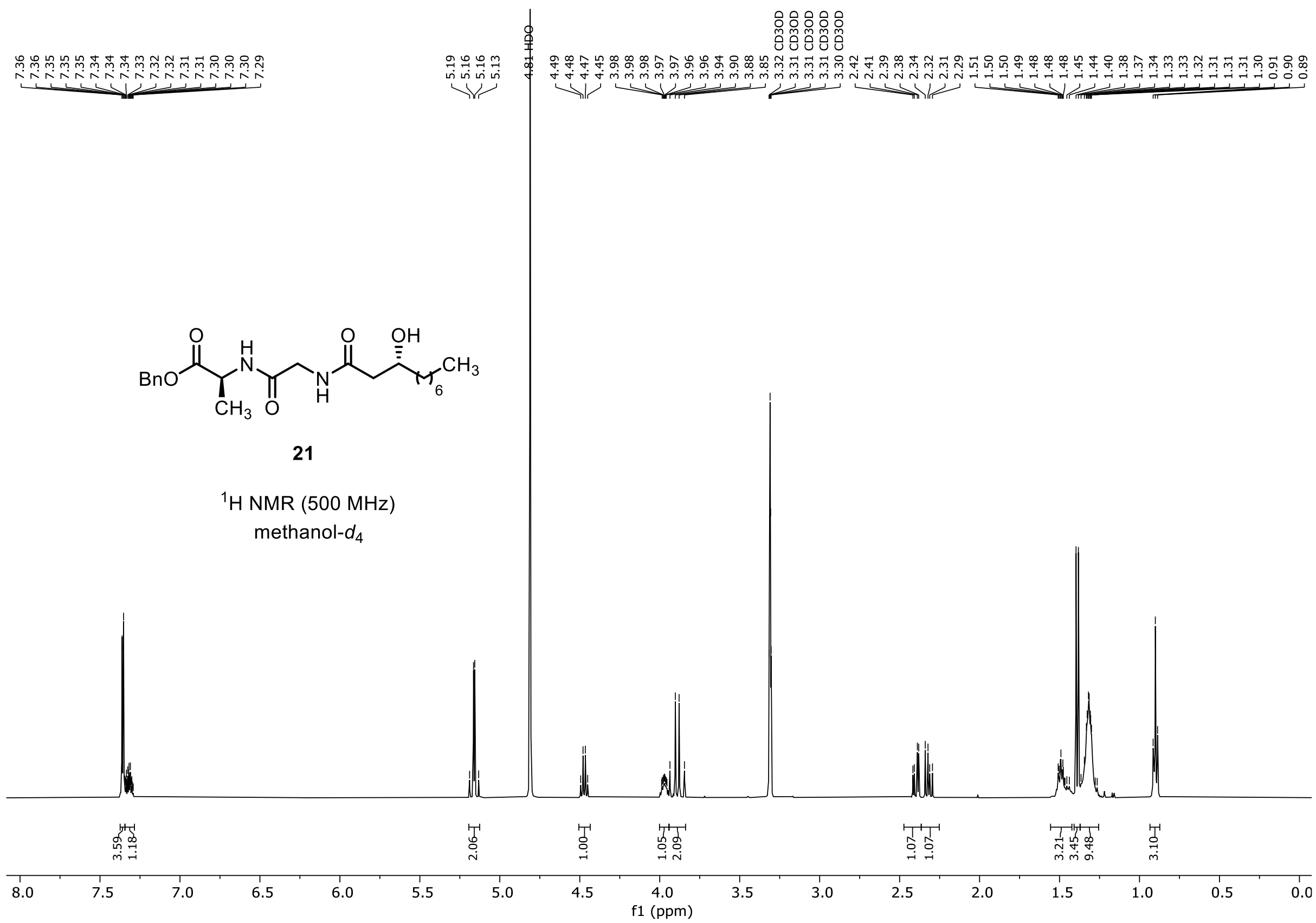
7.36 7.36 7.35 7.35 7.35 7.34 7.34 7.34 7.33 7.32 7.32 7.31 7.31 7.30 7.30 7.30 7.29
5.19 5.16 5.16 5.13
4.81 HDO
4.49 4.48 4.47 4.45
3.98 3.98 3.98 3.97 3.97 3.96 3.96 3.94 3.90 3.88 3.85
3.32 CD3OD 3.31 CD3OD 3.31 CD3OD 3.31 CD3OD 3.30 CD3OD
2.42 2.41 2.39 2.38 2.34 2.32 2.31 2.29
1.51 1.50 1.50 1.49 1.48 1.48 1.48 1.48 1.45 1.44 1.40 1.38 1.37 1.34 1.33 1.33 1.32 1.31 1.31 1.31 1.30 0.91 0.90 0.89
BnO
H
N
O
OH
CH3
6
21
1H NMR (500 MHz)
methanol-d4
3.59 1.18 2.06 1.00 1.05 2.09 1.07 1.07 3.21 3.45 9.48 3.10
8.0 7.5 7.0 6.5 6.0 5.5 5.0 4.5 4.0 3.5 3.0 2.5 2.0 1.5 1.0 0.5 0.0
f1 (ppm)

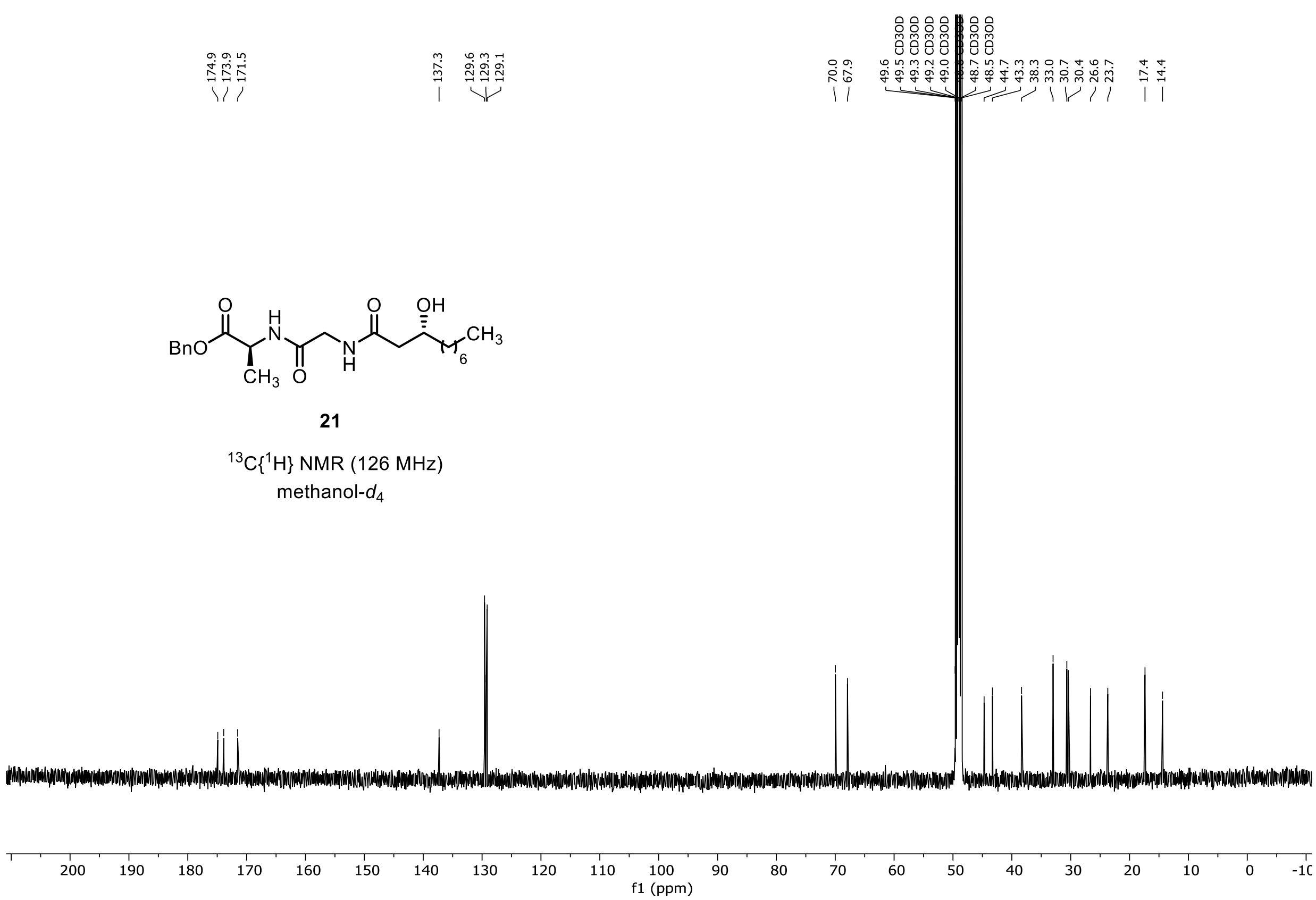
174.9 173.9 171.5
137.3
129.6 129.3 129.1
70.0 67.9
49.6 49.5 CD3OD 49.3 CD3OD 49.2 CD3OD 49.0 CD3OD 48.8 CD3OD 48.7 CD3OD 48.5 CD3OD
44.7 43.3 38.3 33.0 30.7 30.4 26.6 23.7 17.4 14.4
BnO
H
N
O
OH
CH3
6
21
13C{1H} NMR (126 MHz)
methanol-d4
200 190 180 170 160 150 140 130 120 110 100 90 80 70 60 50 40 30 20 10 0 -10
f1 (ppm)

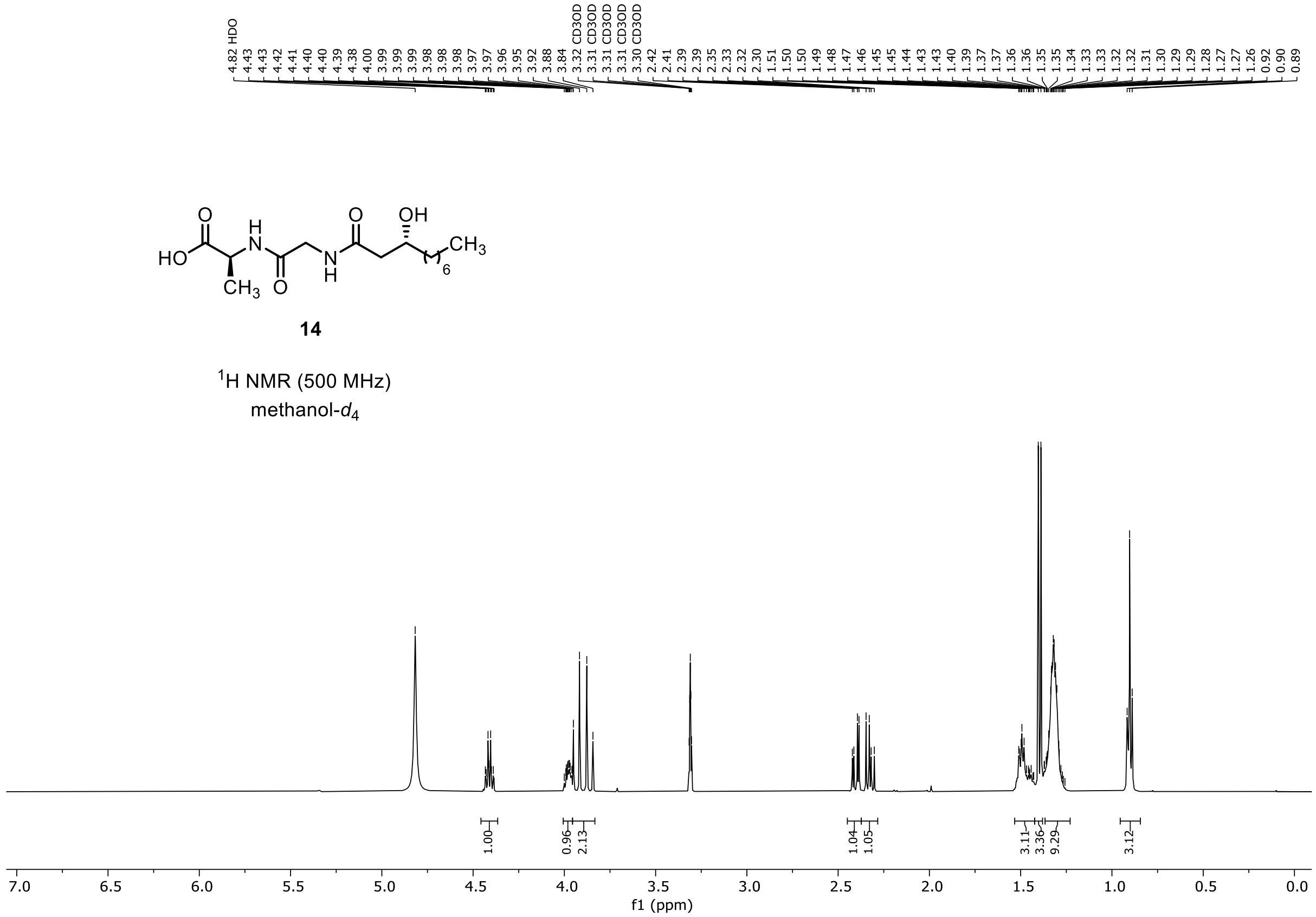

14

^{1}H NMR (500 MHz)
methanol-d_4

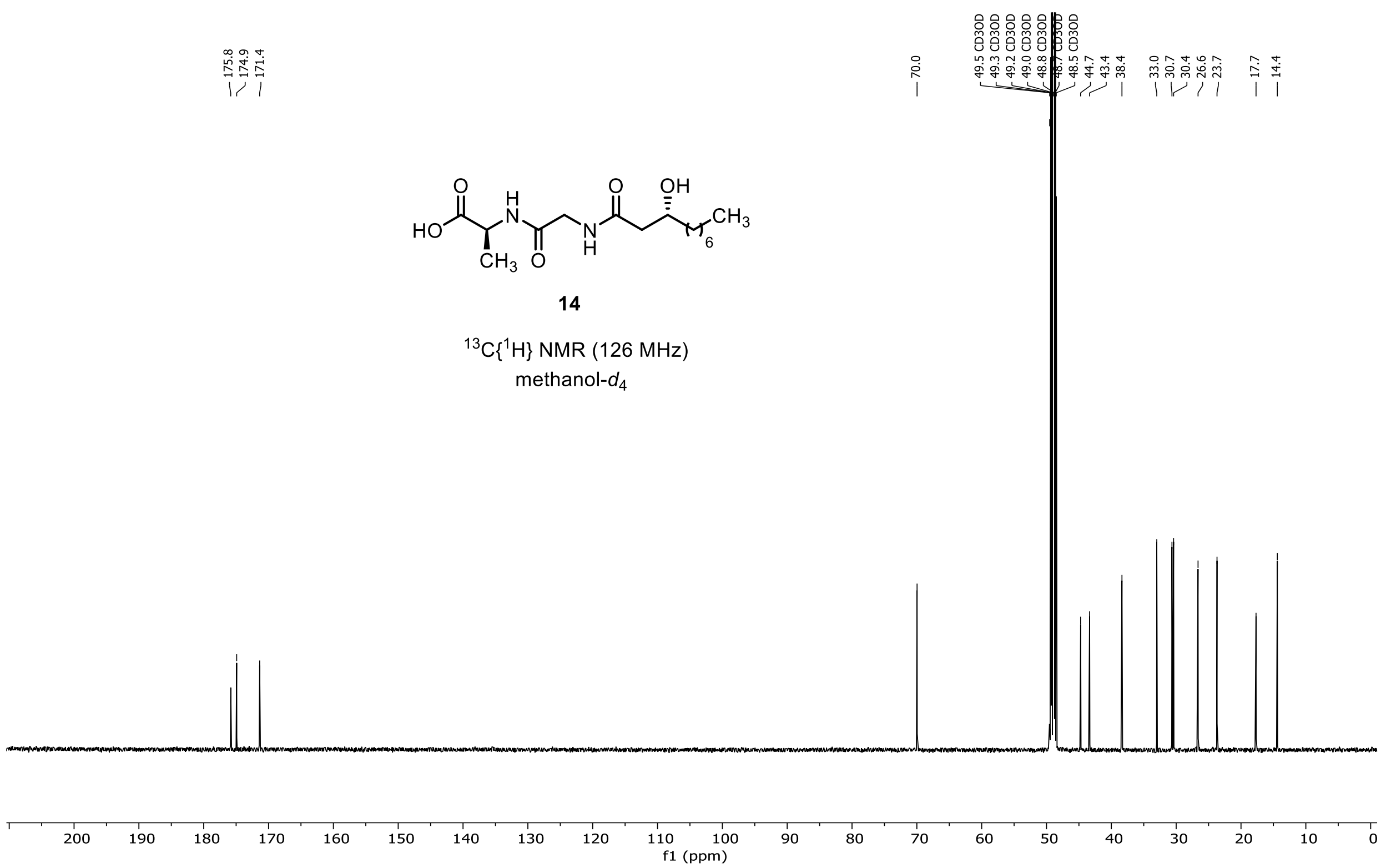

14

$^{13}C\{^{1}H\}$ NMR (126 MHz)
methanol-d_4

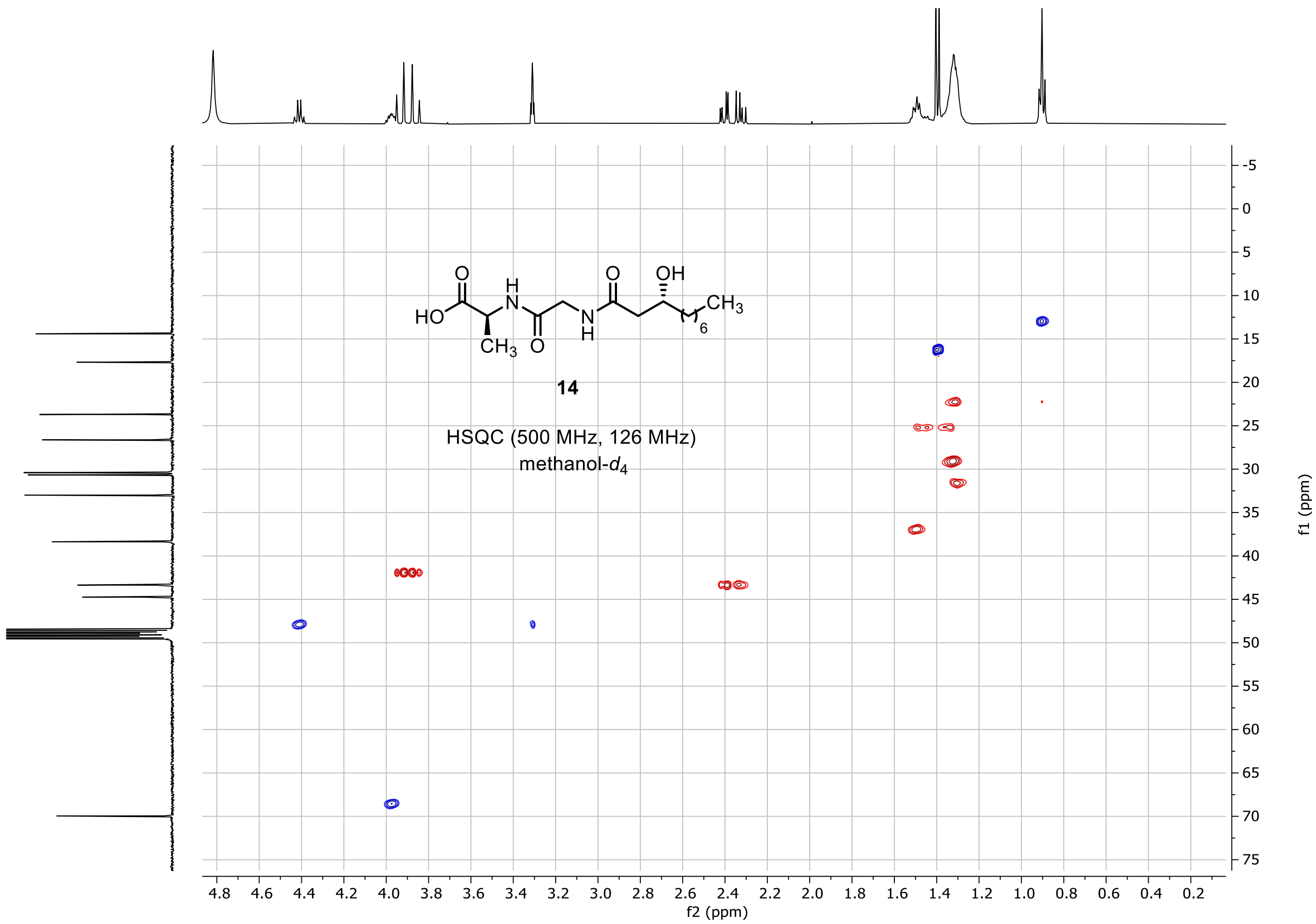
14
HSQC (500 MHz, 126 MHz)
methanol-d4
f2 (ppm)
f1 (ppm)

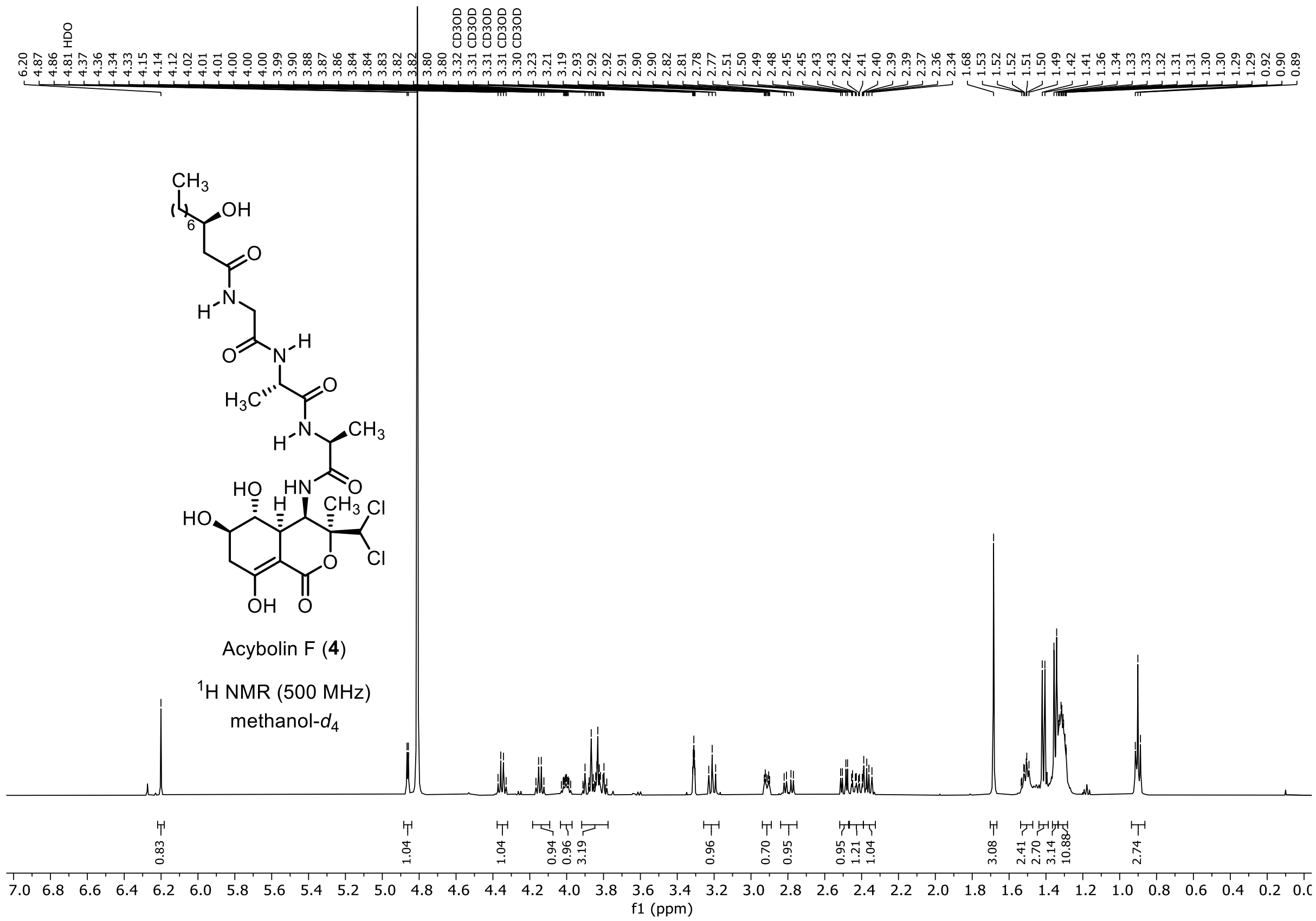
6.20 4.87 4.86 4.81 HDO 4.37 4.36 4.34 4.33 4.15 4.14 4.12 4.02 4.01 4.01 4.00 4.00 4.00 3.99 3.90 3.88 3.87 3.86 3.84 3.84 3.83 3.83 3.82 3.82 3.80 3.80 3.32 CD3OD 3.31 CD3OD 3.31 CD3OD 3.31 CD3OD 3.30 CD3OD 3.23 3.21 3.19 2.93 2.92 2.92 2.91 2.90 2.90 2.82 2.81 2.78 2.77 2.51 2.50 2.49 2.48 2.45 2.45 2.43 2.43 2.42 2.41 2.40 2.39 2.39 2.37 2.36 2.34 1.68 1.53 1.52 1.52 1.51 1.50 1.49 1.42 1.41 1.36 1.34 1.33 1.33 1.32 1.31 1.31 1.30 1.30 1.29 1.29 0.92 0.90 0.89
Acybolin F (**4**)
^{1}H NMR (500 MHz)
methanol-d_4
0.83 1.04 1.04 0.94 0.96 3.19 0.96 0.70 0.95 0.95 1.21 1.04 3.08 2.41 2.70 3.14 10.88 2.74
7.0 6.8 6.6 6.4 6.2 6.0 5.8 5.6 5.4 5.2 5.0 4.8 4.6 4.4 4.2 4.0 3.8 3.6 3.4 3.2 3.0 2.8 2.6 2.4 2.2 2.0 1.8 1.6 1.4 1.2 1.0 0.8 0.6 0.4 0.2 0.0
f1 (ppm)

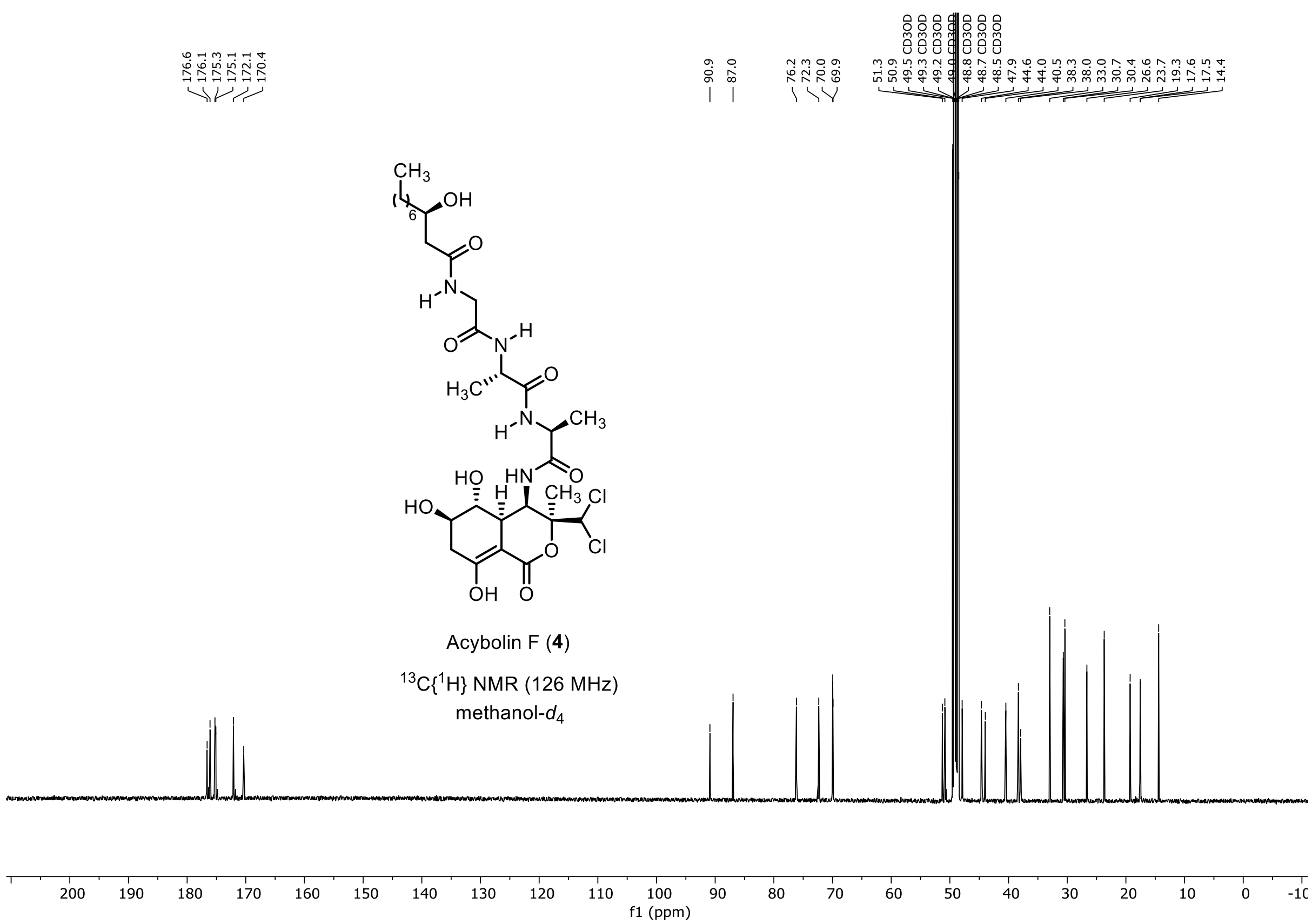
176.6 176.1 175.3 175.1 172.1 170.4 90.9 87.0 76.2 72.3 70.0 69.9 51.3 50.9 49.5 CD3OD 49.3 CD3OD 49.2 CD3OD 49.0 CD3OD 48.8 CD3OD 48.7 CD3OD 48.5 CD3OD 47.9 44.6 44.0 40.5 38.3 38.0 33.0 30.7 30.4 26.6 23.7 19.3 17.6 17.5 14.4
Acybolin F (**4**)
^{13}C{^{1}H} NMR (126 MHz)
methanol-d_4
200 190 180 170 160 150 140 130 120 110 100 90 80 70 60 50 40 30 20 10 0 -10
f1 (ppm)

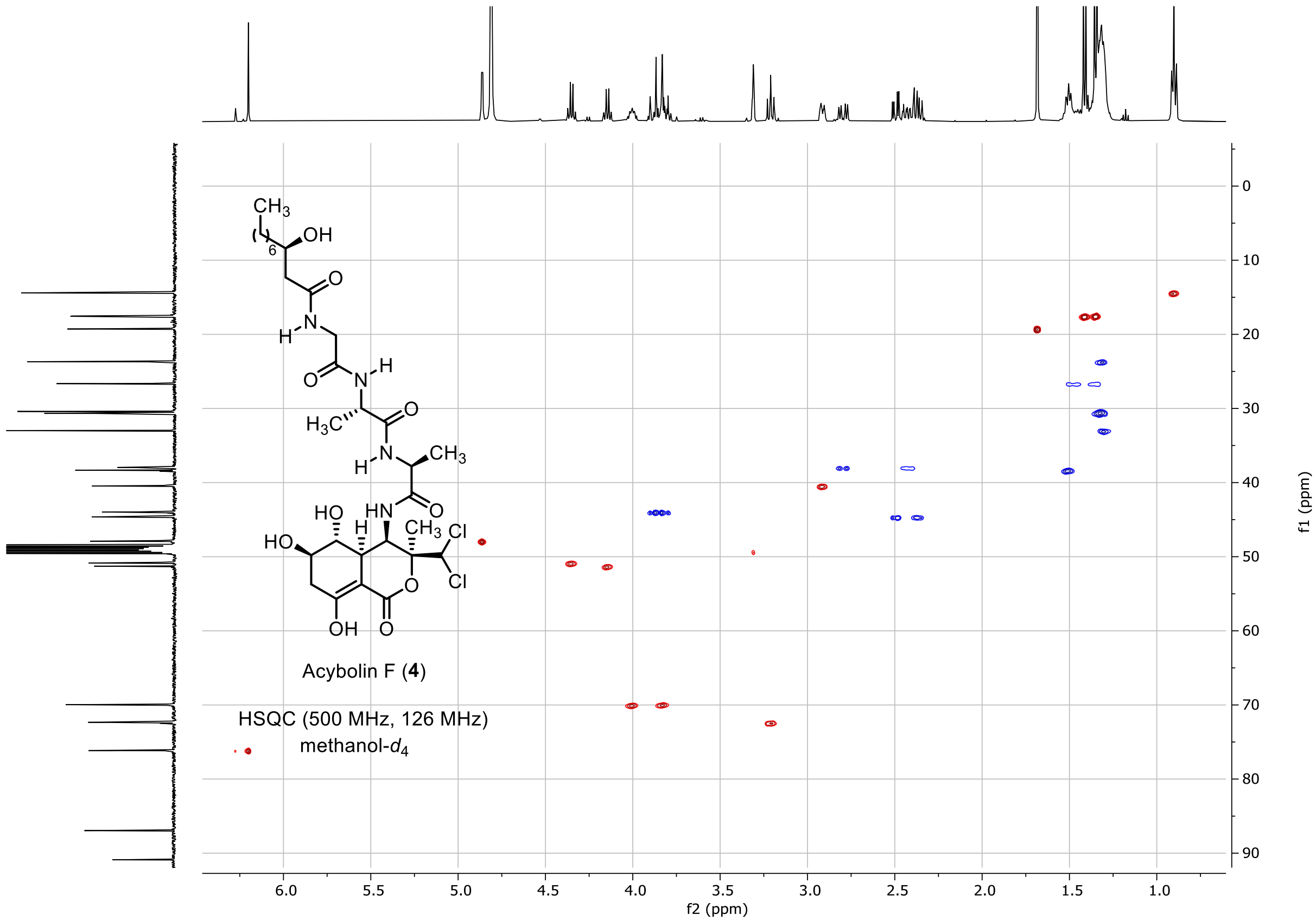

Acybolin F (4)
HSQC (500 MHz, 126 MHz)
methanol-d_4
f2 (ppm)
f1 (ppm)

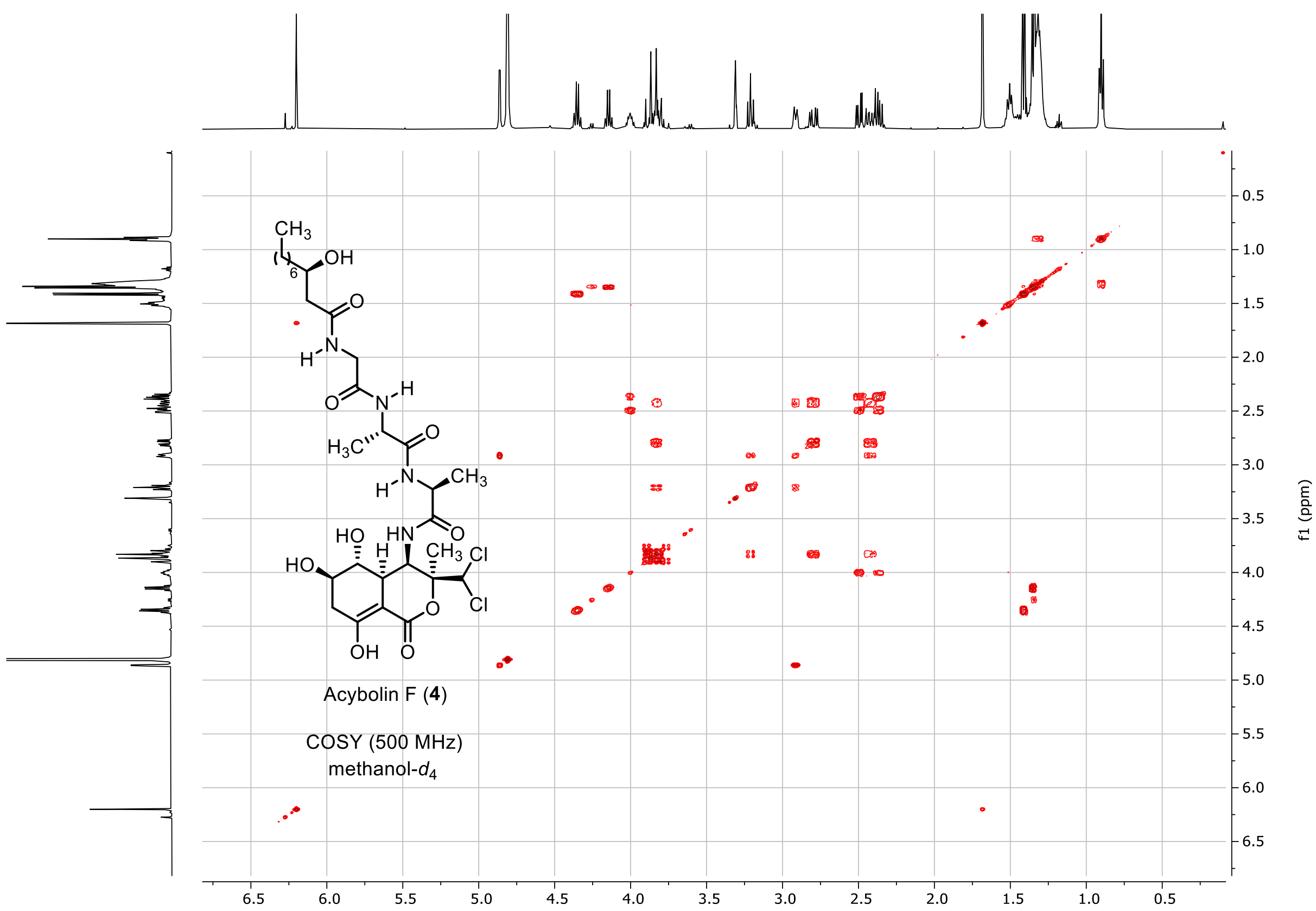

Acybolin F (4)
COSY (500 MHz)
methanol-d_4
f2 (ppm)
f1 (ppm)

NMR SPECTRA OF ACYBOLIN B

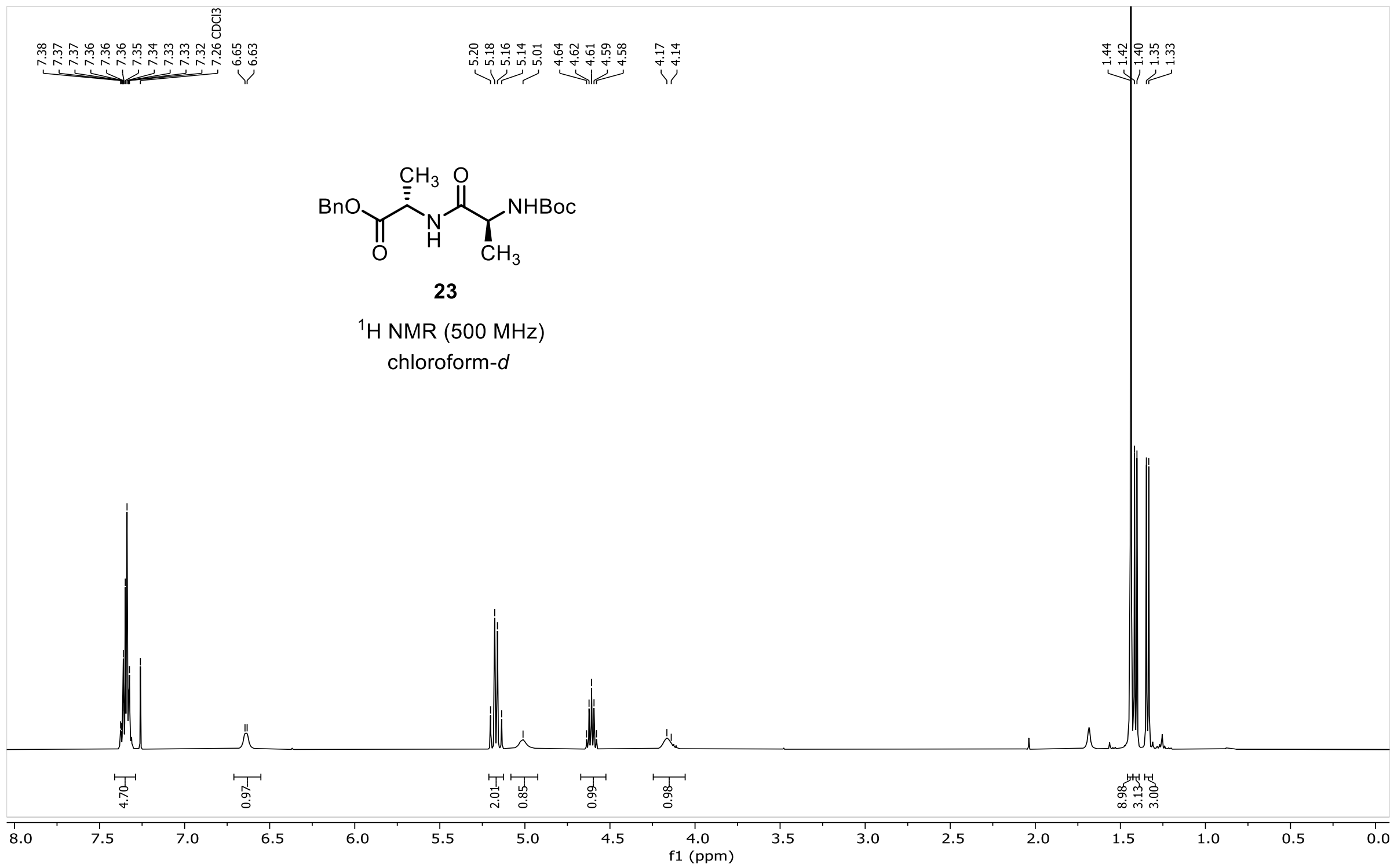

23
^{1}H NMR (500 MHz)
chloroform-d
f1 (ppm)

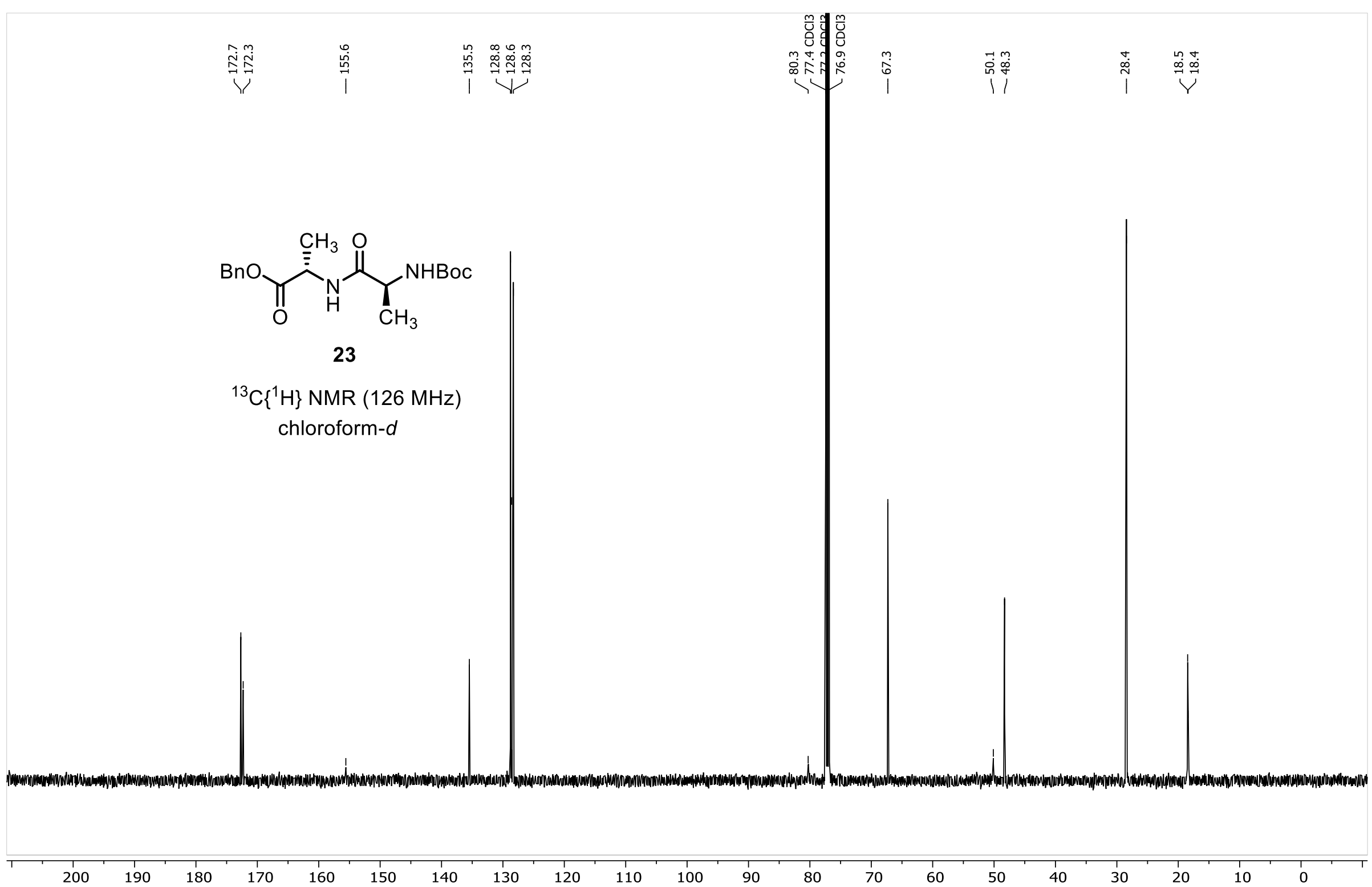

23
^{13}C{^{1}H} NMR (126 MHz)
chloroform-d
f1 (ppm)

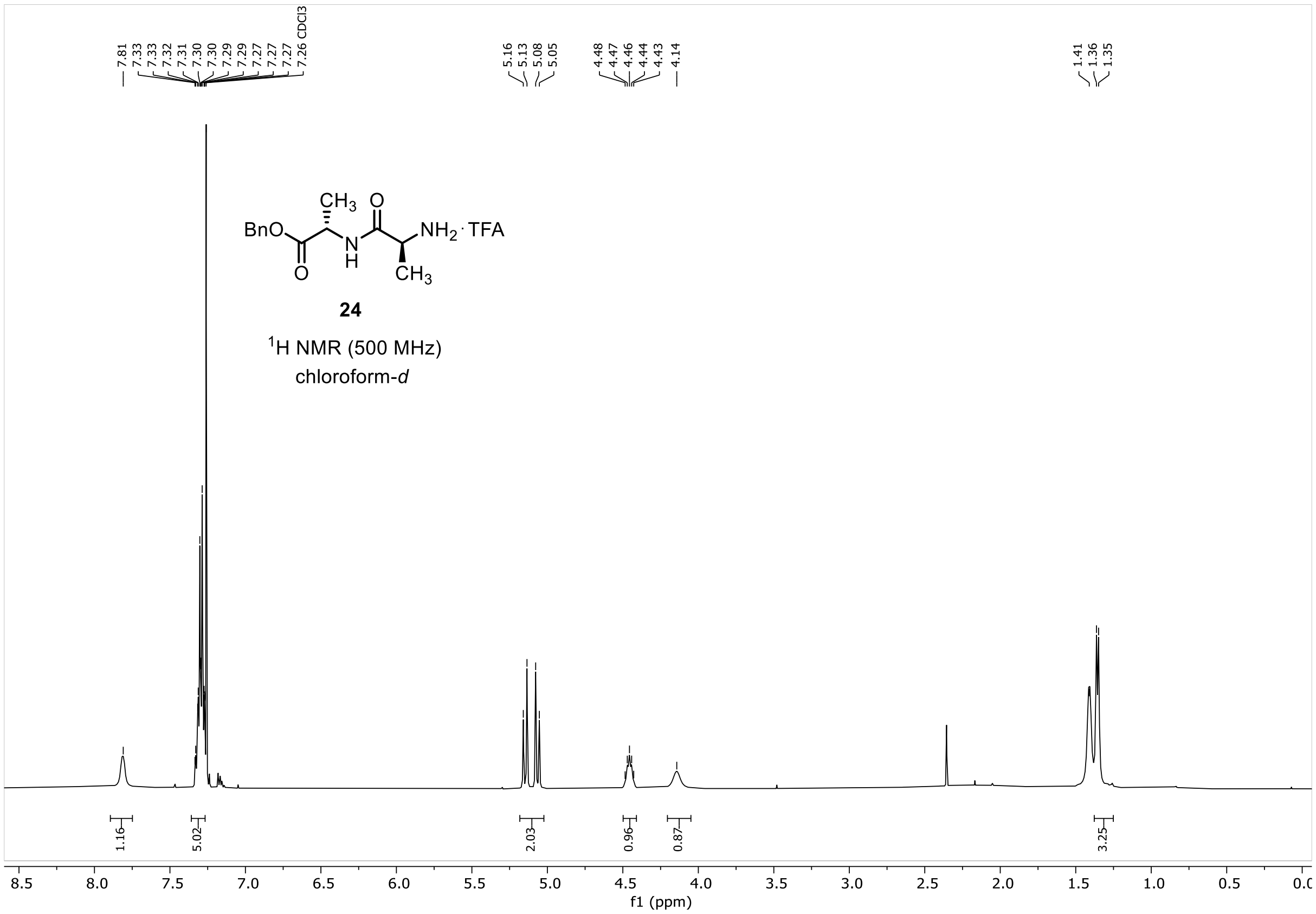
7.81
7.33
7.33
7.32
7.31
7.30
7.30
7.29
7.29
7.27
7.27
7.26 CDCl3
5.16
5.13
5.08
5.05
4.48
4.47
4.46
4.44
4.43
4.14
1.41
1.36
1.35
BnO
CH3
O
N
H
O
NH2 · TFA
CH3
24
1H NMR (500 MHz)
chloroform-d
1.16
5.02
2.03
0.96
0.87
3.25
f1 (ppm)

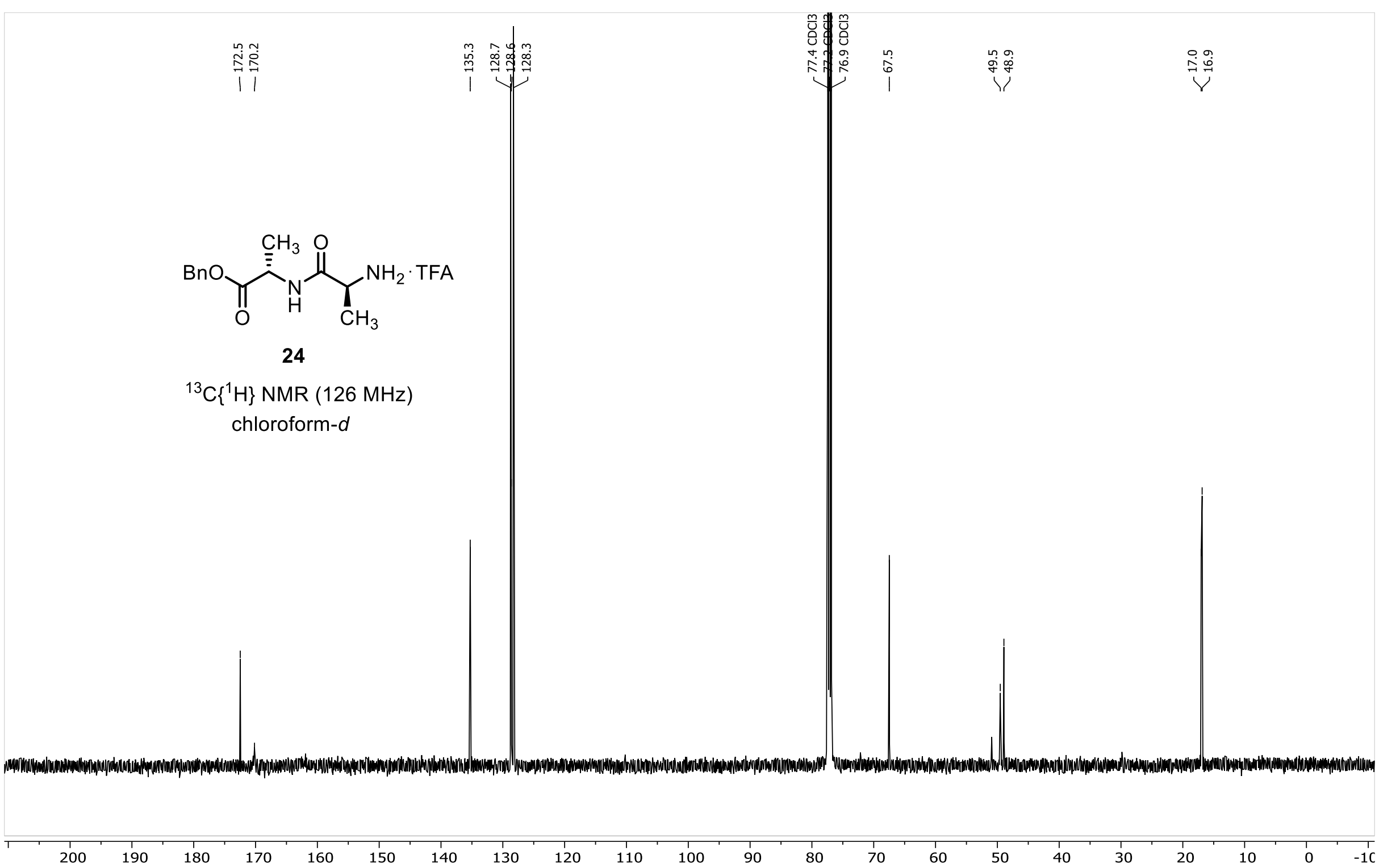
172.5
170.2
135.3
128.7
128.6
128.3
77.4 CDCl3
76.9 CDCl3
67.5
49.5
48.9
17.0
16.9
BnO
CH3
O
N
H
O
NH2 · TFA
CH3
24
13C{1H} NMR (126 MHz)
chloroform-d
f1 (ppm)

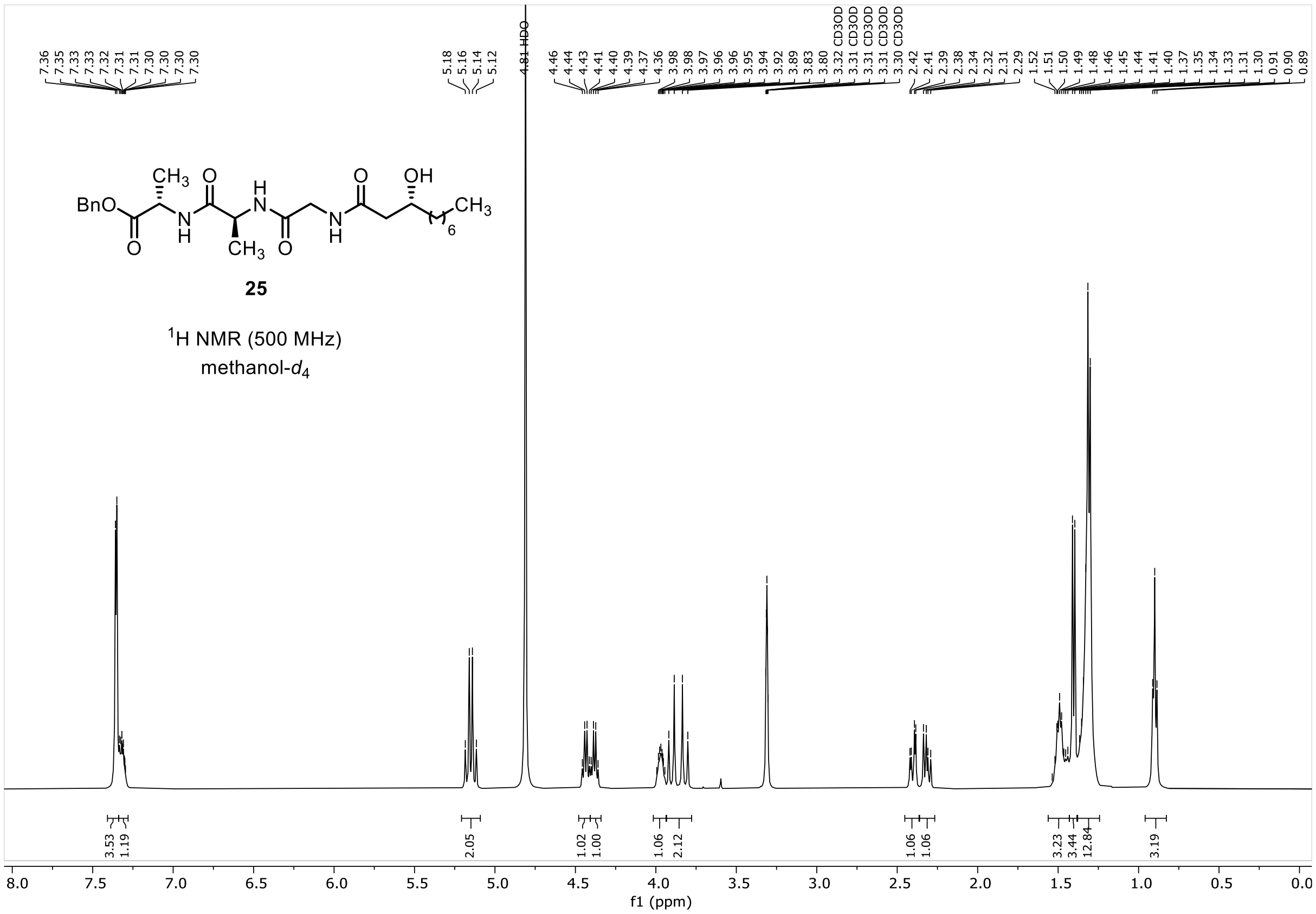
25
^{1}H NMR (500 MHz)
methanol-d_4
f1 (ppm)

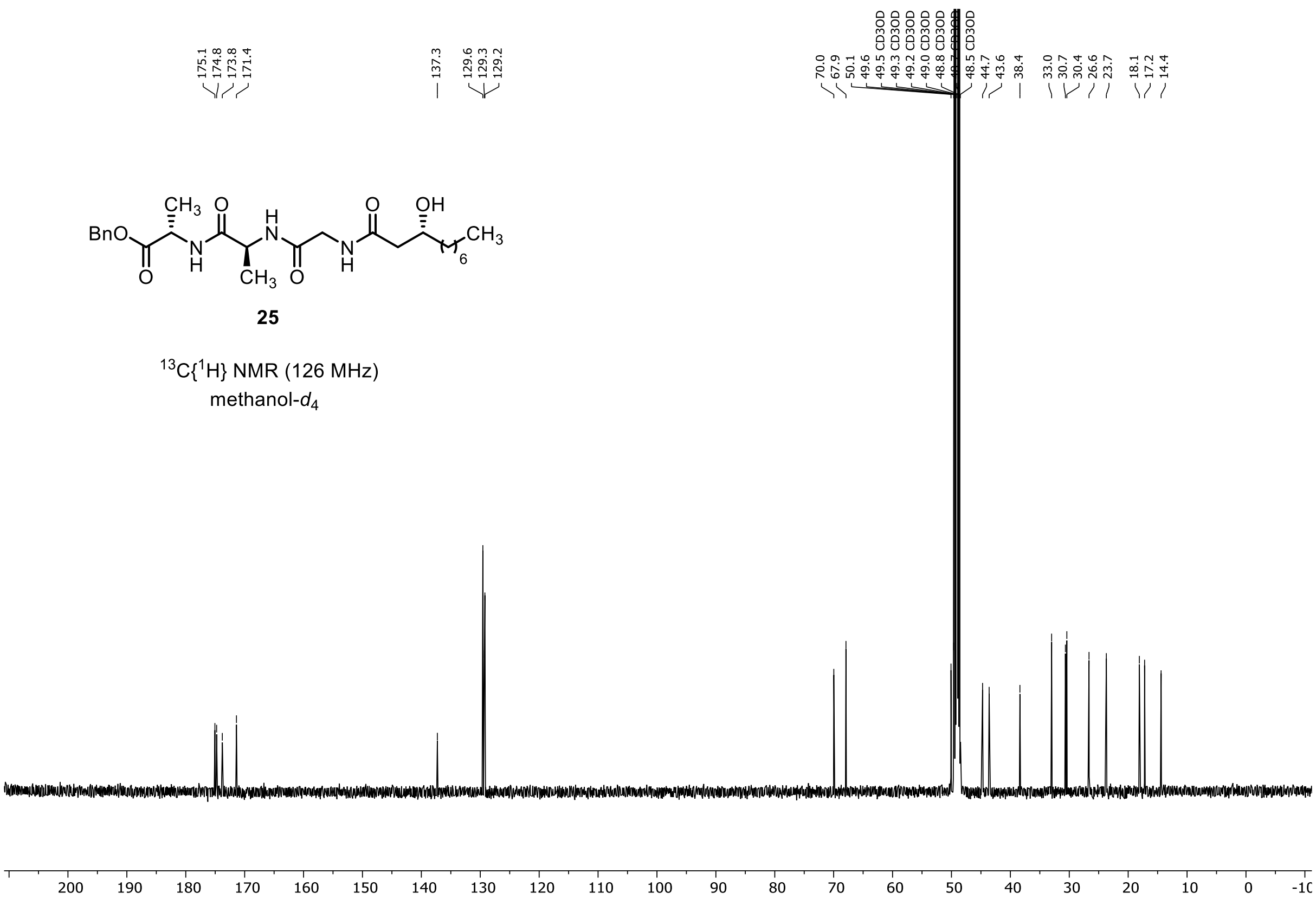
25
^{13}C{^{1}H} NMR (126 MHz)
methanol-d_4
f1 (ppm)

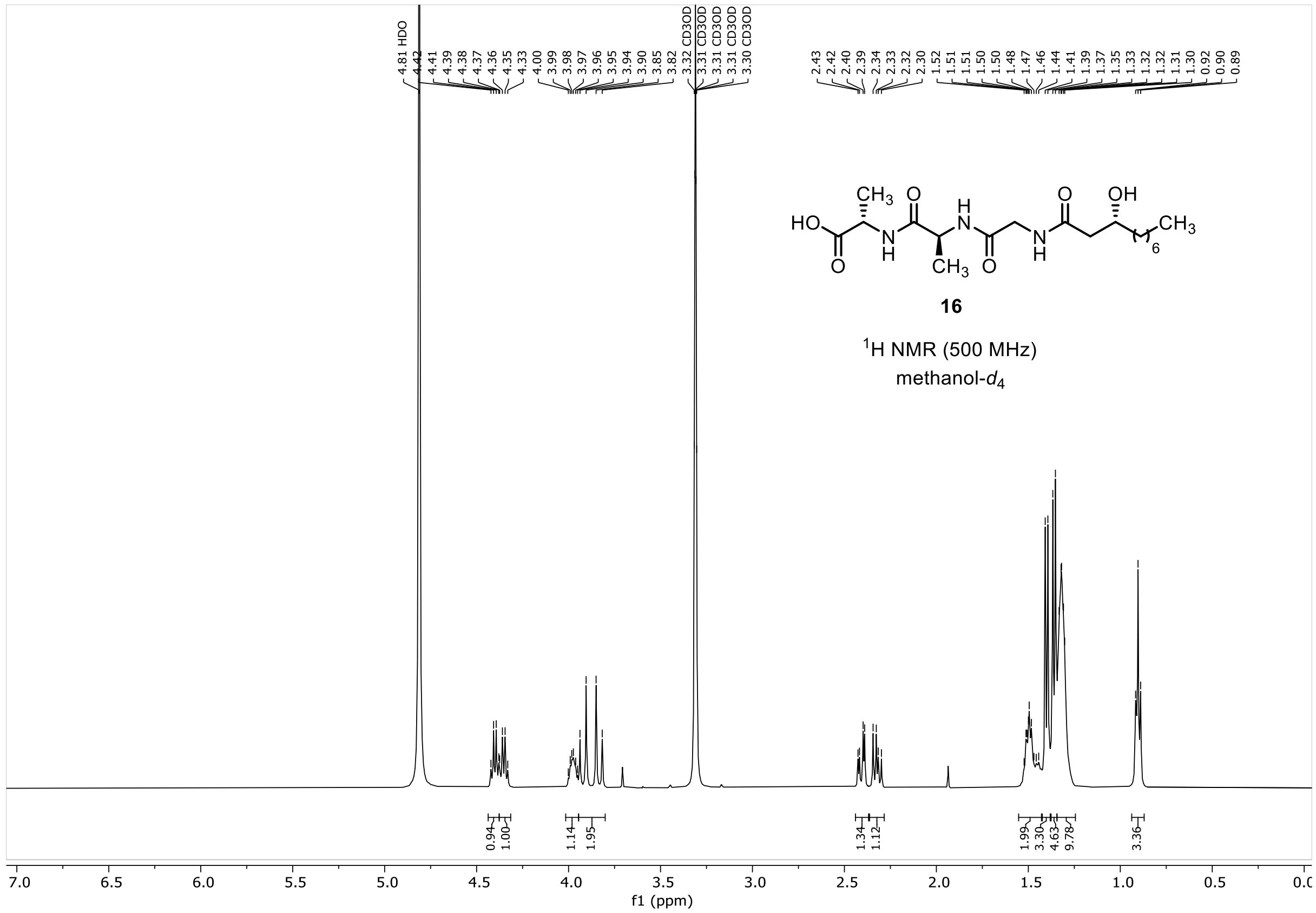
4.81 HDO
4.42
4.41
4.39
4.38
4.37
4.36
4.35
4.33
4.00
3.99
3.98
3.97
3.96
3.95
3.94
3.90
3.85
3.82
3.32 CD3OD
3.31 CD3OD
3.31 CD3OD
3.31 CD3OD
3.30 CD3OD
2.43
2.42
2.40
2.39
2.34
2.33
2.32
2.30
1.52
1.51
1.51
1.50
1.50
1.48
1.47
1.46
1.44
1.41
1.39
1.37
1.35
1.33
1.32
1.32
1.31
1.30
0.92
0.90
0.89
HO
CH3
O
N
H
O
H
N
CH3
O
N
H
O
OH
CH3
6
16
1H NMR (500 MHz)
methanol-d4
0.94
1.00
1.14
1.95
1.34
1.12
1.99
3.30
4.63
9.78
3.36
7.0
6.5
6.0
5.5
5.0
4.5
4.0
3.5
3.0
2.5
2.0
1.5
1.0
0.5
0.0
f1 (ppm)

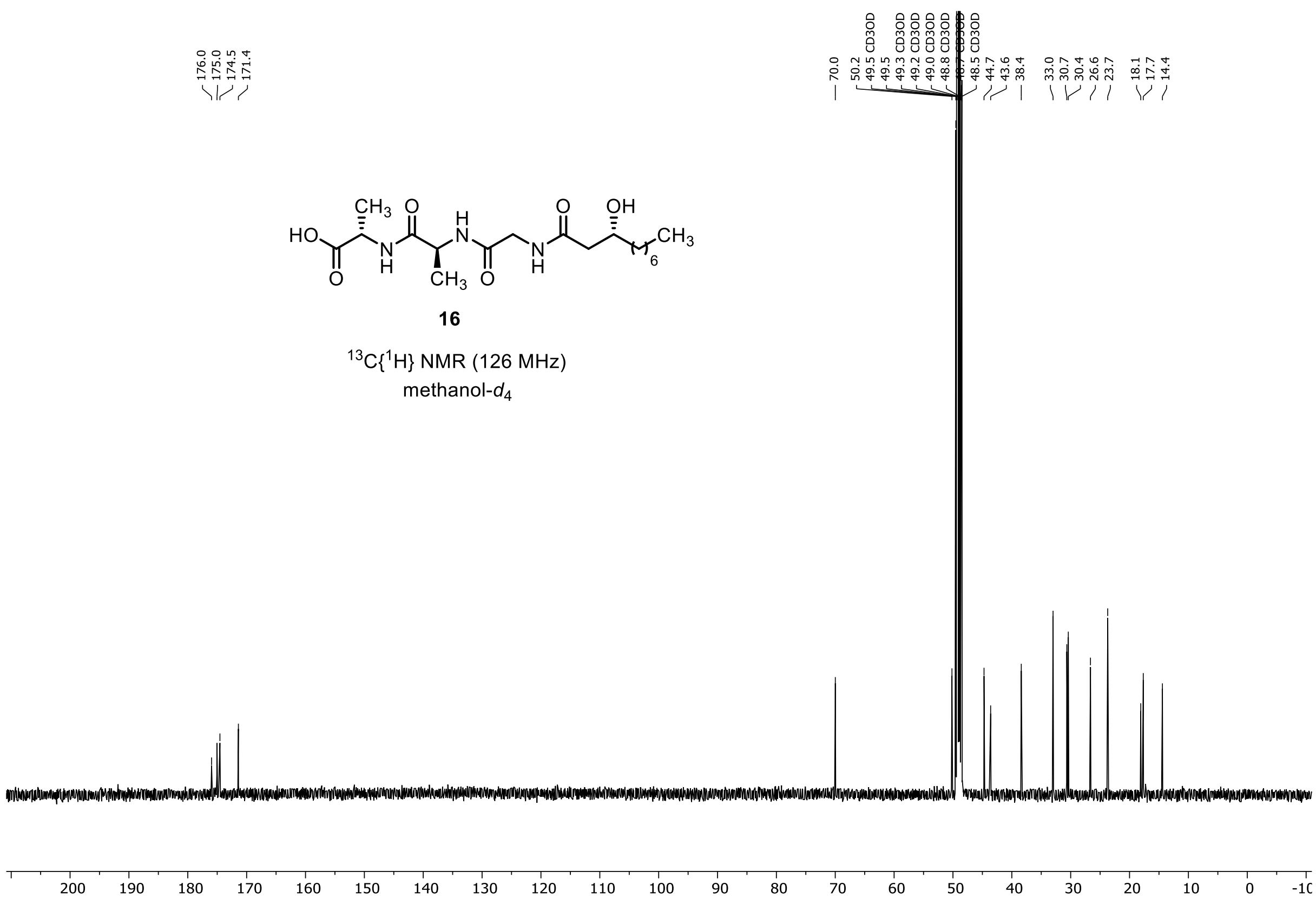
176.0
175.0
174.5
171.4
70.0
50.2
49.5 CD3OD
49.3 CD3OD
49.2 CD3OD
49.0 CD3OD
48.8 CD3OD
48.7 CD3OD
48.5 CD3OD
44.7
43.6
38.4
33.0
30.7
30.4
26.6
23.7
18.1
17.7
14.4
HO
CH3
O
N
H
O
H
N
CH3
O
N
H
O
OH
CH3
6
16
13C{1H} NMR (126 MHz)
methanol-d4
200
190
180
170
160
150
140
130
120
110
100
90
80
70
60
50
40
30
20
10
0
-10
f1 (ppm)

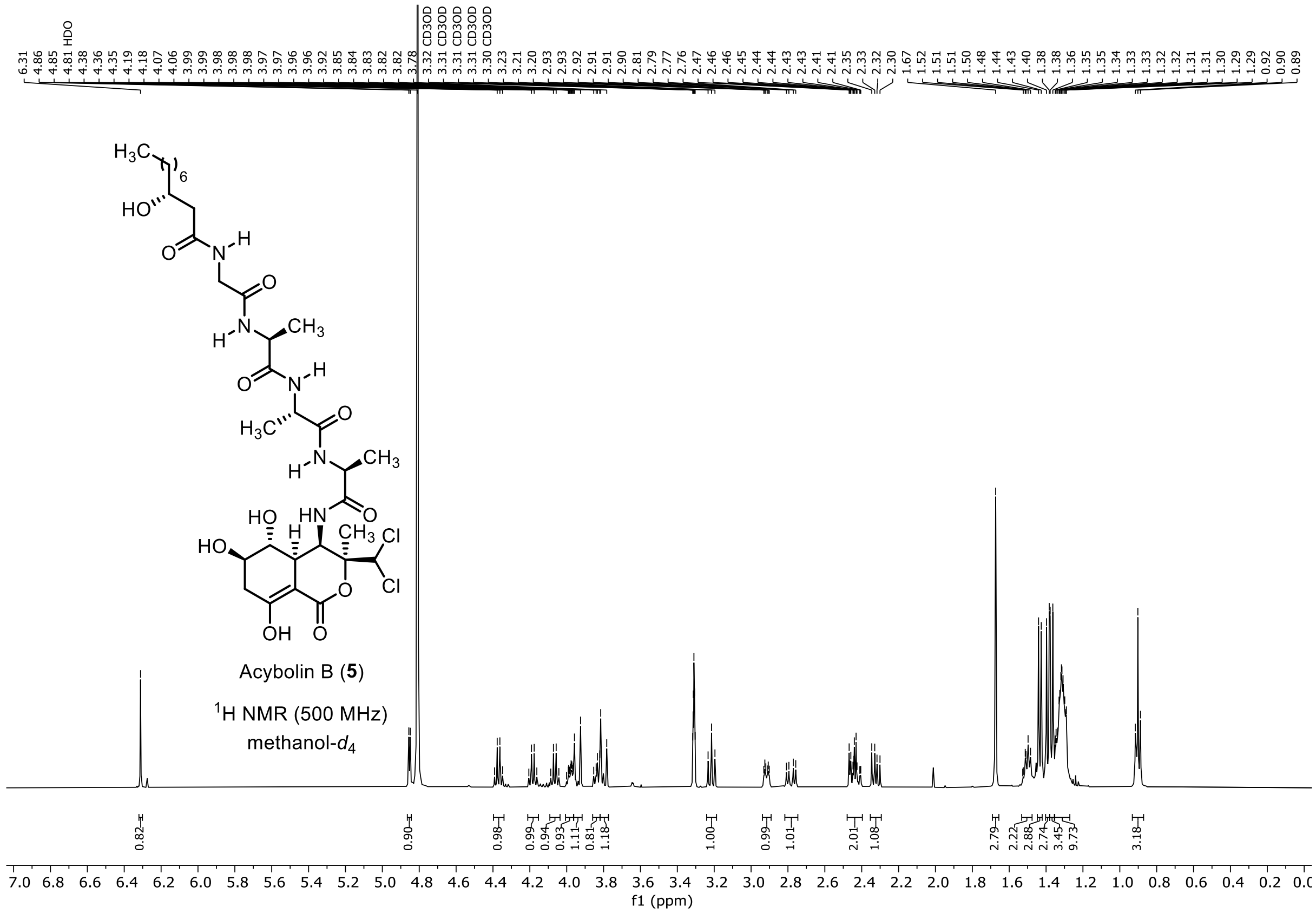
Acybolin B (5)
1H NMR (500 MHz)
methanol-d4
f1 (ppm)

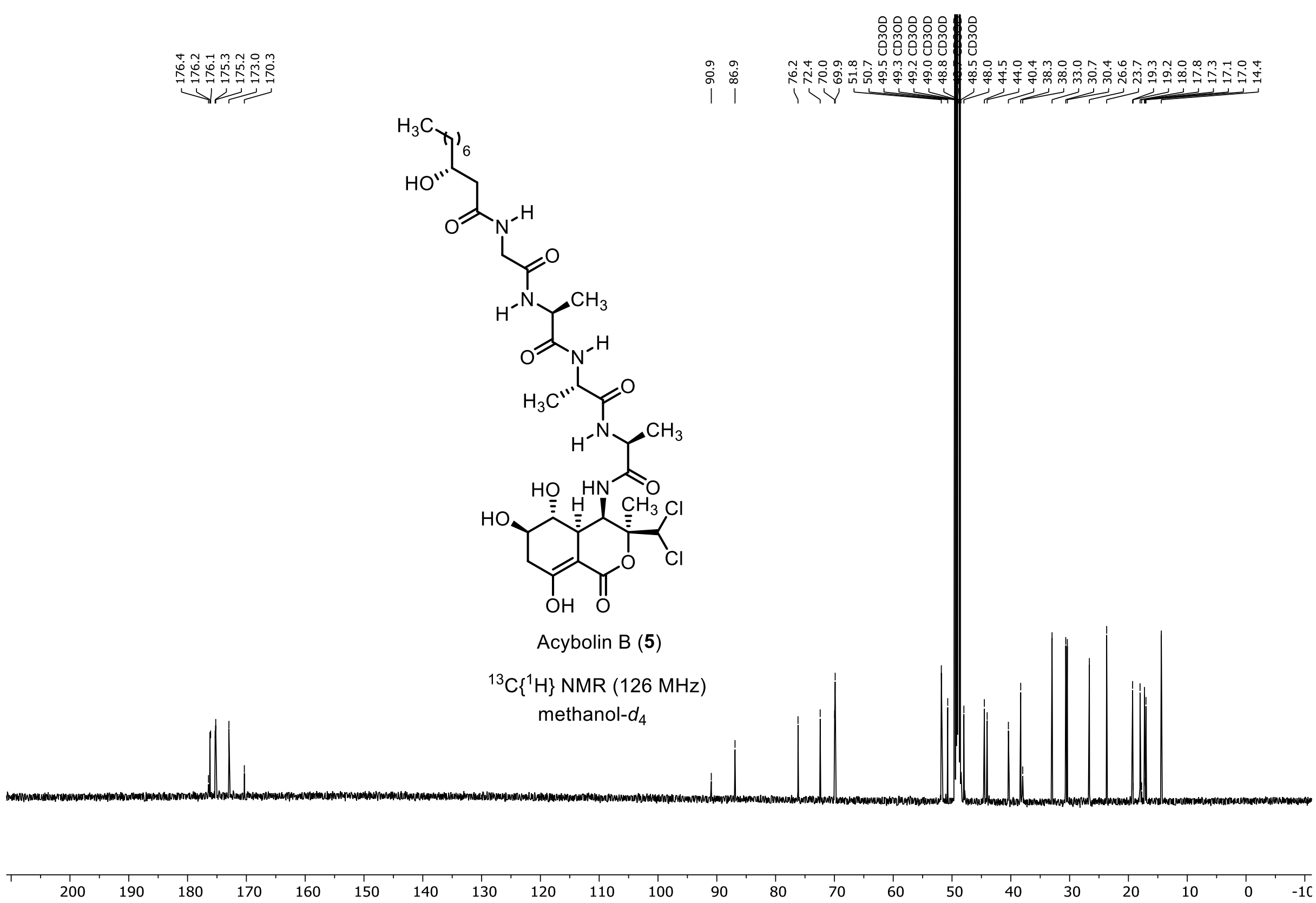
Acybolin B (5)
13C{1H} NMR (126 MHz)
methanol-d4
f1 (ppm)

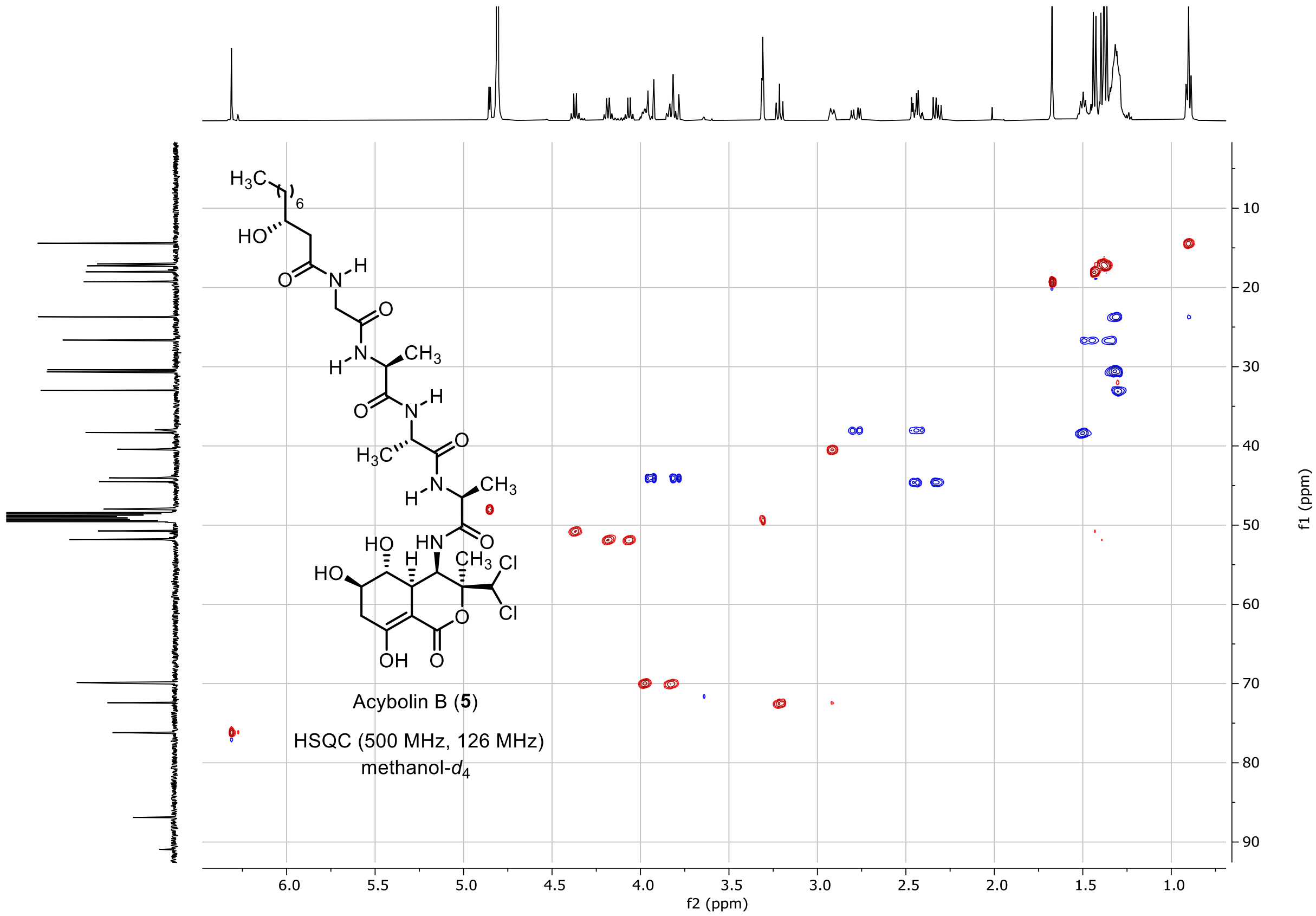

Acybolin B (**5**)
HSQC (500 MHz, 126 MHz)
methanol-d_4
f2 (ppm)
f1 (ppm)

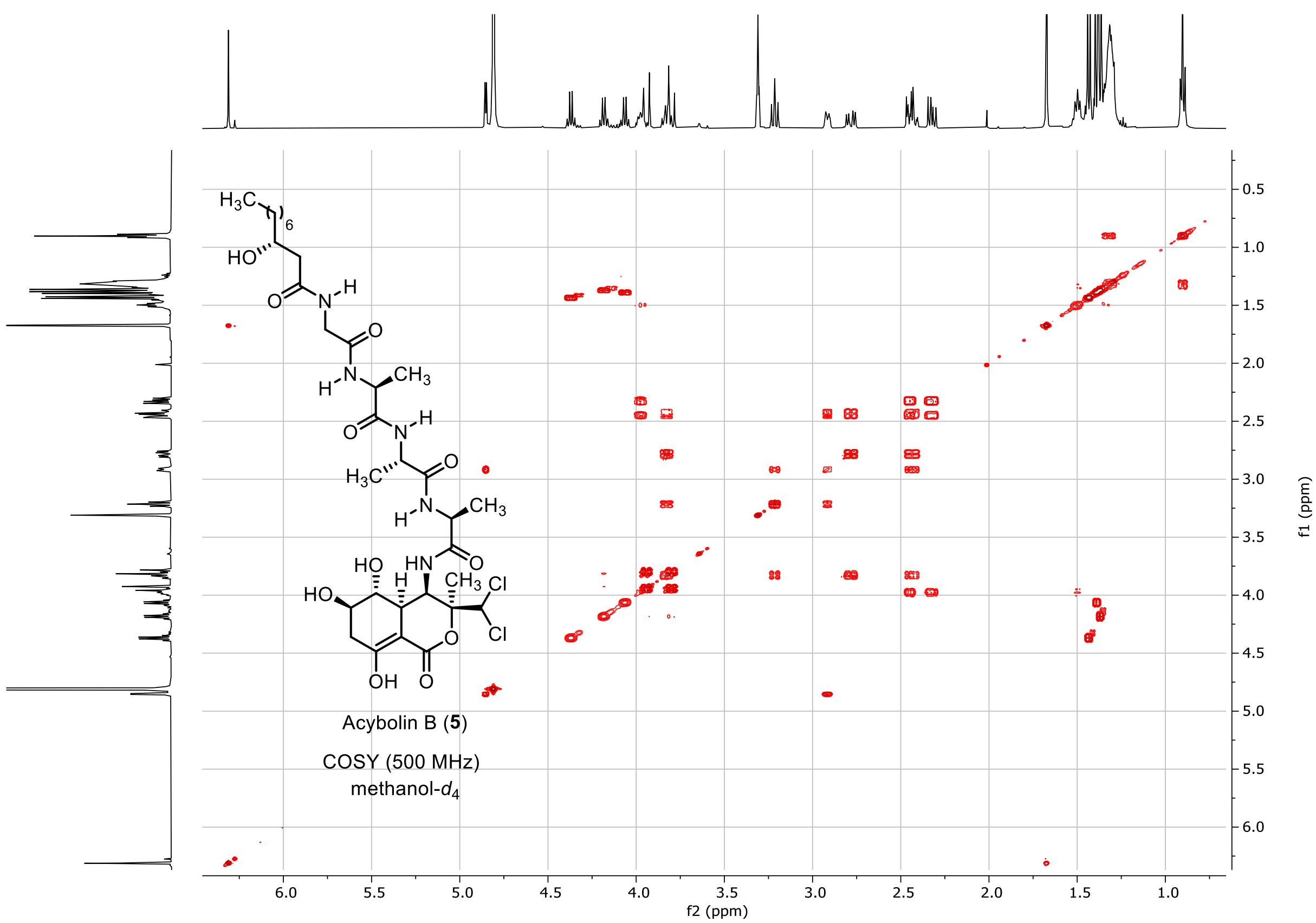

Acybolin B (**5**)
COSY (500 MHz)
methanol-d_4
f2 (ppm)
f1 (ppm)

NMR SPECTRA OF ACYBOLIN H

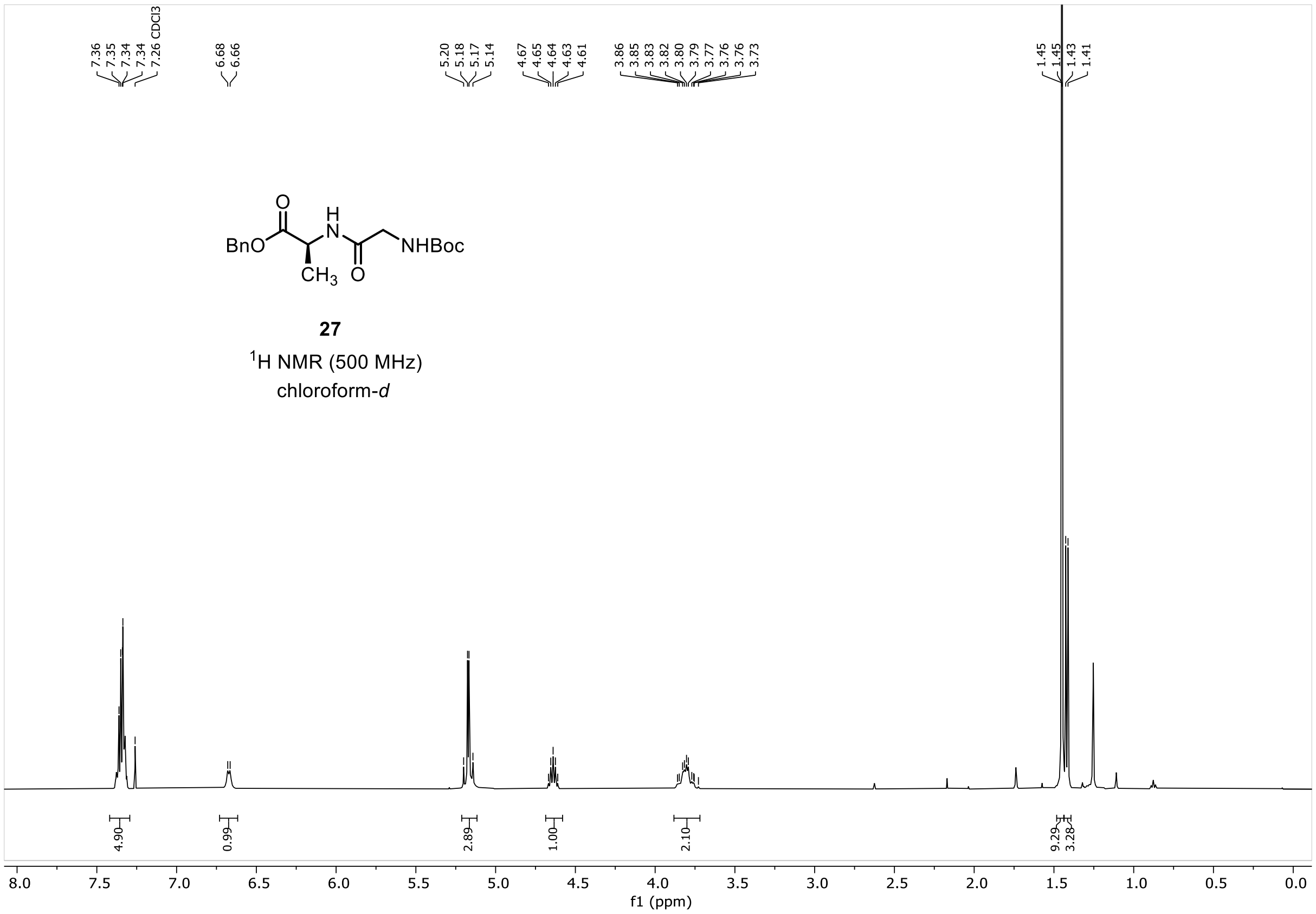

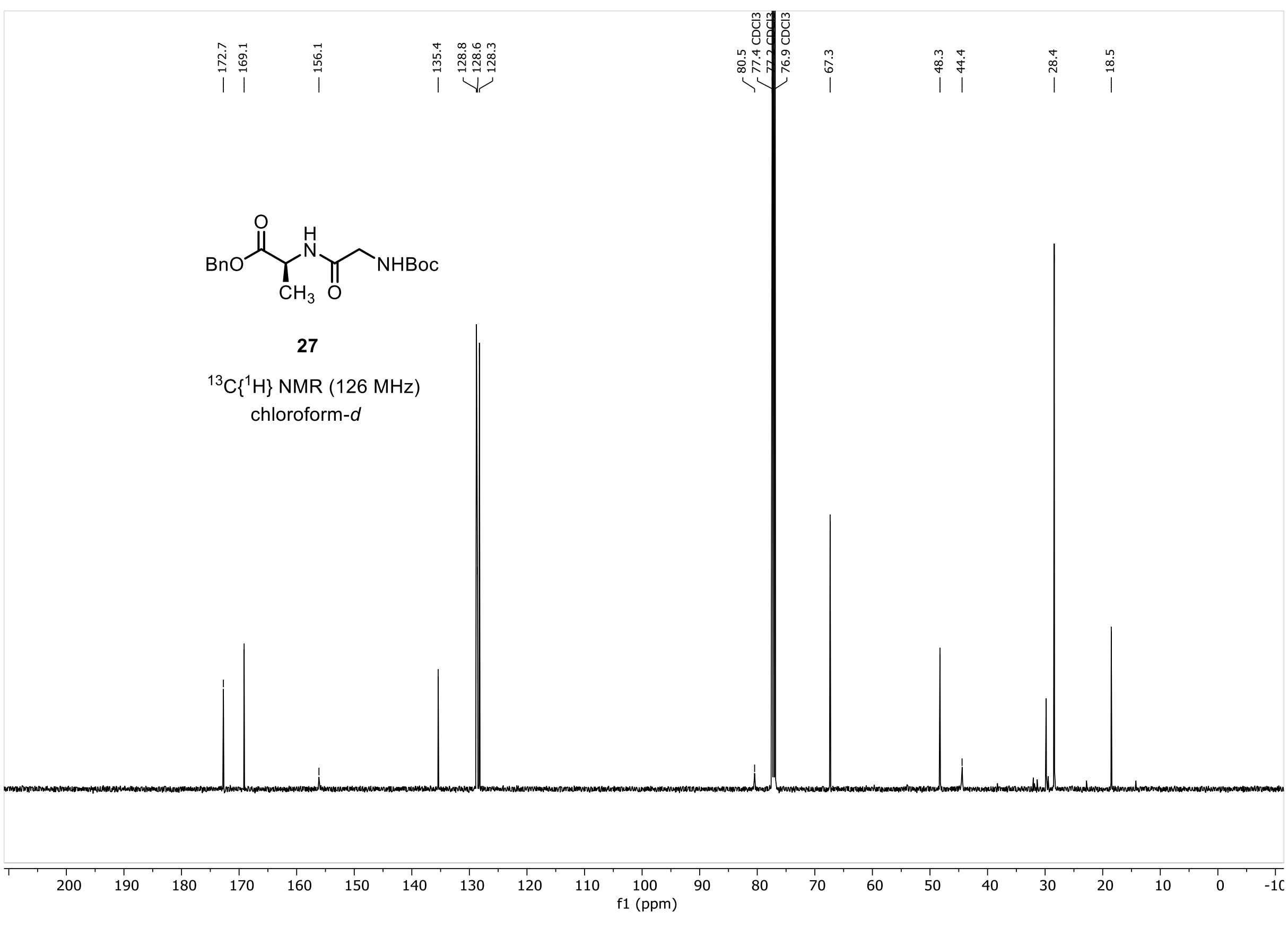

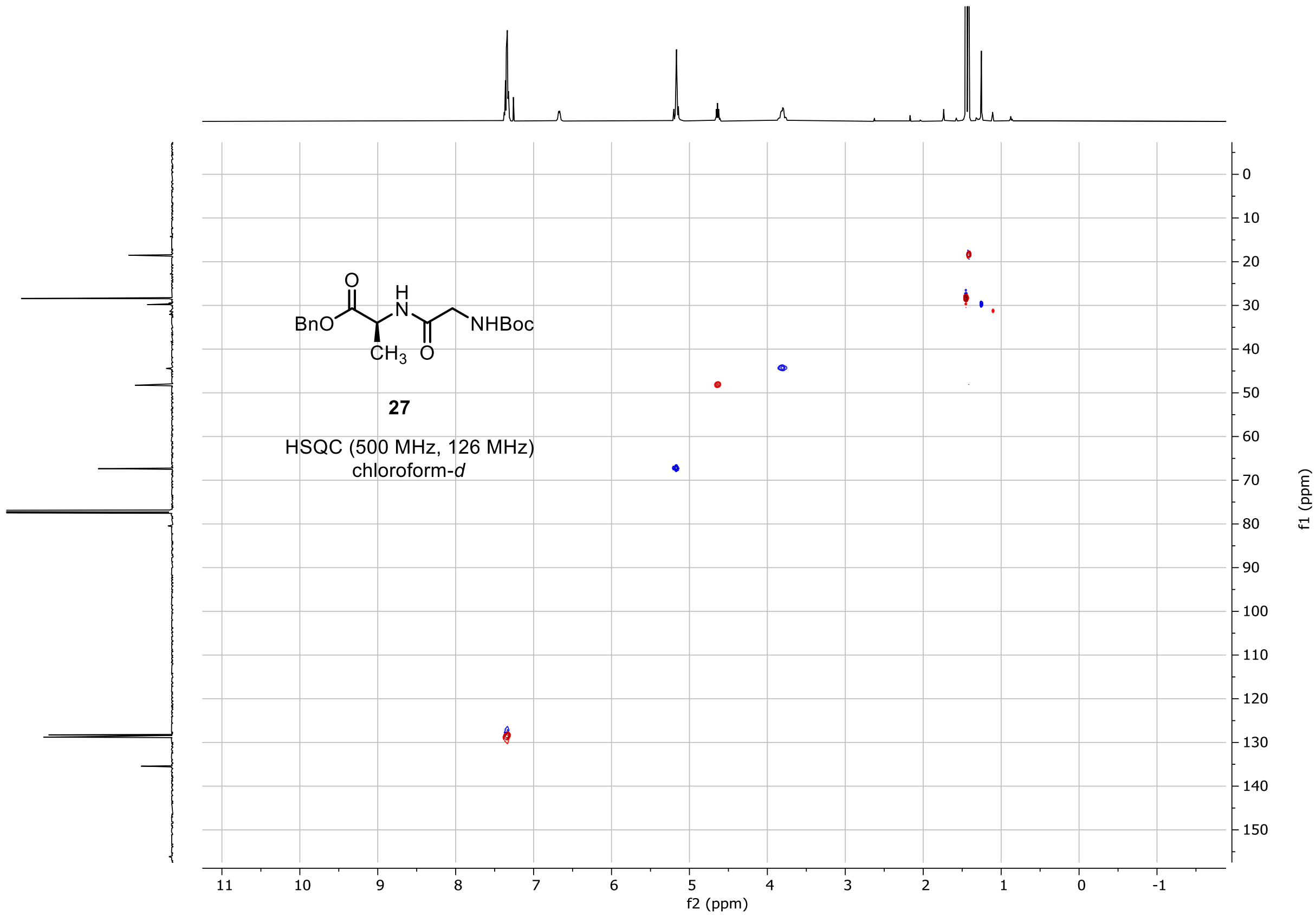
BnO
NHBoc
27
HSQC (500 MHz, 126 MHz)
chloroform-*d*
f2 (ppm)
f1 (ppm)

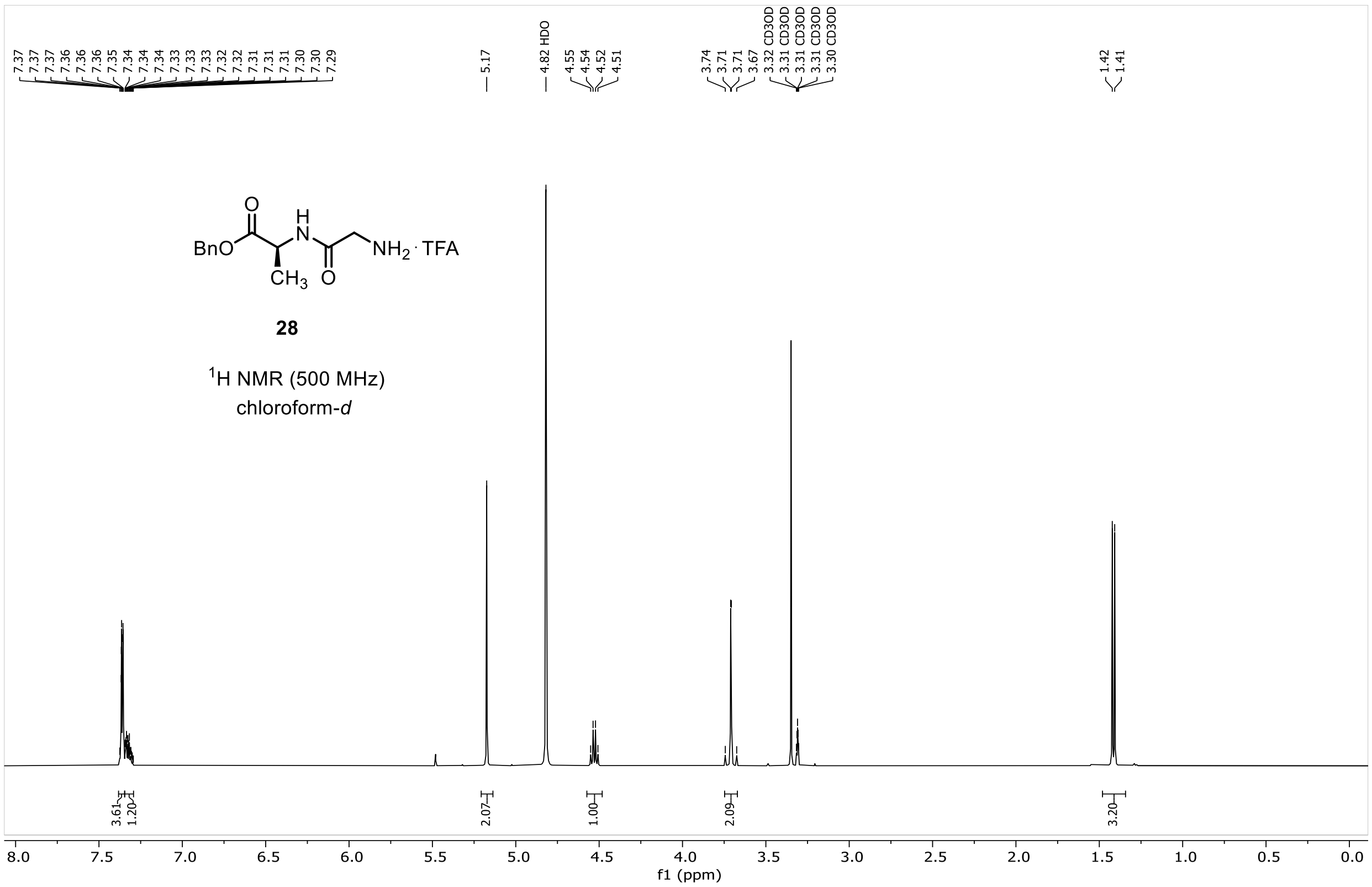
7.37 7.37 7.37 7.36 7.36 7.36 7.35 7.34 7.34 7.34 7.33 7.33 7.33 7.32 7.32 7.31 7.31 7.31 7.30 7.30 7.29
5.17
4.82 HDO
4.55 4.54 4.52 4.51
3.74 3.71 3.71 3.67 3.32 CD3OD 3.31 CD3OD 3.31 CD3OD 3.31 CD3OD 3.30 CD3OD
1.42 1.41
BnO
O
H
N
O
NH2 · TFA
CH3
28
1H NMR (500 MHz)
chloroform-d
3.61
1.20
2.07
1.00
2.09
3.20
8.0 7.5 7.0 6.5 6.0 5.5 5.0 4.5 4.0 3.5 3.0 2.5 2.0 1.5 1.0 0.5 0.0
f1 (ppm)

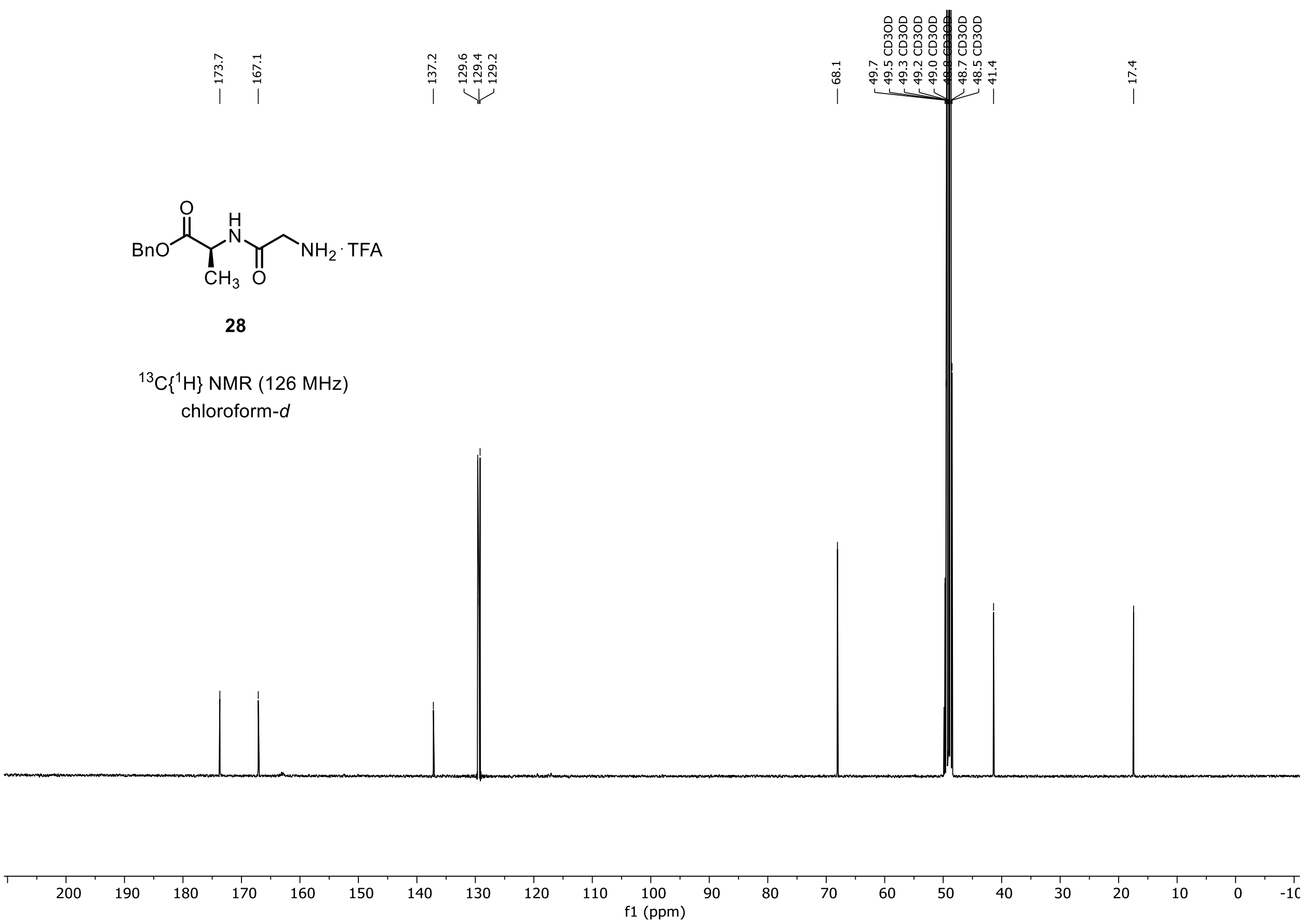
173.7
167.1
137.2
129.6 129.4 129.2
68.1
49.7 49.5 CD3OD 49.3 CD3OD 49.2 CD3OD 49.0 CD3OD 48.8 CD3OD 48.7 CD3OD 48.5 CD3OD
41.4
17.4
BnO
O
H
N
O
NH2 · TFA
CH3
28
13C{1H} NMR (126 MHz)
chloroform-d
200 190 180 170 160 150 140 130 120 110 100 90 80 70 60 50 40 30 20 10 0 -10
f1 (ppm)

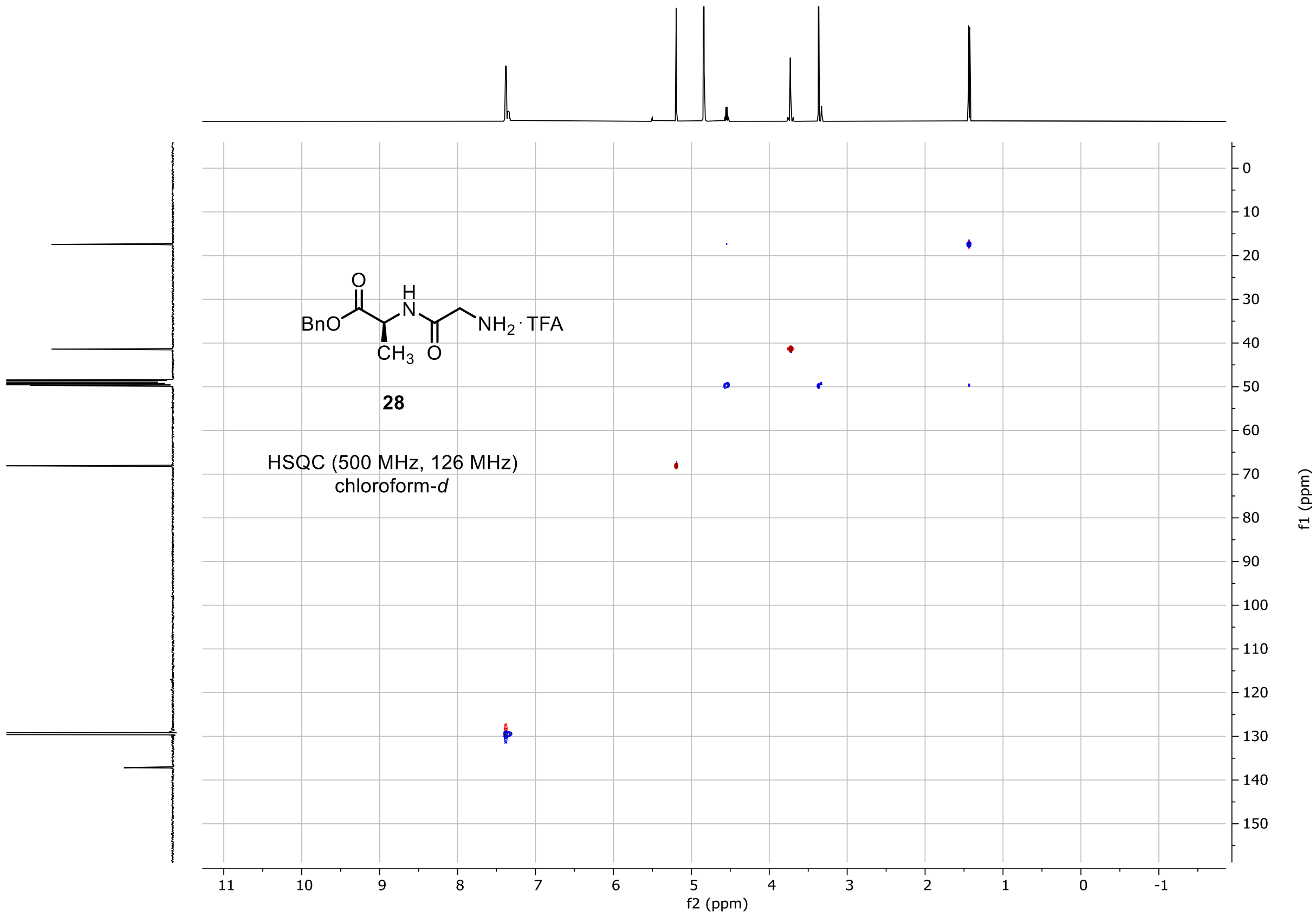
BnO
O
H
N
O
NH2 · TFA
CH3
28
HSQC (500 MHz, 126 MHz)
chloroform-d
f1 (ppm)
f2 (ppm)

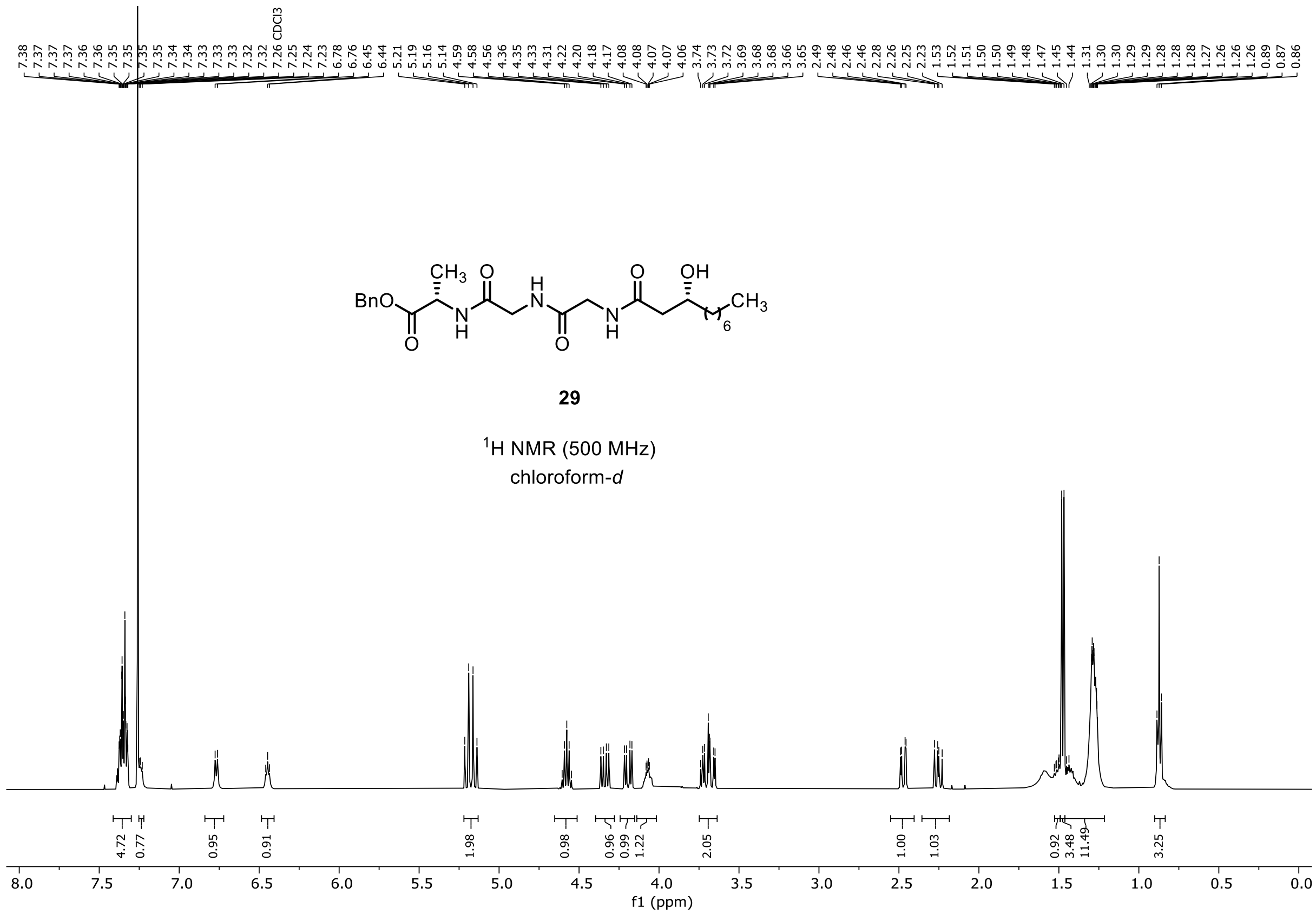
7.38 7.37 7.37 7.37 7.36 7.36 7.35 7.35 7.35 7.35 7.34 7.34 7.33 7.33 7.33 7.32 7.32 7.26 CDCl3 7.25 7.24 7.23 6.78 6.76 6.45 6.44 5.21 5.19 5.16 5.14 4.59 4.58 4.56 4.36 4.35 4.33 4.31 4.22 4.20 4.18 4.17 4.08 4.08 4.07 4.07 4.06 3.74 3.73 3.72 3.69 3.68 3.68 3.66 3.65 2.49 2.48 2.46 2.46 2.28 2.26 2.25 2.25 2.23 1.53 1.52 1.51 1.50 1.50 1.49 1.48 1.47 1.45 1.44 1.31 1.30 1.29 1.29 1.28 1.28 1.28 1.27 1.26 1.26 1.26 0.89 0.87 0.86
BnO
CH3
OH
CH3
6
29
1H NMR (500 MHz)
chloroform-d
4.72 0.77 0.95 0.91 1.98 0.98 0.96 0.99 1.22 2.05 1.00 1.03 0.92 3.48 11.49 3.25
8.0 7.5 7.0 6.5 6.0 5.5 5.0 4.5 4.0 3.5 3.0 2.5 2.0 1.5 1.0 0.5 0.0
f1 (ppm)

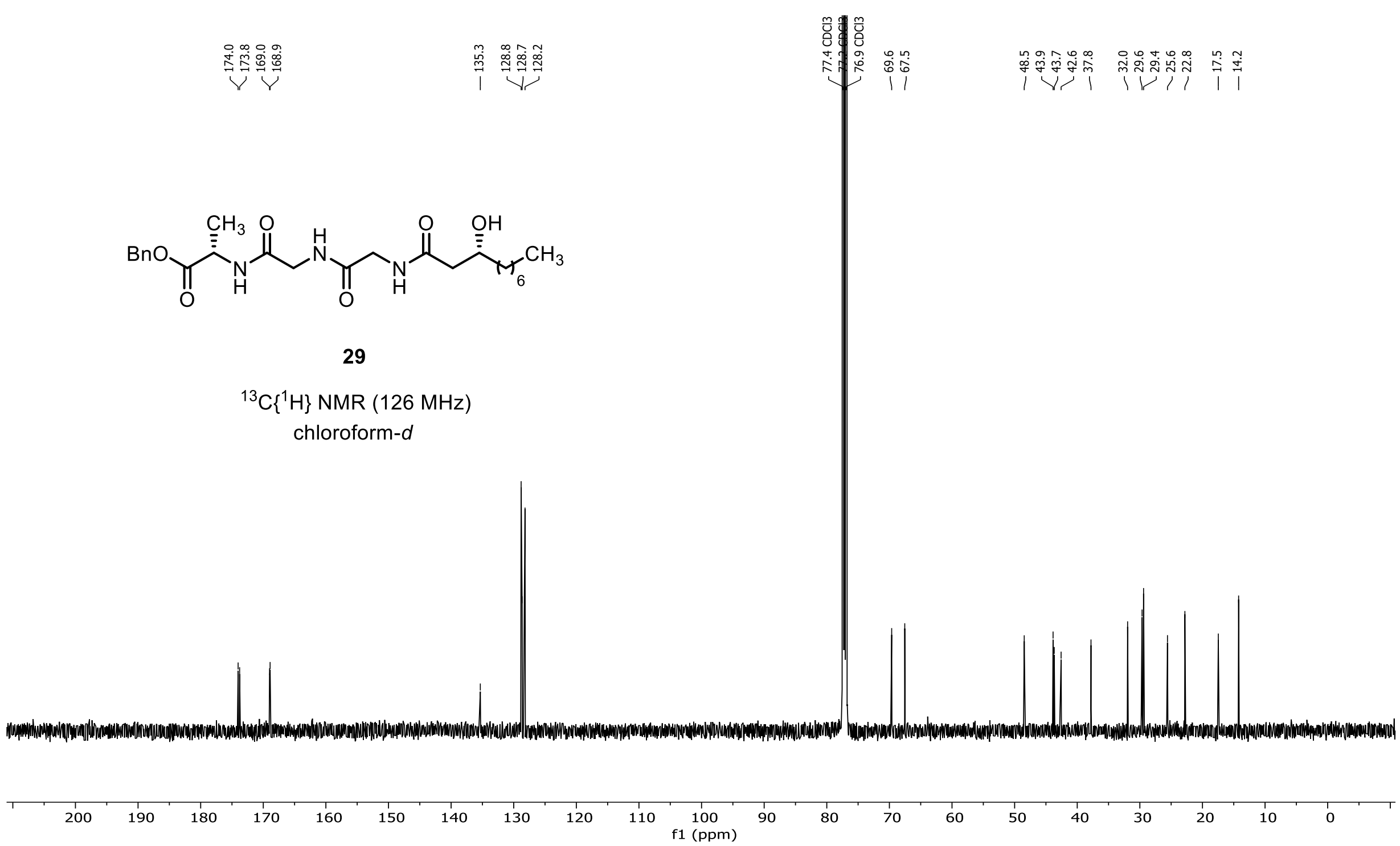
174.0 173.8 169.0 168.9 135.3 128.8 128.7 128.2 77.4 CDCl3 77.2 CDCl3 76.9 CDCl3 69.6 67.5 48.5 43.9 43.7 42.6 37.8 32.0 29.6 29.4 25.6 22.8 17.5 14.2
BnO
CH3
OH
CH3
6
29
13C{1H} NMR (126 MHz)
chloroform-d
200 190 180 170 160 150 140 130 120 110 100 90 80 70 60 50 40 30 20 10 0
f1 (ppm)

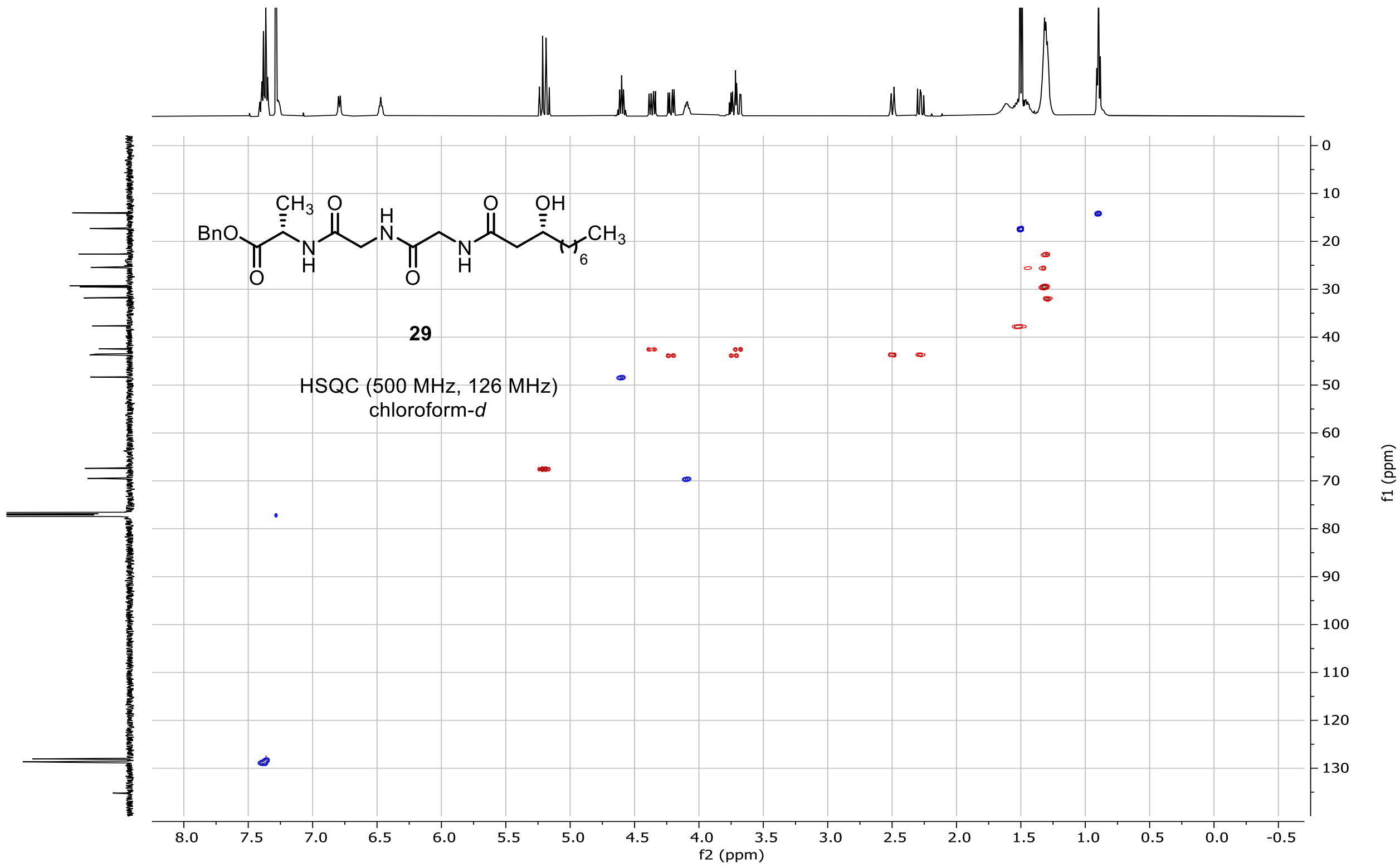
BnO
29
HSQC (500 MHz, 126 MHz)
chloroform-d
f1 (ppm)
f2 (ppm)

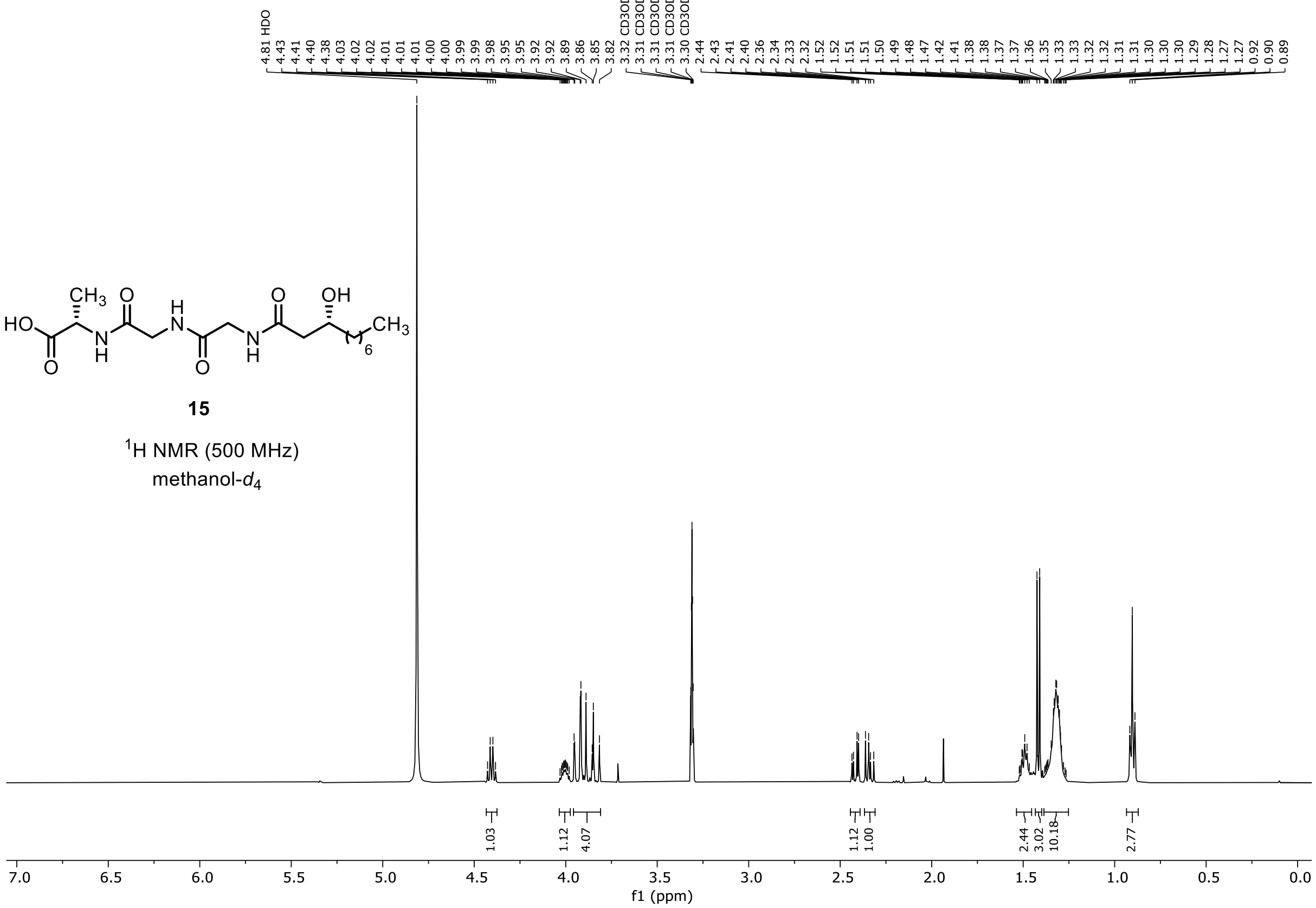

15

^1H NMR (500 MHz)
methanol-d_4

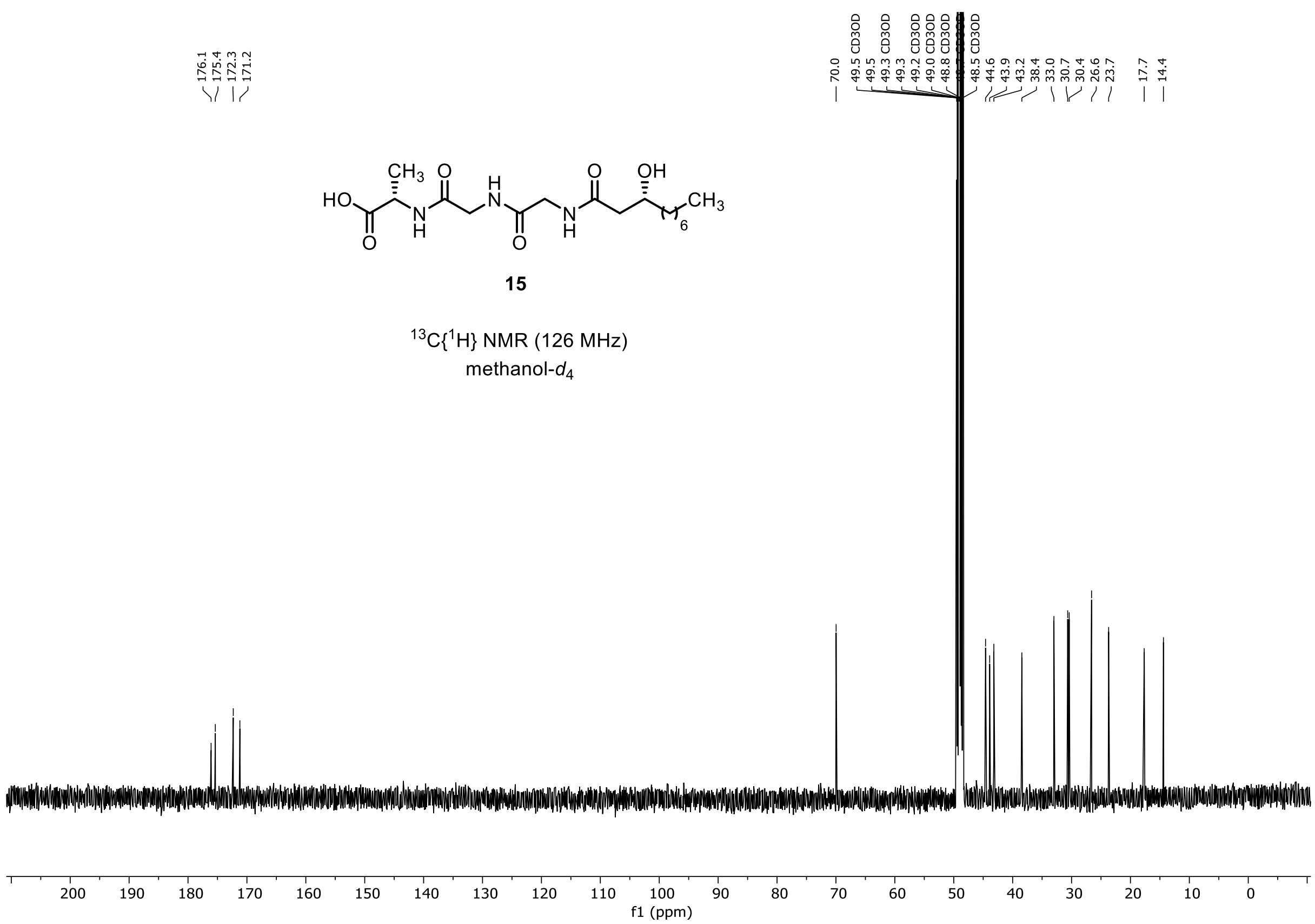

15

$^{13}C\{^1H\}$ NMR (126 MHz)
methanol-d_4

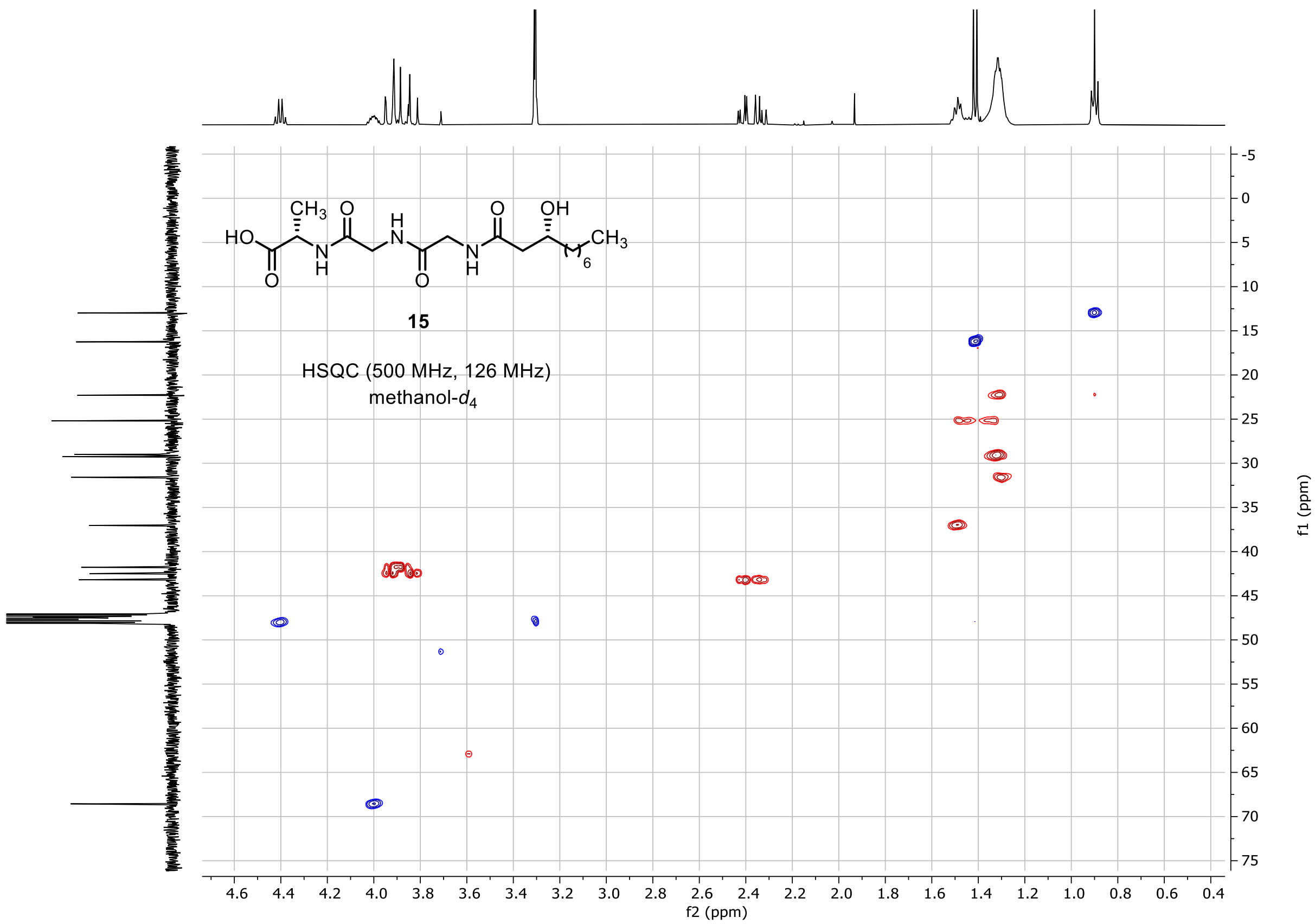
HO
CH3
O
H
N
N
H
O
N
H
O
OH
CH3
6
15
HSQC (500 MHz, 126 MHz)
methanol-d4
f2 (ppm)
f1 (ppm)

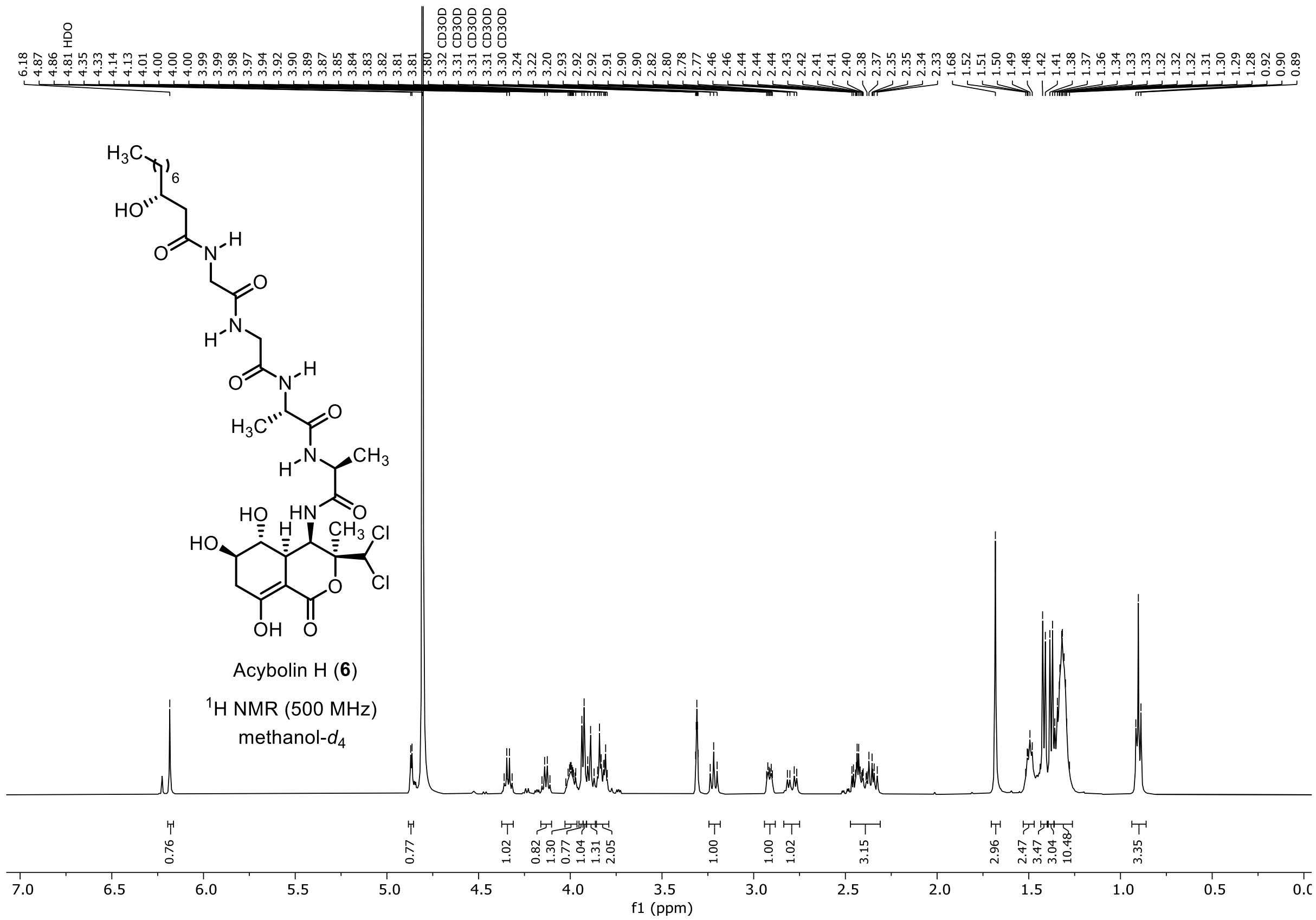
Acybolin H (6)
1H NMR (500 MHz)
methanol-d4
f1 (ppm)

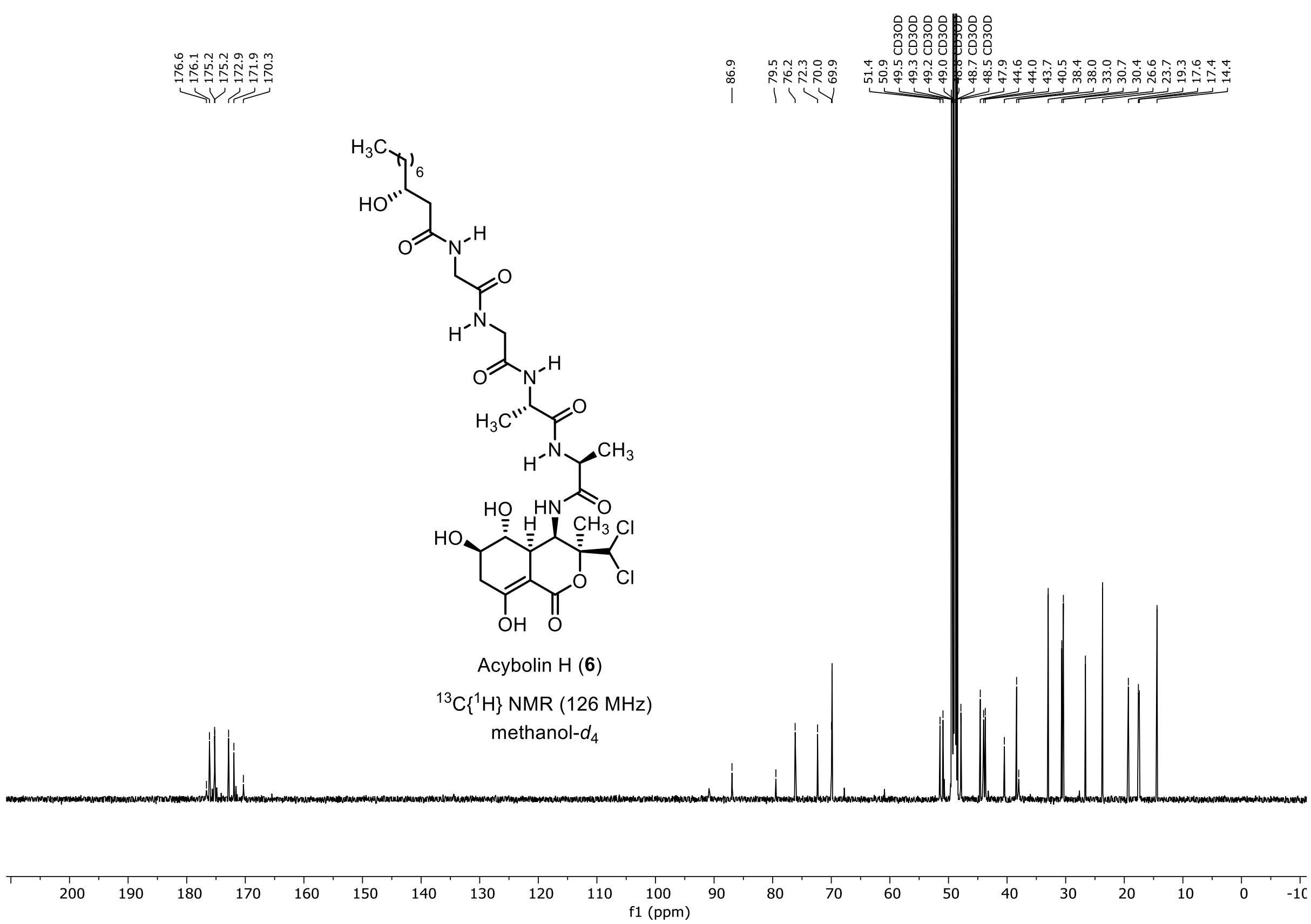
Acybolin H (6)
13C{1H} NMR (126 MHz)
methanol-d4
f1 (ppm)

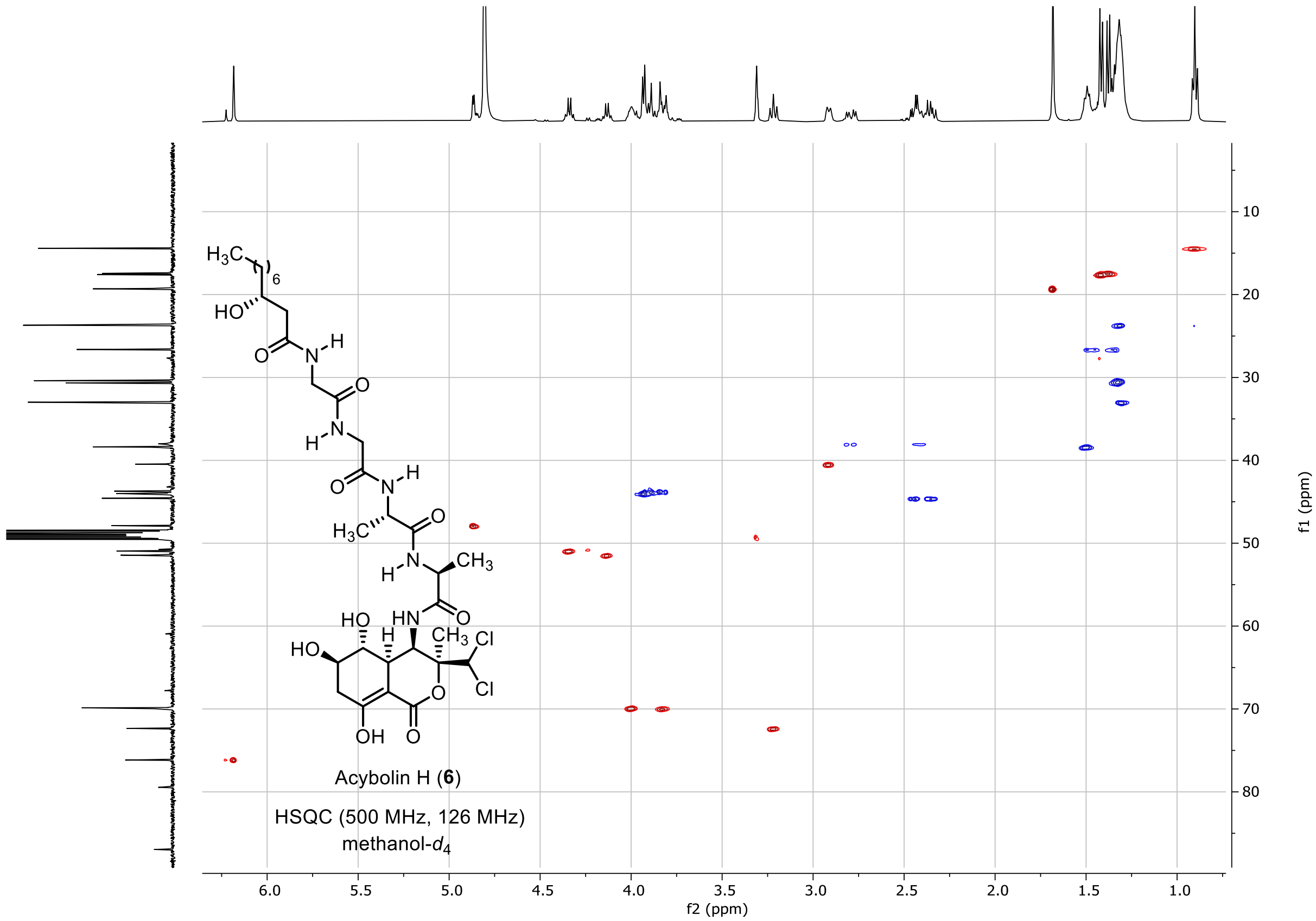
Acybolin H (**6**)
HSQC (500 MHz, 126 MHz)
methanol-d_4
f2 (ppm)
f1 (ppm)

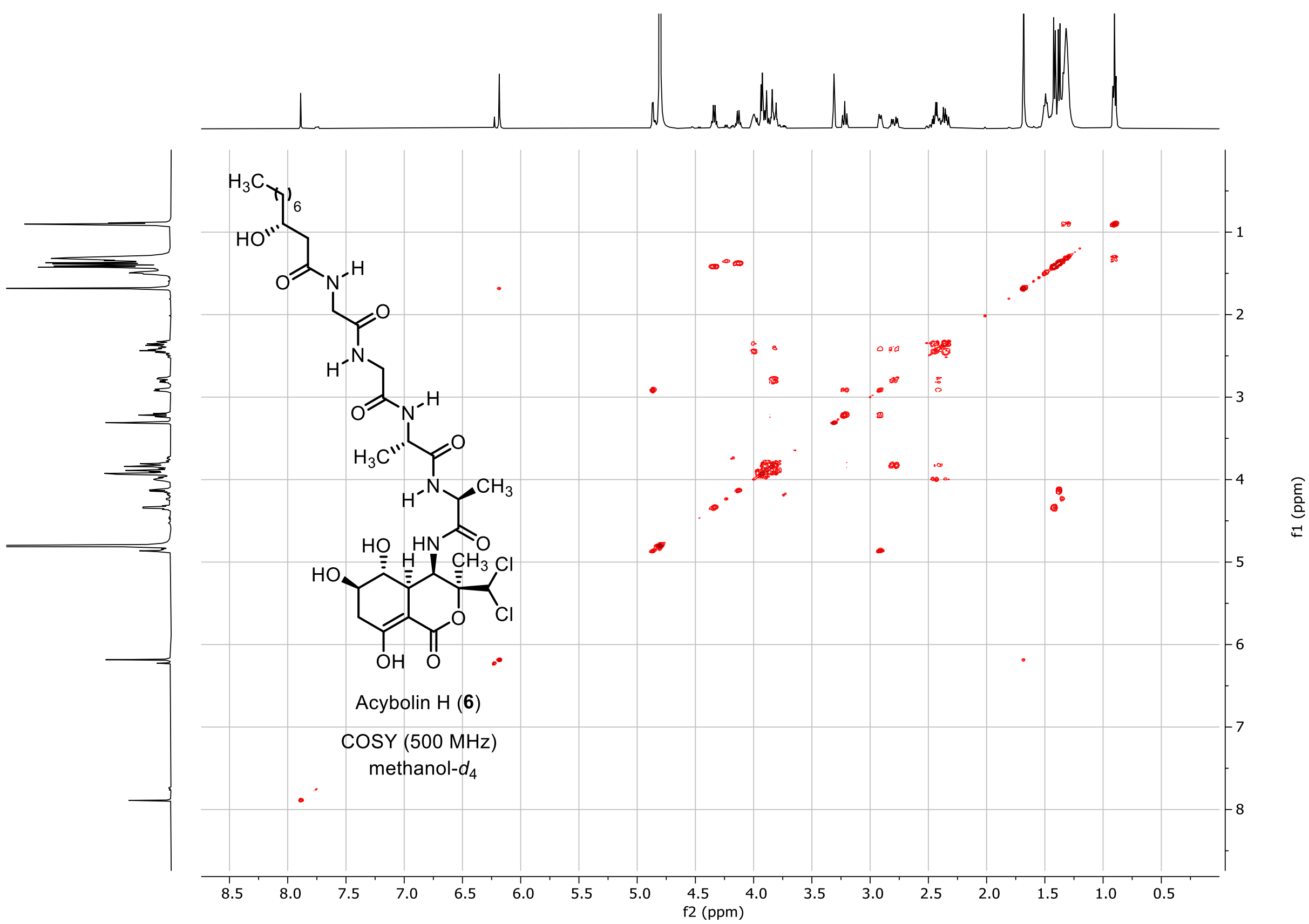
Acybolin H (**6**)
COSY (500 MHz)
methanol-d_4
f2 (ppm)
f1 (ppm)

NMR SPECTRA OF ACYBOLIN I

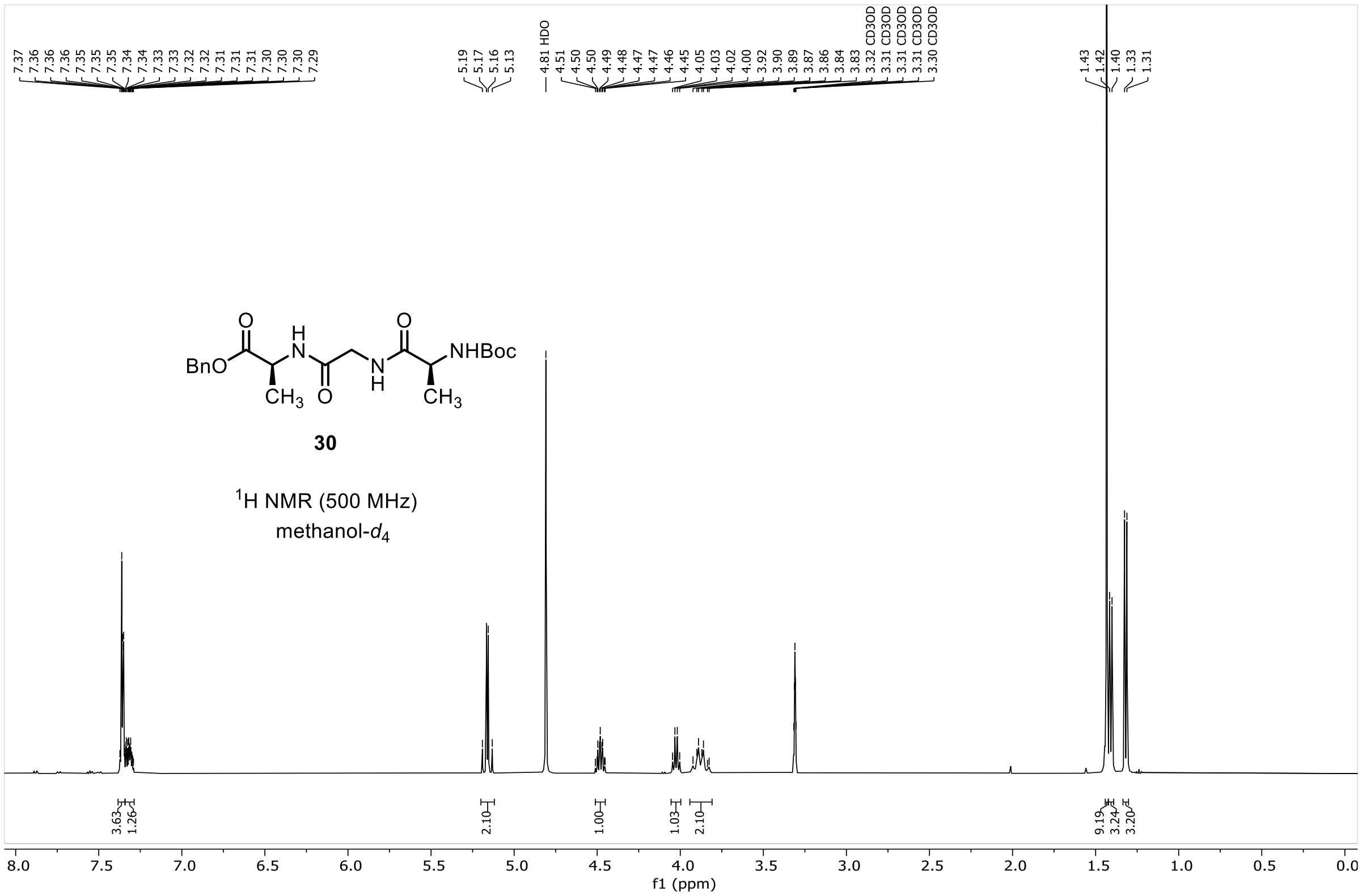

7.37 7.36 7.36 7.36 7.35 7.35 7.35 7.34 7.34 7.34 7.33 7.33 7.32 7.32 7.31 7.31 7.31 7.30 7.30 7.30 7.29
5.19 5.17 5.16 5.13
4.81 HDO
4.51 4.50 4.50 4.49 4.48 4.47 4.47 4.46 4.45 4.05 4.03 4.02 4.00 3.92 3.90 3.89 3.87 3.86 3.84 3.83
3.32 CD3OD 3.31 CD3OD 3.31 CD3OD 3.31 CD3OD 3.30 CD3OD
1.43 1.42 1.40 1.33 1.31
BnO
NHBoc
CH3
30
1H NMR (500 MHz)
methanol-d4
3.63 1.26 2.10 1.00 1.03 2.10 9.19 3.24 3.20
f1 (ppm)

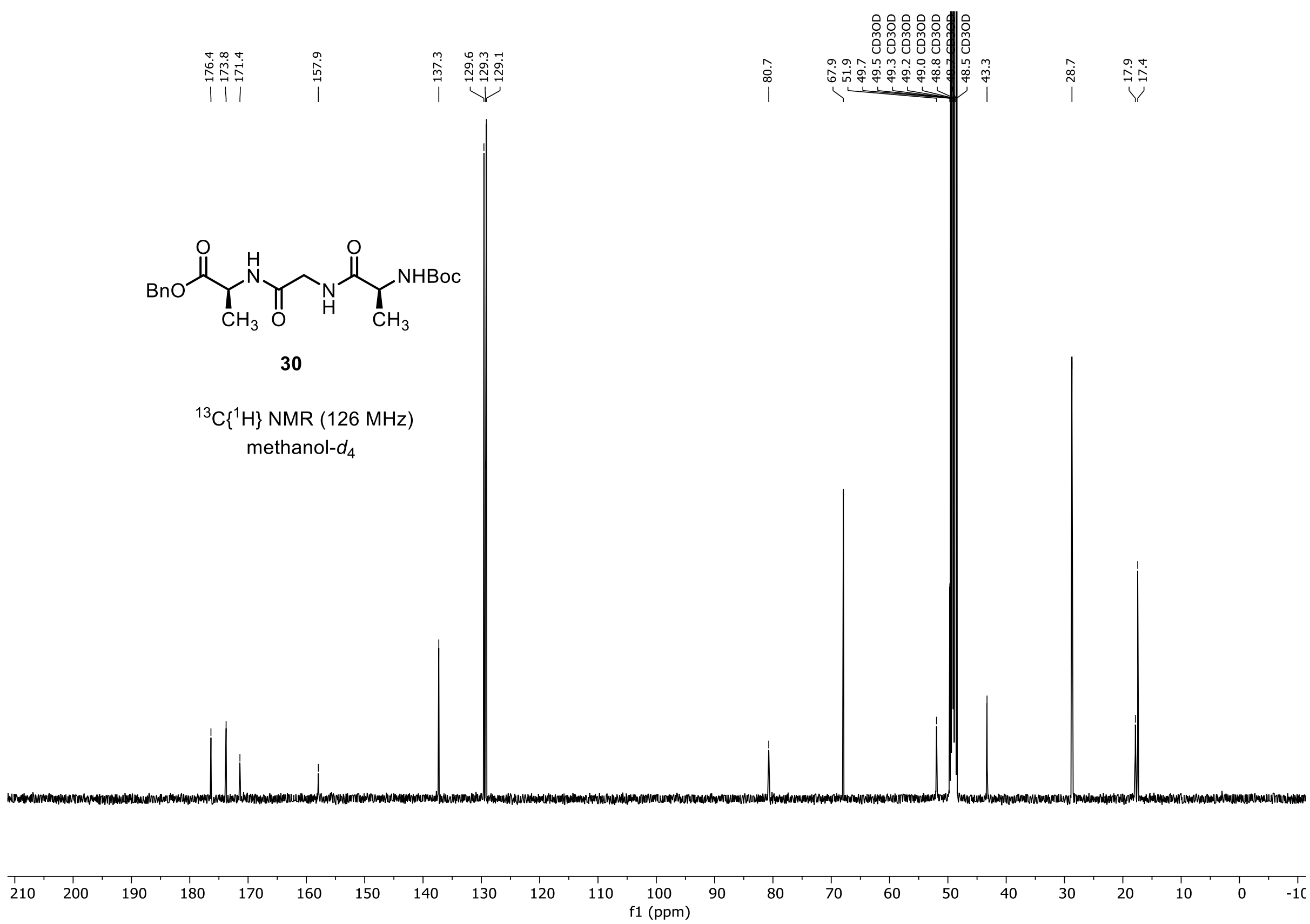

176.4 173.8 171.4 157.9 137.3 129.6 129.3 129.1 80.7 67.9 51.9 49.7 49.5 CD3OD 49.3 CD3OD 49.2 CD3OD 49.0 CD3OD 48.8 CD3OD 48.5 CD3OD 43.3 28.7 17.9 17.4
BnO
NHBoc
CH3
30
13C{1H} NMR (126 MHz)
methanol-d4
f1 (ppm)

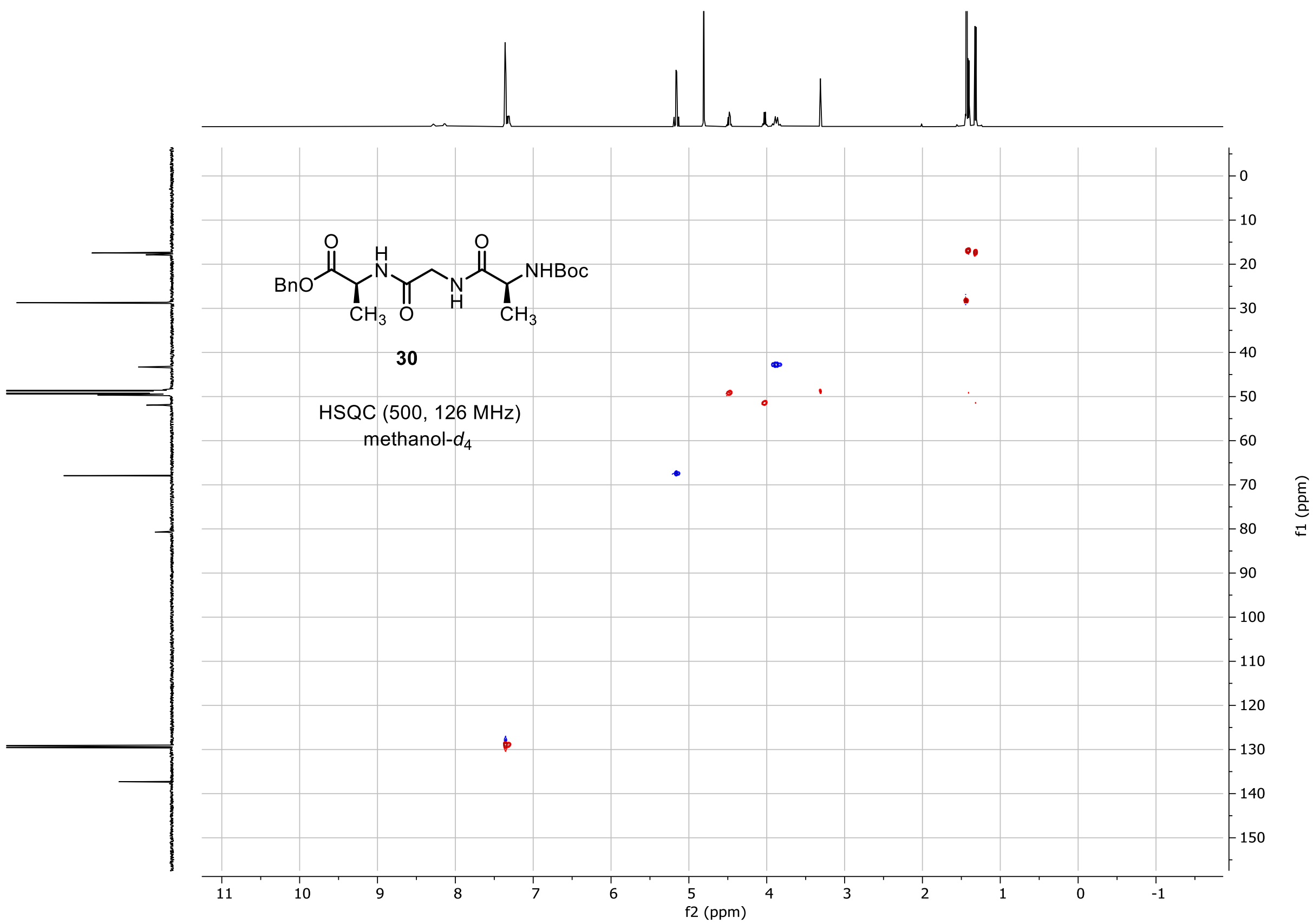
BnO
H
N
NHBoc
N
H
CH3
CH3
O
O
O
30
HSQC (500, 126 MHz)
methanol-d4
f1 (ppm)
f2 (ppm)

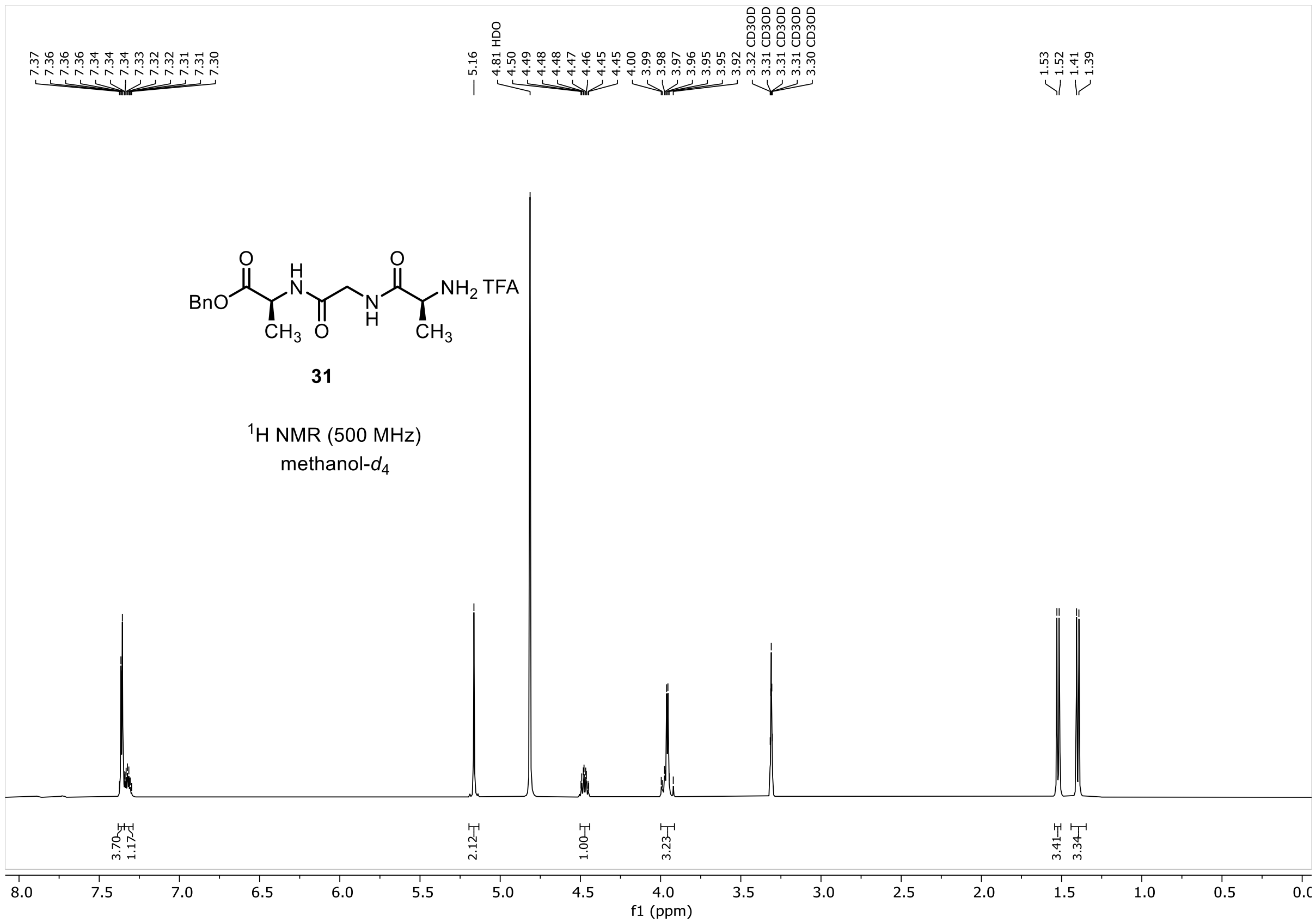
BnO
NH2 TFA
CH3
31
1H NMR (500 MHz)
methanol-d4
f1 (ppm)

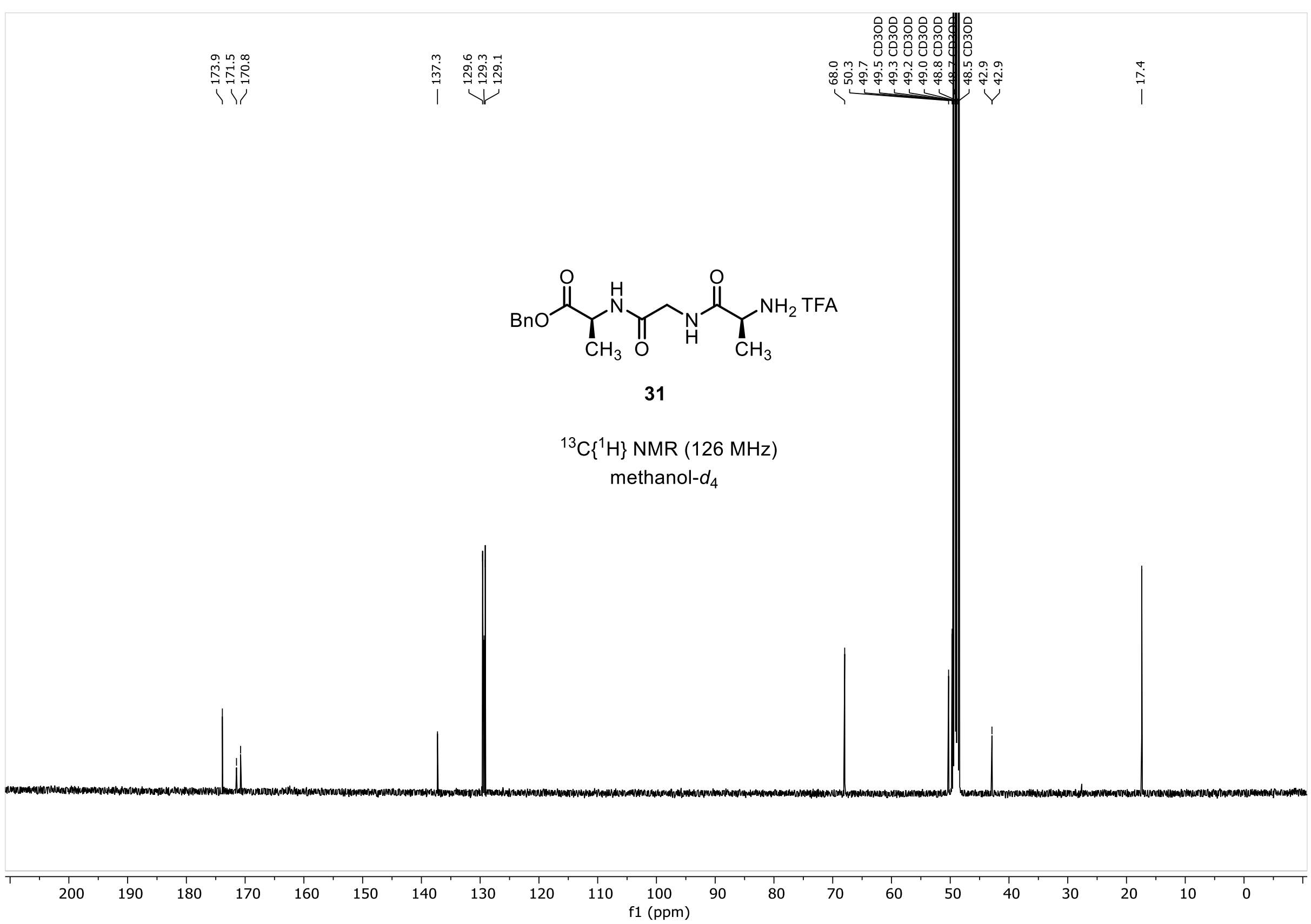
BnO
NH2 TFA
CH3
31
13C{1H} NMR (126 MHz)
methanol-d4
f1 (ppm)

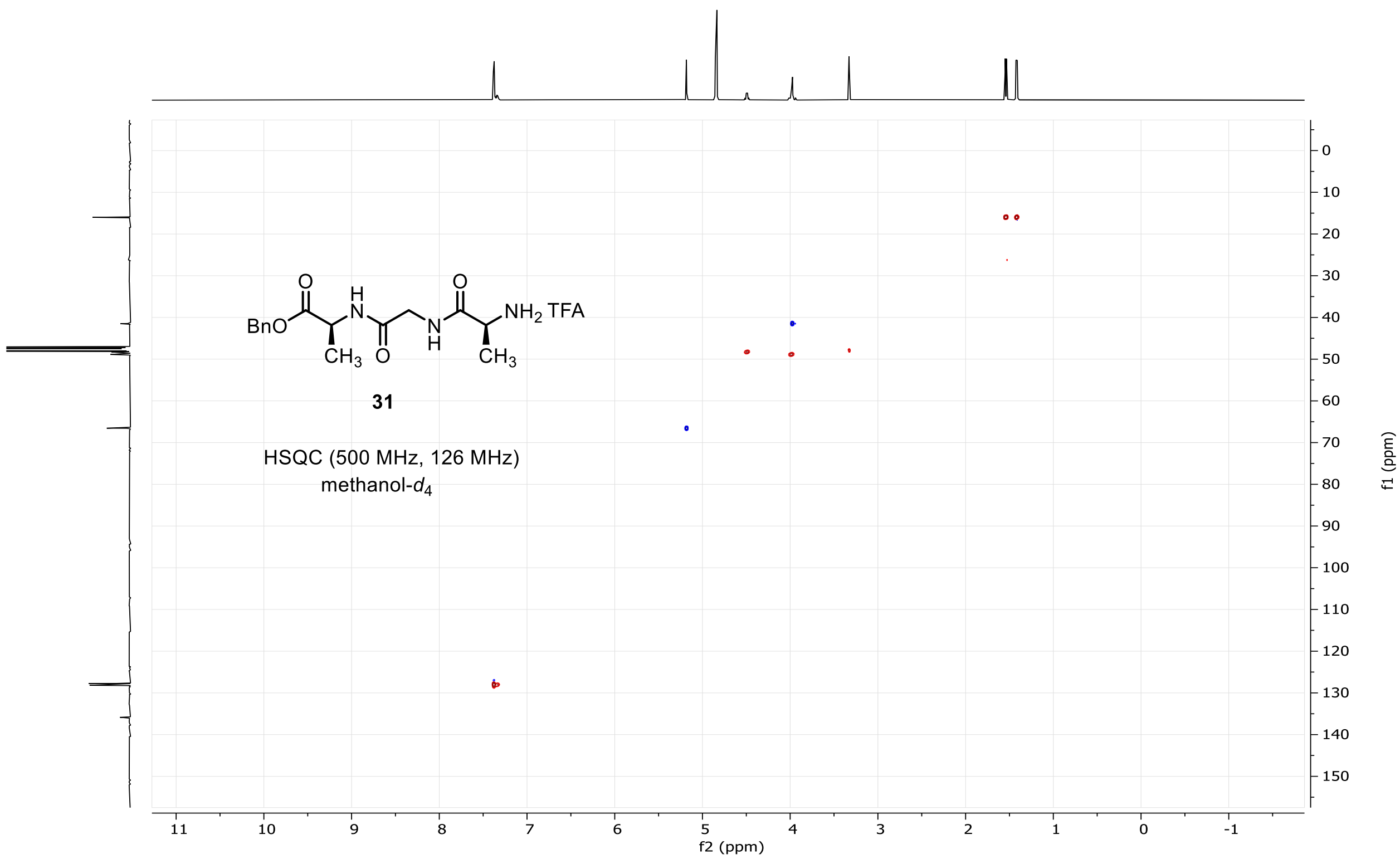
BnO
NH2 TFA
CH3
31
HSQC (500 MHz, 126 MHz)
methanol-d_4
f1 (ppm)
f2 (ppm)

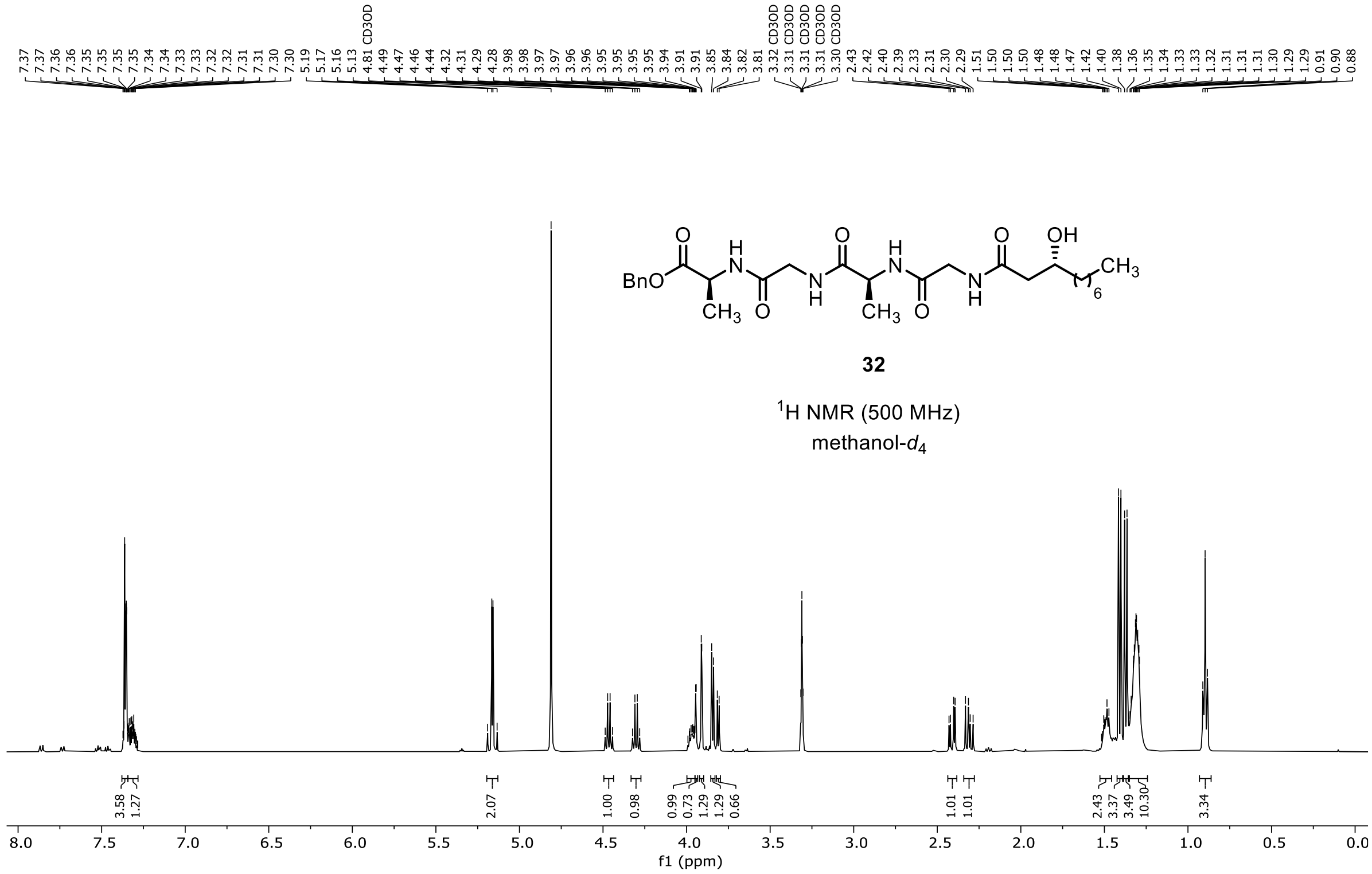
BnO
OH
CH3
32
1H NMR (500 MHz)
methanol-d4
f1 (ppm)

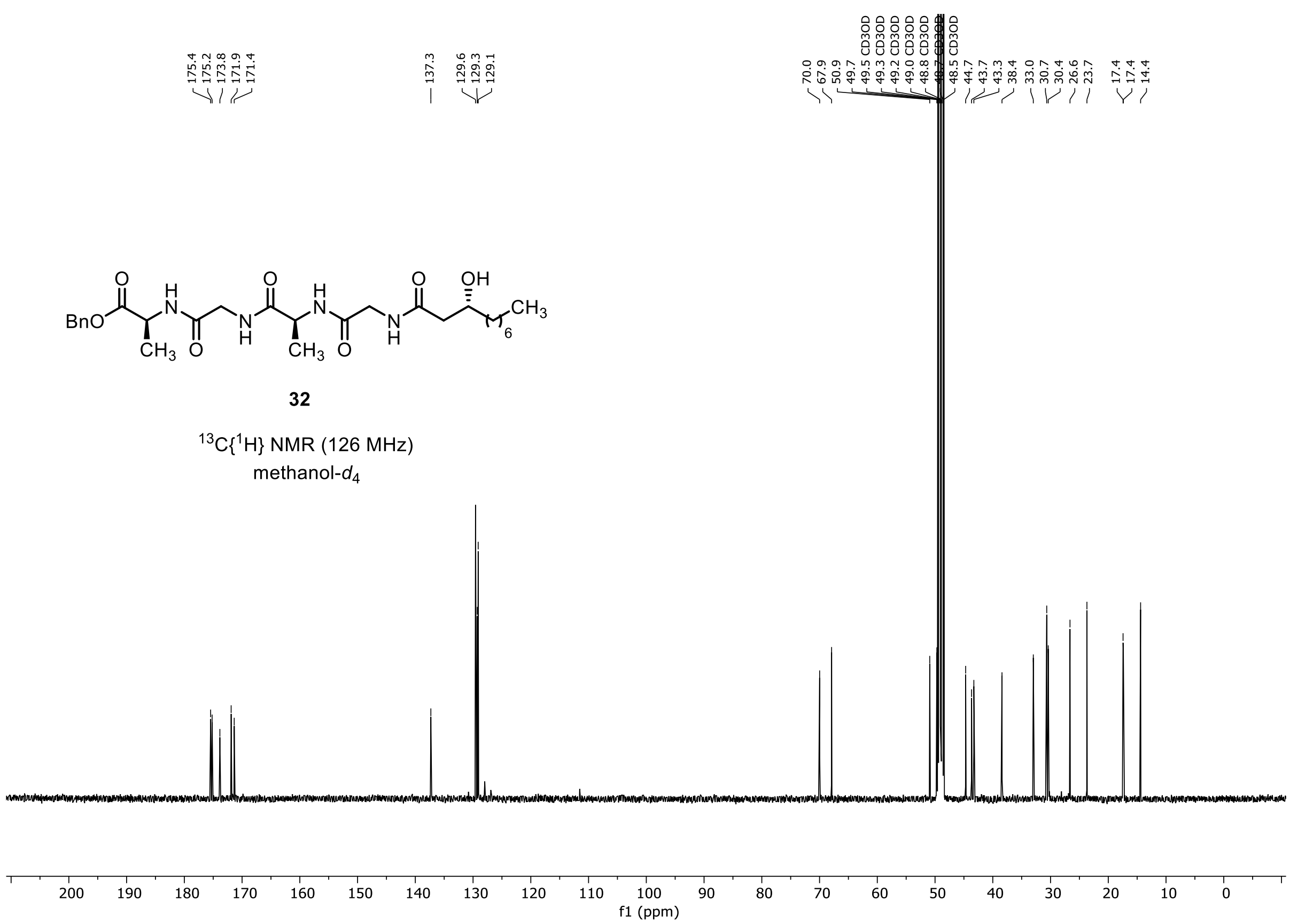
BnO
OH
CH3
32
13C{1H} NMR (126 MHz)
methanol-d4
f1 (ppm)

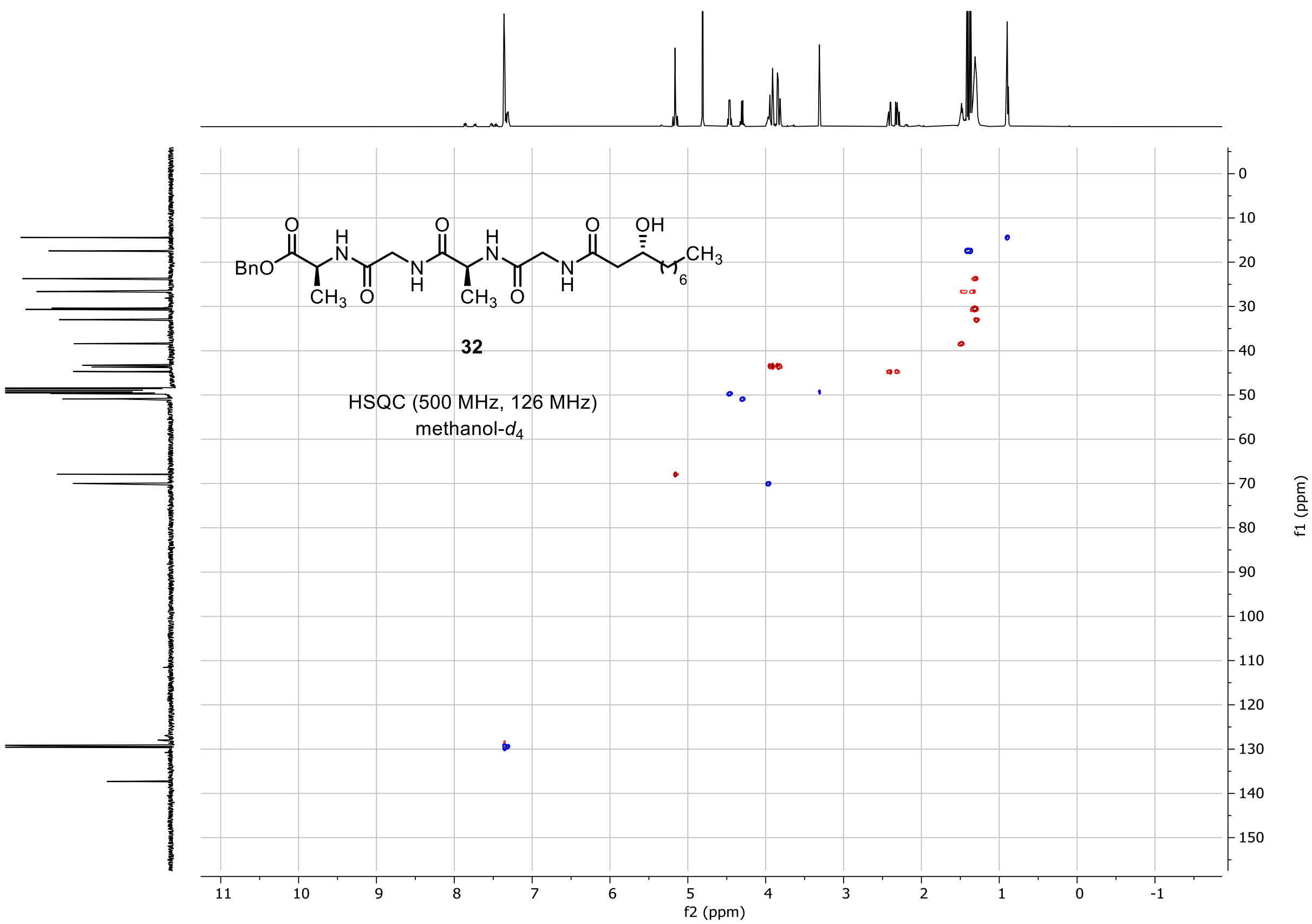
BnO
OH
CH3
32
HSQC (500 MHz, 126 MHz)
methanol-d_4
f1 (ppm)
f2 (ppm)

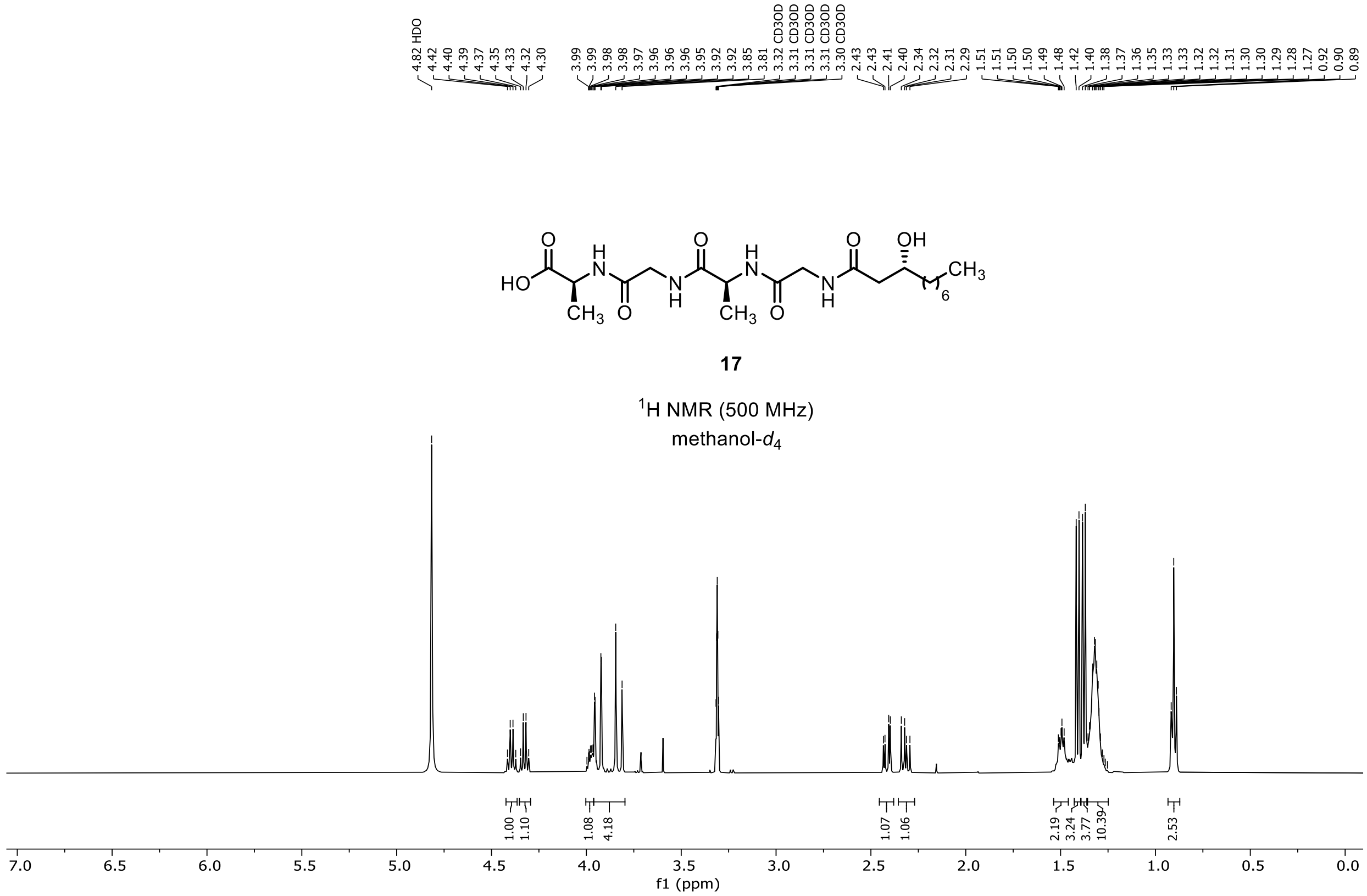
4.82 HDO
4.42 4.40 4.39 4.37 4.35 4.33 4.32 4.30
3.99 3.99 3.98 3.98 3.97 3.96 3.96 3.95 3.92 3.92 3.85 3.81
3.32 CD3OD 3.31 CD3OD 3.31 CD3OD 3.31 CD3OD 3.30 CD3OD
2.43 2.43 2.41 2.40 2.34 2.32 2.31 2.29
1.51 1.51 1.50 1.50 1.49 1.48 1.42 1.40 1.38 1.37 1.36 1.35 1.33 1.33 1.32 1.32 1.31 1.30 1.30 1.29 1.28 1.27
0.92 0.90 0.89
HO
OH
CH3
6
17
1H NMR (500 MHz)
methanol-d4
1.00 1.10 1.08 4.18 1.07 1.06 2.19 3.24 3.77 10.39 2.53
7.0 6.5 6.0 5.5 5.0 4.5 4.0 3.5 3.0 2.5 2.0 1.5 1.0 0.5 0.0
f1 (ppm)

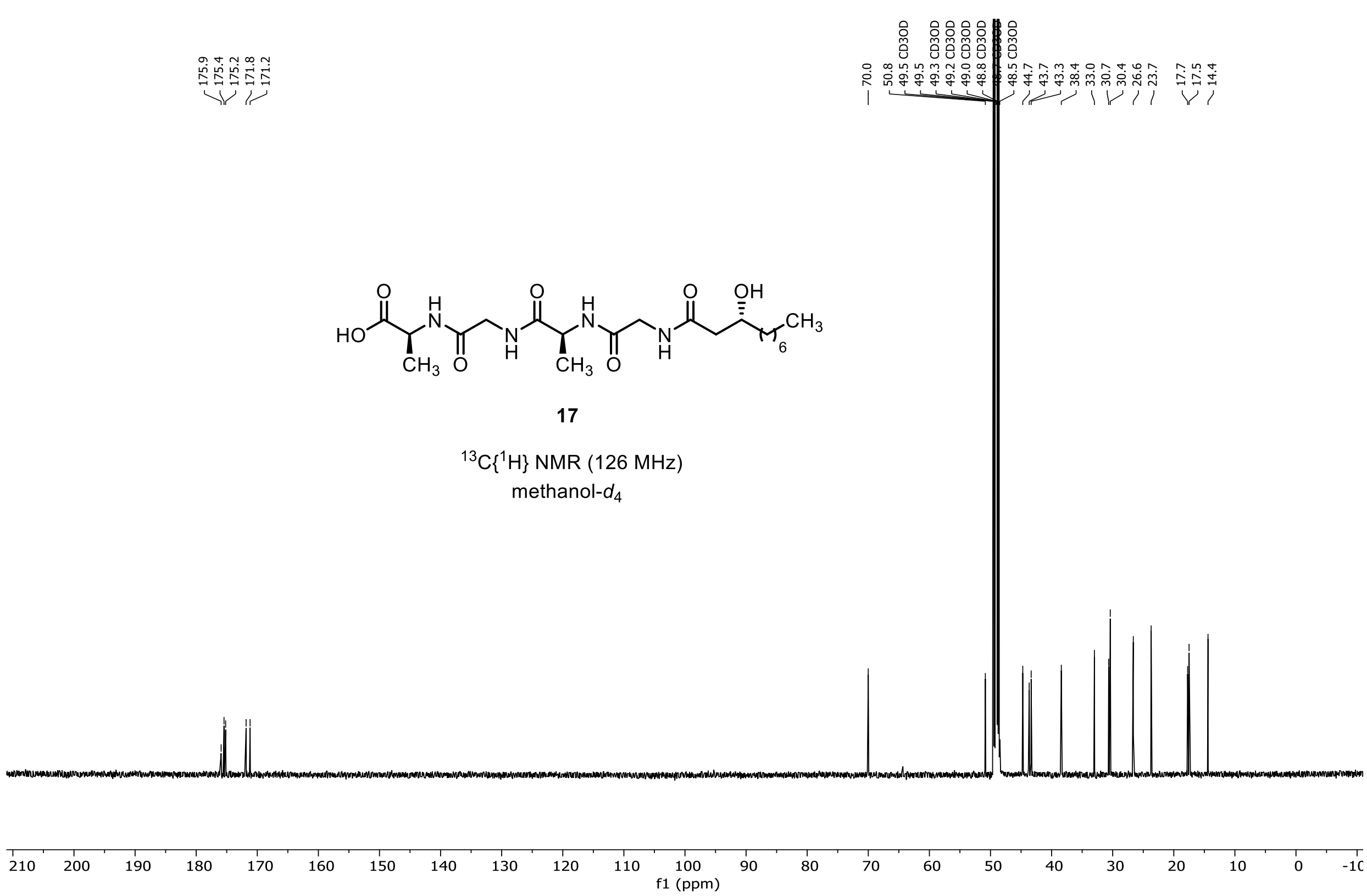
175.9 175.4 175.2 171.8 171.2
70.0
50.8 49.5 CD3OD 49.5 49.3 CD3OD 49.2 CD3OD 49.0 CD3OD 48.8 CD3OD 48.5 CD3OD
44.7 43.7 43.3 38.4 33.0 30.7 30.4 26.6 23.7 17.7 17.5 14.4
HO
OH
CH3
6
17
13C{1H} NMR (126 MHz)
methanol-d4
210 200 190 180 170 160 150 140 130 120 110 100 90 80 70 60 50 40 30 20 10 0 -10
f1 (ppm)

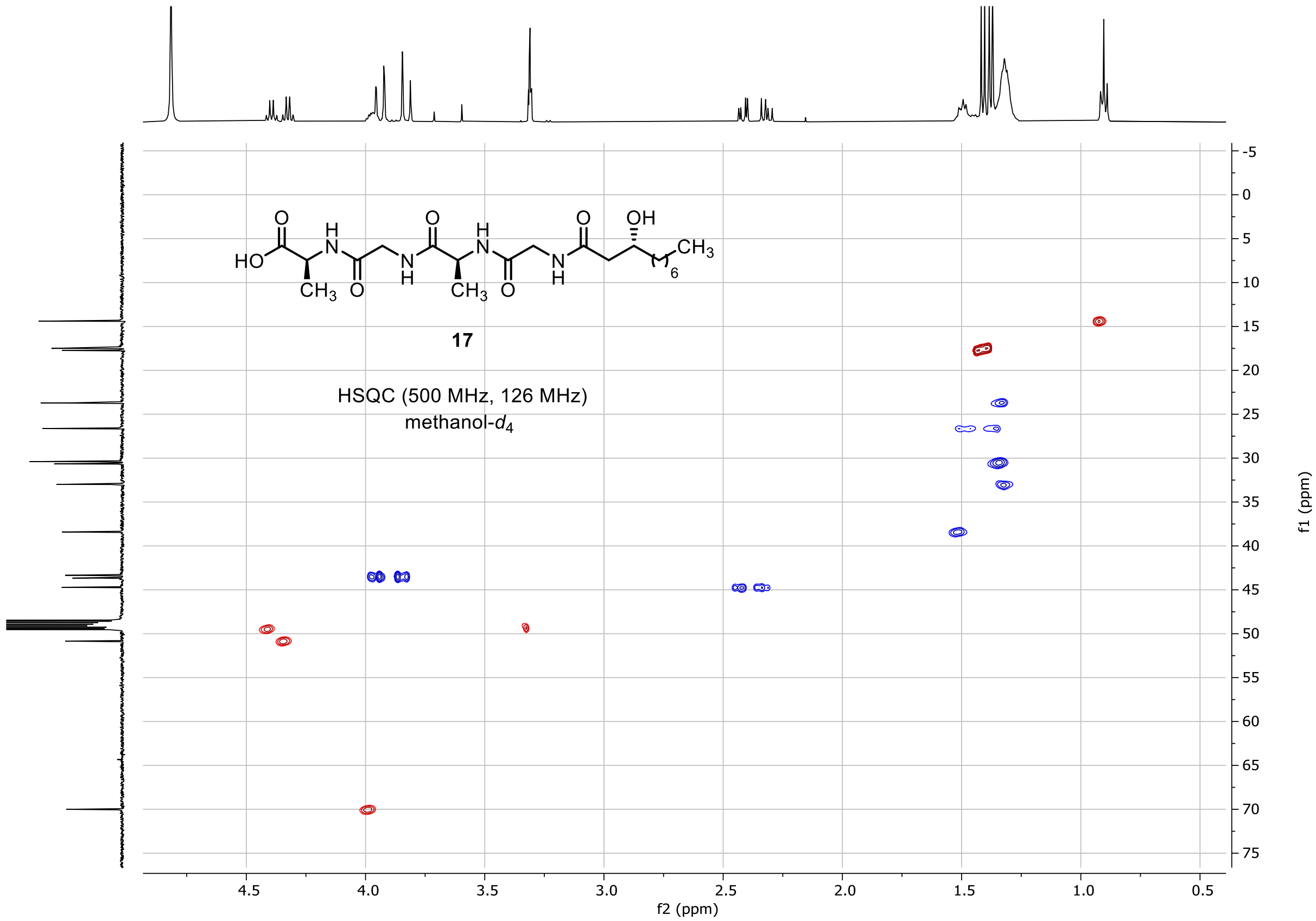
17
HSQC (500 MHz, 126 MHz)
methanol-d_4
f2 (ppm)
f1 (ppm)

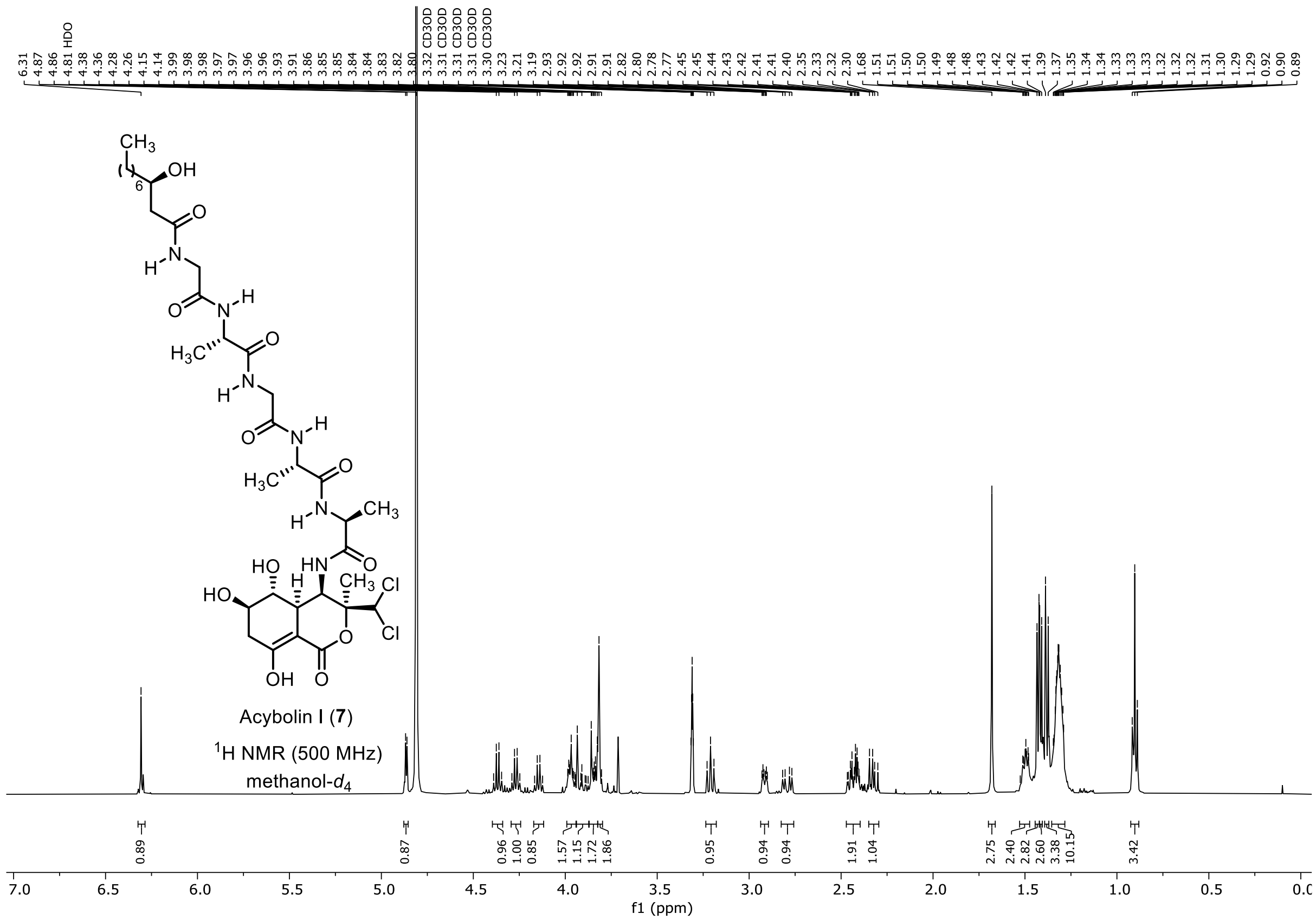
Acybolin I (**7**)
^1H NMR (500 MHz)
methanol-d_4
f1 (ppm)

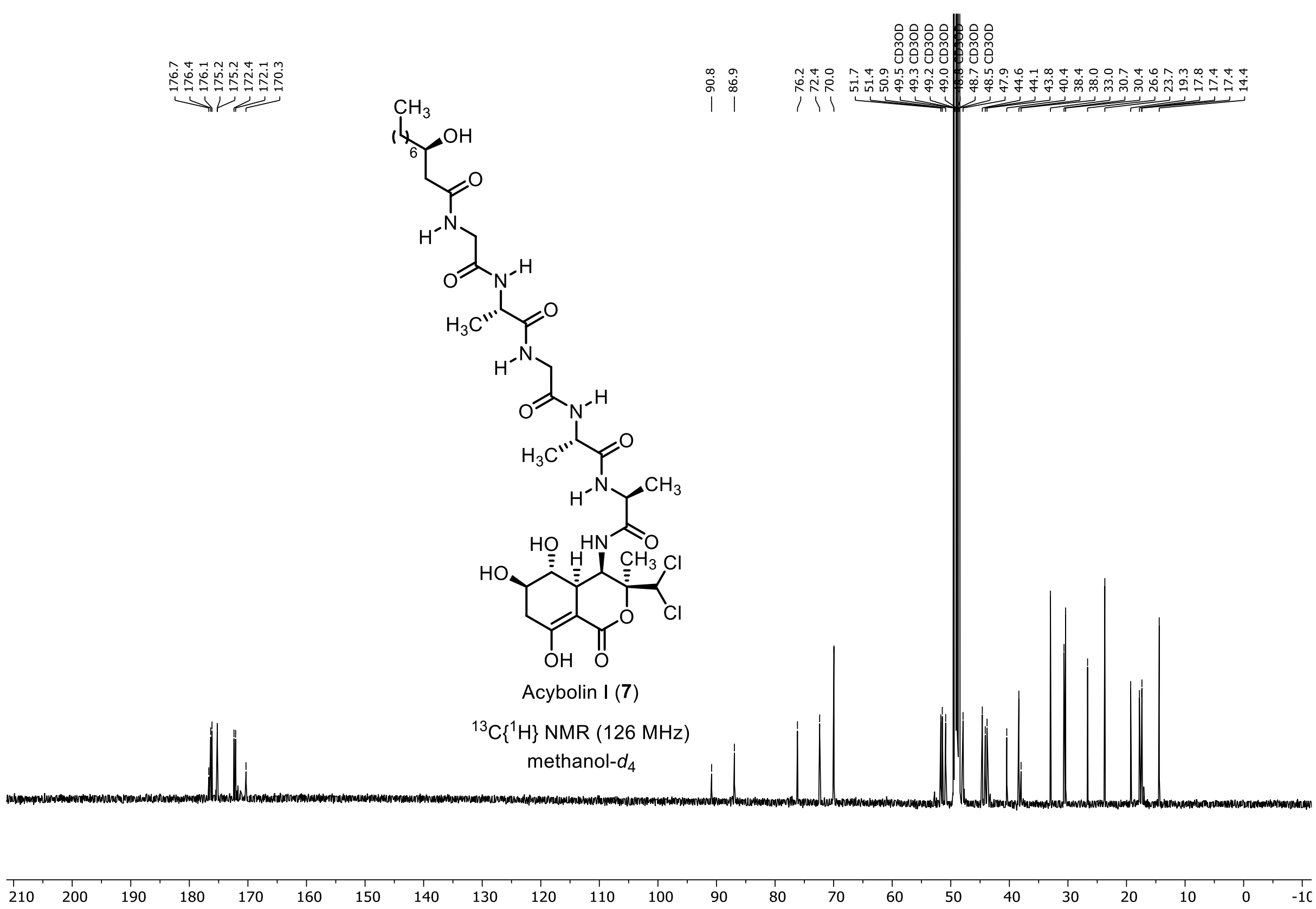
Acybolin I (**7**)
$^{13}C\{^1H\}$ NMR (126 MHz)
methanol-d_4
f1 (ppm)

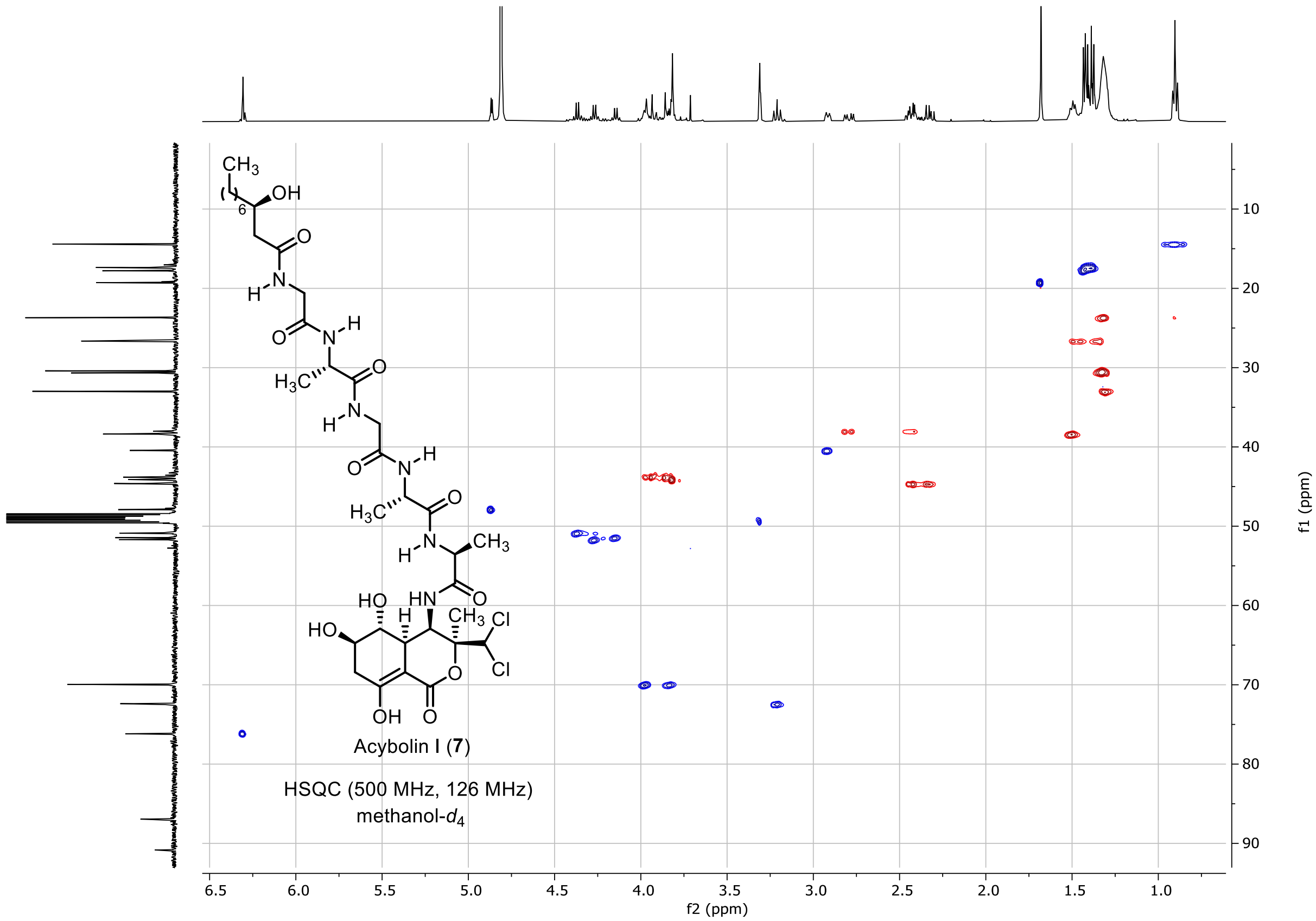

Acybolin I (7)
HSQC (500 MHz, 126 MHz)
methanol-d_4
f2 (ppm)
f1 (ppm)

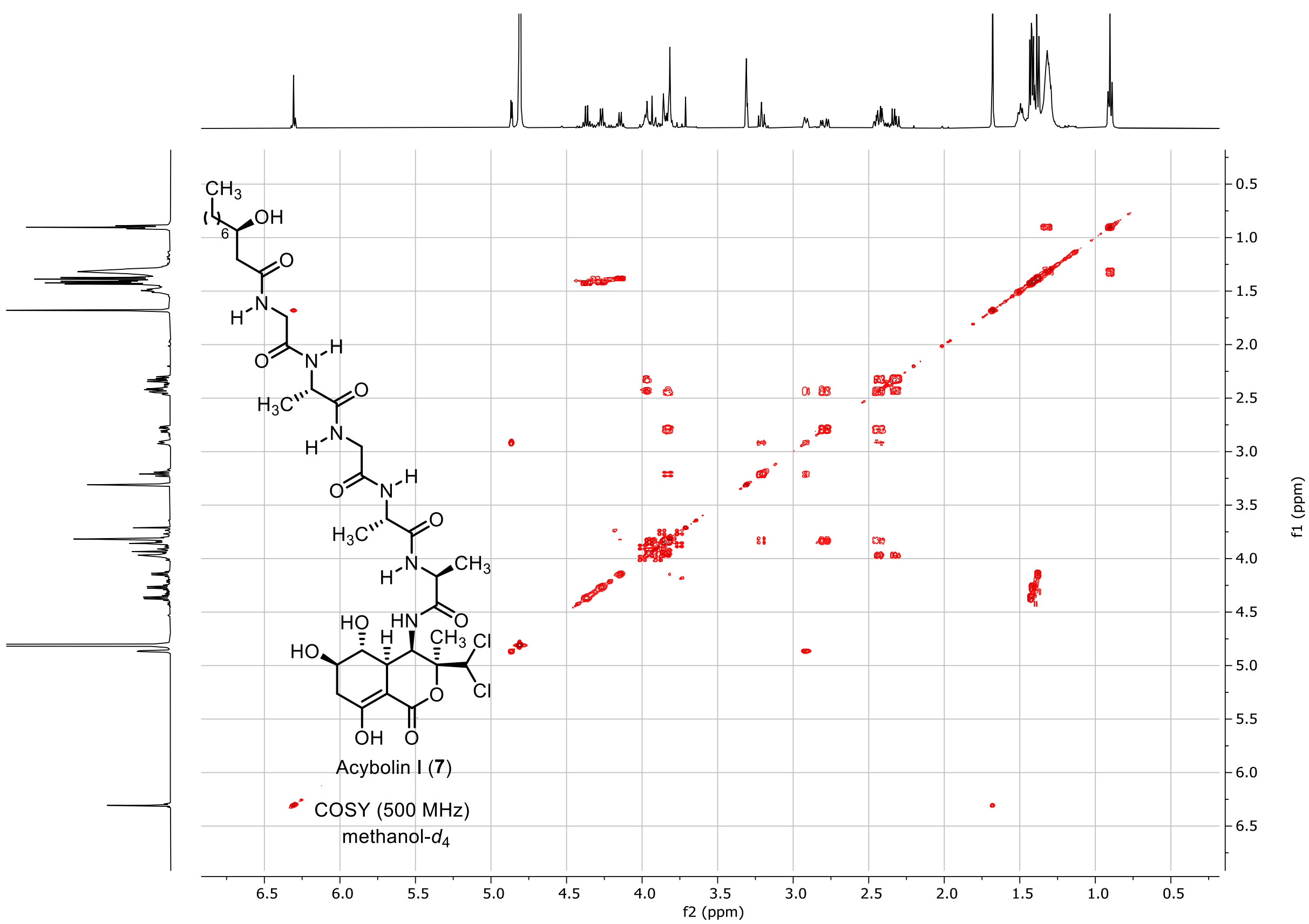

Acybolin I (7)
COSY (500 MHz)
methanol-d_4
f2 (ppm)
f1 (ppm)

NMR SPECTRA OF *EPI*-ACYBOLINS

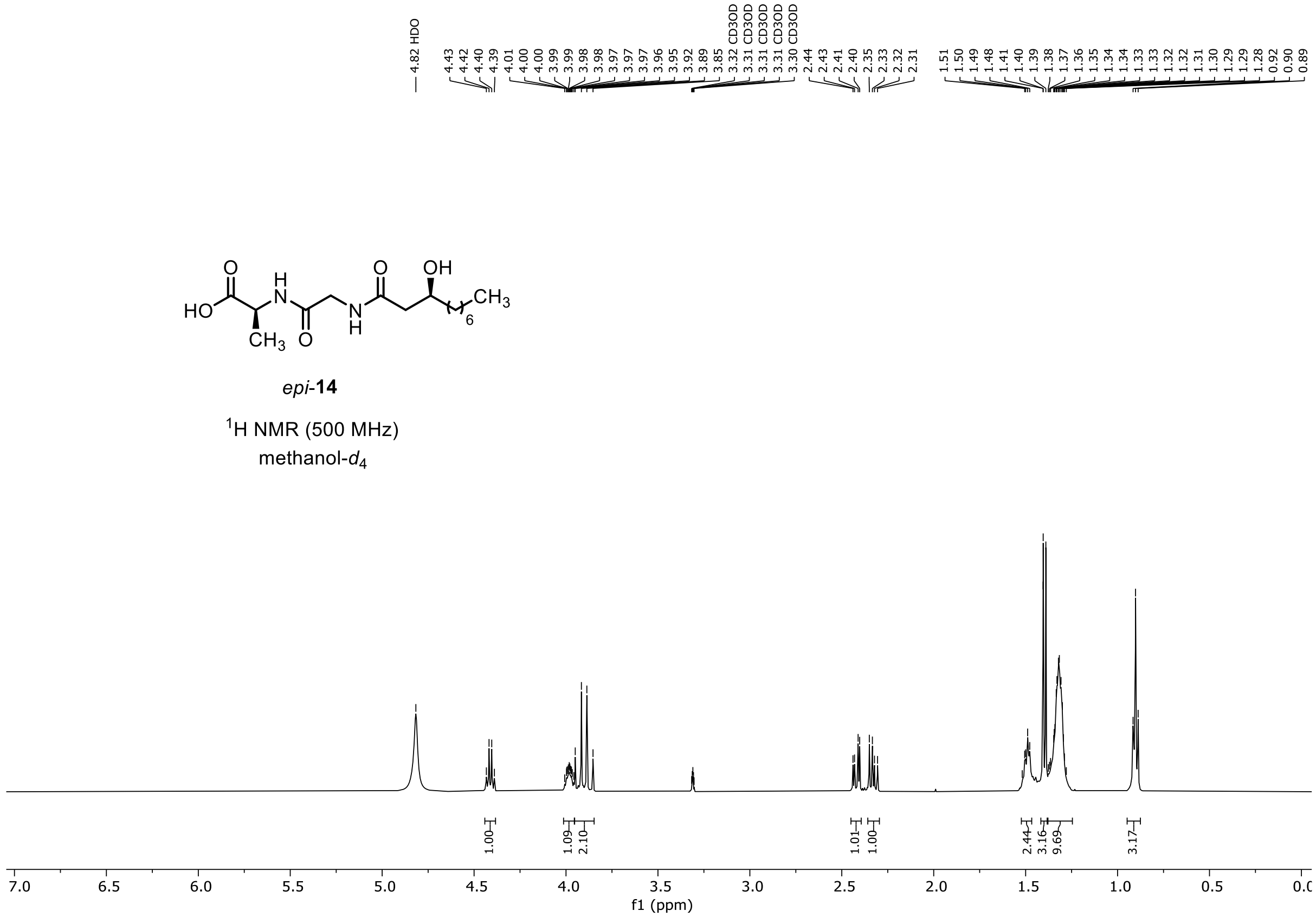
4.82 HDO
4.43 4.42 4.40 4.39 4.01 4.00 4.00 3.99 3.99 3.98 3.98 3.97 3.97 3.96 3.95 3.92 3.89 3.85
3.32 CD3OD 3.31 CD3OD 3.31 CD3OD 3.31 CD3OD 3.30 CD3OD
2.44 2.43 2.41 2.40 2.35 2.33 2.32 2.31
1.51 1.50 1.49 1.48 1.41 1.40 1.39 1.38 1.37 1.36 1.35 1.34 1.34 1.33 1.33 1.32 1.32 1.31 1.30 1.29 1.29 1.28 0.92 0.90 0.89
HO OH CH3 N H N H O O O CH3 6
epi-14
1H NMR (500 MHz)
methanol-d4
1.00 1.09 2.10 1.01 1.00 2.44 3.16 9.69 3.17
7.0 6.5 6.0 5.5 5.0 4.5 4.0 3.5 3.0 2.5 2.0 1.5 1.0 0.5 0.0
f1 (ppm)

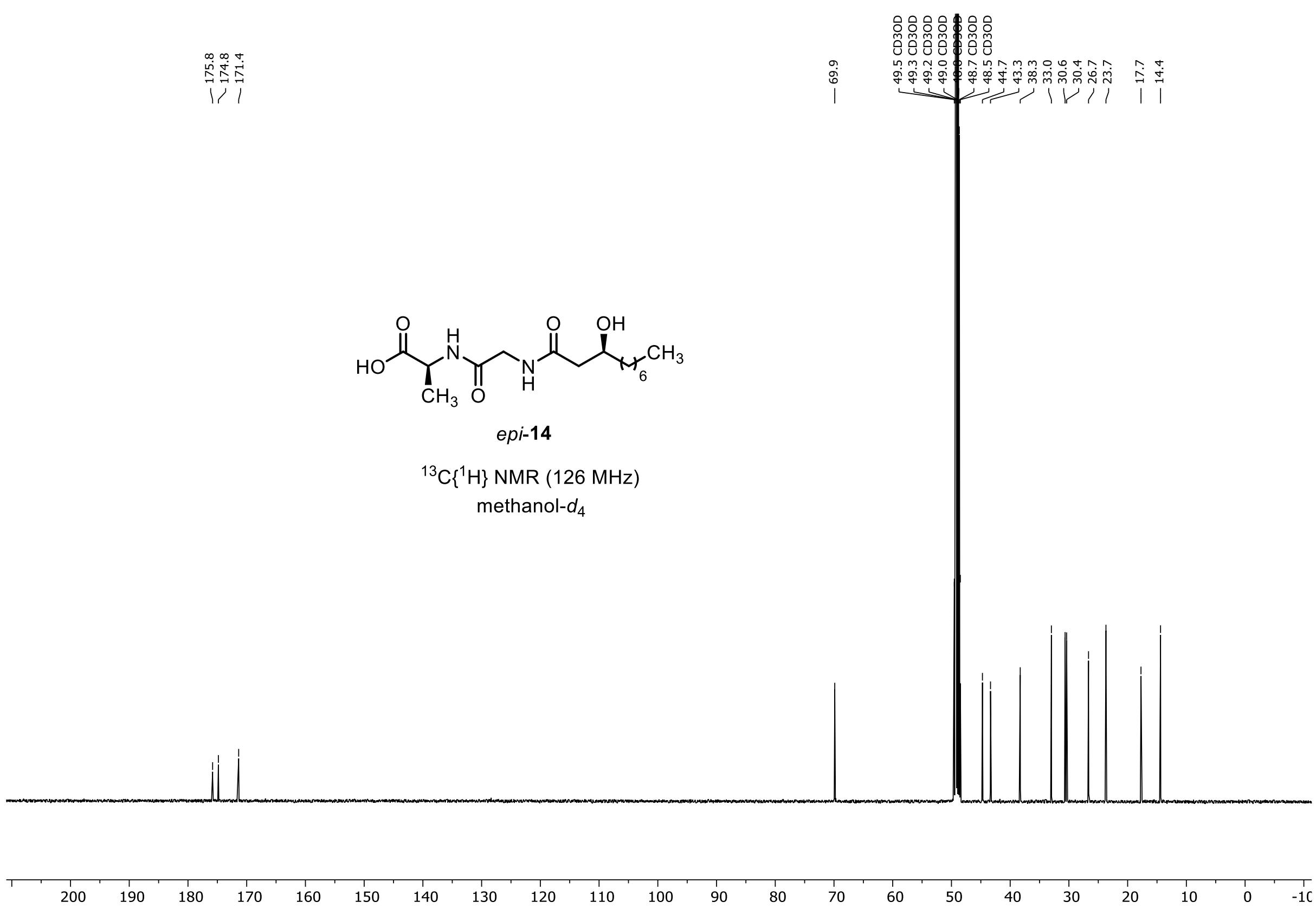
175.8 174.8 171.4
69.9
49.5 CD3OD 49.3 CD3OD 49.2 CD3OD 49.0 CD3OD 48.7 CD3OD 48.5 CD3OD
44.7 43.3 38.3 33.0 30.6 30.4 26.7 23.7 17.7 14.4
HO OH CH3 N H N H O O O CH3 6
epi-14
13C{1H} NMR (126 MHz)
methanol-d4
200 190 180 170 160 150 140 130 120 110 100 90 80 70 60 50 40 30 20 10 0 -10
f1 (ppm)

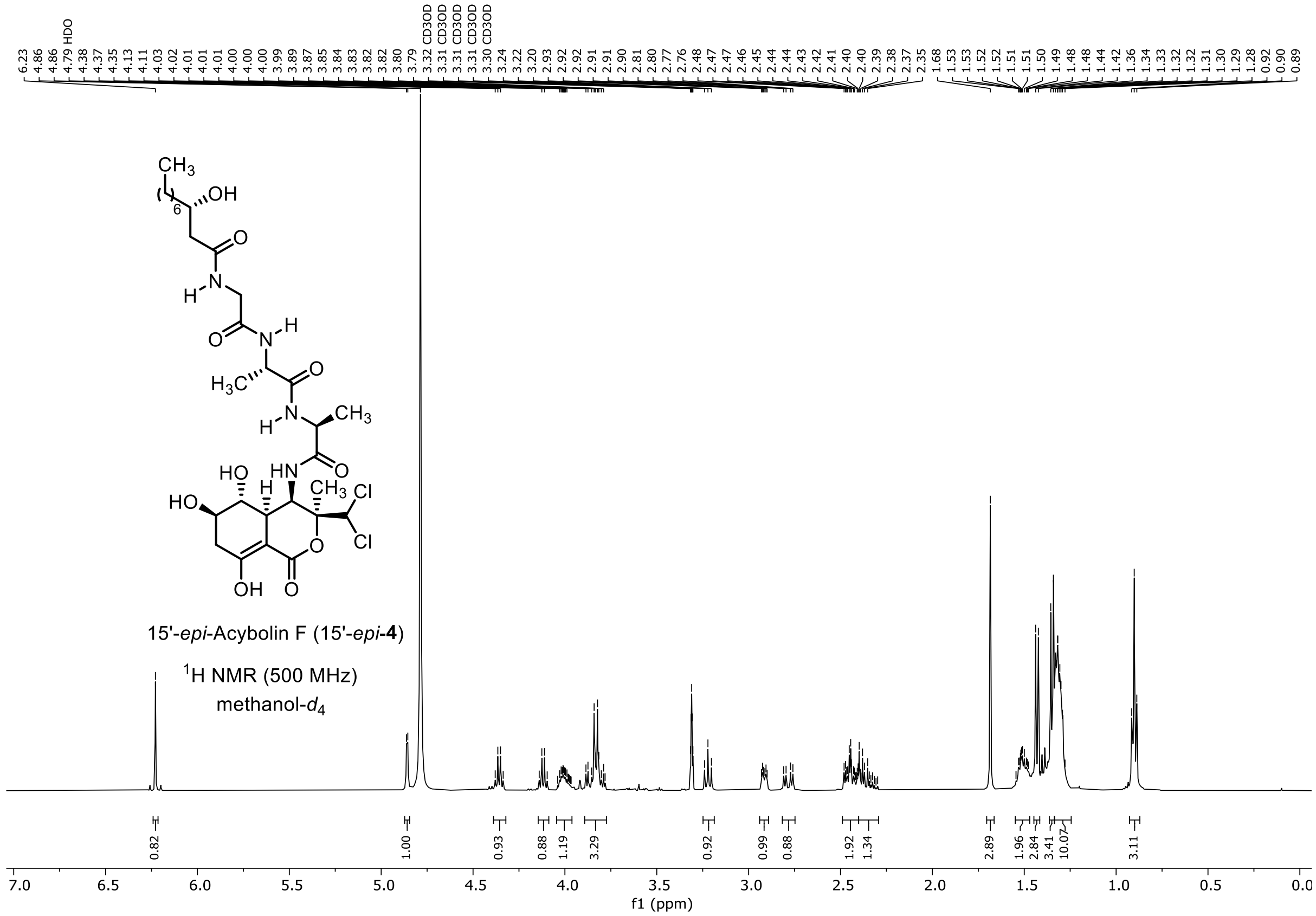

15'-epi-Acybolin F (15'-epi-4)
1H NMR (500 MHz)
methanol-d4
f1 (ppm)

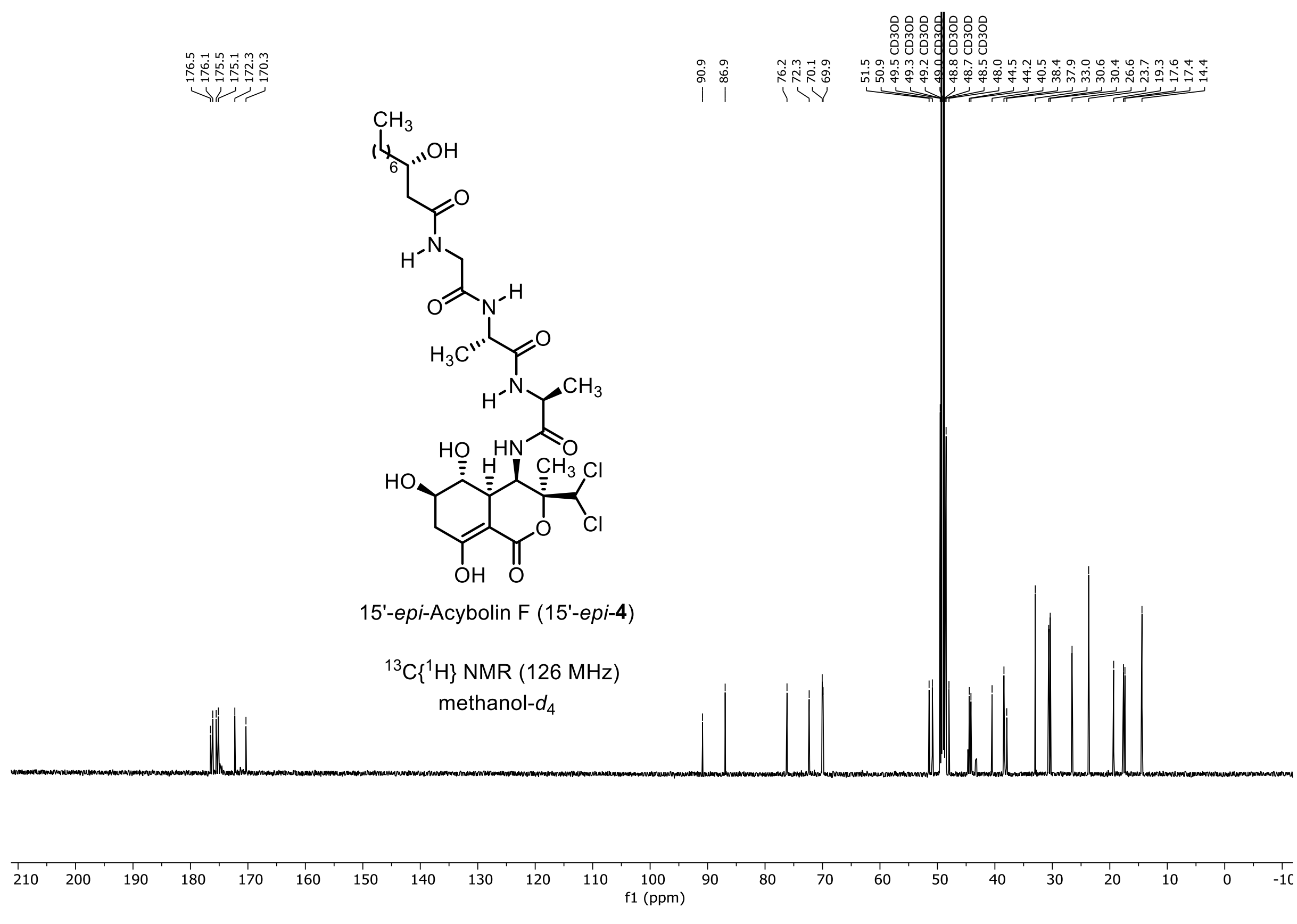

176.5
176.1
175.5
175.1
172.3
170.3
90.9
86.9
76.2
72.3
70.1
69.9
51.5
50.9
49.5
49.3
49.2
49.0
48.8
48.7
48.5
48.0
44.5
44.2
40.5
38.4
37.9
33.0
30.6
30.4
26.6
23.7
19.3
17.6
17.4
14.4
15'-epi-Acybolin F (15'-epi-4)
13C{1H} NMR (126 MHz)
methanol-d4
f1 (ppm)

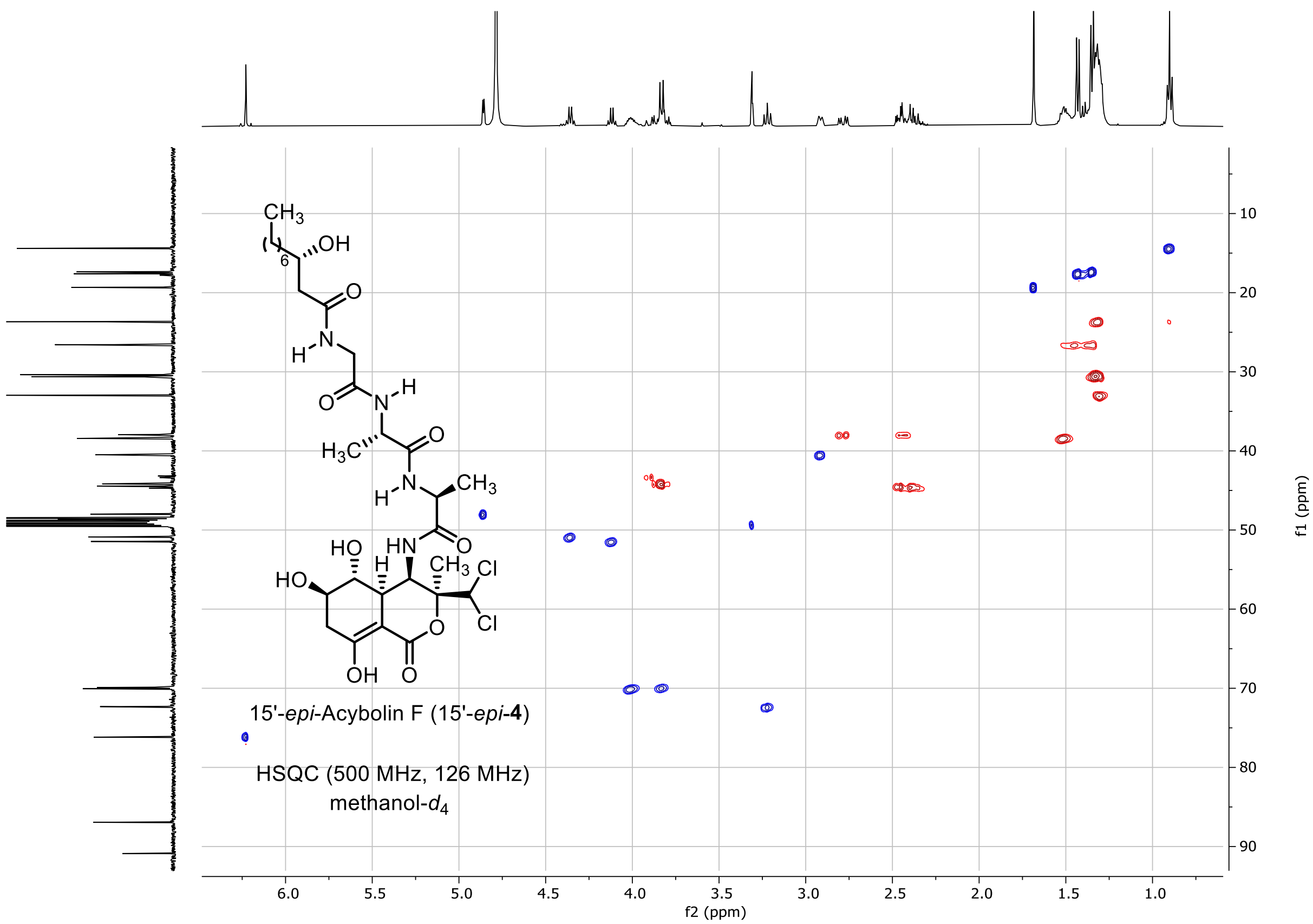
15'-*epi*-Acybolin F (15'-*epi*-**4**)
HSQC (500 MHz, 126 MHz)
methanol-d_4
f2 (ppm)
f1 (ppm)

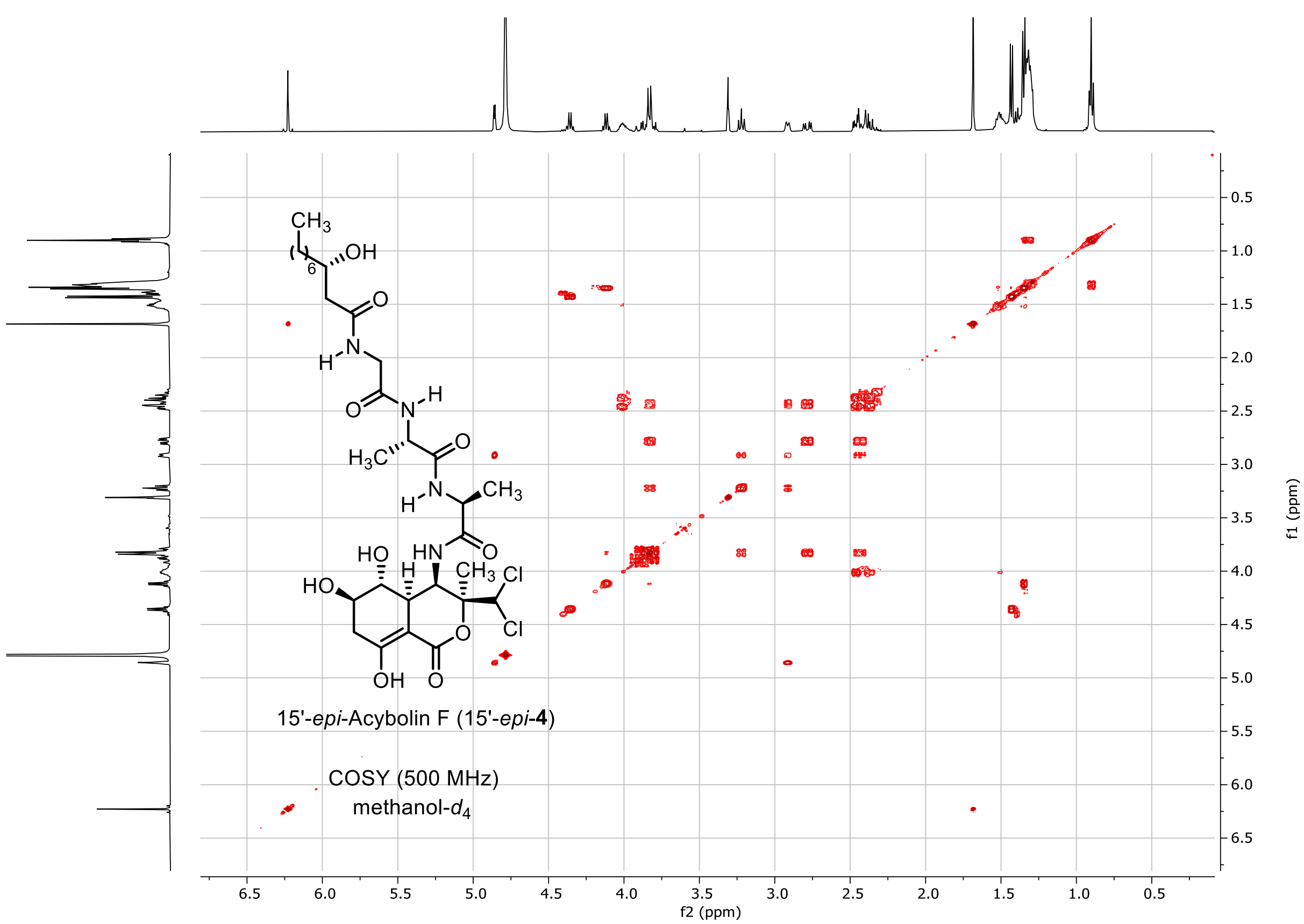
15'-*epi*-Acybolin F (15'-*epi*-**4**)
COSY (500 MHz)
methanol-d_4
f2 (ppm)
f1 (ppm)

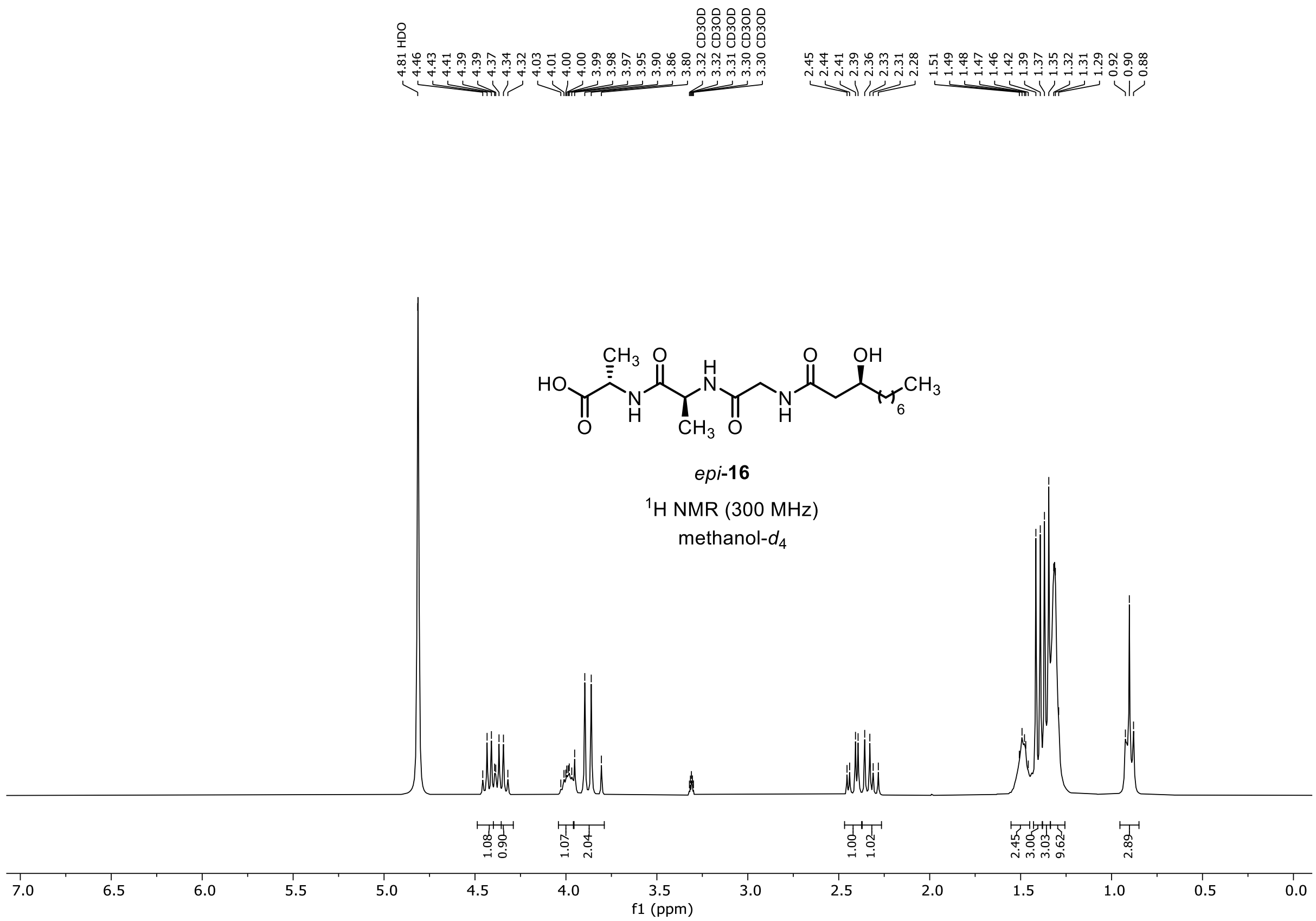
4.81 HDO
4.46 4.43 4.41 4.39 4.39 4.37 4.34 4.32
4.03 4.01 4.00 4.00 3.99 3.98 3.97 3.95 3.90 3.86 3.80
3.32 CD3OD 3.32 CD3OD 3.31 CD3OD 3.30 CD3OD 3.30 CD3OD
2.45 2.44 2.41 2.39 2.36 2.33 2.31 2.28
1.51 1.49 1.48 1.47 1.46 1.42 1.39 1.37 1.35 1.32 1.31 1.29
0.92 0.90 0.88
epi-16
1H NMR (300 MHz)
methanol-d4
1.08 0.90 1.07 2.04 1.00 1.02 2.45 3.00 3.03 9.62 2.89
f1 (ppm)

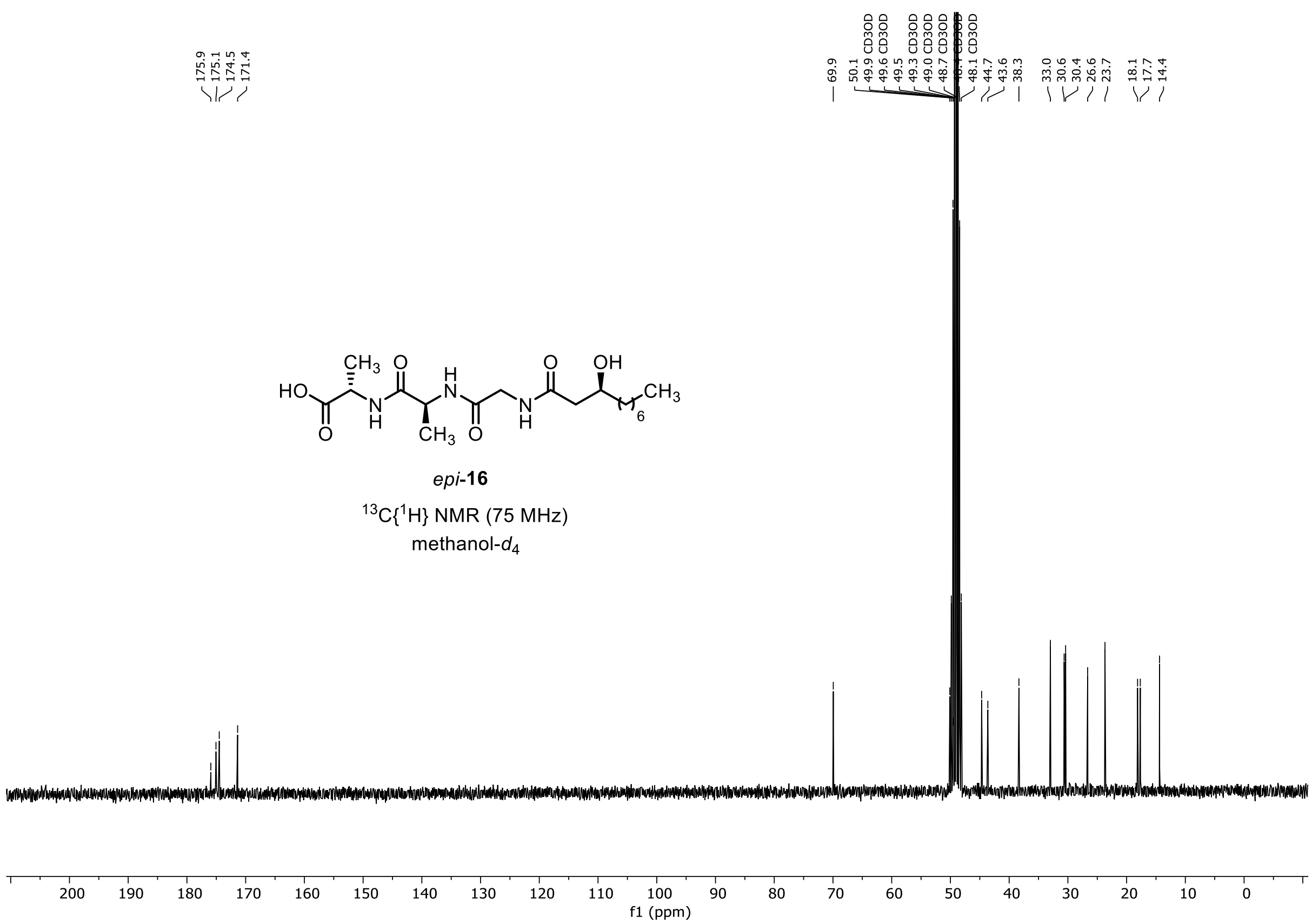
175.9 175.1 174.5 171.4
69.9
50.1 49.9 CD3OD 49.6 CD3OD 49.5 49.3 CD3OD 49.0 CD3OD 48.7 CD3OD 48.1 CD3OD
44.7 43.6 38.3 33.0 30.6 30.4 26.6 23.7 18.1 17.7 14.4
epi-16
13C{1H} NMR (75 MHz)
methanol-d4
f1 (ppm)

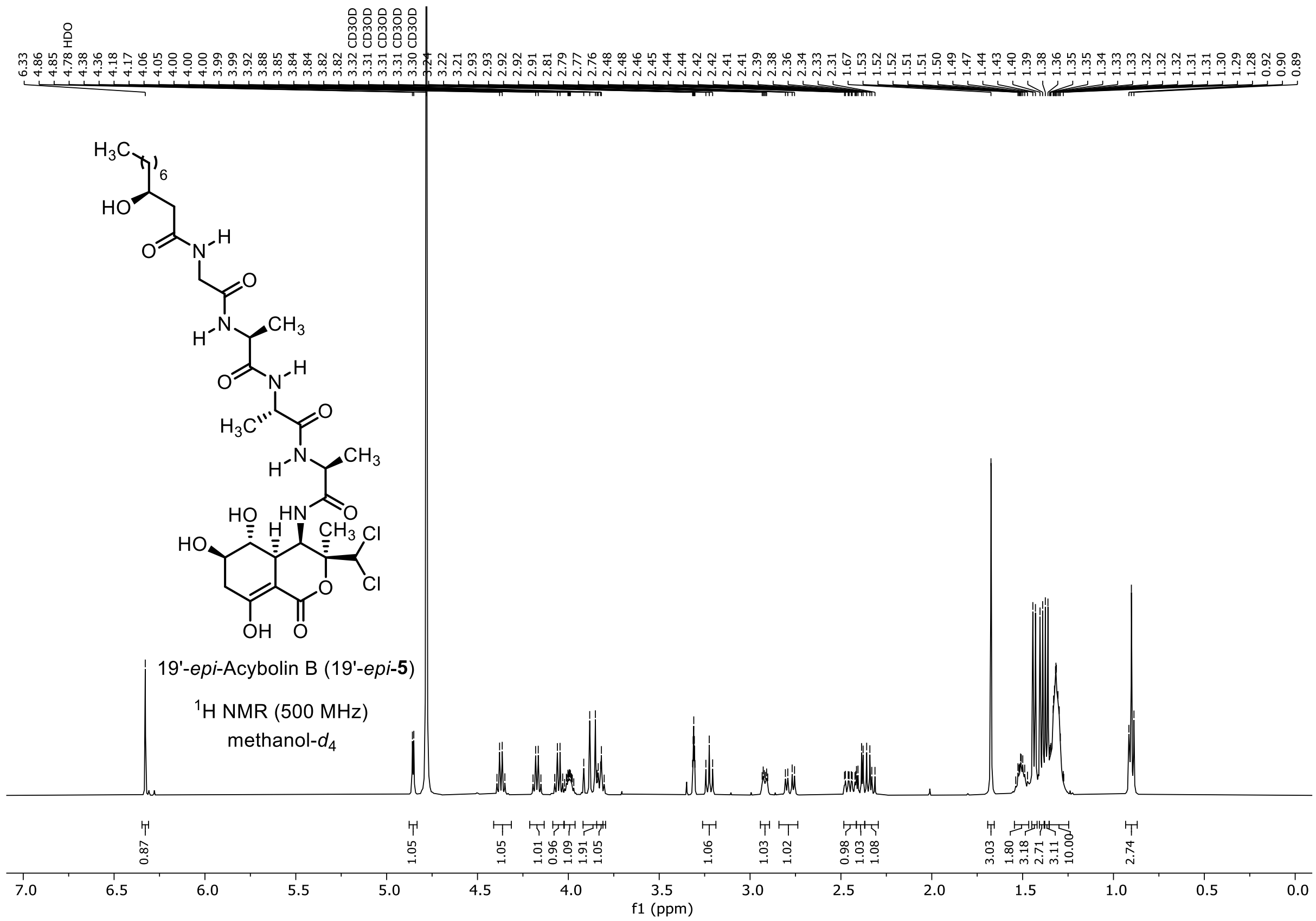
6.33
4.86
4.85
4.78 HDO
4.38
4.36
4.18
4.17
4.06
4.05
4.00
4.00
4.00
3.99
3.99
3.92
3.88
3.85
3.84
3.84
3.82
3.82
3.32 CD3OD
3.31 CD3OD
3.31 CD3OD
3.31 CD3OD
3.30 CD3OD
3.24
3.22
3.21
2.93
2.93
2.92
2.92
2.91
2.81
2.79
2.77
2.76
2.48
2.46
2.45
2.44
2.44
2.42
2.42
2.41
2.41
2.39
2.38
2.36
2.34
2.33
2.31
1.67
1.53
1.52
1.52
1.51
1.51
1.50
1.49
1.47
1.44
1.43
1.40
1.39
1.38
1.36
1.35
1.35
1.34
1.33
1.33
1.32
1.32
1.32
1.31
1.31
1.30
1.29
1.28
0.92
0.90
0.89
19'-epi-Acybolin B (19'-epi-5)
1H NMR (500 MHz)
methanol-d4
0.87
1.05
1.05
1.01
0.96
1.09
1.91
1.05
1.06
1.03
1.02
0.98
1.03
1.08
3.03
1.80
3.18
2.71
3.11
10.00
2.74
7.0
6.5
6.0
5.5
5.0
4.5
4.0
3.5
3.0
2.5
2.0
1.5
1.0
0.5
0.0
f1 (ppm)

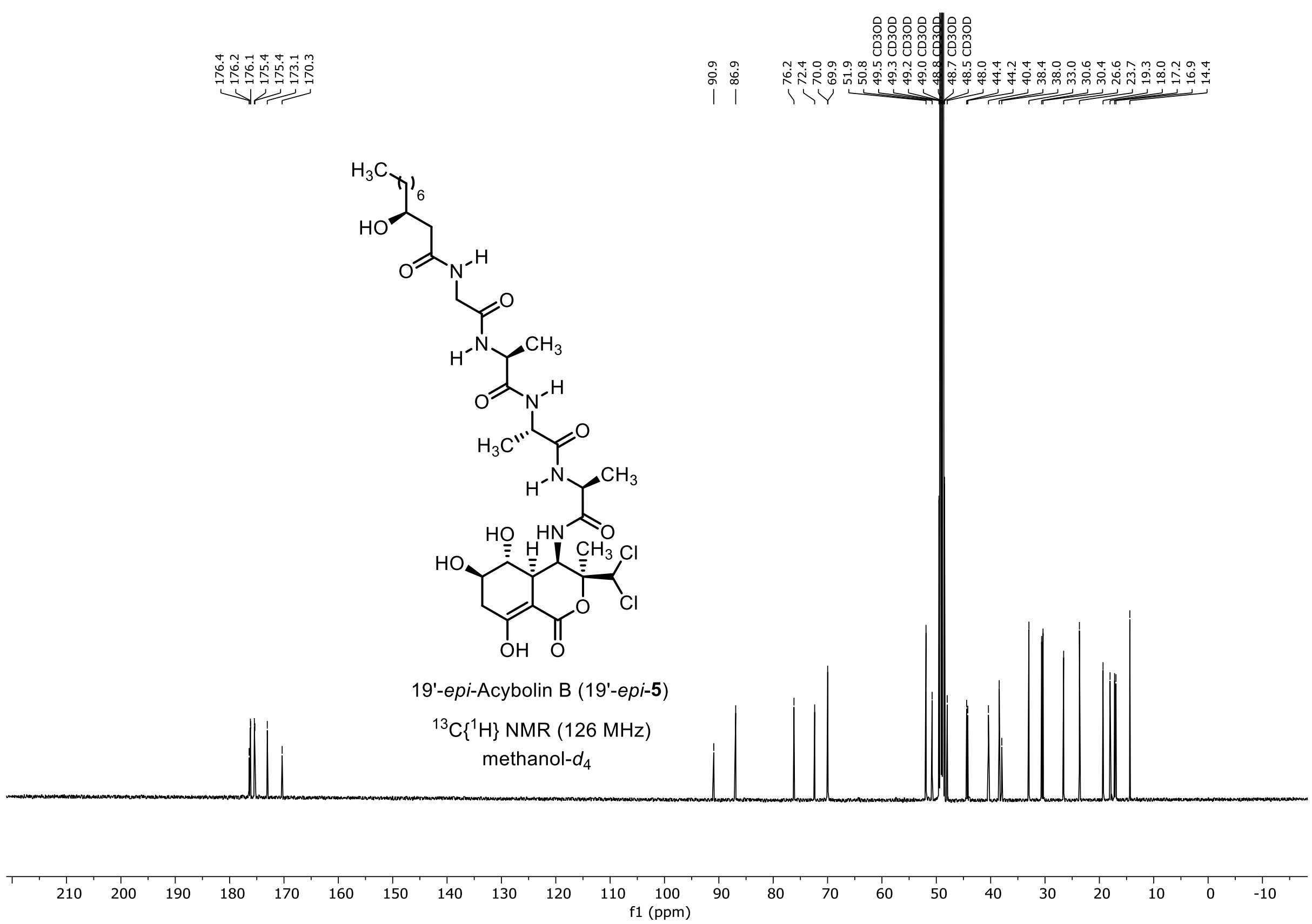
176.4
176.2
176.1
175.4
175.4
173.1
170.3
90.9
86.9
76.2
72.4
70.0
69.9
51.9
50.8
49.5 CD3OD
49.3 CD3OD
49.2 CD3OD
49.0 CD3OD
48.8 CD3OD
48.7 CD3OD
48.5 CD3OD
48.0
44.4
44.2
40.4
38.4
38.0
33.0
30.6
30.4
26.6
23.7
19.3
18.0
17.2
16.9
14.4
19'-epi-Acybolin B (19'-epi-5)
13C{1H} NMR (126 MHz)
methanol-d4
210
200
190
180
170
160
150
140
130
120
110
100
90
80
70
60
50
40
30
20
10
0
-10
f1 (ppm)

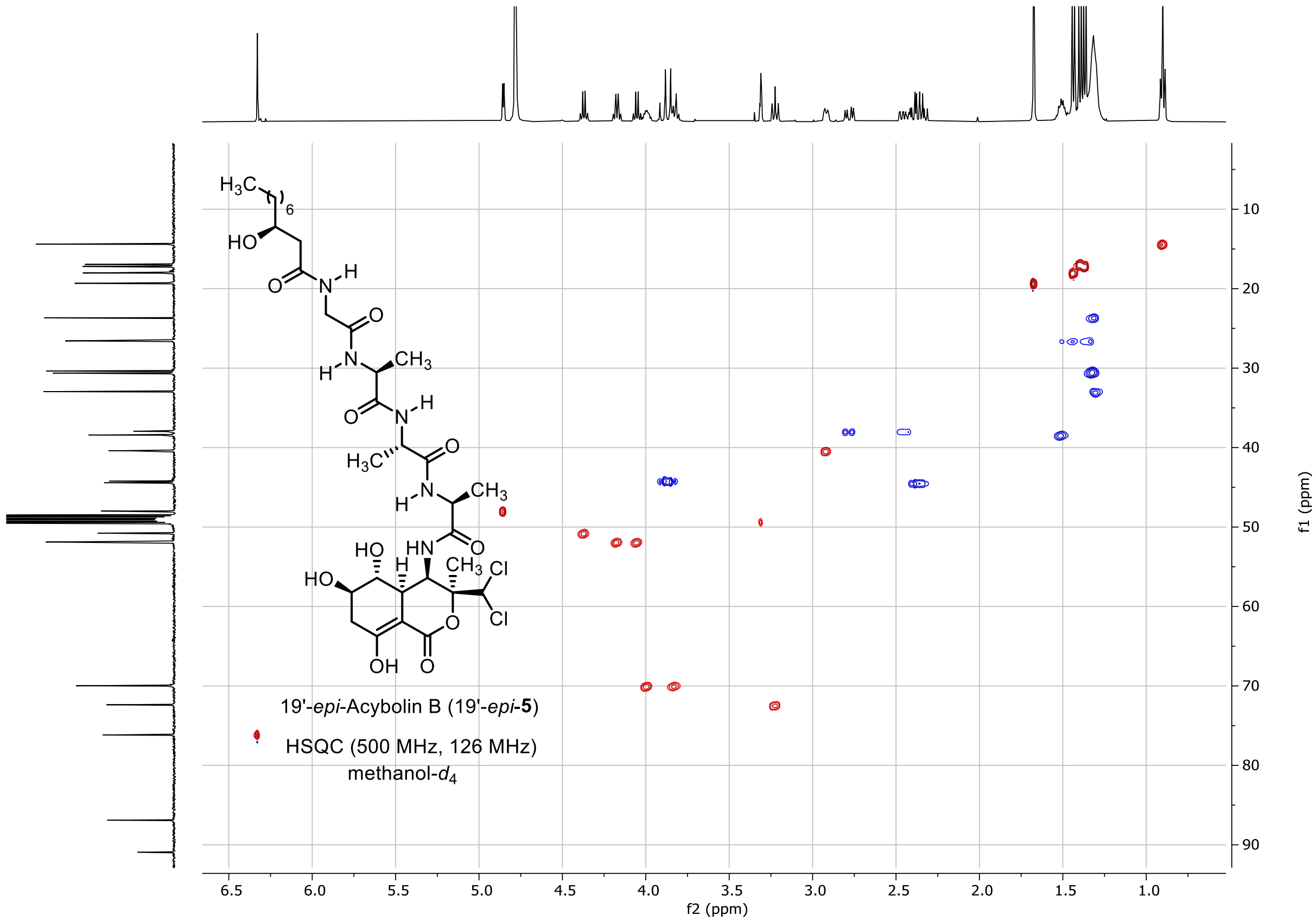
19'-epi-Acybolin B (19'-epi-5)
HSQC (500 MHz, 126 MHz)
methanol-d4
f2 (ppm)
f1 (ppm)

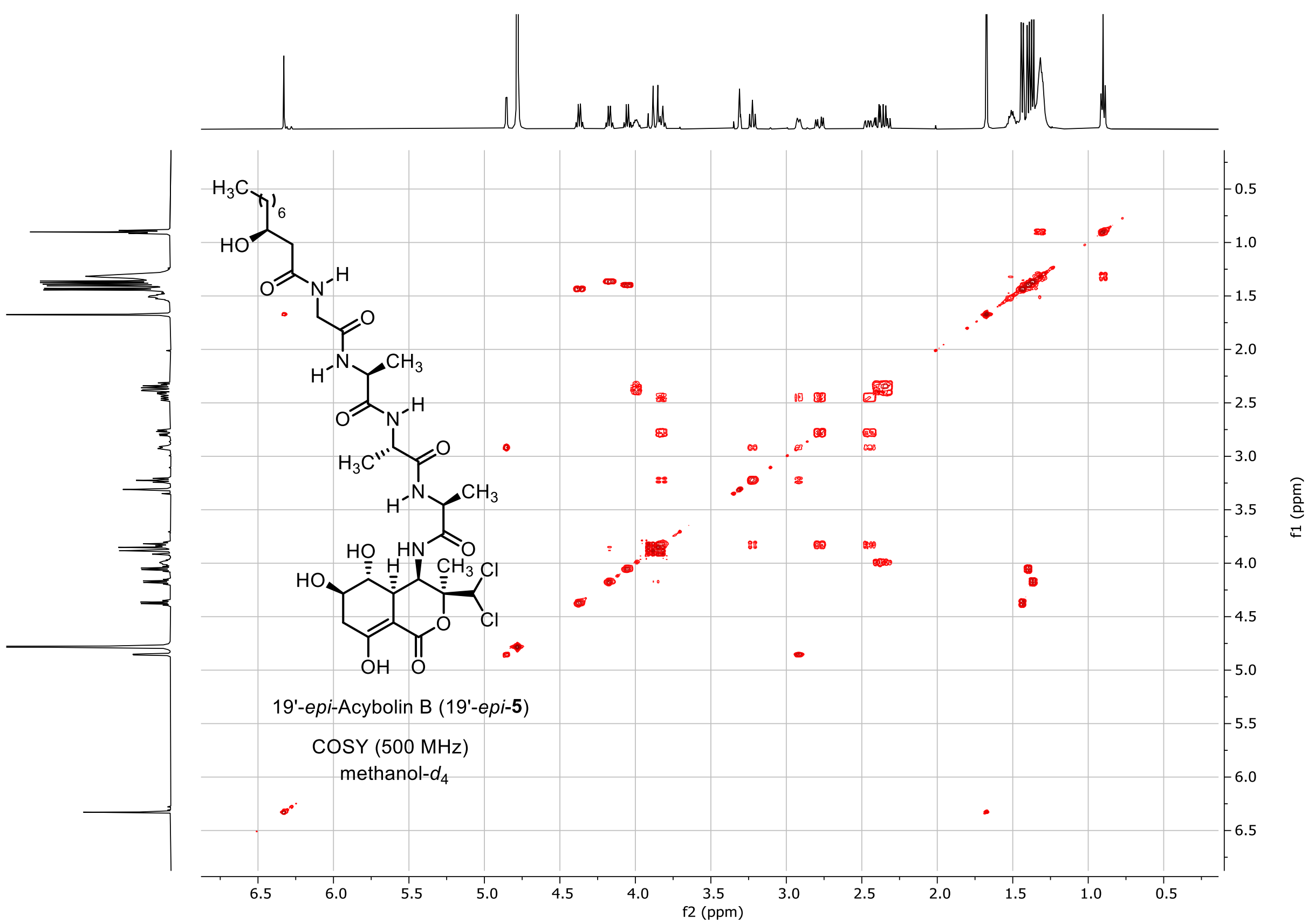
19'-epi-Acybolin B (19'-epi-5)
COSY (500 MHz)
methanol-d4
f2 (ppm)
f1 (ppm)

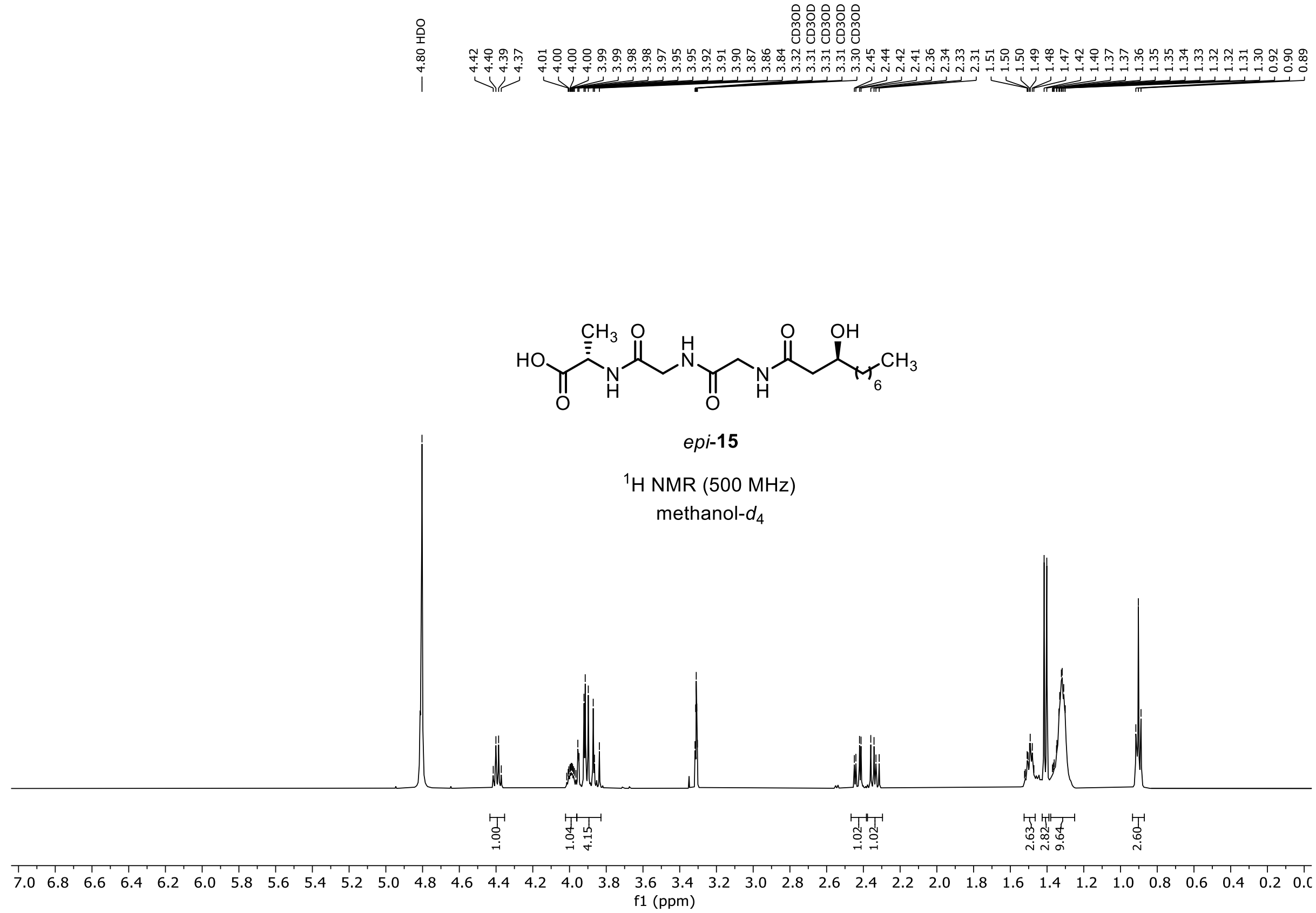
epi-15
^{1}H NMR (500 MHz)
methanol-d_4
f1 (ppm)

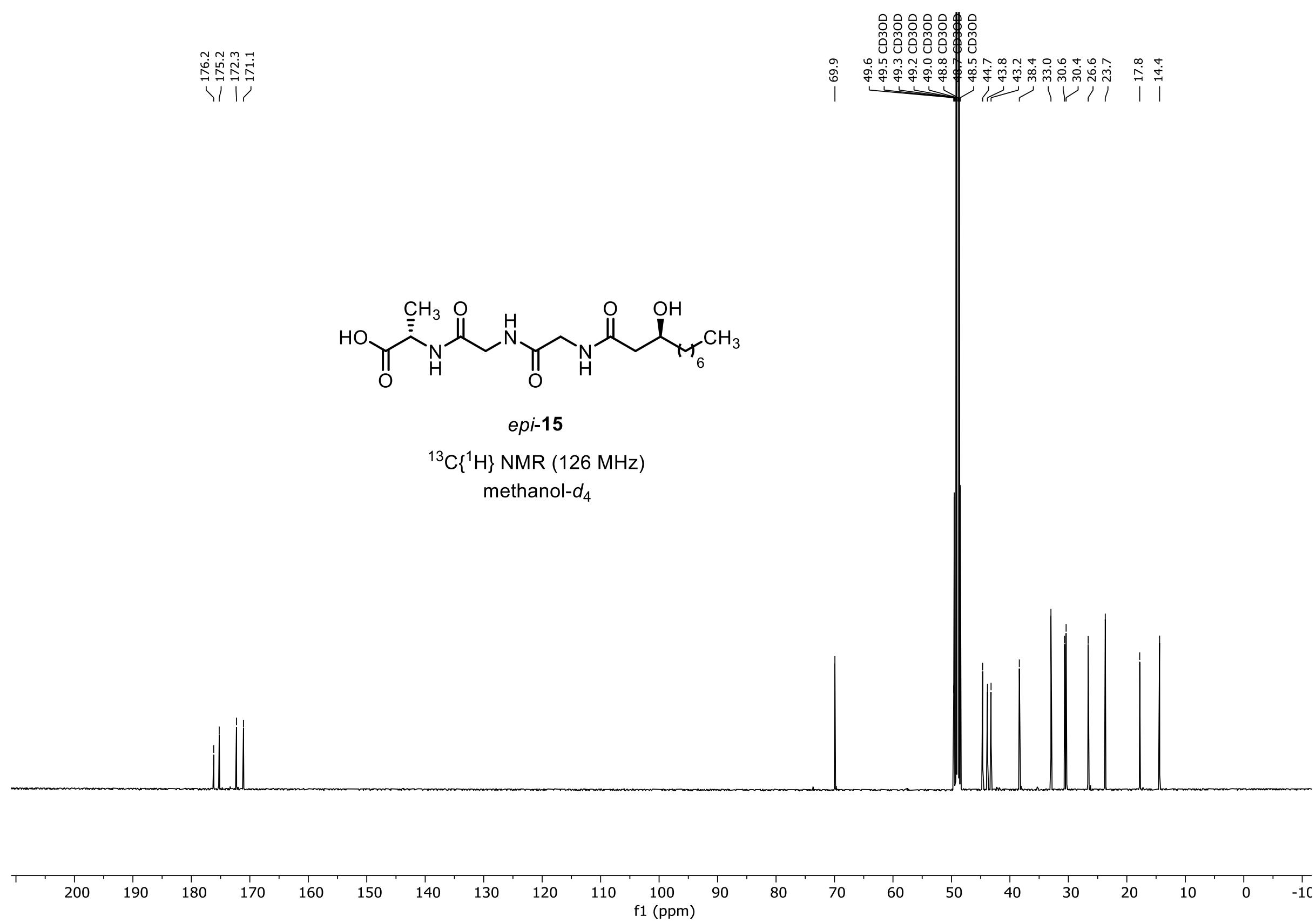
epi-15
^{13}C{^{1}H} NMR (126 MHz)
methanol-d_4
f1 (ppm)

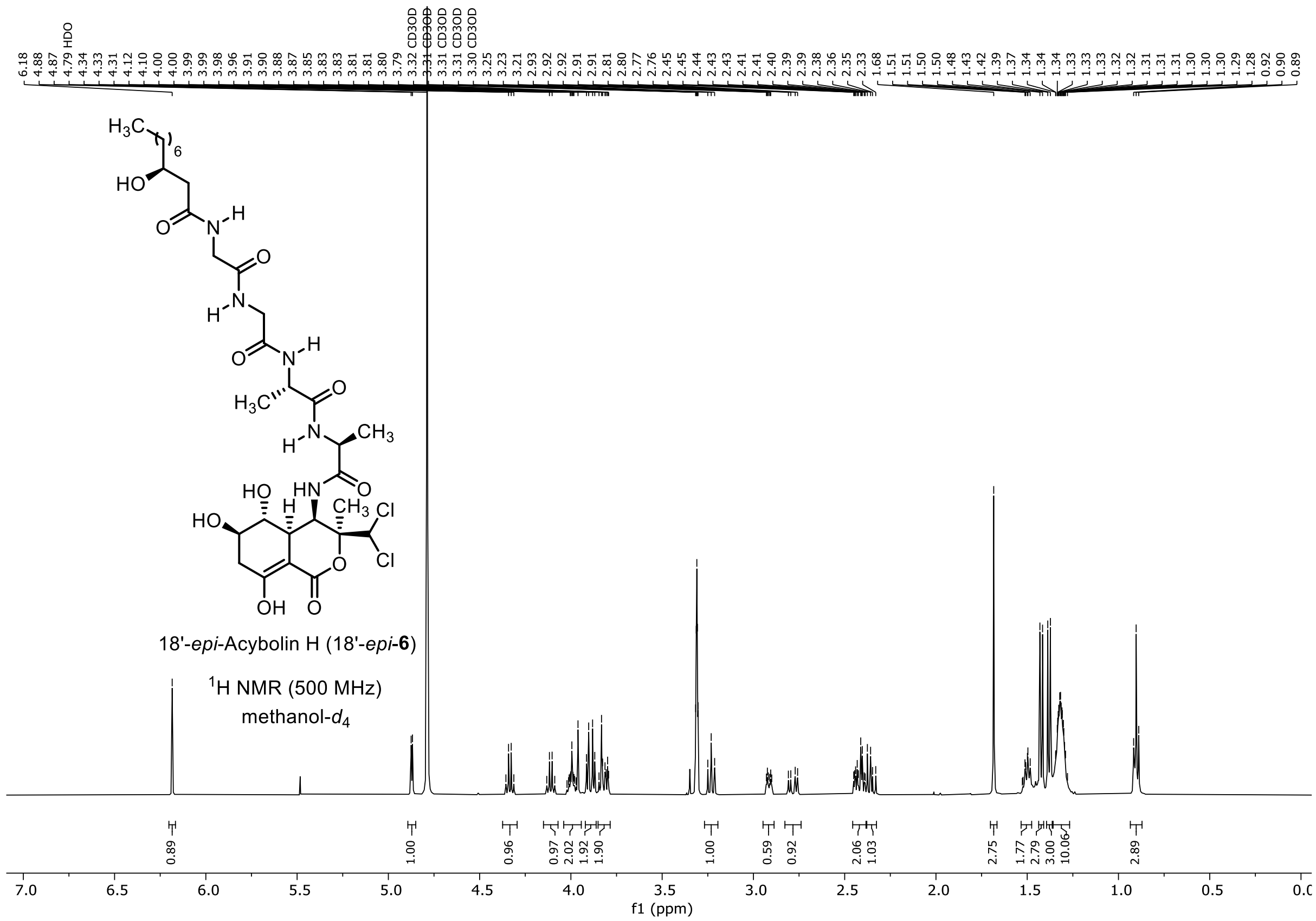
18'-epi-Acybolin H (18'-epi-6)
1H NMR (500 MHz)
methanol-d4
f1 (ppm)

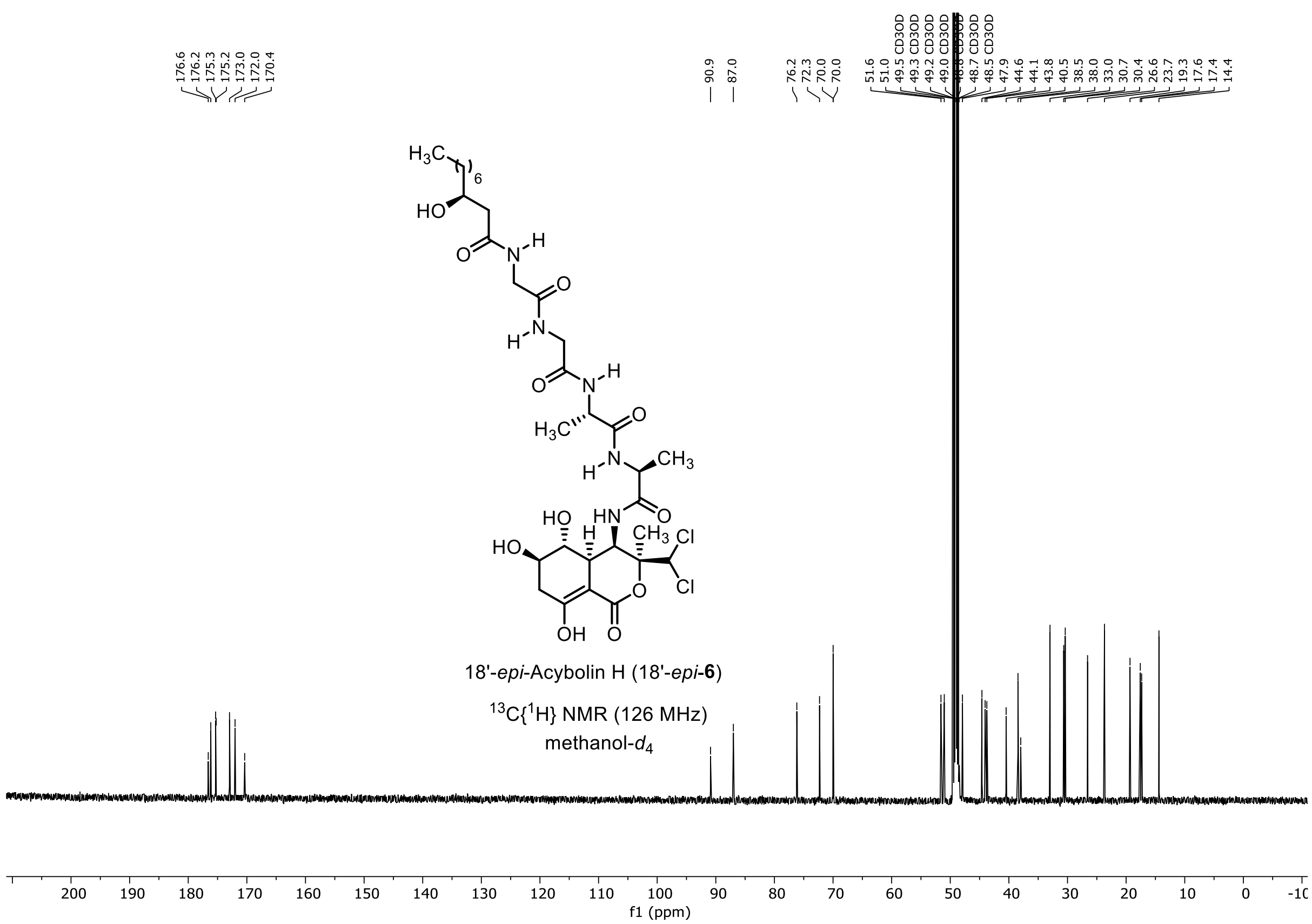
18'-epi-Acybolin H (18'-epi-6)
13C{1H} NMR (126 MHz)
methanol-d4
f1 (ppm)

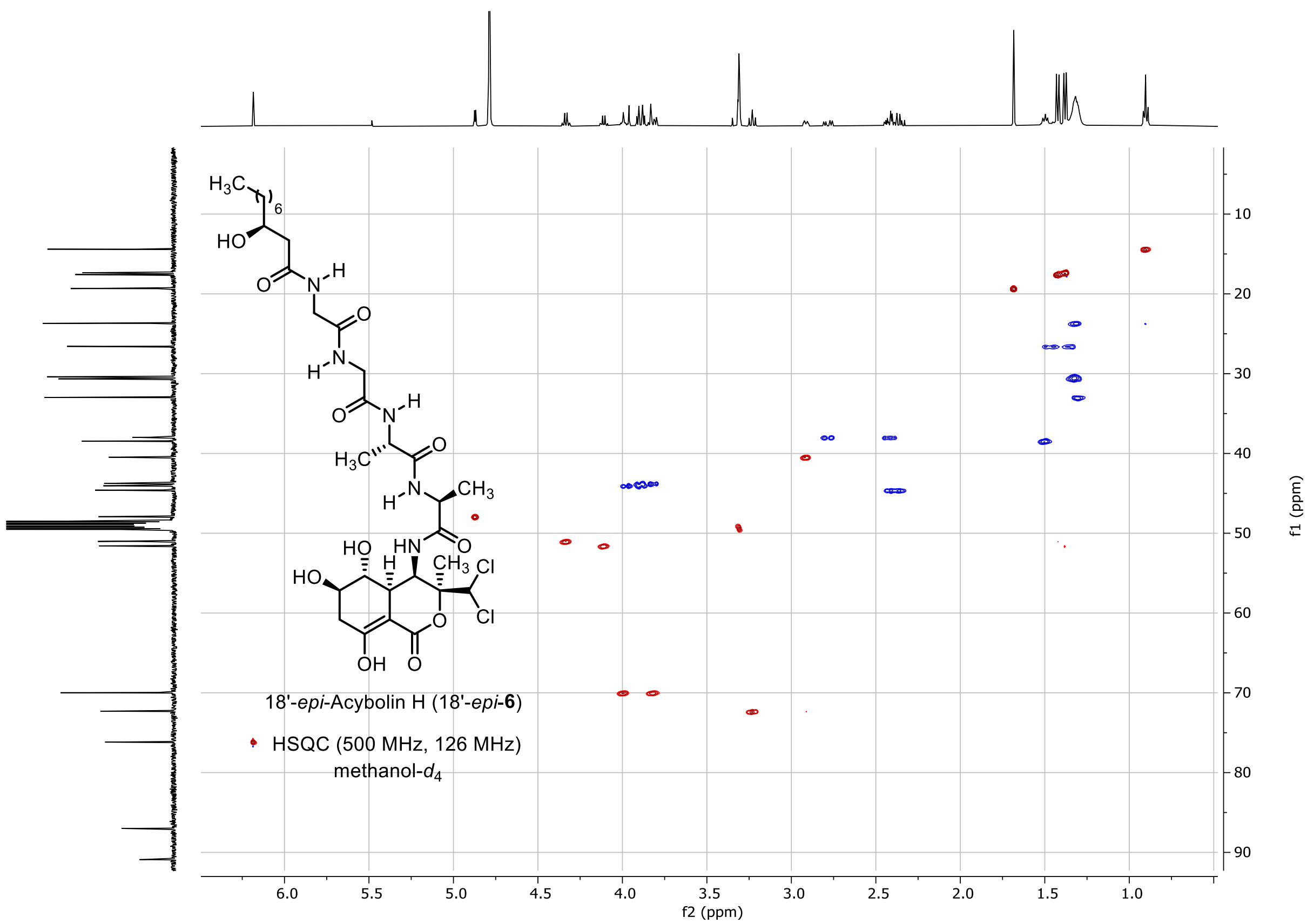
18'-epi-Acybolin H (18'-epi-6)
HSQC (500 MHz, 126 MHz)
methanol-d_4
f2 (ppm)
f1 (ppm)

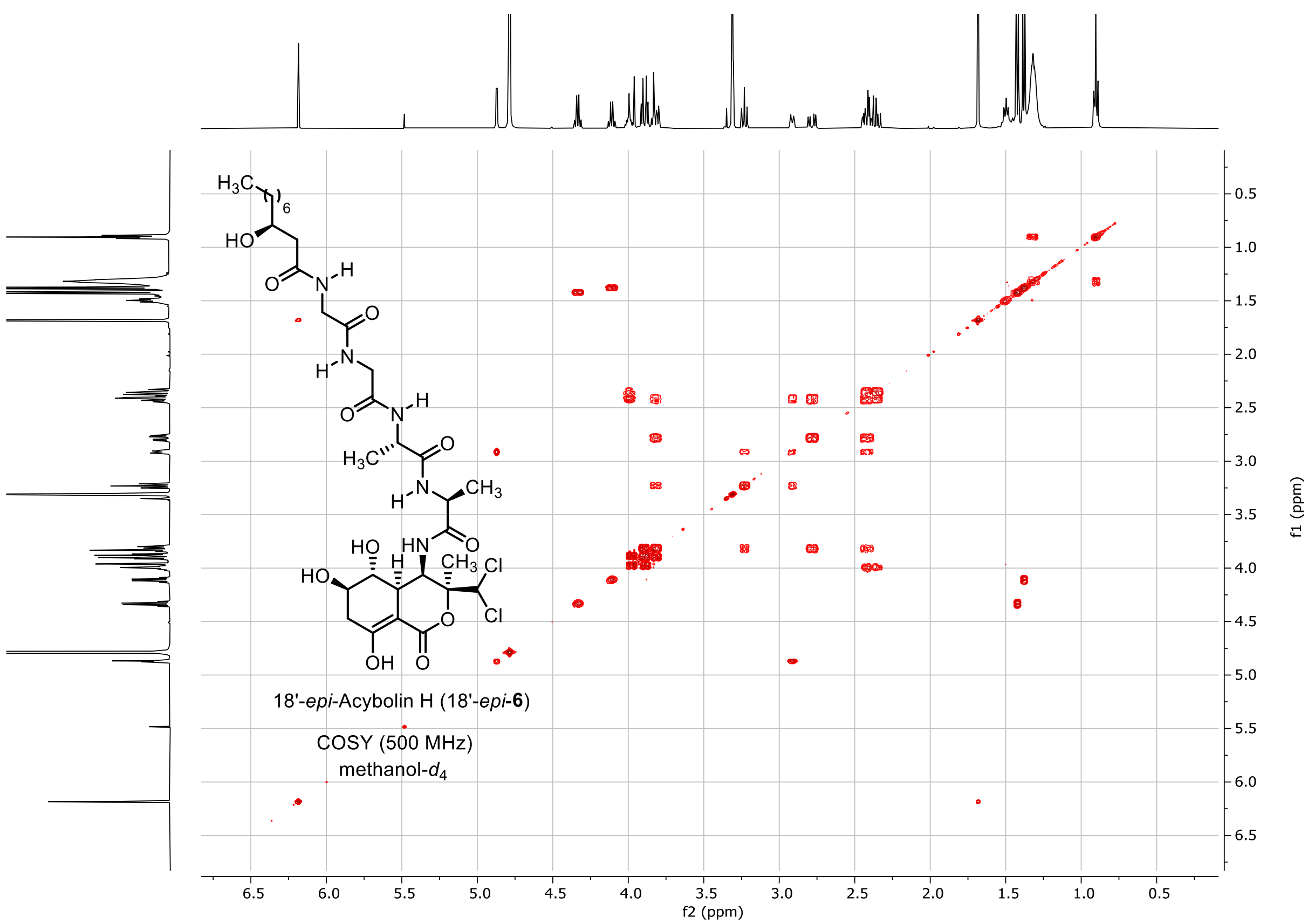
18'-epi-Acybolin H (18'-epi-6)
COSY (500 MHz)
methanol-d_4
f2 (ppm)
f1 (ppm)

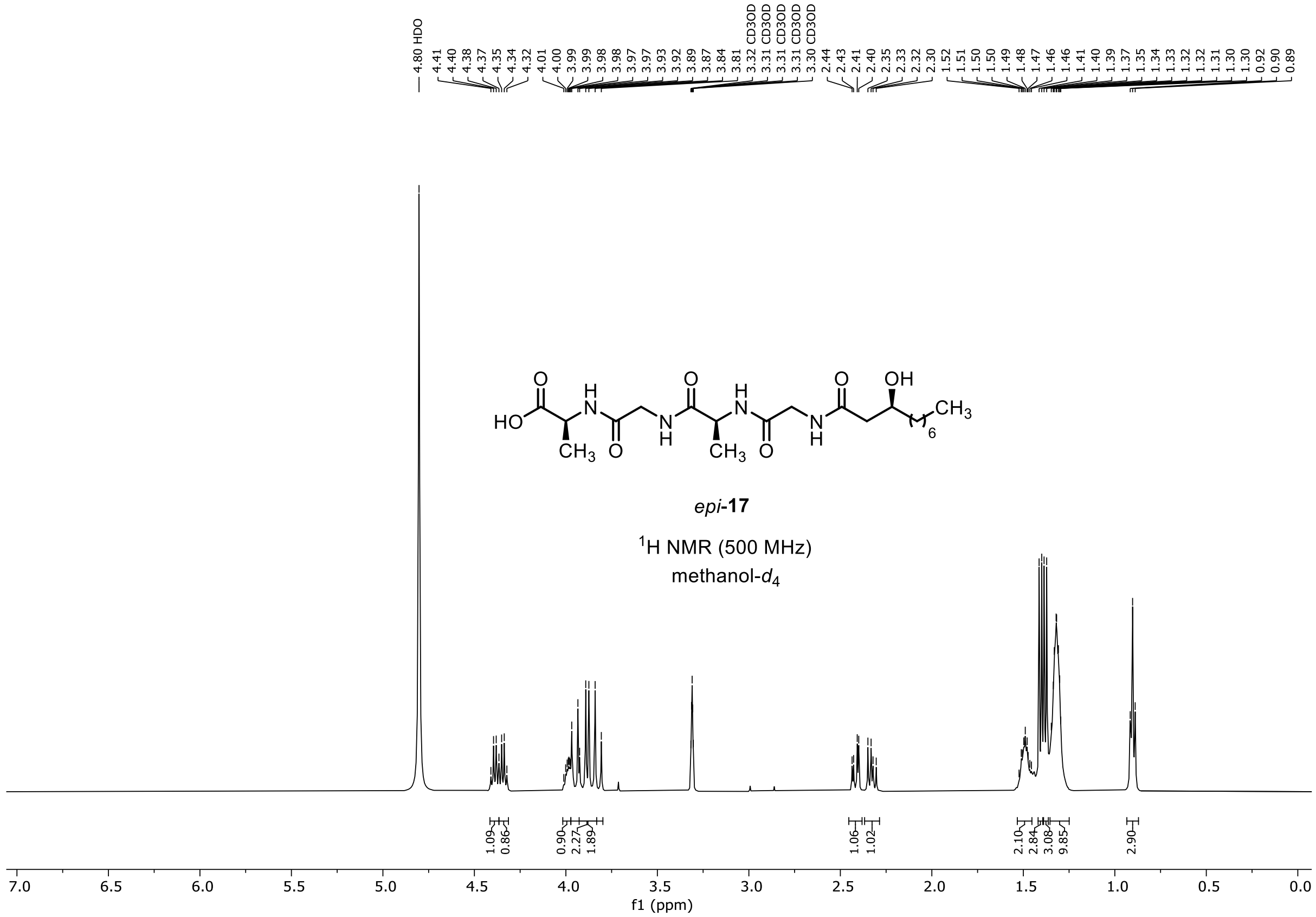

epi-**17**

^1H NMR (500 MHz)

methanol-d_4

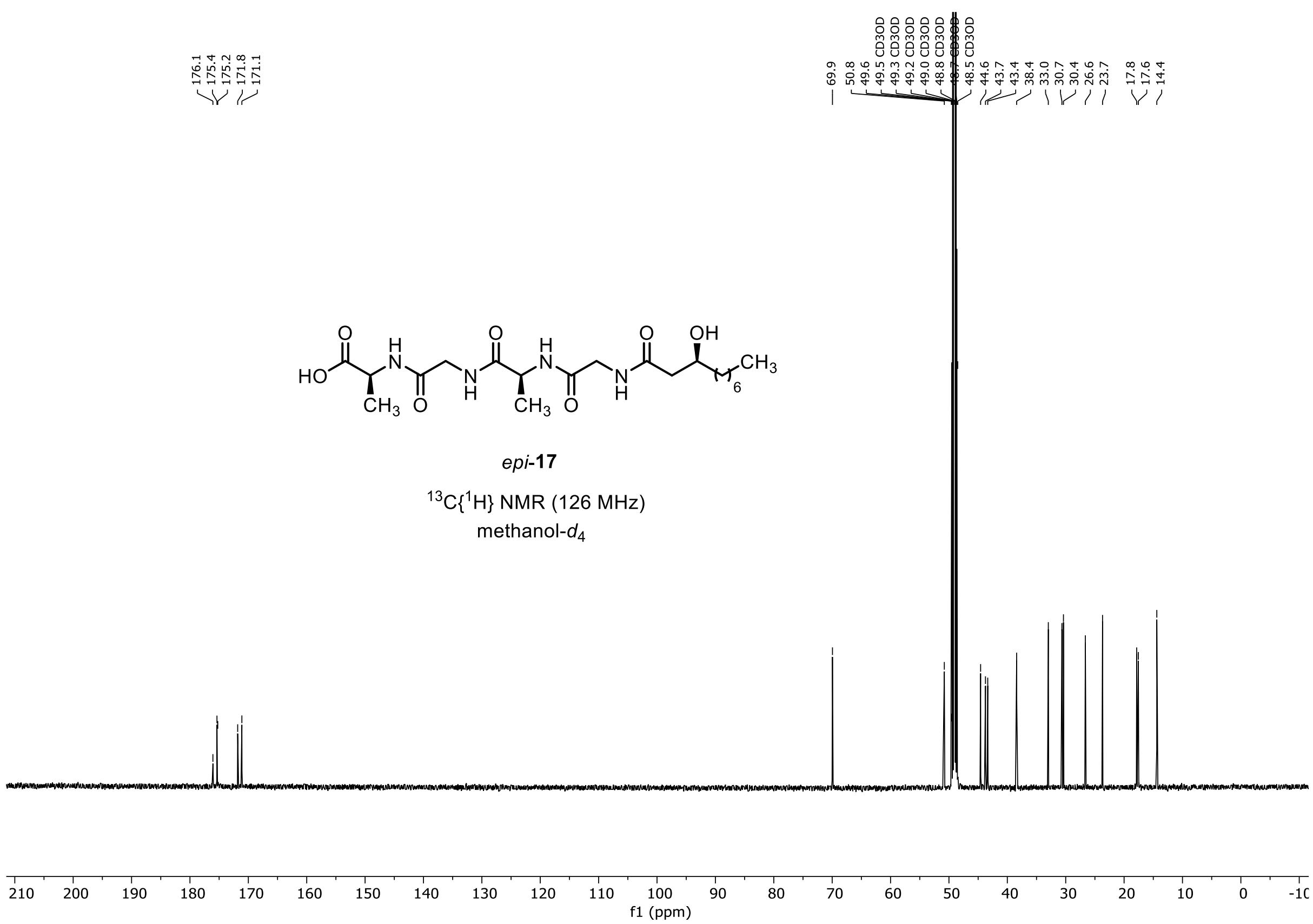

epi-**17**

$^{13}C\{^1H\}$ NMR (126 MHz)

methanol-d_4

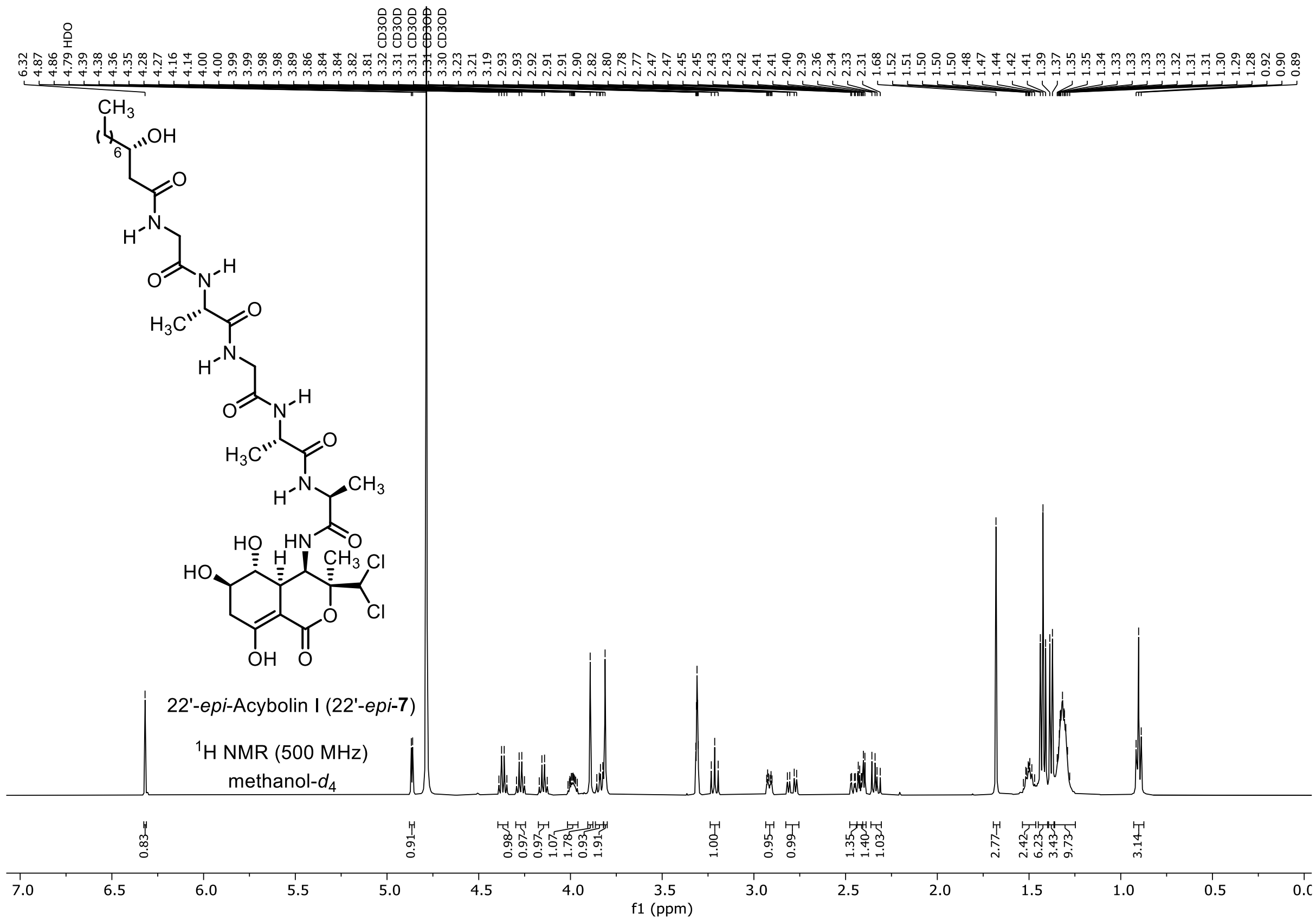
22'-epi-Acybolin I (22'-epi-7)
1H NMR (500 MHz)
methanol-d4
f1 (ppm)

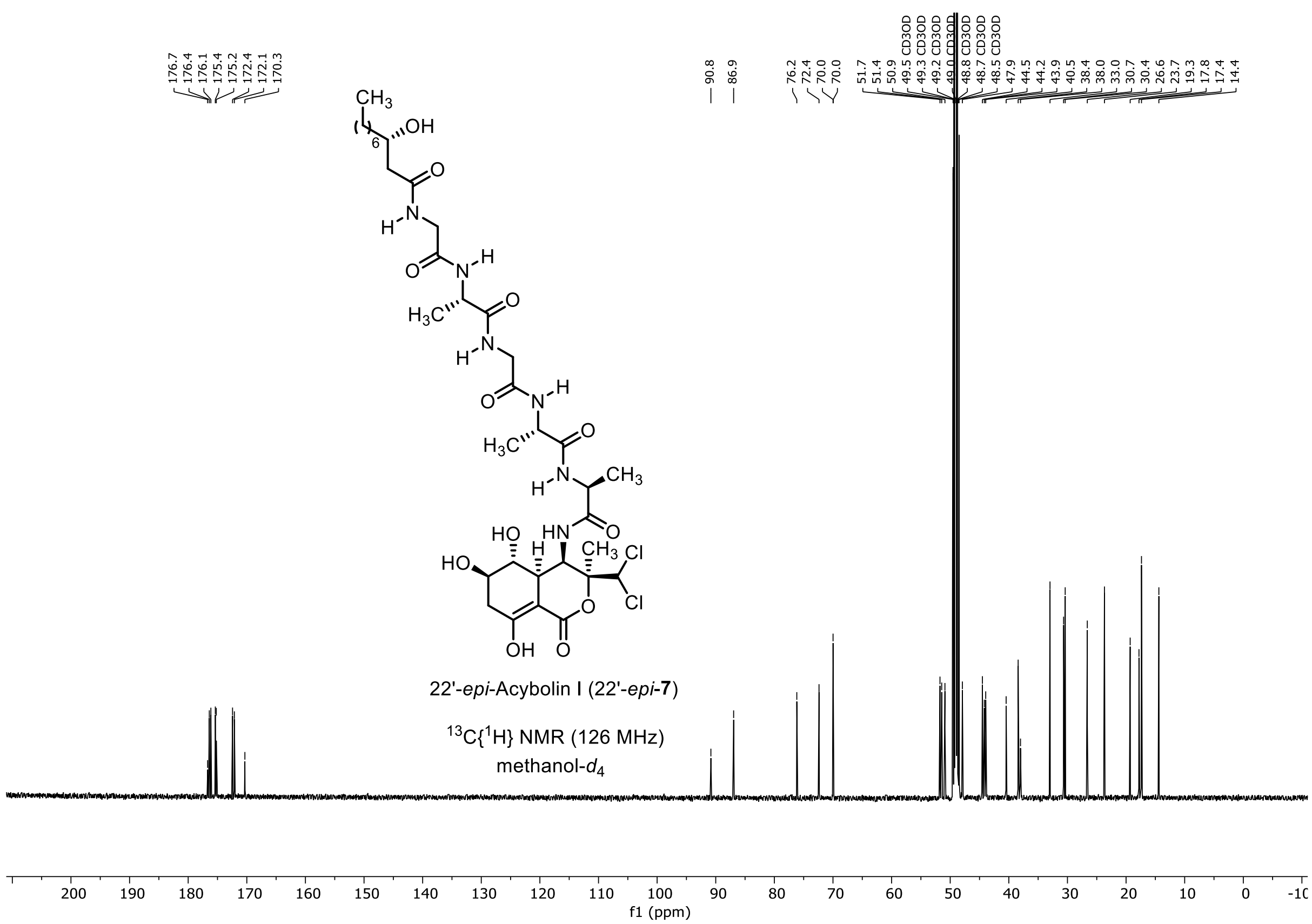
176.7 176.4 176.1 175.4 175.2 172.4 172.1 170.3
90.8
86.9
76.2 72.4 70.0 70.0
51.7 51.4 50.9 49.5 CD3OD 49.3 CD3OD 49.2 CD3OD 49.0 CD3OD 48.8 CD3OD 48.7 CD3OD 48.5 CD3OD 47.9 44.5 44.2 43.9 40.5 38.4 38.0 33.0 30.7 30.4 26.6 23.7 19.3 17.8 17.4 14.4
22'-epi-Acybolin I (22'-epi-7)
13C{1H} NMR (126 MHz)
methanol-d4
f1 (ppm)

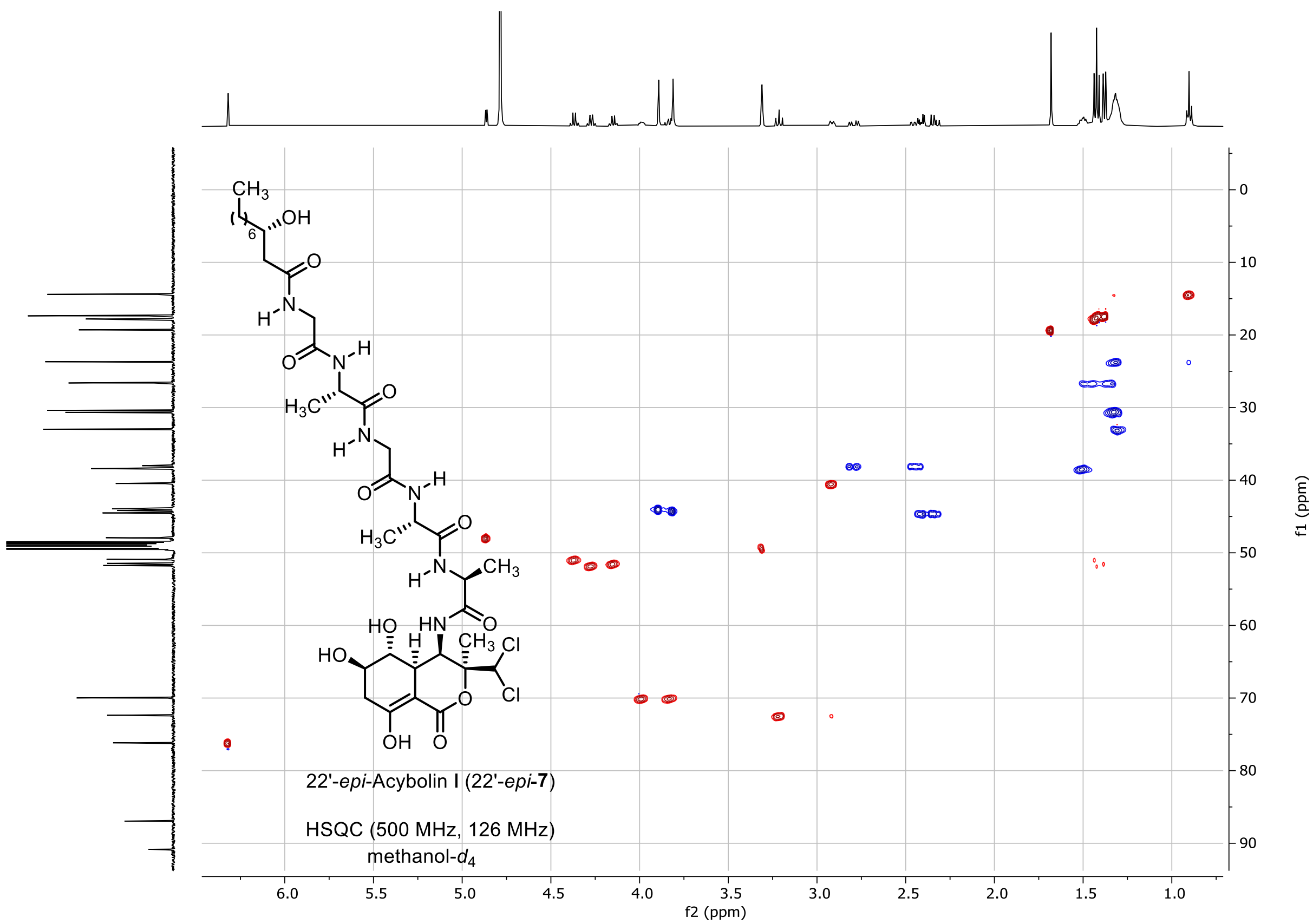
22'-epi-Acybolin I (22'-epi-7)
HSQC (500 MHz, 126 MHz)
methanol-d4
f2 (ppm)
f1 (ppm)

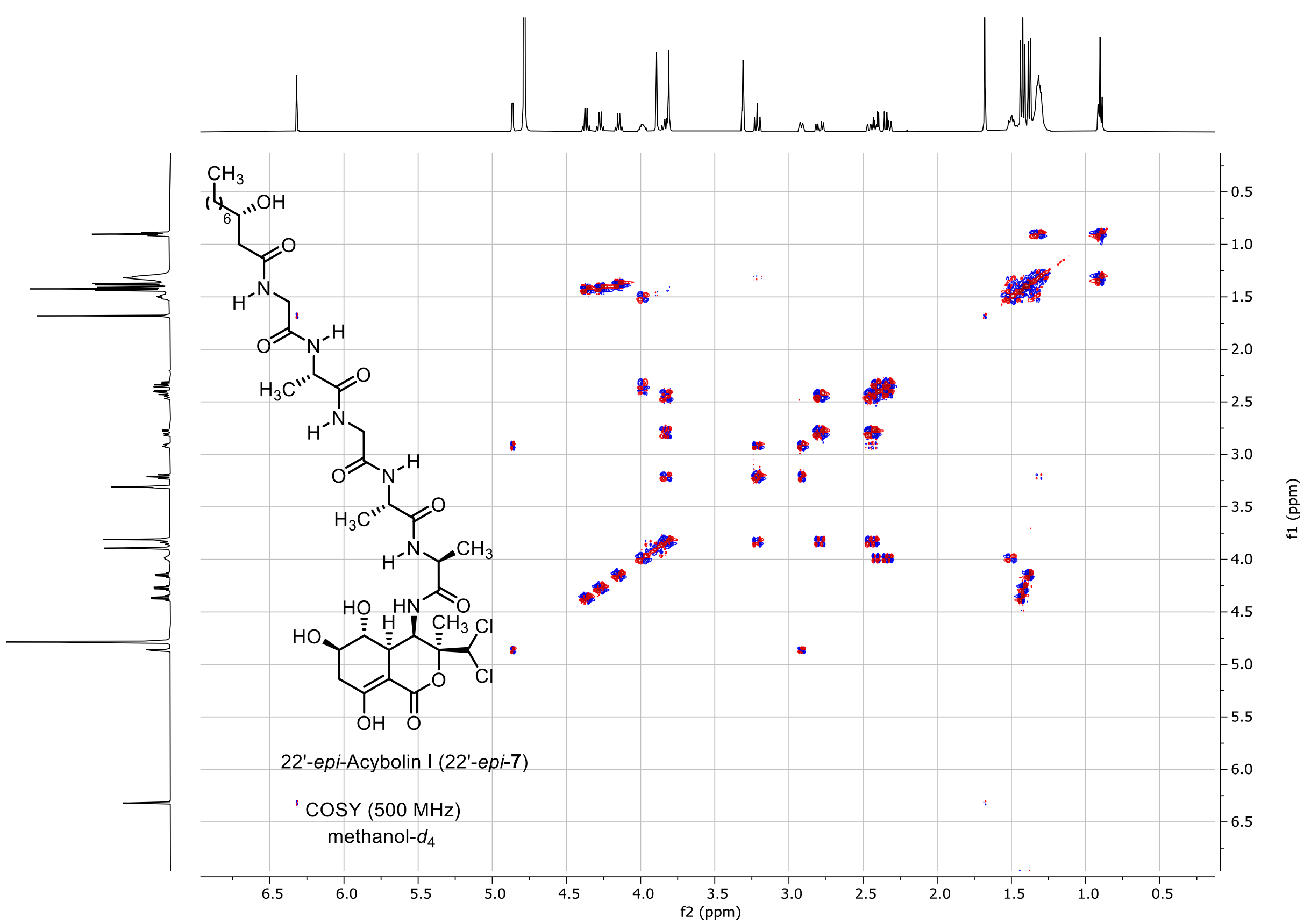
22'-epi-Acybolin I (22'-epi-7)
COSY (500 MHz)
methanol-d4
f2 (ppm)
f1 (ppm)

NMR SPECTRA OF BACTOBOLIN E

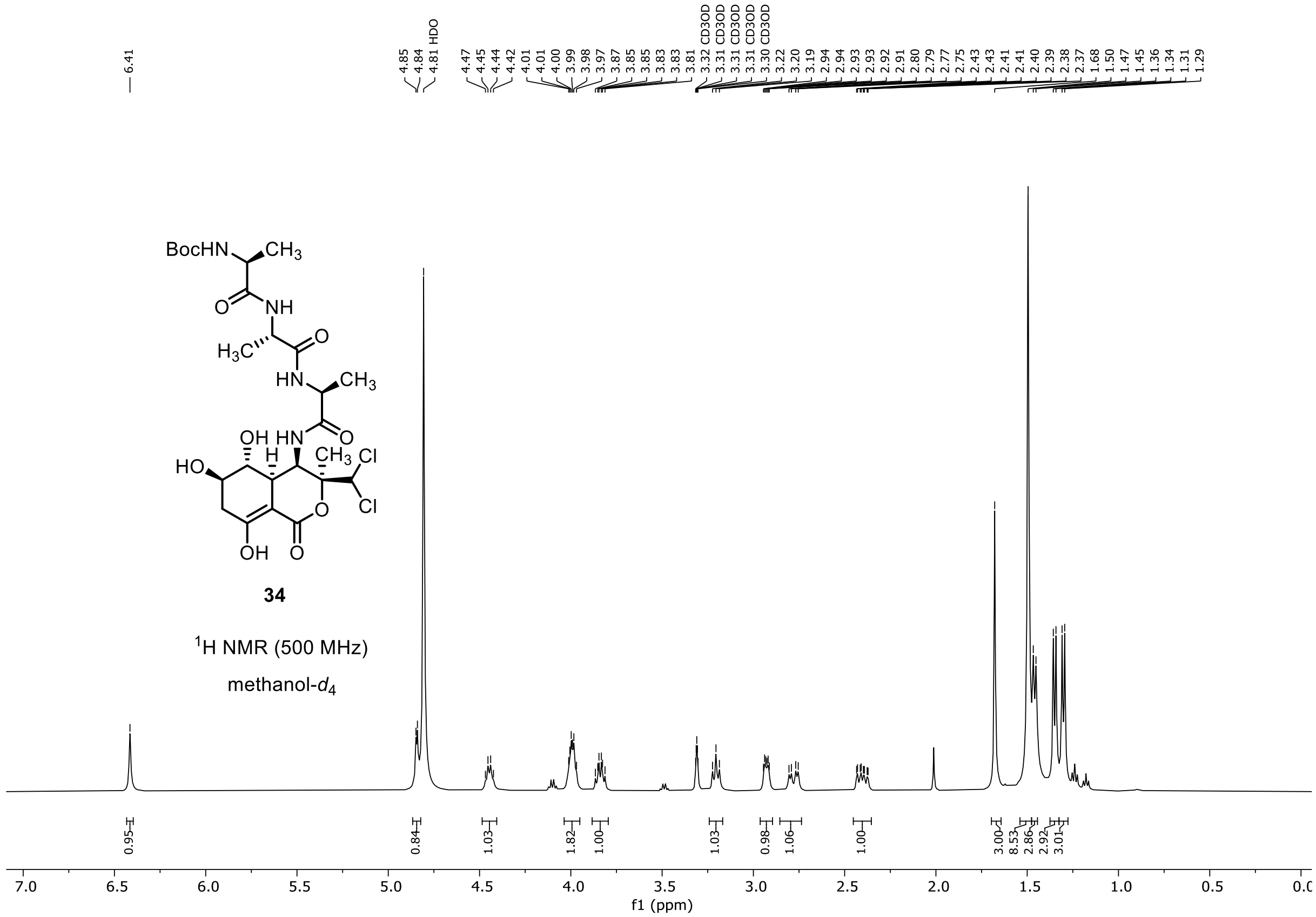

BocHN
CH3
O
NH
H3C
O
HN
CH3
OH
HN
O
HO
H
CH3
Cl
Cl
O
OH
O
34
1H NMR (500 MHz)
methanol-d4
f1 (ppm)

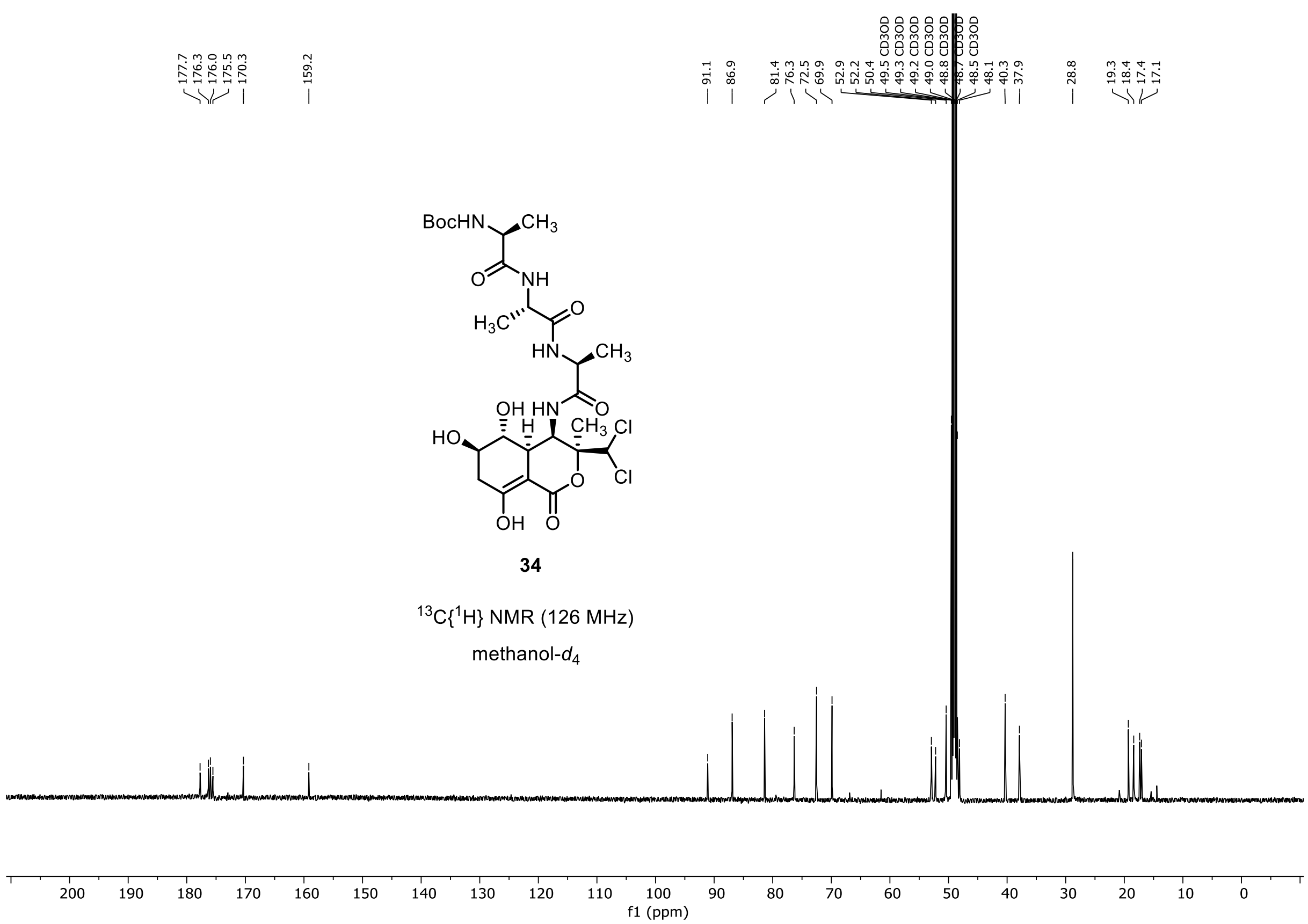

177.7
176.3
176.0
175.5
170.3
159.2
91.1
86.9
81.4
76.3
72.5
69.9
52.9
52.2
50.4
28.8
19.3
18.4
17.4
17.1
34
13C{1H} NMR (126 MHz)
methanol-d4
f1 (ppm)

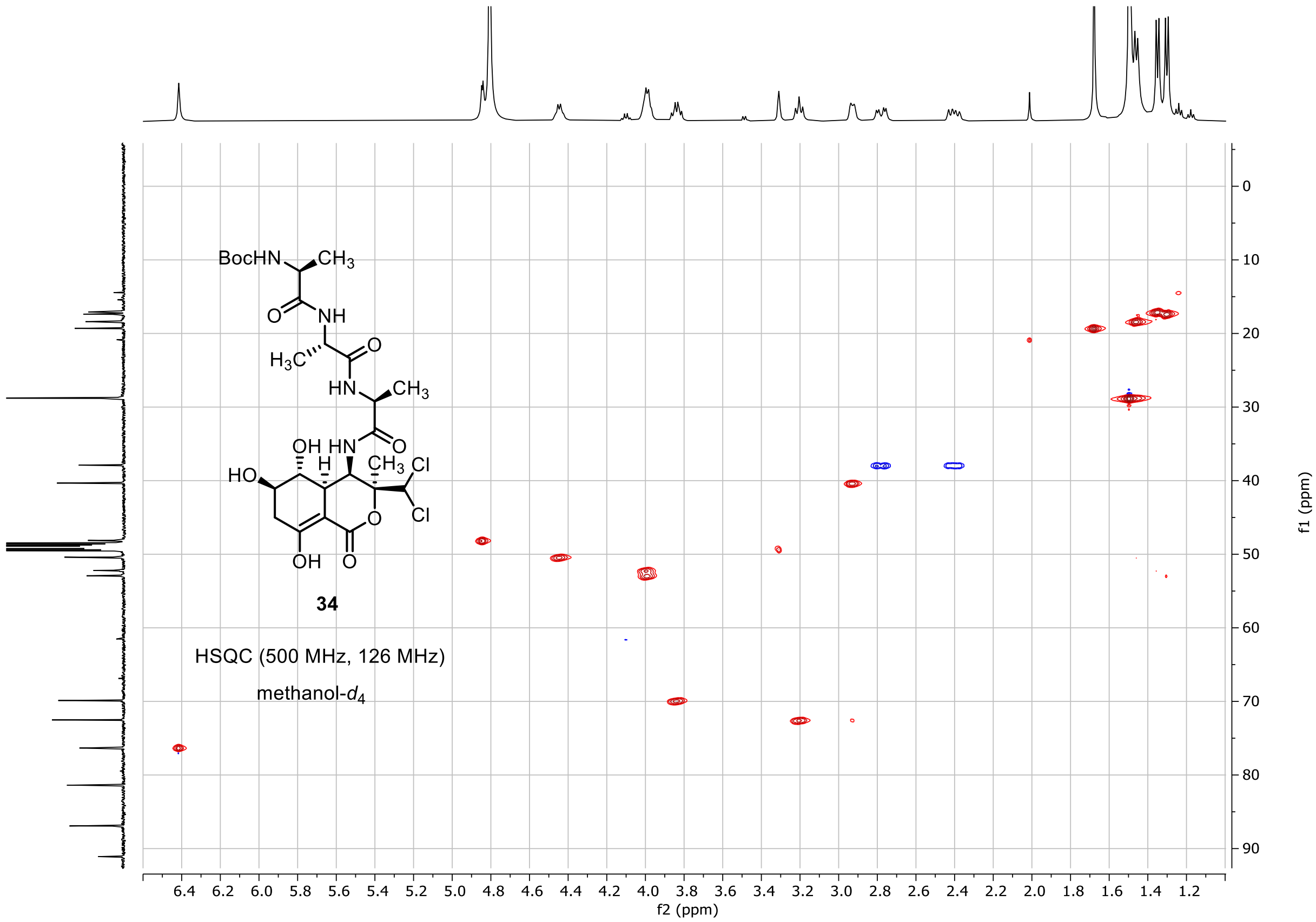
BocHN
CH3
O
NH
H3C
O
HN
CH3
OH HN
HO
H
CH3 Cl
Cl
O
O
OH
34
HSQC (500 MHz, 126 MHz)
methanol-d4
f2 (ppm)
f1 (ppm)

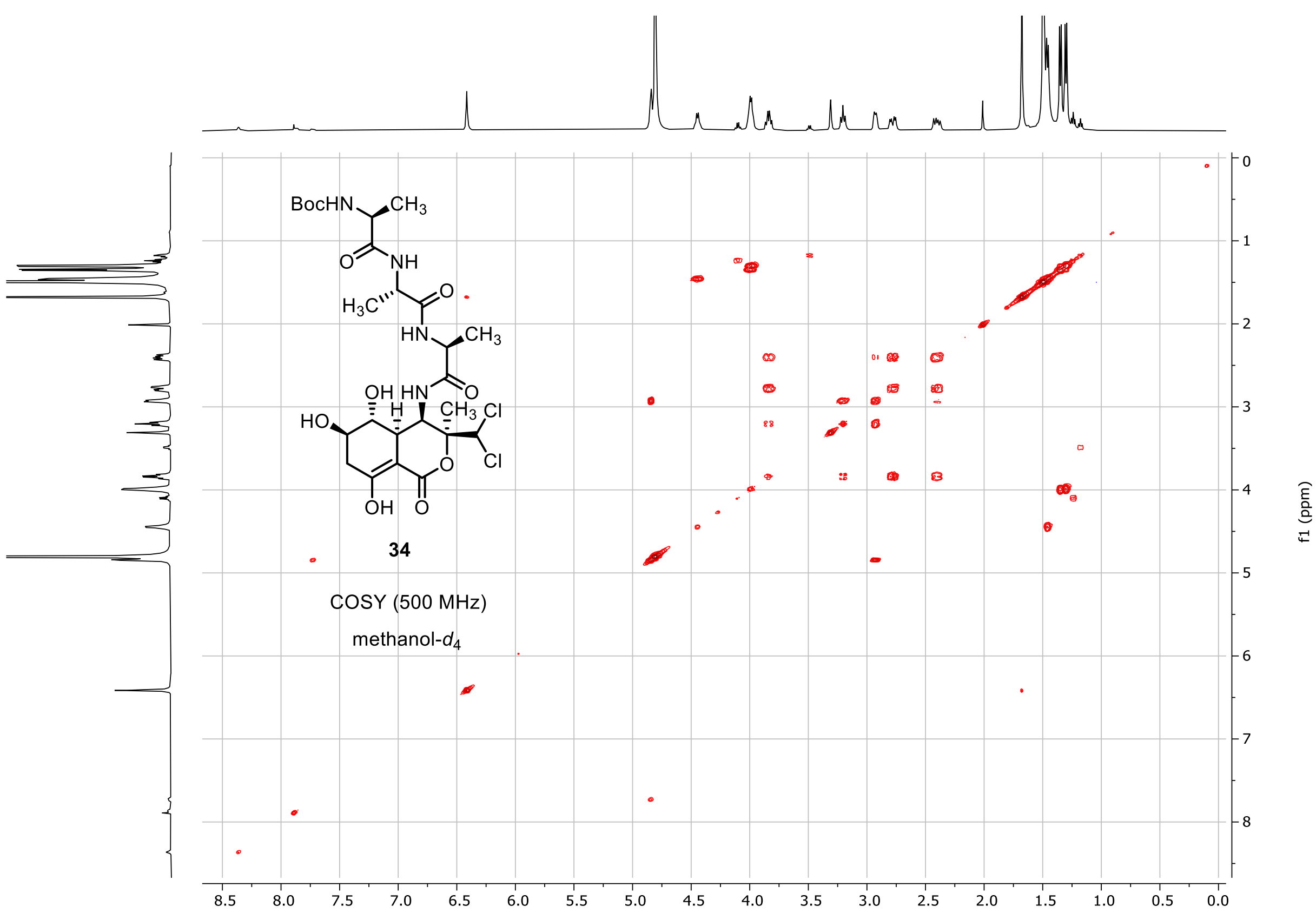
BocHN
CH3
O
NH
H3C
O
HN
CH3
OH HN
HO
H
CH3 Cl
Cl
O
O
OH
34
COSY (500 MHz)
methanol-d4
f2 (ppm)
f1 (ppm)

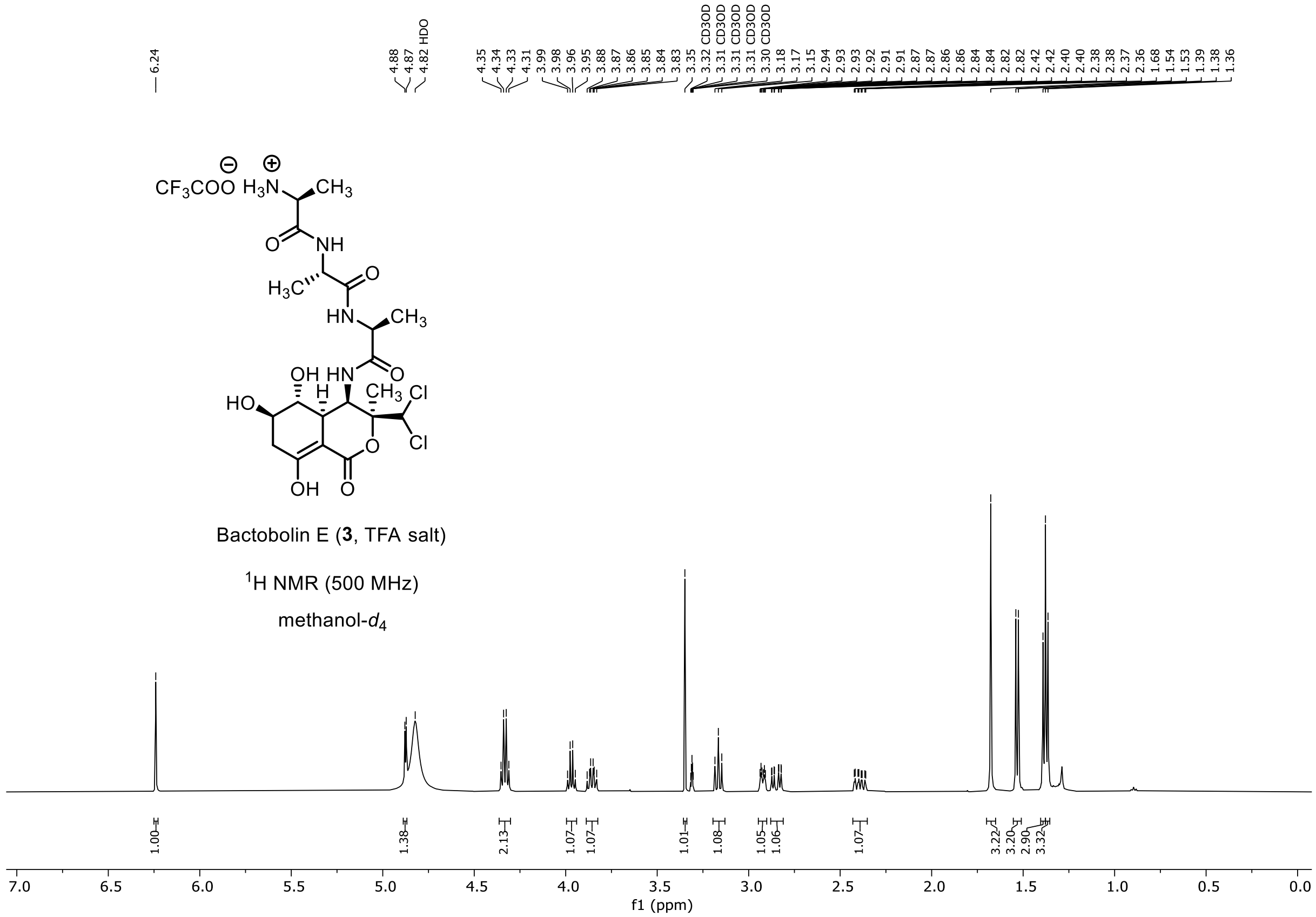
6.24
4.88
4.87
4.82 HDO
4.35
4.34
4.33
4.31
3.99
3.98
3.96
3.95
3.88
3.87
3.86
3.85
3.84
3.83
3.35
3.32 CD3OD
3.31 CD3OD
3.31 CD3OD
3.31 CD3OD
3.30 CD3OD
3.18
3.17
3.15
2.94
2.93
2.93
2.92
2.91
2.91
2.87
2.87
2.86
2.86
2.84
2.84
2.82
2.82
2.42
2.42
2.40
2.40
2.38
2.38
2.37
2.36
1.68
1.54
1.53
1.39
1.38
1.36
Bactobolin E (3, TFA salt)
1H NMR (500 MHz)
methanol-d4
1.00
1.38
2.13
1.07
1.07
1.01
1.08
1.05
1.06
1.07
3.22
3.20
2.90
3.32
f1 (ppm)

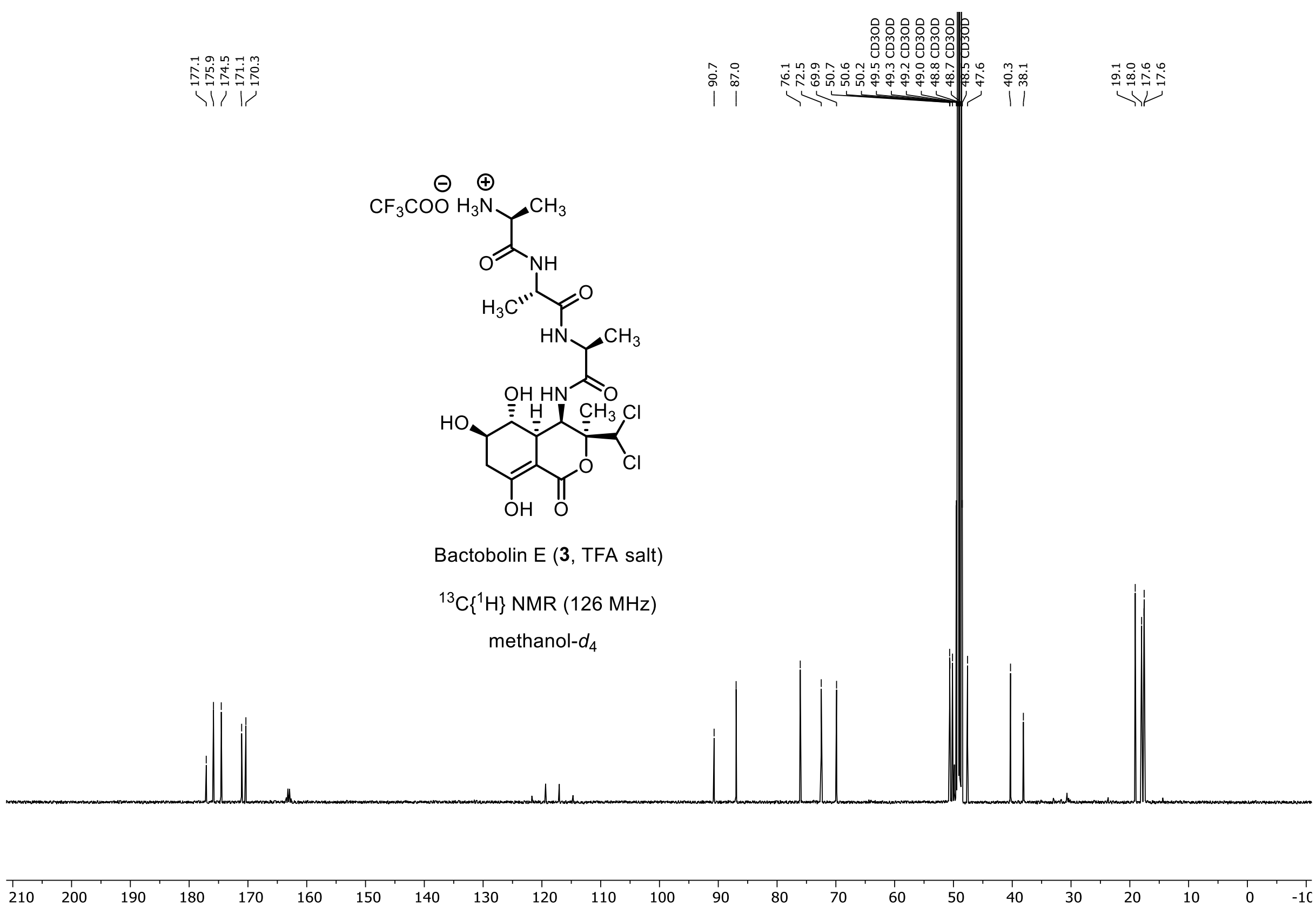
177.1
175.9
174.5
171.1
170.3
90.7
87.0
76.1
72.5
69.9
50.7
50.6
50.2
49.5 CD3OD
49.3 CD3OD
49.2 CD3OD
49.0 CD3OD
48.8 CD3OD
48.7 CD3OD
48.5 CD3OD
47.6
40.3
38.1
19.1
18.0
17.6
17.6
Bactobolin E (3, TFA salt)
13C{1H} NMR (126 MHz)
methanol-d4
f1 (ppm)

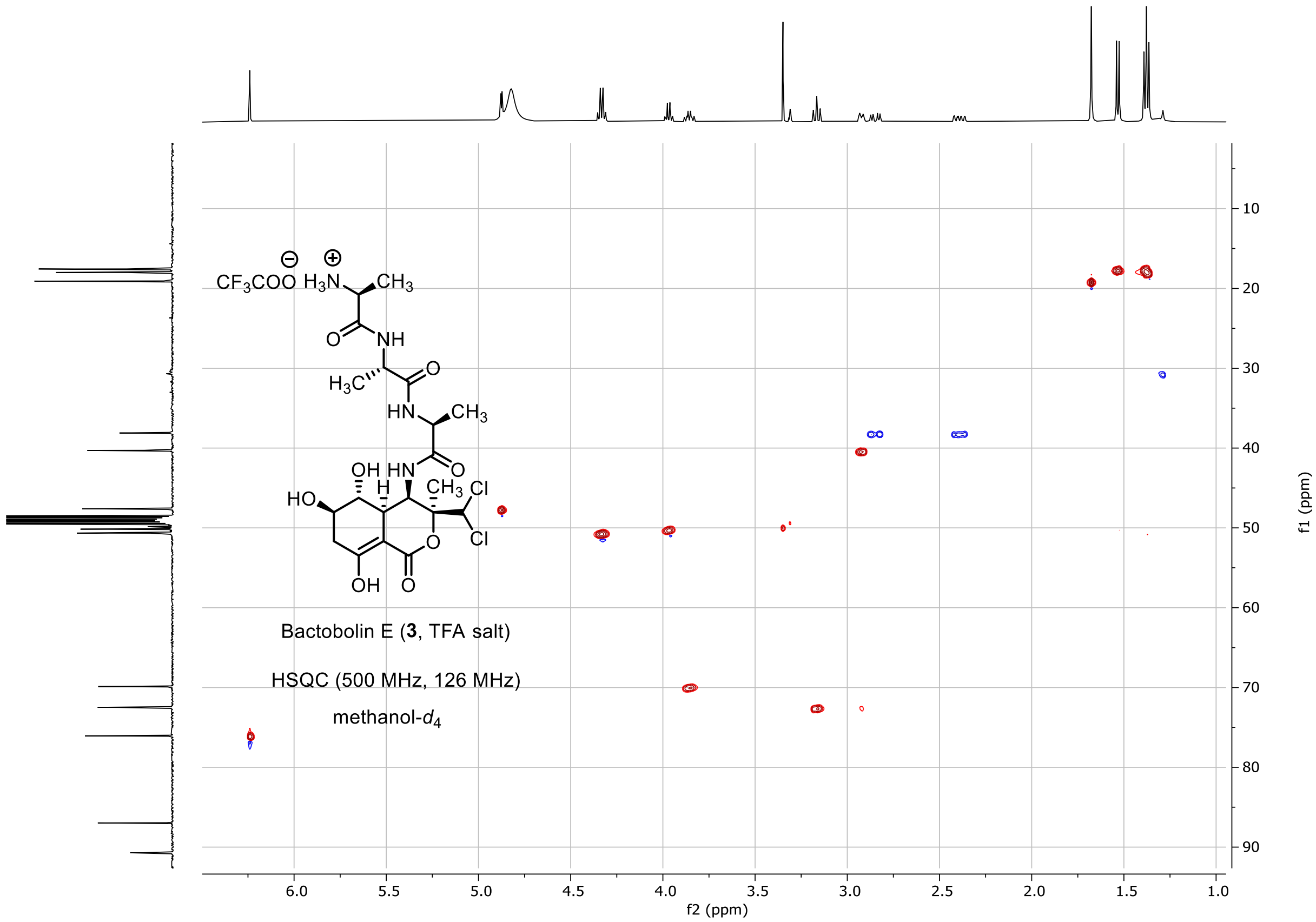

CF3COO H3N CH3
O NH
H3C O
HN CH3
OH HN O
HO H CH3 Cl
Cl
OH O
Bactobolin E (3, TFA salt)
HSQC (500 MHz, 126 MHz)
methanol-d4
f2 (ppm)
f1 (ppm)

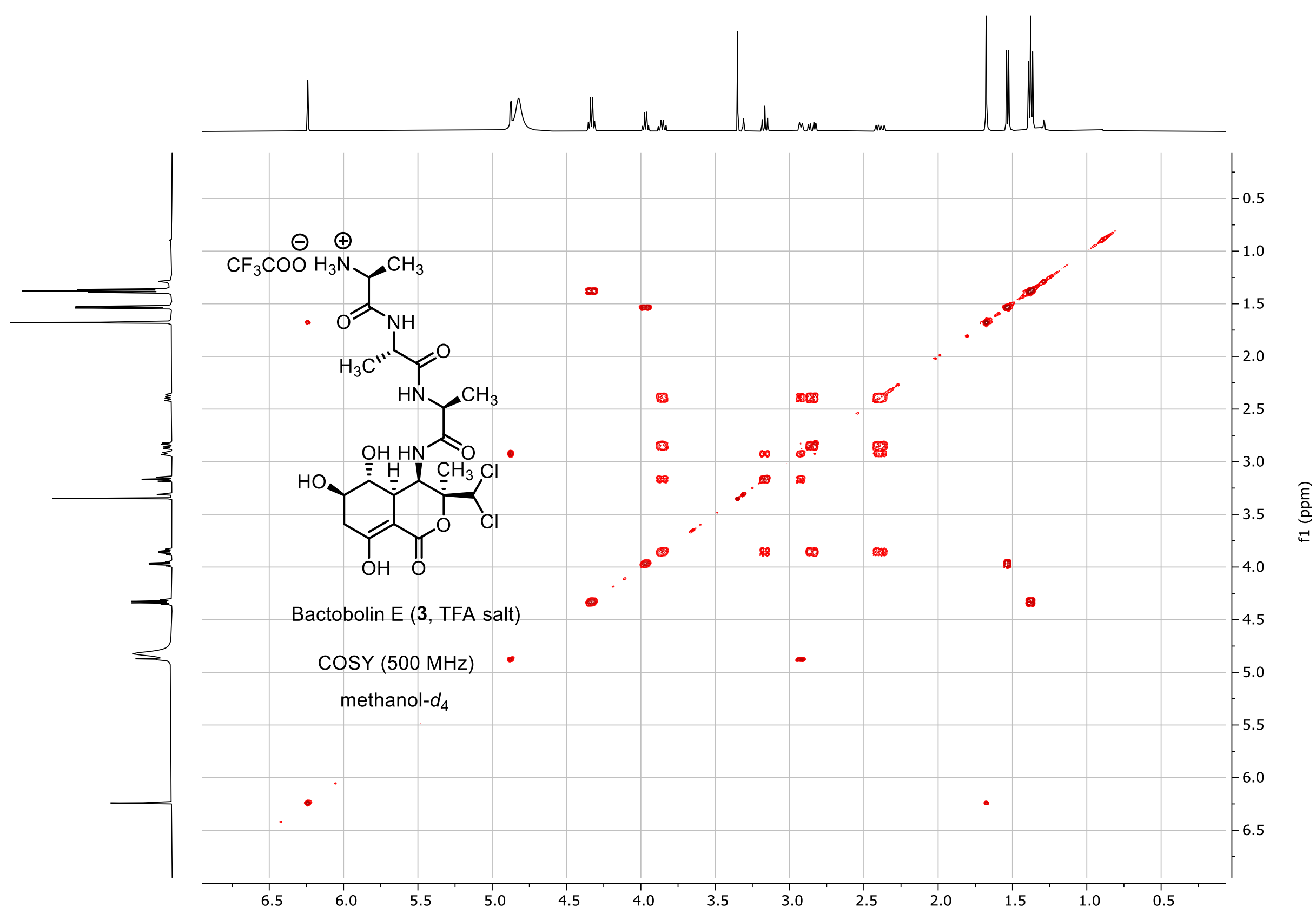

CF3COO H3N CH3
O NH
H3C O
HN CH3
OH HN O
HO H CH3 Cl
Cl
OH O
Bactobolin E (3, TFA salt)
COSY (500 MHz)
methanol-d4
f2 (ppm)
f1 (ppm)

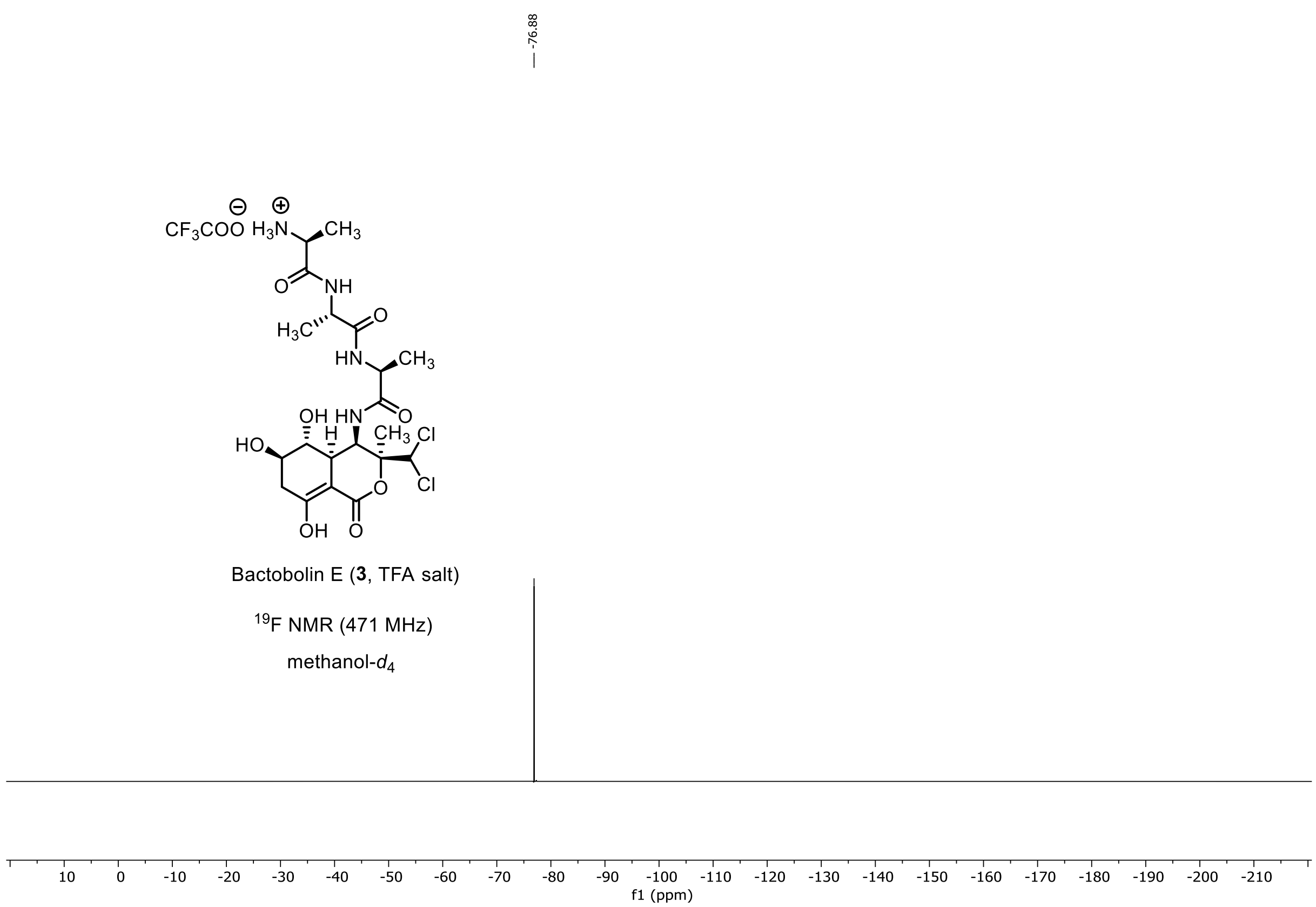

Bactobolin E (**3**, TFA salt)

^{19}F NMR (471 MHz)

methanol-d_4
